# Triple Resonance Experiments for the Rapid Detection of ^103^Rh NMR Shifts: A Combined Experimental and Theoretical Study into Dirhodium and Bismuth–Rhodium Paddlewheel Complexes

**DOI:** 10.1021/jacs.1c06414

**Published:** 2021-08-05

**Authors:** Fabio P. Caló, Giovanni Bistoni, Alexander A. Auer, Markus Leutzsch, Alois Fürstner

**Affiliations:** † Max-Planck-Institut für Kohlenforschung, 45470 Mülheim, Germany

## Abstract

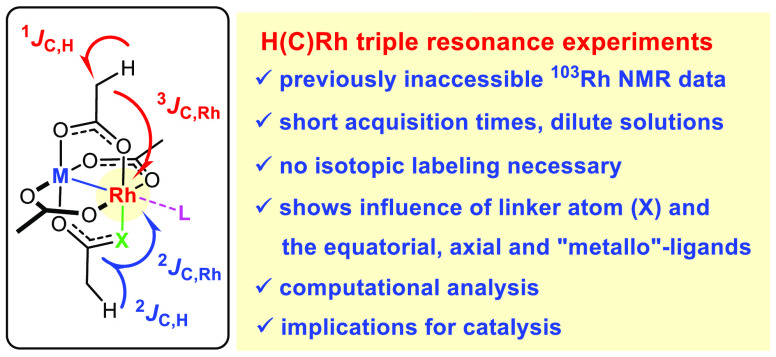

A H(C)Rh triple resonance NMR experiment makes the rapid detection of ^103^Rh chemical shifts possible, which were previously beyond reach. It served to analyze a series of dirhodium and bismuth–rhodium paddlewheel complexes of the utmost importance for metal–carbene chemistry. The excellent match between the experimental and computed ^103^Rh shifts in combination with a detailed analysis of the pertinent shielding tensors forms a sound basis for a qualitative and quantitative interpretation of these otherwise (basically) inaccessible data. The observed trends clearly reflect the influence exerted by the equatorial ligands (carboxylate versus carboxamidate), the axial ligands (solvents), and the internal “metalloligand” (Rh versus Bi) on the electronic estate of the reactive Rh(II) center.

For a shift range on the order of 12.000 ppm, ^103^Rh NMR spectroscopy is, a priori, a cardinal tool to probe the electronic nature of a given rhodium complex. It allows even small electronic and geometric changes in the coordination sphere to be detected, which are difficult, if not even impossible, to assess otherwise.^1−4^ Its exceptional responsiveness to the chemical environment notwithstanding, ^103^Rh NMR is not nearly as routinely used by practitioners as one might assume. An extremely low gyromagnetic ratio in combination with often unduly long relaxation times^5^ offsets the inherent advantages of ^103^Rh as an *I* = 1/2 nucleus of 100% natural abundance.^6−8^ 2D inverse detection techniques based on polarization transfer, most notably HMQC experiments, are the currently best way to overcome this massive hurdle (Scheme 1); they mandate, however, that a sensitive nucleus (^1^H or ^31^P) is (directly) coupled to the Rh-center, which in turn limits the types of complexes that can be covered.^1−4^

**Scheme 1:**
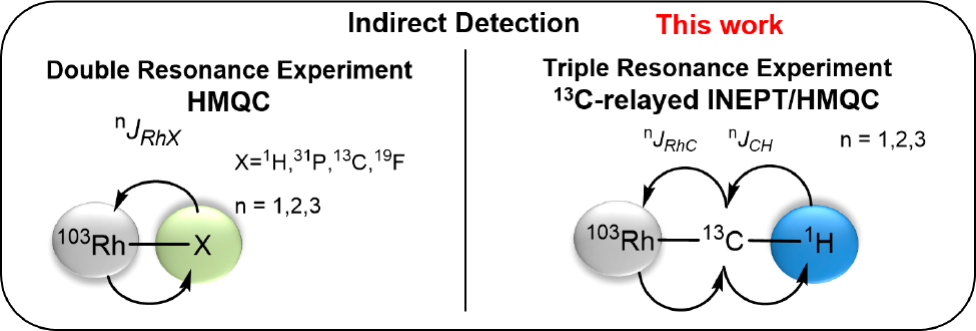
Established and New Methods for the Determination of ^103^Rh Chemical Shifts

Because dirhodium tetracarboxylate complexes lack these requirements, such “paddlewheel” compounds have basically defied scrutiny by ^103^Rh NMR spectroscopy despite their paramount importance in (asymmetric) carbene chemistry and beyond.^9−21^ Not even the ^103^Rh chemical shifts of bare unquenched [Rh_2_(OAc)_4_] (**1**) or [Rh_2_(OTfa)_4_] (**6**) are known,^22−27^ which are the parent members of this series and the starting points for the preparation of innumerous chiral variants by ligand exchange. Outlined below is a convenient NMR experiment that applies to these and other rhodium complexes that were previously beyond reach. A combined experimental and computational approach helps to interpret the now available shift data and in so doing provides insights into the electronic nature of these valuable catalysts.^28−30^

The key to success was the adaptation of a proton-detected triple resonance experiment to the current problem, which draws its high sensitivity from the initial excitation and the final detection of ^1^H (Scheme 1).^31^ After extensive testing, a pulse sequence introduced by Mobley and co-workers was found to give the best results (Figure 1).^32−35^ In the essence, intensive nuclei enhancement by polarization transfer (INEPT) is used for a first magnetization transfer from ^1^H to ^13^C as the relay atom, followed by a heteronuclear multiple quantum correlation (HMQC) transfer from ^13^C to ^103^Rh.^36^

**Figure 1:**
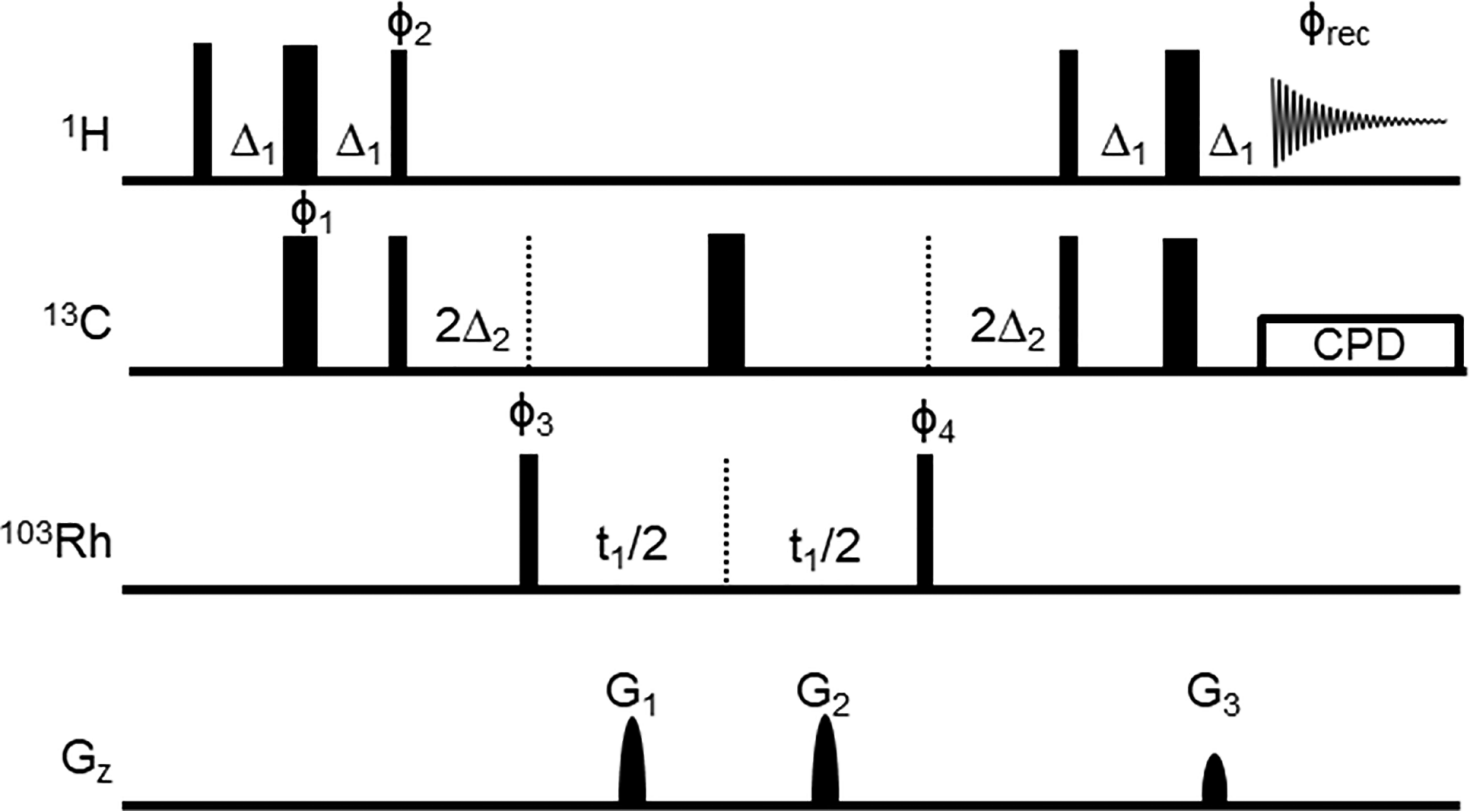
H(C)Rh pulse sequence. The phase cycling scheme is as follows: *x* = pulses without a defined phase; Φ_1_ = *x*, *x*, *x*, *x*, −*x*, −*x*, −*x*, −*x*; Φ_2_ = *y*; Φ_3_ = *x*, −*x*; Φ_4_ = *x*, *x*, −*x*, −*x*; Φ_rec_ = *x*, −*x*, −*x*, *x*. Gradient ratios are as follows: for concentrated samples, G_1_ = 75%, G_2_ = 32%, and G3 = 14.2%; for diluted samples, G_1_ = G_2_ = 80%, G_3_ = 5.1%, *g*_max_ = 51.3 G/cm, Δ_1_ = 1/(4 × *J*_CRh_); Δ_2_ = 1/(4 × *J*_CRh_) for monomeric Rh complexes, Δ_2_ = 1/(12 × *J*_CRh_) for dinuclear Rh(II) paddlewheel complexes.

[Rh(acac)_3_] was chosen for the initial tests because the early literature explicitly stated that the ^103^Rh shift of this compound can only be determined by direct observation.^7^ The triple resonance experiment clearly proves this wrong; it was optimized for magnetization transfer from the protons to the C=O signal (δ_C_ = 188.8 ppm, *J*_C,H_ = 4 Hz) and from ^13^C to ^103^Rh (*J*_C,Rh_ = 1.1 Hz).^38^ The spectrum shown in Figure 2 was recorded in 4 min with a 10 mM solution in CDCl_3_ using nonuniform sampling in the indirect dimension; this result is deemed remarkable in view of a *T*_1_-relaxation time of 39 s (at 9.4 T and 310 K).^5^ The known temperature-dependence of the resonance (1.6 ppm/K) could be easily verified.^1,37^

**Figure 2:**
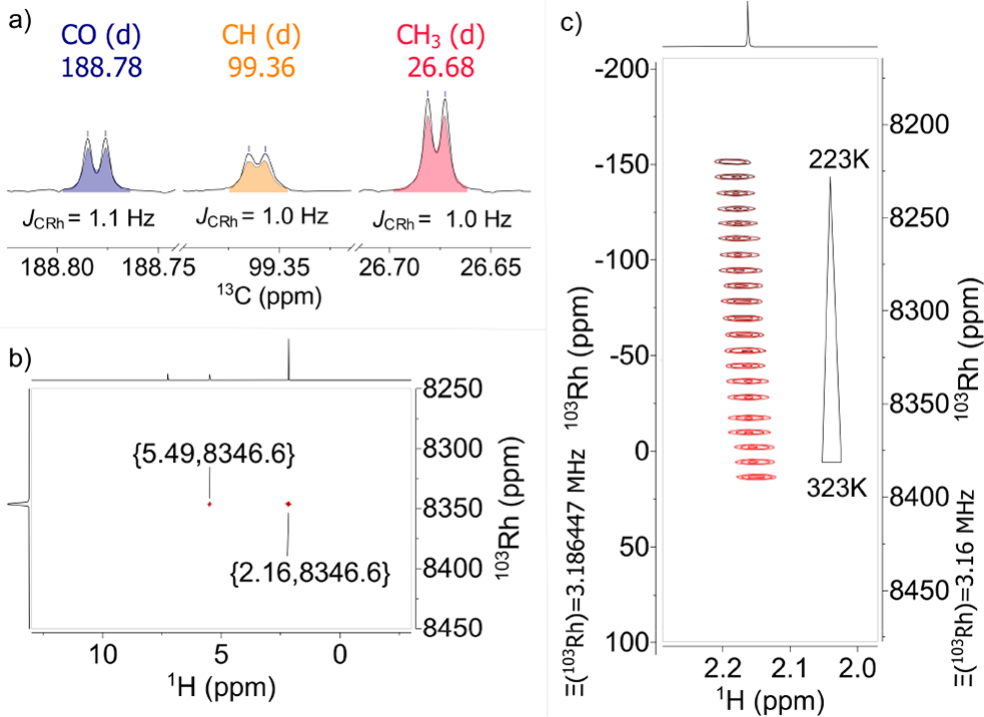
(a) ^13^C{^1^H} NMR signals of Rh(acac)_3_. (b) H(C)Rh spectrum of Rh(acac)_3_ (10 mM). (c) ^103^Rh chemical shift of Rh(acac)_3_ at temperatures between 223 and 323 K; the left axis shows the ^103^Rh chemical shift referenced according to IUPAC recommendations (saturated Rh(acac)_3_ in CDCl_3_ (δ = 0 ppm), Ξ(^103^Rh) = 3.186447%), and the right axis uses the commonly applied reference (Ξ(^103^Rh) = 3.16%)).^37^

The situation in [Rh_2_(OAc)_4_] is slightly more involved because the magnetization will be transferred from one C atom to two chemically equivalent Rh centers. In contrast to a mononuclear complex where the magnetization transfer efficiency of the HMQC step is Δ_2_ = 1/(4 × *J*_CRh_), the optimal delay for an IS_2_ system is theoretically Δ_2_ = 1/(8 × *J*_CRh_).^39^ In our case, however, best results were obtained with Δ_2_ = 1/(12 × *J*_CRh_) (see the Supporting Information). Under these conditions, excellent spectra were recorded with 10–15 mM solutions of isotopically *unlabeled* [Rh_2_(OAc)_4_] in [D_3_]-MeCN with a ≤15 min acquisition time regardless of whether the magnetization transfer pathway I or II was chosen (Figure 3).^40^ Once again, the strong temperature-dependence of the signal (1.33 ppm/K) was easily proven.^41^

**Figure 3:**
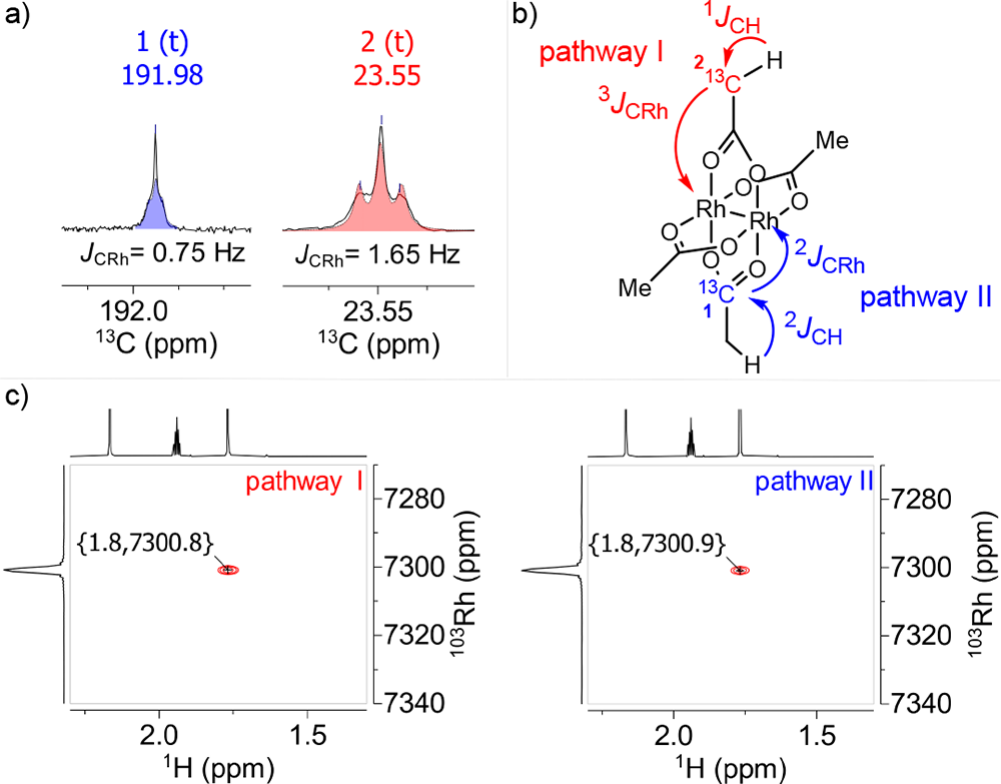
(a) ^13^C{^1^H} NMR signals of [Rh_2_(OAc)_4_] (**1**) in CD_3_CN. (b) Magnetization transfer pathways. (c) H(C)Rh spectra (15 mM) using pathway I or II.

Because acetonitrile acts as kinetically labile ligand to the axial coordination sites on the dimetallic cage of **1**, the effect of different solvents was evaluated (Scheme 2A). The recorded data suggest that [D_8_]-THF and [D_6_]-acetone as supposedly weaker donors than MeCN cause a “deshielding” of the signal, whereas the analogous (catalytically inactive) adduct **1**·PPh_3_ has a notably lower shift.^42−44^

**Scheme 2:**
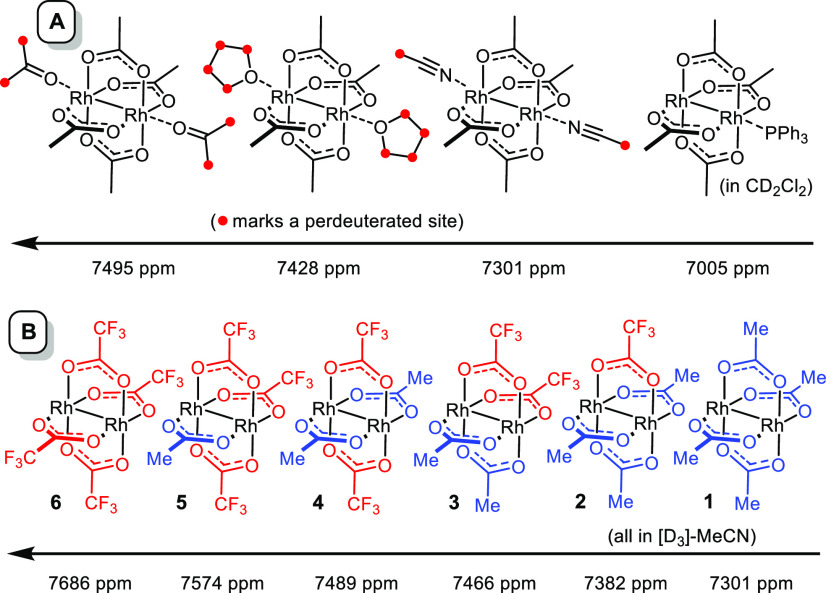
Two Different Test Sets Showing the Influence of the (A) Axial and (B) Equatorial Ligands

Next, the influence of the bridging carboxylate ligands was studied more systematically. To this end, all possible (heteroleptic) dirhodium acetate and trifluoroacetate complexes (**1**–**6**) were prepared and analyzed (Scheme 2B).^45,46^ The recorded data provide a dramatic illustration of the sensitivity of ^103^Rh chemical shifts to changes in the periphery of the nucleus; remote fluorination entails incremental deshielding over a range of no less than 380 ppm. Qualitatively, increased electrophilicity at the rhodium seems to come along with higher δ_Rh_; to scrutinize this aspect, this particular set of complexes was chosen for a detailed computational analysis (see below).

Numerous other dirhodium tetracarboxylate complexes could be analyzed equally well (Figure 4). The recorded δ_Rh_ shifts can be correlated with the p*K*_A_ values of the parent acids,^47^ which capture their donor strengths. Yet, all data fall into a fairly “narrow” shift window (especially if one disregards the highly fluorinated derivatives **5** and **6**), which indicates that the electronic character of the dirhodium core barely changes. One can hence safely conclude that peripheral modifications of the paddlewheels, as practiced in asymmetric catalysis, will hardly change the electronic nature of the catalyst and the derived selectivity-determining transition state.

**Figure 4:**
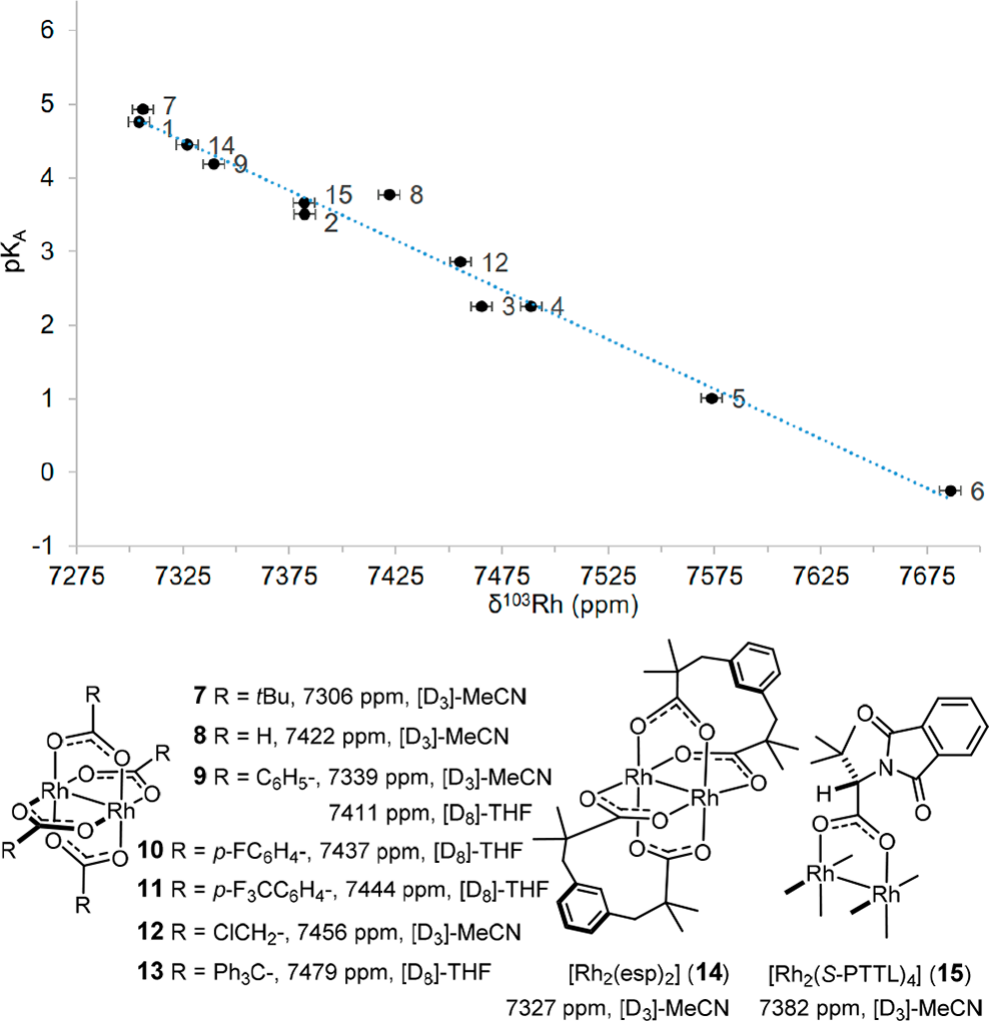
Additional ^103^Rh chemical shift data (298 K, referenced to Ξ(^103^Rh) = 3.16%) and correlation with the p*K*_A_ of the parent carboxylic acids.

The “modest” shift range of the tetracarboxylate complexes is best appreciated by a comparison with dirhodium paddlewheels comprised of one or more N-based ligands (Scheme 3A).^48^ The arguably most instructive example is the heteroleptic complex **16**, since the incorporation of a single −NH group sets the signals of the now chemically different Rh sites >1000 ppm apart. This finding has implications for catalysis, as a chiral relative of **16** was recently shown to be uniquely effective in asymmetric cyclopropanation reactions of α-stannyl(silyl) α-diazoesters.^49^ Indirect evidence suggested that these reactions proceed at the rhodium face carrying the protic −NH group, which according to the shift data is the (much) less electrophilic site; this conclusion clearly mandates further scrutiny. In any case, ^103^Rh NMR makes it unambiguously clear that carboxylate- and carboxamidate-based paddlewheel complexes are very distinct types of catalysts in electronic terms.^48^

**Scheme 3:**
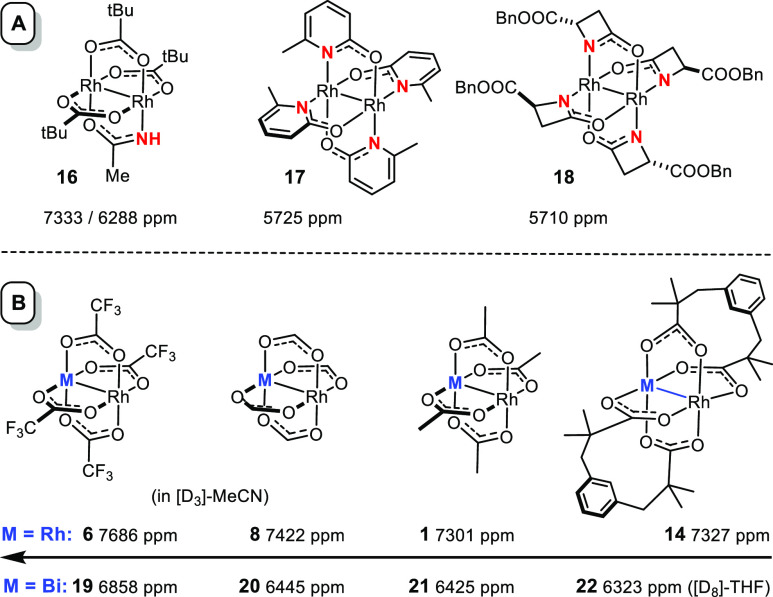
^103^Rh Chemical Shifts of Carboxamidate-Based and Heterobimetallic Complexes

A similar conclusion must be drawn for heterobimetallic [BiRh]-paddlewheel catalysts.^50−53^ They are known to afford much more electrophilic carbene complexes and perform particularly well in asymmetric cyclopropanation reactions.^53−56^ That the 4d orbitals of the Rh(+2) center will sense the incorporation of the sixth-row main group element bismuth as a “metalloligand” is obvious; shift differences of again up to 1000 ppm showcase the magnitude of the effect (Scheme 3B).^46^

Case studies are known in the literature in which the shift of rhodium complexes (or derived reactive intermediates) could be correlated with catalytic performance, but the number is conspicuously small.^2,57−63^ With the technical problems in recording pertinent ^103^Rh NMR spectra come the challenges in interpreting the data, as many different parameters play roles that often prove difficult to disentangle^1−3^ even by computational means.^64−66^ Arguably, however, dirhodium tetracarboxylate complexes in general and the comprehensive subset **1**–**6** in particular are ideally suited for this type of analysis. They all are comprised of the same rigid “lantern” core, and changes in the bite angles and the Rh–O distances are small and secondary interactions within the ligand sphere minute. Hence, the incremental shift changes when going from [Rh_2_(OAc)_4_] (**1**) to [Rh_2_(OTfa)_4_] (**6**) basically reflect electronic rather than geometric changes. A qualitative comparison was therefore deemed legitimate, and a quantitative analysis of the relevant shielding tensors was facilitated because the principal axes coincide in all cases.

Contingent upon the careful optimization of the geometries, the computed shift values reproduce the experimental values remarkably well (Table 1).^67−71^ Differences of no more than 0–36 ppm (on a scale of 12.000 ppm) imply that the chosen level of theory provides an accurate description of complexes of this type. Based on this solid foundation, a more detailed analysis is possible and warranted.

**Table 1:** Comparison between Computed and Experimental ^103^Rh Chemical Shifts^a^^,^^b^

complex	δ_exp_	δ_calc_	*q*	HOMO	LUMO	Δ*E*
**1**	0	0	–0.504	–0.1882	–0.0601	0.1281
**2**	78	80	–0.492	–0.1953	–0.0709	0.1244
**3**	161	152	–0.48	–0.2053	–0.0790	0.1263
**4**	184	169	–0.48	–0.2030	–0.0828	0.1202
**5**	269	275	–0.467	–0.2133	–0.0906	0.1227
**6**	382	346	–0.455	–0.2238	–0.0997	0.1241

a The shift of compound **1** (δ_Rh_ = 7301 ppm) served as a reference.

b Geometry optimization is as follows: B3LYP-D3/def2-TZVPP, CPCM (MeCN)). NMR shifts are as follows: GIAO-ZORA-TPSSh/decontracted SARC-ZORA-TZVPP (Rh), def2-TZVPP (other nuclei), CPCM(MeCN); *q* is the Löwdin atomic charges at Rh; and Δ*E* is the HOMO – LUMO gap.

As shown above, increasing the fluorination of the acetate ligands causes deshielding. The reduced donor ability of the fluorinated ligands renders the complexes increasingly electrophilic; indeed, the computed ^103^Rh chemical shifts can be correlated with the partial charge at Rh (Table 1). Although this picture is intuitive and may provide *rough* guidance for the practitioner, it is—at least—oversimplified. Note that the computed energy of the LUMO decreases when going from **1** to **6**, as expected, but the energy of the HOMO drops to a similar extent such that Δ*E* remains essentially constant. The HOMO – LUMO gap alone does obviously not explain the observed results.

NMR shifts are neither primarily determined by the charge at the metal nor solely by the frontier orbitals. Rather, a chemical shift is an anisotropic property (even though solution NMR spectroscopy provides only the isotropic shift (δ_iso_ = (δ_11_ + δ_22_ + δ_33_)/3). Computational methods allow the individual components of the shielding tensor σ (δ_*ii*_ = σ_iso,ref_ – σ_*ii*_) to be deconvoluted; it is the paramagnetic term (σ = σ_dia_ + σ_para_) that largely determines the shift of a transition metal nucleus.^72^

The paramagnetic contributions arise from magnetically induced admixture of electronically excited states into the electronic ground state by the angular momentum operator (*L̂*_*i*_) as described by the Ramsey equation (see the Supporting Information).^73^ Deshielding in the direction σ_ii,para_ depends on which orbitals ϕ_occ_ and ϕ_vir_ can be coupled via *L̂*_*i*_ and on their relative energies: the smaller the energy gap, the larger the effect.

For complexes **1**–**6**, the dominant contributions to σ_*ii*,para_ stem from interactions of the key frontier orbitals (Figure 5). As expected, all MOs are delocalized over both Rh centers. Due to symmetry, the only nonzero contributions to the σ_*zz*,para_ component stem from the coupling of the virtual orbitals with the HOMO – 2 (d_*xy*_), which is delocalized over the ligands on both axes. As a consequence, σ_*zz*,para_ is always affected by fluorination, irrespective of the position of the halogen atoms. The largest contributions to σ_*yy*,para_ originate from the coupling of the virtual orbitals with the HOMO – 1 (d_*xz*_) orbital, which is mostly delocalized over the ligands on the *x*-axis. Similarly, the largest contributions to σ_*xx*,para_ originate from the coupling of the virtual orbitals with the HOMO (d_*yz*_) orbital, which is mostly delocalized over the ligands on the *y*-axis. Hence, σ_*yy*,para_ and σ_*xx*,para_ mainly respond to fluorination on the *perpendicularx*- and *y*-axes, respectively. Figure 6 illustrates this effect, as only δ_*zz*,para_ shows a near linear correlation with the total chemical shift and increases with the increasing fluorination; in contrast, δ_*yy*,para_ is roughly constant for **1**, **2**, and **4**, all of which are nonfluorinated along the *x*-axis, while δ_*xx*,para_ is basically constant for complexes **4**, **5**, and **6**, which are fully fluorinated along the *y*-axis. Analysis of a given shielding tensor hence provides information about the donor strength of the ligand at the perpendicular position of the lantern core.

**Figure 5:**
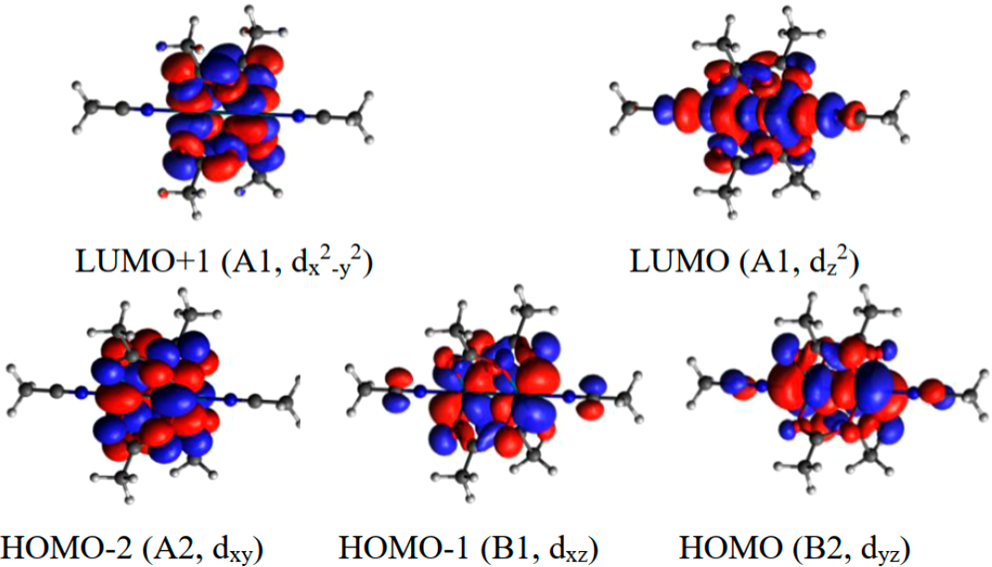
Relevant molecular orbitals of **1** (the associated irreducible representation in the *C*_2v_ molecular point group and the Rh d orbital associated with each MO are shown in brackets).

**Figure 6:**
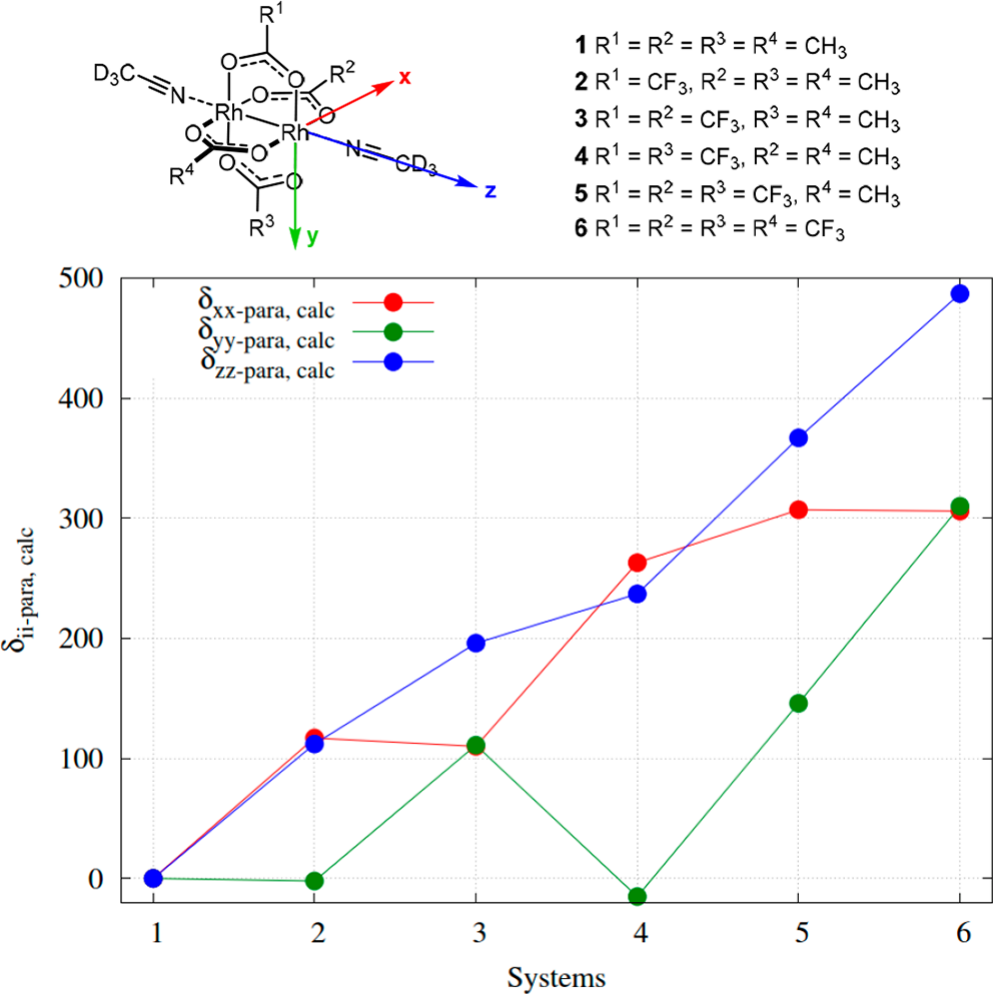
(Top) Principal axis of the shielding tensor of Rh. (Bottom) The paramagnetic contribution to each principal component of the shielding tensor for complexes **1**–**6**.

The subtleties within this series notwithstanding, one will hardly go wrong in assuming that the ≈880 ppm shift difference between [Rh_*2*_(OAc)_*4*_] (**1**) and [BiRh(OAc)_4_] (**21**) bears witness of a drastically different electronic character (Scheme 3). An accurate assessment requires the same kind of analysis as outlined above for the (partly) fluorinated complexes; qualitatively, however, the massive upfield shift suggests that the Rh center, which is the catalytically relevant site of **21**,^53,56^ is (much) less electrophilic. The situation in the heteroleptic species **16** is similarly intuitive; because of its relevance, however, a more detailed profiling is subject to ongoing studies. Likewise, we are using the H(C)Rh triple resonance experiment for investigations into other previously uncompliant rhodium complexes, including (highly) reactive intermediates.^74−77^ Pertinent results will be reported in due course.

## Supporting Information Available

Experimental Section containing supporting NMR data and computational data (PDF)
