# Supplementary material for: Triple Resonance Experiments for the Rapid Detection of 103Rh NMR Shifts: A Combined Experimental and Theoretical Study into Dirhodium and Bismuth–Rhodium Paddlewheel Complexes
Source: J Am Chem Soc. 2021 Aug 5;143(32):12473–9. doi: 10.1021/jacs.1c06414 (PMC8377716; doi:10.1021/jacs.1c06414)
Supplement: Supplementary file 1 — ja1c06414_si_001.pdf [file ja1c06414_si_001.pdf]

# SUPPORTING INFORMATION

# Triple Resonance Experiments for Rapid Detection of $^{103}$Rh NMR Shifts. A Combined Experimental and Theoretical Study into Dirhodium- and Bismuth-Rhodium Paddlewheel Complexes

Fabio P. Caló, Giovanni Bistoni, Alexander A. Auer, Markus Leutzsch,* and Alois Fürstner*

*Max-Planck-Institut für Kohlenforschung, 45470 Mülheim/Ruhr, Germany*

fuerstner@kofo.mpg.de

leutzsch@kofo.mpg.de

## Table of Contents

1. General ........ S2
2. Dirhodium Complexes ........ S3
3. Bismuth-Rhodium Complexes ........ S6
4. Supporting NMR Spectroscopic Data ........ S8
5. Computational Study ........ S11
6. NMR Spectra ........ S34
7. References ........ S83

# 1. General

Unless stated otherwise, all reactions were carried out under argon atmosphere in flame dried Schlenk glassware, ensuring inert conditions. The solvents were purified by distillation over the indicated drying agents under argon: THF, $Et_2O$ (Mg/anthracene), pentane, toluene (Na/K), $CH_2Cl_2$, chlorobenzene ($CaH_2$); MeCN was dried by an absorption solvent purification system based on molecular sieves. Flash chromatography: Merck Geduran silica gel 60 (40 – 63 µm).

NMR spectra were recorded on Bruker AVIII 400, AVIII 500 or AVNeo 600 spectrometers in the solvents indicated at 298 K unless indicated otherwise; chemical shifts (δ) are given in ppm relative to TMS, coupling constants (*J*) in Hz. The solvent signals were used as internal references and the chemical shifts converted to the TMS scale ($CDCl_3$: $\delta_C$ = 77.16 ppm; residual $CHCl_3$: $\delta_H$ = 7.26 ppm; $CD_2Cl_2$: $\delta_C$ = 54.0 ppm; residual $CHDCl_2$: $\delta_H$ = 5.32 ppm; $C_6D_6$: $\delta_C$ = 128.1 ppm; residual $C_6HD_5$: $\delta_H$ = 7.16 ppm; $CD_3CN$: $\delta_C$ = 1.32, 118.3 ppm; residual $CD_2HCN$: $\delta_H$ = 1.94 ppm).[1] Signal assignments were established using $^{1}H$-$^{13}C$-*edited*-HSQC and $^{1}H$-$^{13}C$-HMBC experiments. $^{19}F$ and $^{103}Rh$ NMR shifts were referenced indirectly to the $^{1}H$ NMR frequency of the sample with the '*xiref*'-macro in Bruker TOPSPIN 3.6.2 (for $^{103}Rh$)/TOPSPIN 4.0.6.[2] $^{19}F$ shifts are reported relative to δ($CFCl_3$) = 0 ppm ($\Xi(^{19}F)$= 94.094011 %). $^{103}Rh$ NMR shifts are referenced to $\Xi(^{103}Rh)$ = 3.16% unless indicated otherwise.

1D $^{103}Rh$ NMR (pulse sequence: zg) and 2D $^{1}H$-$^{103}Rh$-HMBC spectra (pulse sequence: hmbcgpqfnd) were acquired on a AVIII 500 MHz NMR spectrometer equipped with a 5 mm BBFO probe ($^{1}H$, $^{19}F$ & $^{31}P$-$^{109}Ag$) with z-gradient which could be tuned on the X-channel to $^{103}Rh$ beyond the specifications. The typical π/2 $^{103}Rh$ pulse length was 40 µs when a pulse power of 180 W was used.

For the 1D $^{103}Rh$ NMR measurements, saturated samples in 5 mm NMR tubes were used. After excitation with a 70° pulse, 32k complex data points were acquired. The total acquisition time per scan was approximately 4s. To obtain an acceptable SNR 15000 scans were averaged (17 h measurement time).

The 1D $^{13}C\{^{1}H, ^{103}Rh\}$ (pulse sequence: zgigf2igf3), 2D $^{13}C\{^{1}H\}$-$^{103}Rh$-HMBC and 2D H(C)Rh spectra were acquired on a AVIII 500 MHz NMR spectrometer equipped with a 5 mm TBI probe ($^{1}H$, $^{31}P$-$^{109}Ag$, $^{13}C$) with z-gradient coil which could be tuned on the broadband X-channel to $^{103}Rh$ beyond the specifications. The typical π/2 $^{103}Rh$ pulse length was 90 µs when a pulse power of 180 W was used.

Spectra with $^{103}Rh$ in the indirect dimension (X-$^{103}Rh$-HMBC, H(C)Rh) were generally sparsely sampled (10% - 15% NUS), which reduced the measurement time significantly.

IR: Alpha Platinum ATR (Bruker), wavenumbers ($\tilde{\nu}$) in $cm^{-1}$ Most medium and weak resonances were omitted.

MS (EI): Finnigan MAT 8200 (70 eV), ESI-MS: ESQ 3000 (Bruker) or Thermo Scientific LTQ-FT or Thermo Scientific Exactive Spectrometer. HRMS: Bruker APEX III FT-MS (7 T magnet), MAT 95 (Finnigan), Thermo Scientific LTQ-FT or Thermo Scientific Exactive Spectrometer. GC-MS spectra were measured on a Shimadzu GCMS-QP2010 Ultra instrument.

Unless stated othewise, all commercially available compounds including the complexes Rh(acac)$_3$, $Rh_2(OAc)_4$ (**1**), $Rh_2(TPA)_4$ (**13**), $Rh_2(esp)_2$ (**14**) and $Rh_2$(*S*-PTTL)$_4$ (**15**) (abcr, Acros, Aldrich, Alfa Aesar, Fluoro Chem, Strem, TCI) were used as received.

$Rh_2(OTfa)_4$ (**6**),[3] $Rh_2(MHP)_4$ (**17**)[4] and $BiRh(OTfa)_4$ (**19**)[5] were prepared according to literature procedures.

## 2. Dirhodium Complexes

The heteroleptic dirhodium acetate/trifluoroacetate complexes (**2**, **3** and **5**) were prepared according to a modified literature protocol, originally reported for the synthesis of **3**.[6] A flame dried two-neck flask with attached reflux condenser was charged with $[Rh_2(OAc)_4]2H_2O$ (200 mg, 0.42 mmol) under Ar atmosphere. Trifluoroacetic acid (20 mL, 261.17 mmol) was added and the resulting suspension was stirred at the indicated temperature. After the allocated reaction time had passed, the mixture was allowed to cool to ambient temperature and all volatile materials were removed under reduced pressure. The resulting green residue contained a mixture of heteroleptic complexes which were separated by flash chromatography (silica, gradient of $CH_3CN$/toluene: 2 : 98 – 1 : 1).

Variation of temperature and reaction time strongly influenced the composition of the obtained mixture. When the reaction mixture was stirred for **150 min at ambient temperature**, the following composition was obtained: $Rh_2(OAc)_3(OTfa)$: 2mg, 0.01 mmol, 1%; *cis*-$Rh_2(OAc)_2(OTfa)_2$: 143 mg, 0.26 mmol, 62%; $Rh_2(OAc)(OTfa)_3$: 67 mg, 0.11 mmol, 27%; $Rh_2(OTfa)_4$: 10 mg, 0.02 mmol, 4%.

After **15 min at 72°C**, the composition was as follows (starting with 150 mg $[Rh_2(OAc)_4]2H_2O$ (0.36 mmol)): *cis*-$Rh_2(OAc)_2(OTfa)_2$: 36 mg, 0.07 mmol 18%; $Rh_2(OAc)(OTfa)_3$: 69 mg, 0.16 mmol, 46%; $Rh_2(OTfa)_4$: 78 mg, 0.12 mmol 33%.

**Dirhodium(II)tris(acetate)trifluoroacetate [$Rh_2(OAc)_3(OTfa)$] (2):** Green solid material; 1H NMR (500 MHz, $CD_3CN$) δ 1.82 (s, 6H), 1.8 (s, 3H) ppm; $^{13}$C-NMR (151 MHz, $CD_3CN$): δ = 194.0, 193.2, 174.1 (q, $J_{C\text{-}F}$ = 38.4 Hz), 23.7, 23.6 ppm ($CF_3$ not detected); $^{19}$F-NMR (565 MHz, $CDCl_3$): δ = −70.5 ppm; HRMS (ESI+): m/z calcd. for $C_8H_9F_3O_8Rh_2Na$ [M+Na]: 518.8252; found: 518.8255.

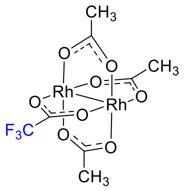


***cis*-Dirhodium(II)bis(acetate)bis(trifluoroacetate) [*cis*-$Rh_2(OAc)_2(OTfa)_2$] (3):** Analytical data is in agreement with the reported data.[6] Blue-green solid material; $^1$H-NMR (600 MHz, $CD_3CN$): δ = 1.85 ppm; $^{13}$C{$^1$H}-NMR (151 MHz, $CD_3CN$): δ = 195.3, 175.1 (q, $J_{C\text{-}F}$ = 38.9 Hz), 111.8 (q, $J_{C\text{-}F}$ = 285.0 Hz), 23.8 (d, $J_{C\text{-}Rh}$ = 1.8 Hz) ppm; $^{19}$F-NMR (565 MHz, $CD_3CN$): δ = −70.5 ppm; HRMS (ESI+): m/z calcd. for $C_8H_6F_6O_8Rh_2Na$ [M+Na]: 572.7969; found: 572.7960.

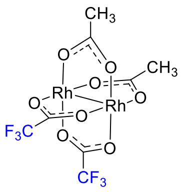


***trans*-Dirhodium(II) bis(acetate)bis(trifluoroacetate) [*trans*-$Rh_2(OAc)_2(OTfa)_2$] (4):** Prepared according to a reported literature protocol.[6] $^1$H-NMR (600 MHz, $CD_3CN$): δ = 1.86 ppm; $^{13}$C{$^1$H}-NMR (151 MHz, $CD_3CN$): δ = 194.5, 175.7 (q, $J_{C\text{-}F}$ = 39.1 Hz), 111.3 (q, $J_{C\text{-}F}$ = 284.4 Hz), 23.9 (d, $^3J_{C\text{-}Rh}$ = 1.4 Hz) ppm; $^{19}$F-NMR (565 MHz, $CD_3CN$): δ = −75.7 ppm; HRMS (ESI-): m/z calcd. for $C_8H_7F_6O_9Rh_2$ [M+OH]: 566.8110; found: 566.8114.

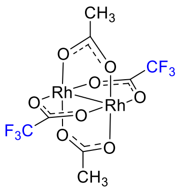

**Dirhodium(II)acetatetris(trifluoroacetate) [$Rh_2(OAc)(OTfa)_3$] (5):** Blue solid material; $^1$H-NMR (500 MHz, $CD_3CN$): δ = 1.96 (s, $CH_3CN$), 1.90 (s, 3H) ppm; $^{13}C\{^1H\}$-NMR (151 MHz, $CD_3CN$): δ = 196.7, 176.8 (q, $^2J_{C-F}$ = 39.6 Hz), 176.0 (q, $^2J_{C-F}$ = 39.4 Hz), 111.4 (m, 2$CF_3$) 24.0 (d, $^3J_{C-Rh}$ = 1.6 Hz) ppm; $^{19}$F-NMR (565 MHz, $CD_3CN$): δ = -70.4, -70.5 ppm; HRMS (DP-EI): m/z calcd. for $C_8H_3F_9O_8Rh_2$ [M+]: 603.77888; found: 603.7798.

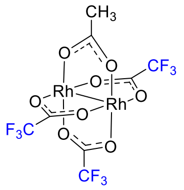


**Dirhodium(II) (acetamidate)tris(pivalate) [$Rh_2(ACAM)(OPiv)_3$] (16):** A flame dried two-neck flask with an attached reflux condenser was charged under Ar with dirhodium(II) tetrakis(acetamidate) dihydrat [$Rh_2(ACAM)$]·$2H_2O$ (80 mg, 0.17 mmol)[7] and pivalic acid (53 mg, 0.51 mmol). Chlorobenzene (20 mL) was added and the purple suspension was first degassed by bubbling Ar through it for 15 min before it was stirred at 132°C for 18 h, during which the color changed from purple to green. The mixture was allowed to cool to ambient temperature and the volatile materials were removed under reduced pressure. The resulting green-blue residue was purified by flash chromatography (silica, acetonitrile:toluene 1:9) to give the title compound as a green solid material (15 mg, 16%). $^1$H-NMR (600 MHz, $CD_3CN$): δ = 4.86 (s, 1H), 1.78 (s, 3H), 0.93 (s, 9H), 0.89 (s, 18H) ppm; $^{13}C\{^1H\}$-NMR (151 MHz, $CD_3CN$): δ = 198.5, 196.8, 187.0, 40.7, 40.5, 28.3, 28.0, 24.2 ppm; $^{15}$N-NMR ($^1$H-$^{15}$N-HMBC, $CD_3CN$ ): δ = 296.0 ppm. HRMS (ESI-): m/z calcd. for $C_{17}H_{30}O_7NRh$ [M-H]: 566.0138; found: 566.0142.

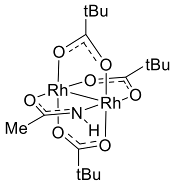


**Dirhodium(II) tetrakis(pivalate) [$Rh_2(OPiv)_4$] (7).** Isolated as a second fraction from the crude product; green solid material (21 mg, 20%); Analytical data matches the literature.[8] $^1$H-NMR (400 MHz, $CD_3CN$): δ = 0.91 (s, 36H) ppm; HRMS (ESI+): m/z calcd. for $C_{20}H_{36}O_8Rh_2Na$ [M+Na]: 633.0407; found: 633.0407.

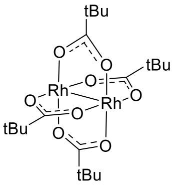


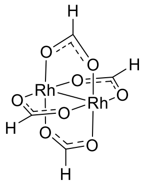


**Dirhodium tetraformiat [$Rh_2(HCO_2)_4$] (8):** Prepared according to a literature procedure.[9] $^1$H-NMR (400 MHz, $CD_3CN$): δ = 6.89 (t, $^3J_{H-Rh}$ = 4.7 Hz, 4H) ppm; $^{13}C\{^1H\}$-NMR (101 MHz, $CD_3CN$): δ = 183.2 ppm.

**Representative Procedure for Ligand Exchange on [$Rh_2(OTfa)_4$] or [$BiRh(OTfa)_4$]. Dirhodium(II) tetra(acetate-2-$^{13}$C) ($Rh_2(OAc\text{-}2\text{-}^{13}C)_4$):** A flame dried Schlenk flask was charged under Ar with dirhodium(II) tetrakis(trifluoroacetate) [$Rh_2(OTfa)_4$] (35 mg, 0.05 mmol) and acetonitrile (0.1 mL). Acetic acid-2-$^{13}$C (44 μL, 0.32 mmol) and triethylamine (19 μL, 0.33 mmol) were added to the red suspension, which was stirred at 60°C for 30 min. The mixture was allowed to cool to ambient temperature before all volatile materials were removed under reduced pressure. The resulting purple residue was purified by flash chromatography (silica, acetonitrile) to give the title compound with axially coordinated acetonitrile as a purple solid compound (24 mg, 97%). $^1$H-NMR (400 MHz, $CD_3CN$): δ = 1.77 (d, $^1J_{CH}$ = 128.7 Hz, 12H) ppm; $^{13}C\{^1H\}$-NMR (101 MHz, $CD_3CN$): δ = 191.7, 23.5 (t, $^3J_{C-Rh}$ = 1.8 Hz) ppm; HRMS (ESI+): m/z calcd. for $^{13}C_4C_4H_{12}O_8Rh_2Na$ [M+Na]: 468.8669; found: 468.8674.

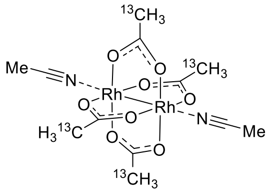

**Dirhodium(II) tetra(acetate-1-$^{13}$C) [$Rh_2$(OAc-2-$^{13}$C)$_4$].** Prepared analogously from [$Rh_2(OTfa)_4$] (35 mg, 0.05 mmol), acetic acid-1-$^{13}$C (44 μL, 0.32 mmol) and triethylamine (19 μL, 0.33 mmol) as a purple solid material (21 mg, 82%). $^1$H-NMR (400 MHz, $CD_3CN$): δ = 1.77 (d, $^2J_{C\text{-}H}$ = 6.4 Hz, 1H) ppm; $^{13}C\{^1H\}$-NMR (101 MHz, $CD_3CN$): δ = 191.9, 23.5 (d, $^2J_{C\text{-}C}$ = 56.7 Hz) ppm; HRMS (ESI+): m/z calcd. for $^{13}C_4C_4H_{12}O_8Rh_2Na$ [M+Na]: 468.8669; found: 468.8671.

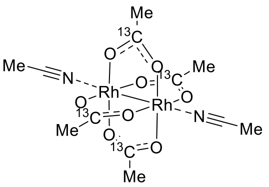


**Dirhodium(II) tetra(benzoate) [$Rh_2(OBz)_4$] (9):** Prepared analogously from ($Rh_2(OTfa)_4$) (30 mg, 0.05 mmol), benzoic acid (35 mg, 0.29 mmol) and triethylamine (40 μL, 0.29 mmol) as a green solid material (33 mg, 94%). $^1$H-NMR (400 MHz, $CD_3CN$): δ = 7.88 – 7.78 (m, 8H), 7.43 (m, 4H), 7.35 – 7.26 (m, 8H) ppm; $^{13}C\{^1H\}$-NMR (101 MHz, $CD_3CN$): δ = 186.2, 133.1, 132.3, 129.2, 129.0 ppm; HRMS (ESI+): m/z calcd. for $C_{28}H_{20}O_8Rh_2Na$ [M+Na]: 712.9161; found: 712.9157.

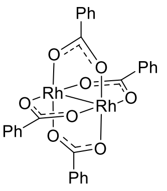


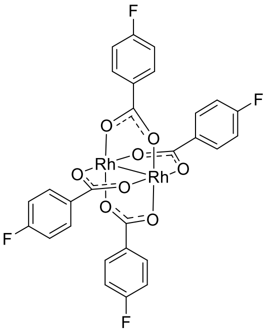


**Dirhodium(II) tetrakis(*p*-fluorobenzoate) [$Rh_2$(*p*-F-$C_6H_4COO)_4$] (10):** Prepared analogously from [$Rh_2(OTfa)_4$] (30 mg, 0.05 mmol), *p*-fluorobenzoic acid (39 mg, 0.28 mmol) and triethylamine (40 μL, 0.29 mmol) as a blue-green solid material (30 mg, 86%). Analytical data matches the literature.[10] $^1$H-NMR (400 MHz, THF-$d_8$): δ = 7.95 – 7.83 (m, 8H), 6.96 (t, *J* = 8.7 Hz, 8H) ppm; HRMS (ESI+): m/z calcd. for $C_{28}H_{16}O_8F_4Rh_2Na$ [M+Na]: 784.8784; found: 784.8772.

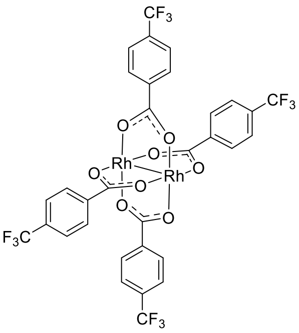


**Dirhodium(II) tetrakis(*p*-trifluoromethylbenzoate) [$Rh_2$(*p*-$F_3C$-$C_6H_4COO)_4$] (11):** Prepared analogously from [$Rh_2(OTfa)_4$] (30 mg, 0.05 mmol), *p*-trifluoromethylbenzoic acid (39 mg, 0.28 mmol) and triethylamine (40 μL, 0.29 mmol) as a green solid material (35 mg, 80%). Analytical data matches the literature.[10] $^1$H-NMR (400 MHz, THF-$d_8$): δ = 8.02 (d, *J* = 8.1 Hz, 8H), 7.59 (d, *J* = 8.2 Hz, 8H) ppm; HRMS (APPI+): m/z calcd. for $C_{32}H_{17}O_8F_{12}Rh_2$ [M+H]: 962.8836; found: 962.8834.

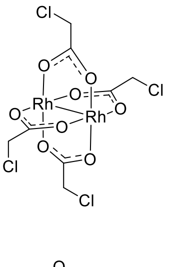


**Dirhodium(II) tetrakis(chloroacetate) (12):** Prepared analogously from [$Rh_2(OTfa)_4$] (30 mg, 0.05 mmol), chloroacetic acid (16 mg, 0.28 mmol) and triethylamine (40 μL, 0.29 mmol) as a green solid material (35 mg, 80%). $^1$H-NMR (600 MHz, $CD_3CN$): δ = 3.92 (s, 8H) ppm; $^{13}C\{^1H\}$-NMR (151 MHz, $CD_3CN$): δ = 187.8, 42.4 ppm; HRMS (ESI+): m/z calcd. for $C_8H_8O_8Cl_4Rh_2Na$ [M+Na]: 600.6976; found: 600.6973.

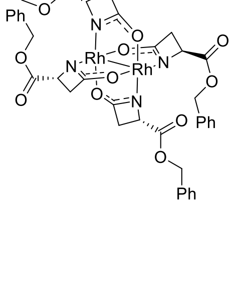


**[$Rh_2$(*S*-BNAZ)$_4$] (18):** Prepared according to a literature procedure.[11] The crude material was purified by HPLC (150 mm x 30 mm, YMC-Triart C18, 5 μm, 4.6 mm i.D., 10 % methanol in water, 42.5 mL·min$^{-1}$, 7 min, UV 220 nm; retention time of product: 3.5 min).

$^1$H-NMR (400 MHz, $CD_3CN$): δ = 7.48 – 7.27 (m, 20H), 5.22 – 5.05 (m, 8H), 3.79 – 3.72 (m, 4H), 3.23 (dd, *J* = 13.4, 4.6 Hz, 2H), 3.03 (m, 4H), 2.85 (dd, *J* = 13.4, 4.6 Hz, 2H) ppm; $^{13}C\{^1H\}$-NMR (101 MHz, $CD_3CN$): δ = 189.6, 189.3, 174.7, 173.9,

137.6, 137.2, 129.7, 129.4, 129.3, 129.2, 129.1, 128.9, 67.5, 67.0, 53.4, 52.9, 44.0, 43.1 ppm. HRMS (ESI+): m/z calcd. for $C_{44}H_{40}O_{12}Rh_2Na$ [M+Na]: 1045.0645; found: 1045.0649.

# 3. Bismuth-Rhodium Complexes

**Bismuth-rhodium tetra(acetate-2-$^{13}$C) (20-2-$^{13}$C):** Prepared analogously from [BiRh(OTfa)$_4$] (25 mg, 0.03 mmol), acetic acid-2-$^{13}$C (30 μL, 0.22 mmol) and triethylamine (13 μL, 0.22 mmol) as a yellow solid material, which precipitated during the reaction and was isolated by filtering the reaction mixture and washing the yellow solid compound with cold acetonitrile (11 mg, 69%). NMR samples were prepared by heating a suspension of the title compound in $CD_3CN$ to reflux for 1 min, until the compound had partially dissolved. $^1$H-NMR (400 MHz, $CD_3CN$): δ = 1.93 (d, $^1J_{C-H}$ = 129.9 Hz, 12H) ppm; $^{13}$C{$^1$H}-NMR (101 MHz, $CD_3CN$): δ =22.4 (d, $^3J_{C-Rh}$= 0.9 Hz) ppm (Carboxylate-C not detected); HRMS (ESI+): m/z calcd. for $^{13}C_4C_4H_{12}O_8BiRhNa$ [M+Na]: 574.9417; found: 574.9420.

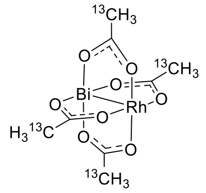

**Bismuth-rhodium tetra(acetate) [BiRh(OAc)$_4$] (20):** Prepared analogously from [BiRh(OTfa)$_4$] (50 mg, 0.07 mmol), acetic acid (55 μL, 0.39 mmol) and triethylamine (23 μL, 0.40 mmol) as a yellow solid material, which precipitated during the reaction and was isolated by filtering the mixture and washing the yellow solid material with cold acetonitrile (28mg, 78%). NMR samples were prepared by heating a suspension of the title compound in $CD_3CN$ to reflux for 1 min, until the compound had partially dissolved. Analytical data matches the literature.[12] $^1$H-NMR (400 MHz, $CD_3CN$): δ = 1.91 (s, 12H) ppm; $^{13}$C{$^1$H}-NMR (151 MHz, $CD_3CN$): δ = 187.2, 22.4 ppm; HRMS (ESI+): m/z calcd. for $C_8H_{12}O_8BiRhNa$ [M+Na]: 570.928; found: 570.9282.

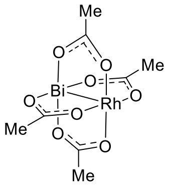

**Bismuth-rhodium tetraformiat [BiRh(HCO$_2$)$_4$] (21):** Prepared analogously from [BiRh(OTfa)$_4$] (15 mg, 0.02 mmol), formic acid (5 μL, 0.12 mmol) and triethylamine (17 μL, 0.12 mmol) and purified by flash chromatography (silica, $CH_3CN$ : toluene 1:9) to give the title compound as a yellow solid (6 mg, 66%). $^1$H-NMR (400 MHz, $CD_3CN$): δ = 8.44 (d, $^3J$ = 3.0 Hz, 4H) ppm; $^{13}$C{$^1$H}-NMR (101 MHz, $CD_3CN$): δ = 177.4 ppm; HRMS (ESI+): m/z calcd. for $C_4H_5O_8BiRh$ [M+H]: 492.8838; found: 492.8836.

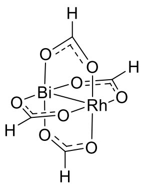

**[BiRh(esp)$_2$] (22):** Prepared analogously from [BiRh(OTfa)$_4$] (8 mg, 0.01 mmol), α,α,α',α'-tetramethyl-1,3-benzodipropionic acid (9 mg, 0.06 mmol) and triethylamine (9 μL, 0.6 mmol); purification by flash chromatography (silica, $CH_3CN$) gave the title compound as a yellow solid material (9 mg, 93%). Analytical data matched the literature.[12] $^1$H-NMR (400 MHz, $CD_3CN$): δ = 7.08 (t, $J$ = 7.5 Hz, 2H), 6.89 (dd, $J$ = 7.6, 1.8 Hz, 4H), 6.72 (m, 2H), 2.79 (d, $^2J_{H-H}$ = 12.5 Hz, 4H), 2.55 (d, $^2J_{H-H}$ = 12.6 Hz, 4H), 1.07 (s, 12H), 0.98 (s, 12H) ppm; HRMS (ESI+): m/z calcd. for $C_{32}H_{40}O_8BiRhNa$ [M+Na]: 887.1472; found: 887.1472.

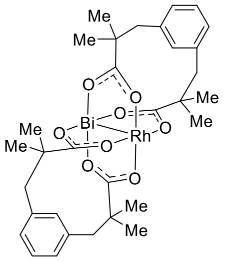

**Table S1**. Overview of $^{103}$Rh NMR shifts of different rhodium paddlewheel complexes. All shifts were measured at 298K and are referenced to Ξ($^{103}$Rh) = 3.16 MHz.

| # | Compound | pKa ligand | solvent | δ $^{103}$Rh [ppm] | NMR-experiment |
|---|---|---|---|---|---|
| **1** | $Rh_2(OAc)_4$ | 4.76 [13] | $CD_3CN$ | 7301 | H(C)Rh |
| | | | [$D_8$]-THF | 7427 | H(C)Rh |
| | | | [$D_6$]-acetone | 7495 | H(C)Rh |
| | | | $CD_2Cl_2$ / $PPh_3$ | 7005 | H(C)Rh |
| **2** | $Rh_2(OAc)_3(OTfa)$ | 3.51[c] | $CD_3CN$ | 7382 | H(C)Rh |
| **3** | *cis*-$Rh_2(OAc)_2($ OTfa $)_2$ | 2.26[c] | $CD_3CN$ | 7466 | H(C)Rh |
| **4** | *trans*-$Rh_2(OAc)_2($ OTfa $)_2$ | 2.26 [c] | $CD_3CN$ | 7489 | H(C)Rh |
| **5** | $Rh_2(OAc)($ OTfa $)_3$ | 1.00[c] | $CD_3CN$ | 7574 | H(C)Rh |
| **6** | $Rh_2($ OTfa $)_4$ | -0.25[b] | $CD_3CN$ | 7686 | 1D-$^{103}$Rh |
| **7** | $Rh_2(OPiv)_4$ | 5.05[13] | $CD_3CN$ | 7306 | H(C)Rh |
| **8** | $Rh_2(HCO_2)_4$ | 3.77[b] | $CD_3CN$ | 7422 | $^{1}$H-$^{103}$Rh-HMBC |
| **9** | $Rh_2(OBz)_4$ | 4.20[b] | $CD_3CN$ | 7339 | H(C)Rh |
| | | | [$D_8$]-THF | 7411 | H(C)Rh |
| **10** | $Rh_2$(*p*-$FC_6H_4COO)_4$ | 4.14[d] | [$D_8$]-THF | 7437 | H(C)Rh |
| **11** | $Rh_2($ *p*-$F_3CC_6H_4COO)_4$ | 3.69[d] | [$D_8$]-THF | 7444 | H(C)Rh |
| **12** | $Rh_2(ClCH_2CO_2)_4$ | 2.86[13] | $CD_3CN$ | 7456 | H(C)Rh |
| **13** | $Rh_2(TPA)_4$ | 4.03[d] | [$D_8$]-THF | 7479 | H(C)Rh |
| **14** | $Rh_2(esp)_2$ | 4.45[d] | $CD_3CN$ | 7327 | H(C)Rh |
| **15** | $Rh_2(PTTL)_4$ | 3.66 | $CD_3CN$ | 7382 | H(C)Rh |
| **16** | $Rh_2(ACAM)(OPiv)_4$ | | $CD_3CN$ | 7333/6288 | H(C)Rh |
| **17** | $Rh_2(mhp)_4$ | 12.12[d] | $CD_2Cl_2/CD_3CN$ (50/50 *v/v*) | 5725 | H(C)Rh |
| | | | | 5726 | $^{1}$H-$^{103}$Rh-HMBC |
| **18** | $Rh_2(BNAZ)_4$ | 13.75[d] | $CD_3CN$ | 5709 | $^{1}$H-$^{103}$Rh-HMBC |
| **19** | $BiRh(TFA)_4$ | -0.25[b] | $CD_3CN$ | 6858 | 1D-$^{103}$Rh |
| **20** | $BiRh(OAc)_4$ | 4.76 [13] | $CD_3CN$ | 6423 | H(C)Rh |
| **21** | $BiRh(HCO_2)_4$ | 3.77[b] | $CD_3CN$ | 6445 | HMBC |
| **22** | $BiRh(esp)_2$ | 4.45[d] | [$D_8$]-THF | 6323 | H(C)Rh |

[a] M. B. Smith, March's Advanced Organic Chemistry: Reactions, Mechanisms, and Structure 7th Edition, Wiley, New York, 2001. [b] D.H. Ripin, D.A. Evans, pKa's of Inorganic and Oxo-Acids, available at http://ccc.chem.pitt.edu/wipf/MechOMs/evans_pKa_table.pdf [c] The arithmetic mean of the pKa values of the ligands were used [d] Predicted pKa values from Scifinder; Calculated using Advanced Chemistry Development (ACD/Labs) Software V11.02 (© 1994-2021 ACD/Labs)

# 4. Supporting NMR Spectroscopic Data

### Comments on the H(C)Rh sequence

Example parameters are shown in the following Table:

**Table S2**. Typical H(C)Rh acquisition parameters (example: $Rh_2(OAc)_4$ in $CD_3CN$)

**Channel**

| | $^{1}$**H** | $^{13}$**C** | $^{103}$**Rh** |
| --- | --- | --- | --- |
| | f1 | | f2 |
| *pulse program* | H(C)Rh | | |
| *Offset (ppm)* | 5 | 22.565 | -1000* |
| *SW (ppm)* | 20 | x | 500 |
| *TD* | 2048 | | 128 |
| *P90 (µs)* | 8.8 | 23.3 | 90 |
| *PL (W)* | 13 | 240 | 180 |
| *CPD* | | waltz16 (1120.00 µs, 0.157W) | |

** $\Xi(^{103}Rh)$= 3.186447%*

| | |
| --- | --- |
| NS | 8 |
| GPNAM | SMSQ10.100 |
| GPZ(1) | 80% |
| GPZ(2) | 80% |
| GPZ(3) | 5.06% |
| gradient pulse | 1 ms |
| $\Delta_1$ | 1.95 ms |
| $\Delta_2$ | 52 ms |
| NUS | 10% |

Generally, a second H(C)Rh with the same Rh offset O3, but different SW (normally 700 ppm) in the indirect dimension was acquired to make sure that the observed peak is not folded. For highly concentrated samples (e.g. sat. $Rh(acac)_3$ in $CDCl_3$), a significant $t_1$ noise from $^{1}H$ attached to $^{12}C$ was observed in the spectra. *Mobley*[14] suggested a modified gradient scheme in his work, which is aimed to suppress artefacts from direct $^{1}H$-X couplings due to a better coherence pathway selection at a cost of losing half of the signal. Although there was no residual $^{1}H$-$^{103}Rh$ coupling present in the systems, the $t_1$ noise was also significantly reduced. The alternative gradient ratios for the H(C)Rh sequence are the following: G1:G2:G3=75:42:14.2.

### HMQC Magnetisation Transfer – The Importance of $\Delta_2$

During the initial measurements, $\Delta_2 = 1/(4\times J_{CRh})$ was used in the H(C)Rh sequence for the $Rh_2(OAc)_4$ sample in $CD_3CN$. None of the two proposed magnetisation transfer pathways resulted in an observable NMR signal; only $t_1$ noise was observed. When taking a closer look at the magnetization transfer, a difference to mononuclear rhodium complexes becomes visible. In the latter case the magnetization is only transferred to a single $^{103}Rh$, similar to analogue classic $^{1}H$,$^{13}C$ measurements at natural abundance. In dirhodium paddlewheel systems, the magnetization is transferred from one carbon to two chemically equivalent $^{103}Rh$ nuclei. The magnetization transfer efficiency in the HMQC step of generic $IS_n$ spin systems has been investigated by *Xiang* et al.[15] Adapted for the H(C)Rh sequence, the cross peak intensity *I* can be described as following:

$$I \sim [\sin(\pi J_{CRh} 2\Delta_2)\cos^{n-1}(\pi J_{CRh} 2\Delta_2)]^2 \qquad \text{(I)}$$

whereby $2\Delta_2$ is the delay for the $^{13}C$-$^{103}Rh$ transfer in the pulse sequence, $J_{CRh}$ the $^{13}C$-$^{103}Rh$ coupling constant and *n* the chemically equivalent $^{103}Rh$ atoms coupled to $^{13}C$.

The theoretically optimal magnetization transfer delay for the $IS_2$ spin system in $Rh_2(OAc)_4$ is at $\Delta_2 = 1/(8\times J_{CRh})$. To verify this experimentally, $Rh_2(^{13}CH_3COO)_4$ and $Rh_2(CH_3{}^{13}COO)_4$ were used as model substrates. Different H(C)Rh spectra with different delays $\Delta_2$ were acquired and compared to the theoretical prediction (Figure S1). Experimentally it was found that an good cross peak intensity for both transfer pathways is obtained at a delay of $\Delta_2 = 1/(12\times J_{CRh})$, which does not match the theoretical prediction. As $J_{CRh}$ is small in the Rh(II) systems of interest (<1.7 Hz), the transverse multiple quantum relaxation, simplified denoted as $T_2^*$, has a significant contribution to the transfer efficiency. When adding a relaxation term to equation (I):

$$I_{\text{exp}} \sim I * e^{\frac{-4\Delta_2}{T_2^*}} \qquad \text{(II)}$$

the experimentally observed values can be described by the theoretical equation. The value $\Delta_2$ is multiplied by factor 4 as is present 4 times in the H(C)Rh pulse sequence. These results also show limitations of the general determination of $^{103}Rh$ shifts with the current H(C)Rh sequence. If $J_{CRh}$ becomes smaller or $T_2^*$ decreases further, the expected $^{103}Rh$ cross peak intensity becomes smaller and the detection of the peak more challenging.

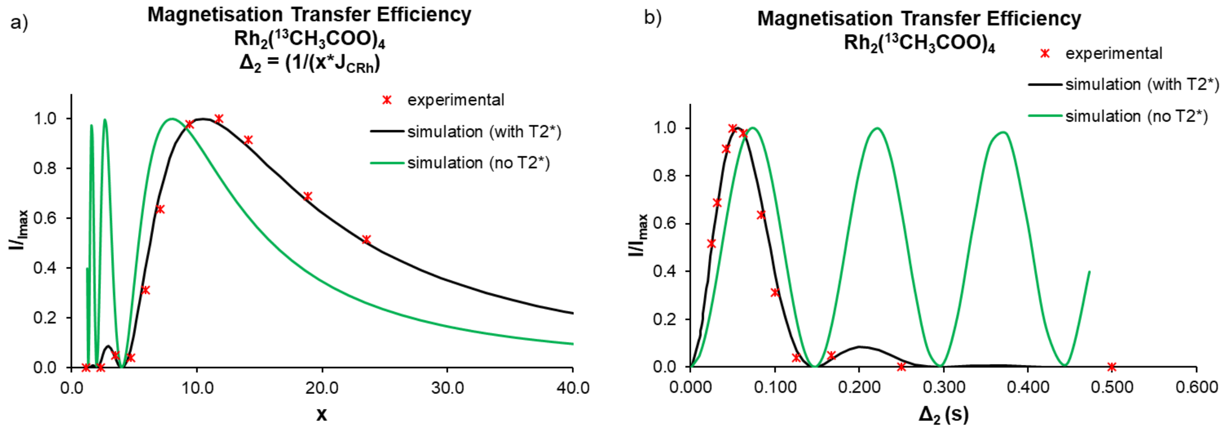


**Figure S1**. Analysis of the magnetization transfer efficiency from $^{13}C$ to $^{103}Rh$. Relative cross peak intensity $I/I_{max}$ plotted against a) x for $\Delta_2 = 1/(x \times J_{CRh})$ and b) against $\Delta_2$. Red crosses show the experimentally measured values, the green line the theoretical expectation for equation (I) and the black line the theoretical values for equation (II). Values used for the simulations: $n_{Rh} = 2$; $J_{CRh} = 1.7$ Hz, $T_2^* = 0.24$. Sample: 15 mM $Rh_2(^{13}CH_3COO)_4$ in $CD_3CN$

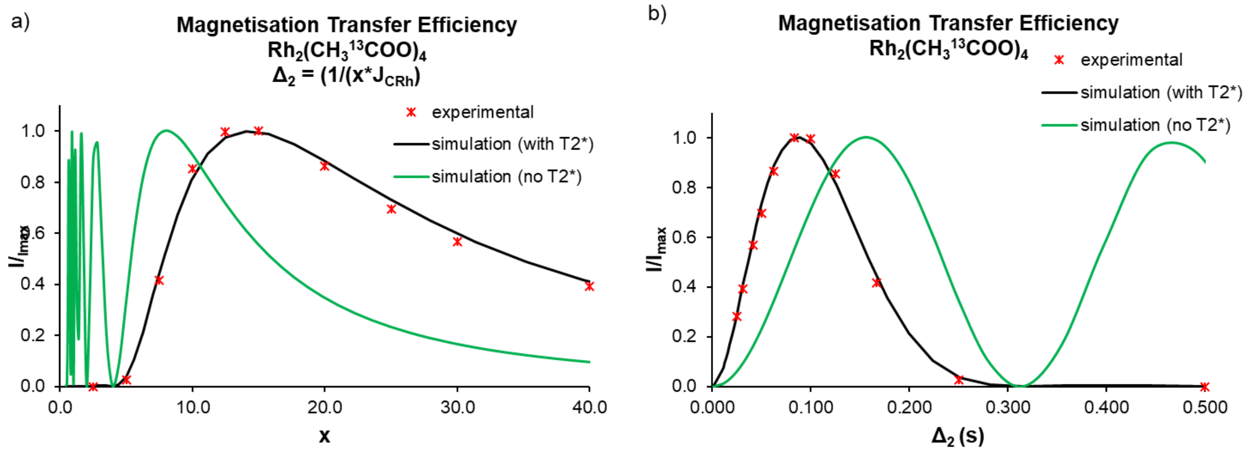


**Figure S2**. Analysis of the magnetization transfer efficiency from $^{13}C$ to $^{103}Rh$. Relative cross peak intensity $I/I_{max}$ plotted against a) x for $\Delta_2 = 1/(x \times J_{CRh})$ and b) against $\Delta_2$. Red crosses show the experimentally measured values, the green line the theoretical expectation for equation (I) and the black line the theoretical values for equation (II). Values used for the simulations: $n_{Rh} = 2$; $J_{CRh} = 0.8$ Hz, $T_2^* = 0.24$ s. Sample: 15 mM $Rh_2(CH_3{}^{13}COO)_4$ in $CD_3CN$

The detailed analysis and optimization of the delay $\Delta_2$ helped to significantly improve the cross peak signal intensity (factor >20) for $Rh_2({}^{13}CH_3COO)_4$ from the H(C)Rh sequence. Using the optimized parameters ($\Delta_2 = 1/(12*J_{CRh})$), it was possible to determine the $^{103}Rh$ chemical shieldings of a non-enriched sample of $Rh_2(OAc)_4$ sample as well as all other complexes shown in this manuscript.

# 5. Computational Study

The systems considered in this study are shown in Figure S3.

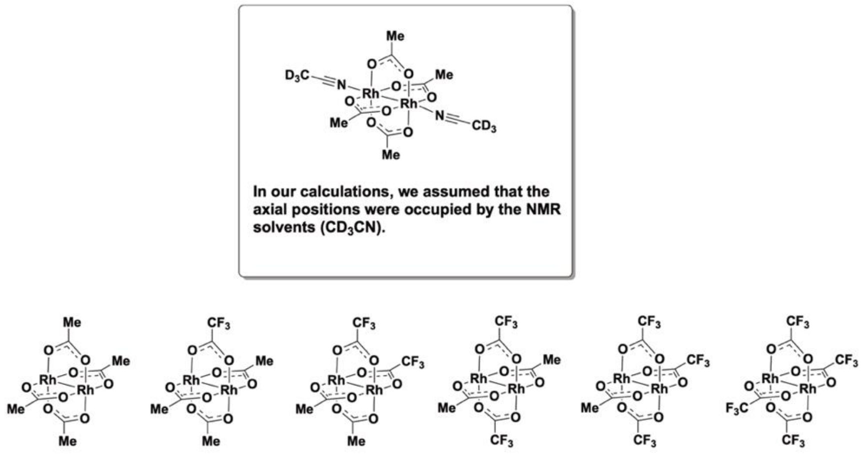


1 2 3 4 5 6

**Figure S3**. Systems studied in the present work

Note that the values of chemical shifts we compute using electronic structure theory which will be compared with experiment need to be referenced to a standard. For this purpose, we used the shielding of compound **1**, hence providing some compensation of systematic errors and ensuring optimal comparability. This is also done because of the special type of reference used for $^{103}$Rh chemical shifts (see Experimental Section). Furthermore, due to numerical influences in the optimization and the property calculations, one might observe small asymmetries in the shieldings for nuclei that should be equivalent by symmetry. While these are typically small, in the following we report the arithmetic averages throughout the main manuscript. Further details can be found in the computational section below.

### Choice of Basis Set and Influence of the DFT Integration Grid

The NMR chemical shift is a property that stresses the electron density close to the nucleus and has basis set requirements which are quite different from energies or geometry optimizations.[16] Hence, we have chosen to apply large triple-zeta quality basis sets, which were fully decontracted to allow for maximum flexibility in the core region.

Another important parameter besides the basis set is the integration grid used for computing numerical integrals. In order to assess the quality of the integration grid used in DFT, especially in conjunction with the ZORA approximation, we have computed the $^{103}$Rh NMR chemical shifts using the standard grid available in ORCA 5.0 (“defgrid 2”) in comparison to a tight integration grid that should provide results close to convergence. The results at the TPSS/unc-def2-TZVPP level of theory in conjunction with the geometry optimized using CPCM and CPCM for the GIAO-NMR calculation are shown in Table S2.

**Table S2** Comparison of results obtained with standard "defgrid2" and tight "defgrid3" DFT integration grid settings. Geometries obtained using CPCM, NMR shifts calculated at the TPSSh/unc-def2-TZVPP (incl. CPCM, "defgrid3" settings).

| | *defgrid2 settings* | | | | *defgrid3 settings* | | | |
|---|---|---|---|---|---|---|---|---|
| Comp. | $\sigma_{iso}$ $Rh_1$ | $\sigma_{iso}$ $Rh_2$ | $\sigma_{iso}$ avg | $\delta_{iso}$ | $\sigma_{iso}$ $Rh_1$ | $\sigma_{iso}$ $Rh_2$ | $\sigma_{iso}$ avg | $\delta_{iso}$ |
| **1** | -6841.63 | -6994.49 | -6918.06 | 0.00 | -6856.62 | -6863.74 | -6860.18 | 0.00 |
| **2** | -6975.99 | -6895.05 | -6935.52 | 17.46 | -6936.29 | -6943.07 | -6939.68 | 79.50 |
| **3** | -7004.31 | -7051.59 | -7027.95 | 109.89 | -7009.53 | -7014.65 | -7012.09 | 151.91 |
| **4** | -7010.67 | -7127.20 | -7068.93 | 150.88 | -7037.21 | -7021.84 | -7029.53 | 169.35 |
| **5** | -7325.22 | -6976.30 | -7150.76 | 232.70 | -7129.95 | -7139.39 | -7134.67 | 274.49 |
| **6** | -7257.47 | -7195.02 | -7226.25 | 308.19 | -7207.44 | -7204.24 | -7205.84 | 345.66 |

Overall, the effect of a tighter integration is substantial in the absolute shieldings and still amounts to 50 ppm for the relative shifts with regard to compound **1**. As the computational effort is not drastically increased if the "defgrid 3" settings are employed, these will be used in the following, assuming that grid size effects are by far smaller when going to even tighter integration grids. Note that applying less dense grids for the evaluation of NMR chemical shifts in this case also leads to stronger asymmetry in the equivalent $^{103}$Rh shifts for species such as compound **1**, which is also why standard grids are not recommended in this case.

### Effect of Relativistic Contributions to the $^{103}$Rh NMR Chemical Shifts

In order to assess the importance of relativistic effects, we have applied the Zeroth Order Regular Approximation (ZORA) developed by van Lenthe et al.[17,18,19] and extended to NMR Chemical shifts using the GIAO formalism as described by Wolff, Ziegler, van Lenthe and Baerends.[20,21] As we have used a new re-implementation of the scheme described in the ORCA 5.0 package, we will give some details of this implementation here. In principle, our implementation strictly follows the GIAO formalism outlined in the literature,[22,23] applying the ZORA Hamiltonian in the SCF for the calculation of the density. The ZORA Hamiltonian is defined as follows :

$$h^{ZORA}(\pi) = \boldsymbol{\sigma} \cdot \boldsymbol{\pi} \frac{K}{2} \boldsymbol{\sigma} \boldsymbol{\pi} + V \tag{1}$$

$$K = \left(1 - \frac{V}{2c^2}\right)^{-1} \tag{2}$$

with the typical scaling factor K which goes to 1 in the non-relativistic limit. For the analytical evaluation of the GIAO NMR chemical shifts, in addition to including the ZORA Hamiltonian terms in the CPSCF,

this leads to modified operators required for the computation of the para- and diamagnetic contributions of the shielding tensor. Equations 3-8 illustrate this difference in the operators.

$$\sigma^d = \sum_{\mu\nu} P_{\mu\nu} \langle\mu| \frac{\partial^2 h^{11}}{\partial \mathbf{B_i} \partial \mathbf{m_K}} |\nu\rangle \tag{3}$$

$$\left(\frac{\partial^2 h^{11}_{\mu\nu}}{\partial \mathbf{B} \partial \mathbf{m_k}}\right)_{nrel} = \frac{\alpha^2}{2} \langle\mu| \frac{(\mathbf{r}_k \cdot \mathbf{r}_N)\mathbf{1} - \mathbf{r}_k \tilde{\mathbf{r}}_N + i\mathbf{Q}_{MN}\mathbf{r}\tilde{\mathbf{L}}_K}{r_K^3} |\nu\rangle \tag{4}$$

$$\left(\frac{\partial^2 h^{11}_{\mu\nu}}{\partial \mathbf{B} \partial \mathbf{m_k}}\right)_{ZORA} = \frac{\alpha^2}{2} \langle\mu| \frac{K[(\mathbf{r}_k \cdot \mathbf{r}_N)\mathbf{1} - \mathbf{r}_k \tilde{\mathbf{r}}_N] + iK\mathbf{Q}_{MN}\mathbf{r}\tilde{\mathbf{L}}_K}{r_K^3} |\nu\rangle \tag{5}$$

$$\sigma^p = \sum_{\mu\nu} \tilde{P}_{\mu\nu} \langle\mu| \frac{\partial h^{01}}{\partial \mathbf{m_K}} |\nu\rangle \tag{6}$$

$$\left(\frac{\partial h^{01}_{\mu\nu}}{\partial \mathbf{m_K}}\right)_{nrel} = \alpha^2 \langle\mu| \frac{\mathbf{r_N} \times \mathbf{p}}{r_N^3} |\nu\rangle \tag{7}$$

$$\left(\frac{\partial h^{01}_{\mu\nu}}{\partial \mathbf{m_K}}\right)_{ZORA} = \alpha^2 \langle\mu| \frac{iK}{4} \frac{\mathbf{r_N} \times \mathbf{p}}{r_N^3} |\nu\rangle \tag{8}$$

The dia- and paramagnetic contributions to the shielding constants are evaluated as the corresponding derivatives of the density and operators, including the density and the perturbed density evaluated in the CPSCF equations with the magnetic field components as perturbation (eq. 3 and 6). The property integrals required for the evaluation of the contributions are given in equations 4,5 and 7,8 for the dia- and paramagnetic contributions (using Gauge Including Atomic Orbitals). Here one can see that the basic difference in the operator derivatives arise from the scaling with K.

Due to this feature, the corresponding property integrals are typically not evaluated analytically, and our new implementation is based on a numerical integration for the evaluation of the operators from equations 5 and 8. However, as these property integrals exhibit an extremely steep dependence with 1/r, the numerical integration for ZORA requires special care. In order to avoid problems with numerical integration of singular functions, the ORCA 5.0 implementation is built around the "subtraction of singularity" strategy, subtracting the analytical non-ZORA integrals from the ZORA counterpart, such that effectively only the numerical integration of the additional ZORA contribution enters the resulting integral, hence making to solution robust and accurate for the standard grids used in the ORCA program package.[24]

**Table S3**. Comparison of results obtained with and without the ZORA relativistic approximation. Geometries obtained using CPCM, NMR shifts calculated at the TPSSh/unc-def2-TZVPP (incl. CPCM, “defgrid3” settings)

| | non-relativistic | | | | ZORA | | | |
|---|---|---|---|---|---|---|---|---|
| Comp. | $\sigma_{iso}$ $Rh_1$ | $\sigma_{iso}$ $Rh_2$ | $\sigma_{iso}$ avg | $\delta_{iso}$ | $\sigma_{iso}$ $Rh_1$ | $\sigma_{iso}$ $Rh_2$ | $\sigma_{iso}$ avg | $\delta_{iso}$ |
| **1** | -6829.88 | -6829.77 | -6829.82 | 0.00 | -6856.62 | -6863.74 | -6860.18 | 0.00 |
| **2** | -6899.32 | -6907.50 | -6903.41 | 73.59 | -6936.29 | -6943.07 | -6939.68 | 79.50 |
| **3** | -6977.74 | -6981.48 | -6979.61 | 149.79 | -7009.53 | -7014.65 | -7012.09 | 151.91 |
| **4** | -7009.36 | -6991.39 | -7000.37 | 170.55 | -7037.21 | -7021.84 | -7029.53 | 169.35 |
| **5** | -7094.62 | -7111.26 | -7102.94 | 273.12 | -7129.95 | -7139.39 | -7134.67 | 274.49 |
| **6** | -7181.85 | -7181.79 | -7181.82 | 352.00 | -7207.44 | -7204.24 | -7205.84 | 345.66 |

While this conceptually simple scalar relativistic approach does not contain higher-order or spin-orbit-coupling effects, the results in Table S4 show that relativistic effects should be small in general, as the ZORA correction only amounts to changes of less than 7 ppm and typically 2-3 ppm for the shifts. Hence, we assume that relativistic effects are small and captured sufficiently well by using the ZORA approximation in our case.

### Influence of Reference Geometry and CPCM in the Computed NMR Shieldings

In order to assess the importance of solvent effects for the computation of the NMR shieldings, three schemes are compared in the following: (1) Optimizing the molecular geometry without any solvent correction and computing the NMR chemical shifts at a given level of theory, (2) optimizing the geometry including an implicit solvent correction and then computing the NMR chemical shifts at the same level of theory, and (3) optimizing the geometry with implicit solvation and including the implicit solvation also in the calculation of the NMR chemicals shifts.

**Table S4.** Comparison of the TPSSh results using geometries and NMR chemical shifts computed with and witout using an implicit solvation model. Right column: experimental reference values. Level of theory: TPSSh/unc-def2-TZVPP (inc. ZORA, “defgrid3” settings)

| | Geometry and shieldings without implicit solvation | | | | Geometry obtained using CPCM | | | | Geometry obtained using CPCM, shieldings computed incl. CPCM | | | | |
|---|---|---|---|---|---|---|---|---|---|---|---|---|---|
| Comp. | $\sigma_{iso}$ $Rh_1$ | $\sigma_{iso}$ $Rh_2$ | $\sigma_{iso}$ avg | $\delta_{iso}$ | $\sigma_{iso}$ $Rh_1$ | $\sigma_{iso}$ $Rh_2$ | $\sigma_{iso}$ avg | $\delta_{iso}$ | $\sigma_{iso}$ $Rh_1$ | $\sigma_{iso}$ $Rh_2$ | $\sigma_{iso}$ avg | $\delta_{iso}$ | $\delta_{iso}$ exp. |
| **1** | -6811.49 | -6816.69 | -6814.09 | 0.00 | -6928.49 | -6933.55 | -6931.02 | 0.00 | -6856.62 | -6863.74 | -6860.182 | 0 | 0.0 |
| **2** | -6877.52 | -6889.10 | -6883.31 | 69.22 | -6982.18 | -6990.68 | -6986.43 | 55.41 | -6936.29 | -6943.07 | -6939.682 | 79.5 | 77.8 |
| **3** | -7033.37 | -7039.11 | -7036.24 | 222.15 | -7033.37 | -7039.11 | -7036.24 | 105.22 | -7009.53 | -7014.65 | -7012.090 | 151.91 | 161.2 |
| **4** | -6959.61 | -6944.89 | -6952.25 | 138.16 | -7054.54 | -7033.55 | -7044.04 | 113.02 | -7037.21 | -7021.84 | -7029.529 | 169.35 | 184.3 |
| **5** | -7039.34 | -7045.30 | -7042.32 | 228.23 | -7118.08 | -7129.08 | -7123.58 | 192.56 | -7129.95 | -7139.39 | -7134.672 | 274.49 | 269.4 |
| **6** | -7115.47 | -7110.21 | -7112.84 | 298.75 | -7161.16 | -7156.37 | -7158.76 | 227.74 | -7207.44 | -7204.24 | -7205.842 | 345.66 | 381.7 |

The results are displayed in Table S4 together with the experimental shifts. It becomes obvious, that a critical ingredient for good agreement between theory and experiment are well-converged geometries that include the most important contributions for the effective effects of the solvent including implicit solvent corrections for the computation of the actual shieldings.

## Influence of the Functional

In recent studies performed in our group, the TPSS and TPSSh functional have been found to offer very good accuracy at low computational cost for the calculation of a broad range of NMR chemical shifts, including several elements besides Carbon and Hydrogen.[25] For this reason, we have chosen to use these functionals. Table S5 displays the results for the TPSS and TPSSH functional in comparison to experiment. While the difference is noticable but not large, the TPSSH results show better agreement with experiment. This indicates that the obtained trends are fairly robust with choice of functional, while the functional which is considered as “higher rung” functional also delivers more accurate results.[26]

**Table S5**. Comparison between computed and experimental $^{103}$Rh NMR chemical shifts at the TPSS and TPSSh level of theory (“defgrid3” settings, geometry and shieldings obtained using CPCM, shieldings calculated using the unc-def2-TZVPP basis and ZORA approximation).

| | TPSS | | | | TPSSh | | | | |
|---|---|---|---|---|---|---|---|---|---|
| Comp. | $\sigma_{iso}$ $Rh_1$ | $\sigma_{iso}$ $Rh_2$ | $\sigma_{iso}$ avg | $\delta_{iso}$ | $\sigma_{iso}$ $Rh_1$ | $\sigma_{iso}$ $Rh_2$ | $\sigma_{iso}$ avg | $\delta_{iso}$ | $\delta_{iso}$ exp. |
| **1** | -6240.09 | -6247.56 | -6243.82 | 0.00 | -6856.62 | -6863.74 | -6860.18 | 0.00 | 0.0 |
| **2** | -6315.27 | -6320.12 | -6317.69 | 73.87 | -6936.29 | -6943.07 | -6939.68 | 79.50 | 77.8 |
| **3** | -6381.42 | -6386.36 | -6383.89 | 140.07 | -7009.53 | -7014.65 | -7012.09 | 151.91 | 161.2 |
| **4** | -6404.25 | -6392.81 | -6398.53 | 154.71 | -7037.21 | -7021.84 | -7029.53 | 169.35 | 184.3 |
| **5** | -6492.35 | -6497.53 | -6494.94 | 251.11 | -7129.95 | -7139.39 | -7134.67 | 274.49 | 269.4 |
| **6** | -6559.74 | -6556.81 | -6558.28 | 314.46 | -7207.44 | -7204.24 | -7205.84 | 345.66 | 381.7 |

## Analysis of the NMR Shielding Tensors using Ramsey’s Expression

To gain a deeper insight into the relationship between the NMR chemical shifts and the electronic structure of these complexes, it is useful to analyze the principal components of the shielding tensor (Table S7). A graphical representation of the data is shown in Figure S4. For the sake of simplicity, only the paramagnetic contributions of the shielding tensor are discussed (the diamagnetic component is small and roughly constant for all the systems investigated here, as shown in Table S7).

**Table S6**. Decomposition of the paramagnetic $^{103}Rh$ NMR $\delta_{para,\ calc}$ shift into its principal tensor components, panel on the right displays the anisotropy of the $^{103}Rh$ shielding tensor using compound **1** as example.

| Compound | $\delta_{para,\ calc}$ | $\delta_{dia,\ calc}$ | $\delta_{xx,\ calc}$ | $\delta_{yy,\ calc}$ | $\delta_{zz,\ calc}$ |
|---|---|---|---|---|---|
| **1** | 0 | 0 | 0 | 0 | 0 |
| **2** | 80 | 0 | 117 | -2 | 112 |
| **3** | 153 | 1 | 110 | 111 | 196 |
| **4** | 171 | 2 | 263 | -15 | 237 |
| **5** | 277 | 3 | 307 | 146 | 367 |
| **6** | 349 | 3 | 306 | 310 | 487 |

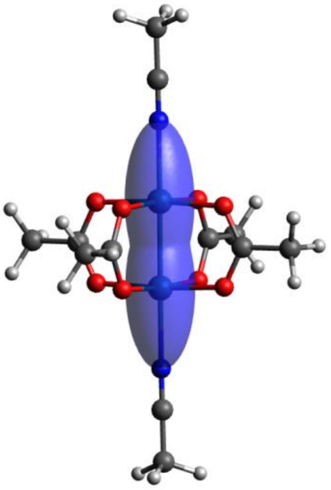

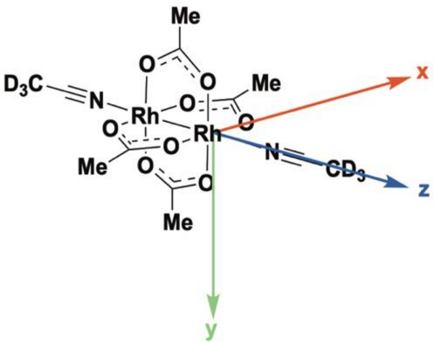


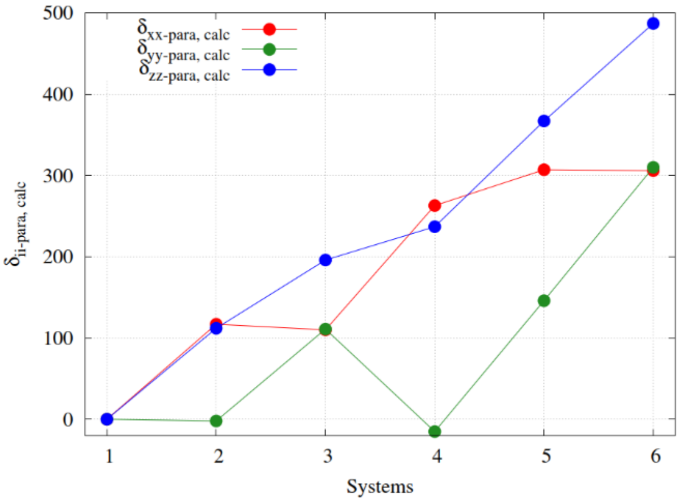


**Figure S4**. Left: The principal axis of the shielding tensor of Rh; right: the paramagnetic contribution to each principal component of the shielding tensor for the systems investigated here.

It is important to emphasize that the individual principal components of different systems can be safely compared because the principal axes coincide in all cases. The orientation of the principal axes is dictated by the local symmetry at the Rh center, and all the systems investigated here are classified by the lowest common point group, which is $C_{2v}$.

The individual components of the shielding tensor show non-intuitive variations along the series:

I. $\delta_{zz,calc}$ is the only component that shows a qualitative linear correlation with the total chemical shift. It increases with increasing the fluorination.

II. $\delta_{yy,\ calc}$ is roughly constant for complexes **1, 2** and **4,** which are not fluorinated along the x-axis

III. $\delta_{xx,\ calc}$ is roughly constant for complexes **4,5,6**, which are all fully fluorinated along the y-axis.

These variations can be interpreted in a simple orbital picture using the Ramsey formula:[27]

$$\sigma_{ii-para} = \sum_{virt}\sum_{occ} \frac{\langle \varphi_{occ}|\widehat{L_\iota}|\varphi_{vir}\rangle\langle \varphi_{vir}|\widehat{L_\iota}/r^3|\varphi_{occ}\rangle}{E_{vir-}E_{occ}}$$

In which $\Psi_{occ}$ and $\Psi_{vir}$ denote occupied and virtual orbitals, respectively; $E_{occ}$ and $E_{vir}$ represent the corresponding orbital energies and $\widehat{L_\iota}$ is the angular momentum operator. Thus, deshielding in the direction $\sigma_{ii,para}$ depends on which $\Psi_{occ}$and $\Psi_{vir}$ orbitals can be coupled by the corresponding $\widehat{L_\iota}$ operator and by the relative energy between the orbitals.

For the complexes studied here, the three occupied molecular orbitals of highest energy correlate with the $d_{xy}$, $d_{xz}$ and $d_{yz}$ orbitals of Rh, whilst the virtual orbitals correlate with the $d_{x2-y2}$ and $d_{z2}$ (Figure S5).

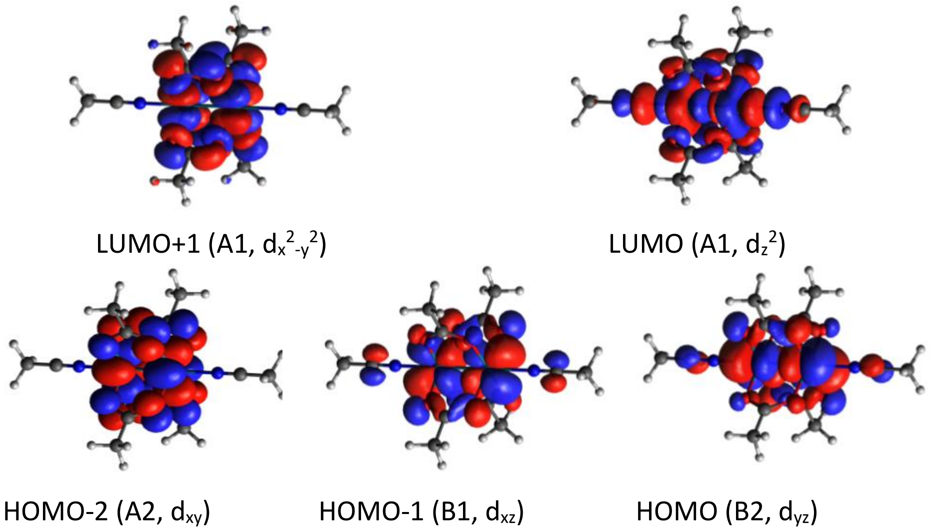


**Figure S5.** Key frontier orbitals for compound **1**. The associated irreducible representation in the $C_{2v}$ molecular point group is reported in brackets. The Rh d orbital associated with each molecular orbital is also shown in brackets.

As all complexes studied here are classified as $C_{2v}$ symmetry, these orbitals transform like the irreducible representations of this molecular point group. Thus, the $d_{xy}$, $d_{xz}$, $d_{yz}$, $d_{x2-y2}$ and $d_{z2}$ orbitals of Rh feature A2, B1, B2, A1 and A1 symmetry, respectively. The angular momentum operator belongs to the same irreducible representation of the corresponding rotation operator,[28] i.e., B2, B1 and A2 for $L_X$, $L_Y$and $L_Z$, respectively. By evaluating which products of irreducible representations of orbtials and operator yield the totally symmetric representation, it is found that the only non-zero contributions from the orbitals to the $\sigma_{xx,para}$, $\sigma_{yy,para}$ and $\sigma_{zz,para}$ components of the shielding tensor stem from the virtual orbitals with the occupied $d_{yz}$ and $d_{xz}$ and $d_{xy}$ orbitals, respectively.

Thus, a detailed analysis of shielding tensor can provide unique information into the nature of the bond between the Rh and the ligands. In fact, for symmetry reasons, the $d_{xy}$ orbital of Rh can mix with the ligands on both axis, whilst the $d_{xz}$ and $d_{yz}$ orbitals only mix with the orbitals of the ligands on the x- and y-axis, respectively. As a consequence, fluorination will always affect $\delta_{zz,}$ irrespective of the position of the substitution, whilst $\sigma_{xx,para}$ and $\sigma_{yy,para}$ mainly respond to fluorination on the

perpendicular y- and x-axis, respectively. These results demonstrate the connection between the experimentally observed shifts and the nature of the Rh-ligand bond for these complexes.

Note that this applies to all compounds that were analyzed (**1**-**6**). For comparison, the frontier orbitals for all computed compounds are displayed in below:

## Compound 1

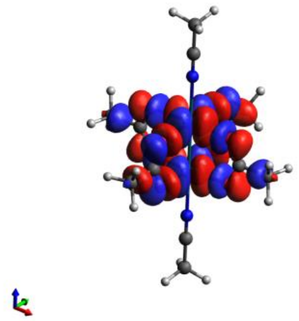

$d_{x^2-y^2}$ LUMO+2

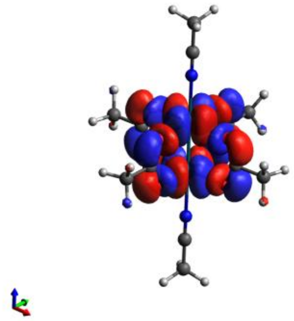

$d_{x^2-y^2}$ LUMO+1

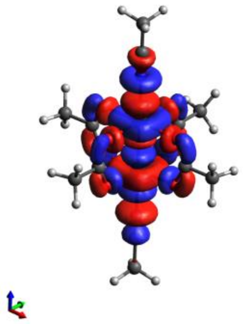

$d_{z^2}$ LUMO

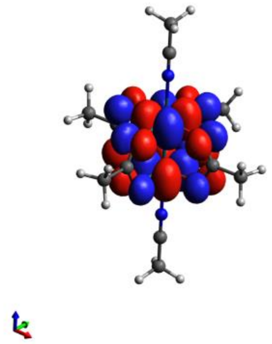

$d_{xy}$ HOMO-2

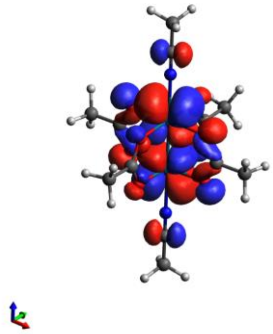

$d_{xz}$ HOMO-1

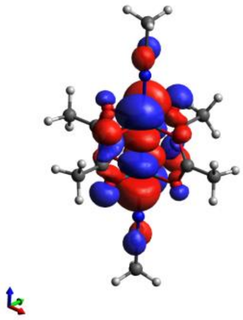

$d_{yz}$ HOMO

**Compound 2**

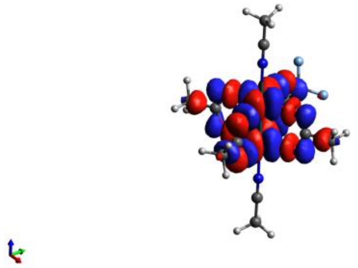
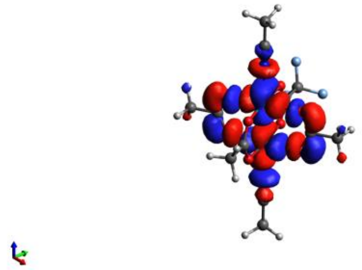
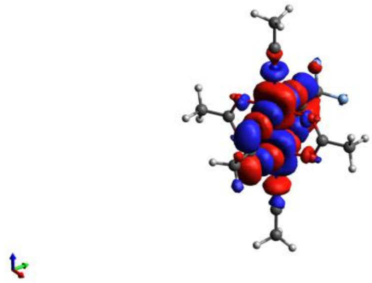

$d_{x^2-y^2}$ LUMO+2 $d_{x^2-y^2}$ LUMO+1 $d_{z^2}$ LUMO

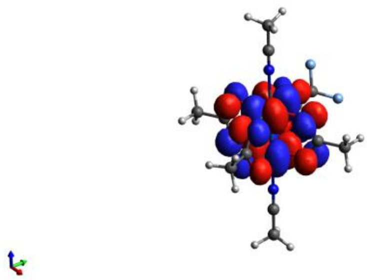
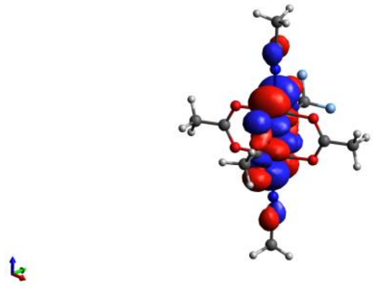
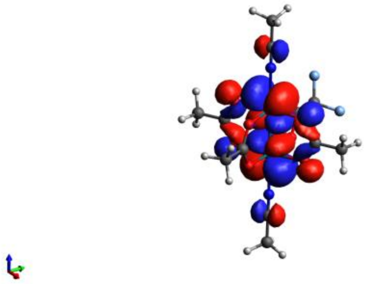

$d_{xy}$ HOMO-2 $d_{yz}$ HOMO-1 $d_{xz}$ HOMO

**Compound 3**

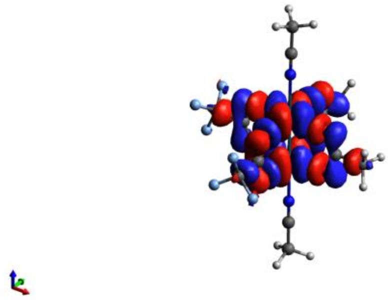
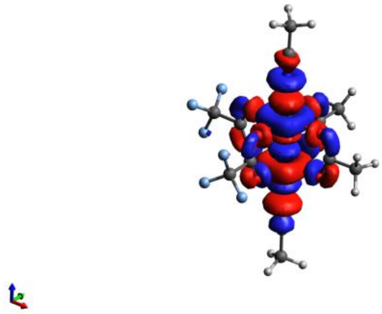
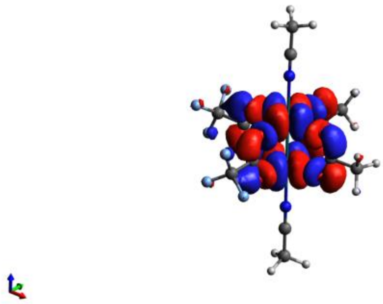

$d_{x^2-y^2}$ LUMO+2 $d_{z^2}$ LUMO+1 $d_{x^2-y^2}$ LUMO

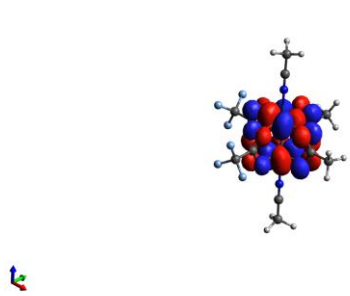
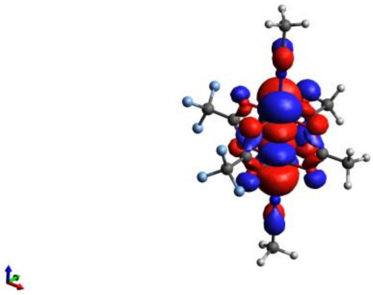
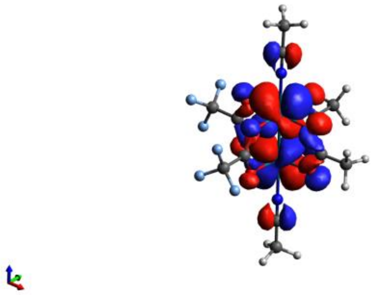

$d_{xy}$ HOMO-2 $d_{yz}$ HOMO-1 $d_{xz}$ HOMO

**Compound 4**

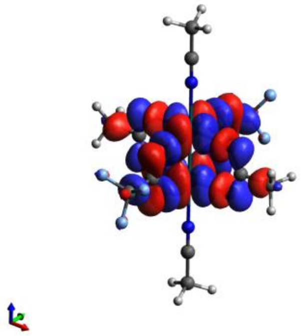

$d_{x^2-y^2}$ LUMO+2

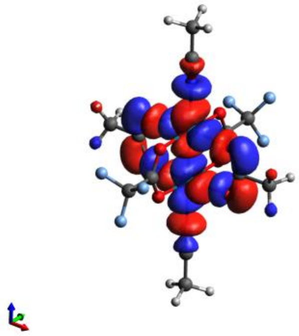

$d_{x^2-y^2}$ LUMO+1

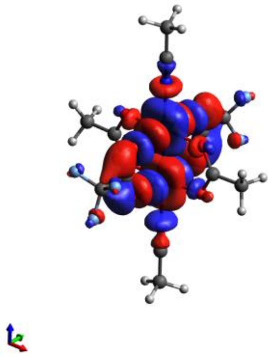

$d_{x^2}$ LUMO

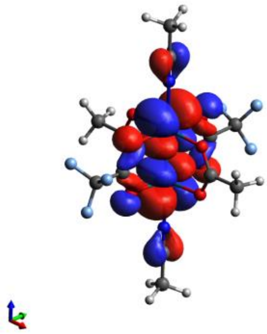

$d_{yz}$ HOMO-2

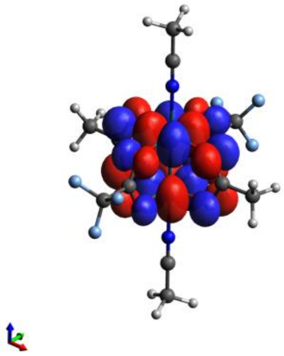

$d_{xy}$ HOMO-1

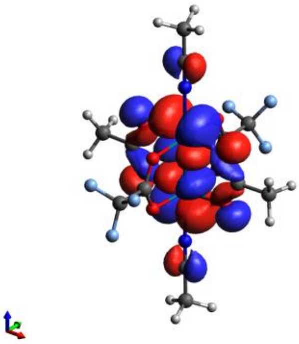

$d_{xz}$ HOMO

**Compound 5**

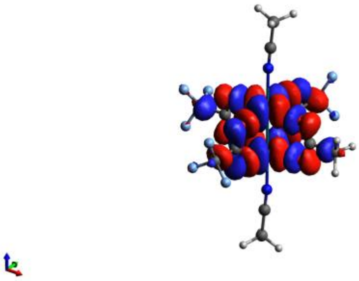

$d_{x^2-y^2}$ LUMO+2

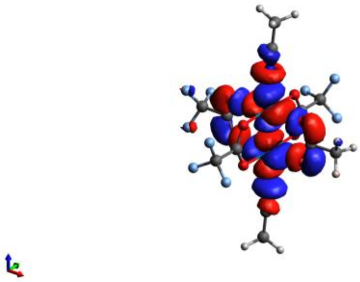

$d_{x^2-y^2}$ LUMO+1

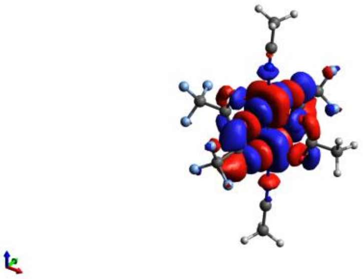

$d_{x^2}$ LUMO

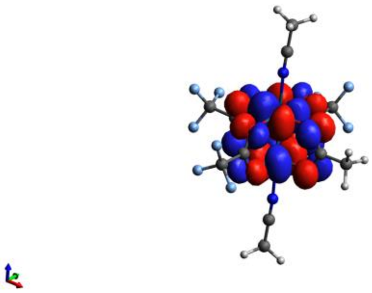

$d_{xy}$ HOMO-2

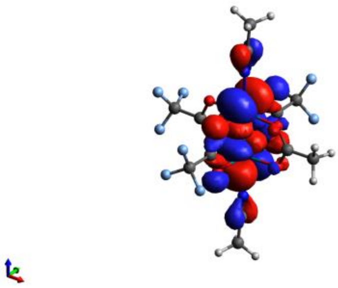

$d_{yz}$ HOMO-1

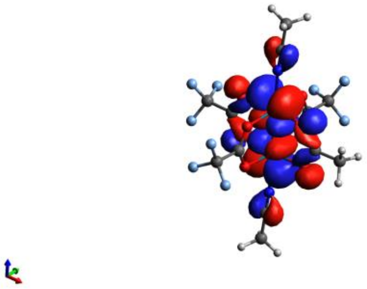

$d_{xz}$ HOMO

## Compound 6

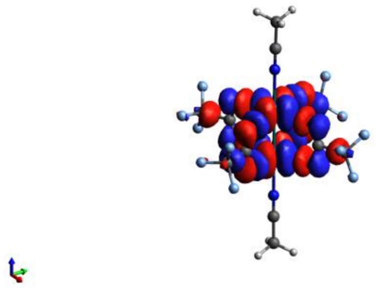 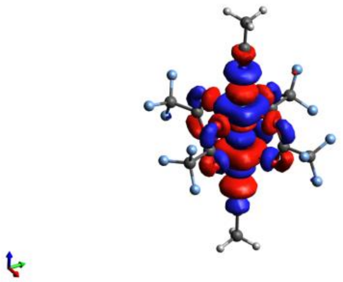 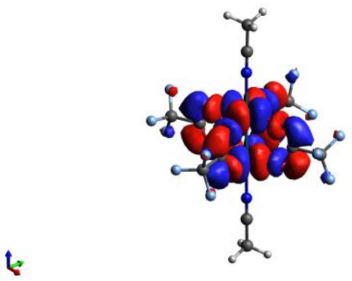

$d_{x^2-y^2}$ LUMO+2 $d_{z^2}$ LUMO+1 $d_{x^2-y^2}$ LUMO

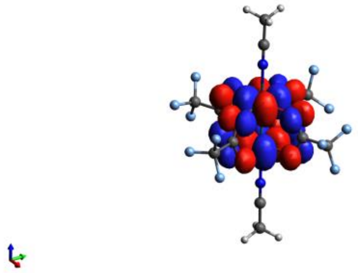 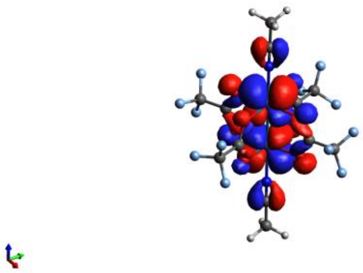 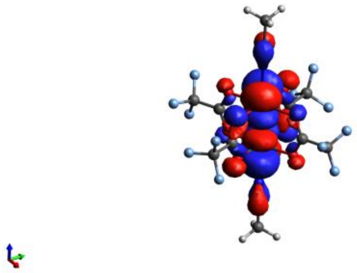

$d_{xy}$ HOMO-2 $d_{xz}$ HOMO-1 $d_{yz}$ HOMO

**Computational Details**

The computational study has been carried out using a local development version of the ORCA 5.0 program package. All geometries have been optimized at the B3LYP-D3/def2-tzvp level of theory[29,30,31,32,33] employing very tight convergence criteria for SCF and structure optimization and very conservative DFT grids. In order to account for the effect of solvation, the CPCM model (acetonitrile)[34] has been applied. All minima have been confirmed by normal mode analysis.

GIAO NMR chemical shift calculations[35,36,37,38,39] have been carried out using the TPSS and TPSSH functional[40,41,42] employing the RI and RIJCOSX approximations as previous benchmarks have demonstrated good performance of this family of functionals.[25] In order to provide a sufficiently flexible basis set for the description of the 103Rh NMR chemical shifts, the SARC-ZORA-TZVPP basis set[43] has been used for Rh, while the def2-TZVPP basis set[32,33] has been chosen for all other nuclei and all basis set have been decontracted and complemented with the Autoaux auxiliary basis sets[44] available in ORCA. In order to assess whether scalar relativistic effects have a considerable effect on the relative chemical shifts, the ZORA approximation has been employed. This includes a new local implementation of the computation of NMR chemical shieldings following the scheme of Barends et al. (see above).[45] For all NMR calculations very tight SCF convergence and tight integration grid settings have been employed in conjunction with the CPCM model (Acetonitrile), see above.

**Geometries and complete set of NMR chemcial shifts**

# 1

```
42
  C   6.21271631411349      0.45591405129841      3.13115275989291
  C   7.13565441100939      1.54891553538627      3.59504547055573
  O   6.99648580944699      1.95973526327767      4.78532550666839
  Rh  8.26582457587901      3.40645381622545      5.51760407127364
  O   9.60971942897008      4.85159877707754      6.10492526302135
  C   10.47812593188086      5.26884080108469      5.28222388264636
  C   11.40068458354789      6.36209359243894      5.74618322969183
  Rh  9.34785751243447      3.41084542035957      3.35947627034676
  O   10.61736101551509      4.85781007738536      4.09200015565479
  O   8.00403890920208      1.96600352215534      2.77225059109897
  O   8.01309512634564      4.86825957183082      2.78294118308944
  C   7.14677374803361      5.28434130374059      3.60842367716209
  C   6.23022138356561      6.38581899011324      3.15205435861836
  O   10.60880103883995      1.95157996177410      4.08106083933558
  C   10.47935589216711      1.54669375544230      5.27445752448014
  C   11.46686836787327      0.52570483560406      5.76838519687862
  O   7.00539128210615      4.86610766739902      4.79586310266079
  O   9.60125848264930      1.94953129059200      6.09407698404827
  H   6.08413572652533      0.49570369977689      2.05150742890686
  H   6.66196235131068     -0.50609713916576      3.39061398918534
  H   5.25061531861972      0.53055720690888      3.63420401914898
  H   11.01599277079829     -0.10239178715463      6.53415106260568
  H   11.83492969667629     -0.08101247374357      4.94361238152137
  H   12.31400039915283      1.05452829087162      6.21262344092124
  H   12.36160307288117      6.29014532320341      5.24053265908971
  H   10.94918372091912      7.32401267611750      5.49030845673235
  H   11.53200767433862      6.31996893395958      6.82542581394581
  H   5.26865534937833      6.31523794708989      3.65666454465100
  H   6.68669193820047      7.34337470609975      3.41540852304300
  H   6.09930070157134      6.35249853354603      2.07246237548519
  N   7.25078774156462      3.40203120713788      7.52124236640498
  C   6.71474791675285      3.39631652598117      8.53626346220451
  C   6.03698725282983      3.38936019499697      9.81703691492973
  H   6.32360656934793      4.27237357658375     10.38917510188215
  H   4.95776637258754      3.39651016416145      9.66049420692786
  H   6.31398607364154      2.49391100720482     10.37440725507174
  N   10.36290346243928      3.41511562335444      1.35588600153984
  C   10.89805744386133      3.41933698561603      0.34039365039007
  C   11.57472499625445      3.42431161621144     -0.94095972803321
  H   11.28751382369458      2.54045605231228     -1.51150070248712
  H   11.29733161146080      4.31894016523127     -1.49944648632726
  H   12.65407620161112      3.41728473051288     -0.78531980486479
```

| Nucleus | Element | Isotropic | Anisotropy |
|---|---|---|---|
| 0 | C | 161.281 | 49.169 |
| 1 | C | -9.519 | -78.215 |
| 2 | O | 128.544 | 354.120 |
| 3 | Rh | -6856.621 | -6449.175 |
| 4 | O | 135.103 | 373.065 |
| 5 | C | -9.356 | -82.514 |
| 6 | C | 161.885 | 48.055 |
| 7 | Rh | -6863.742 | -6415.878 |
| 8 | O | 138.106 | 372.801 |
| 9 | O | 129.007 | 354.358 |
| 10 | O | 128.446 | 362.825 |
| 11 | C | -9.371 | -79.801 |

| | | | |
|---|---|---|---|
| 12 | C | 161.534 | 49.070 |
| 13 | O | 136.529 | 364.327 |
| 14 | C | -9.546 | -77.027 |
| 15 | C | 161.658 | 48.349 |
| 16 | O | 129.620 | 361.773 |
| 17 | O | 135.952 | 366.432 |
| 18 | H | 29.896 | 6.445 |
| 19 | H | 29.596 | 5.562 |
| 20 | H | 29.855 | 6.427 |
| 21 | H | 29.950 | 6.449 |
| 22 | H | 29.961 | 6.414 |
| 23 | H | 29.752 | 5.761 |
| 24 | H | 29.998 | 6.212 |
| 25 | H | 29.843 | 6.106 |
| 26 | H | 29.997 | 6.317 |
| 27 | H | 29.926 | 6.427 |
| 28 | H | 29.697 | 5.471 |
| 29 | H | 29.969 | 6.478 |
| 30 | N | 33.220 | 404.643 |
| 31 | C | 66.434 | 328.934 |
| 32 | C | 185.801 | 26.384 |
| 33 | H | 29.316 | 9.892 |
| 34 | H | 29.291 | 9.871 |
| 35 | H | 29.310 | 9.943 |
| 36 | N | 33.095 | 405.299 |
| 37 | C | 66.536 | 329.065 |
| 38 | C | 185.736 | 26.516 |
| 39 | H | 29.312 | 9.924 |
| 40 | H | 29.319 | 9.871 |
| 41 | H | 29.319 | 9.902 |

**2**

```
42
Coordinates from ORCA-job opt
  C   6.23637459599114      0.44581084346502      3.09604602972484
  C   7.18279765912262      1.57629295336362      3.57544515177191
  O   8.02868657996944      1.97242826331893      2.74502099699637
  Rh  9.36708813047956      3.44116859978985      3.36369033363655
  N   10.39409544113958      3.46811201070728      1.37409447457554
  C   10.94023259748188      3.46935361955494      0.36478617192769
  C   11.63102432794518      3.47016402493052     -0.90861926507162
  O   7.00213861193787      1.93817059516663      4.75956023376335
  Rh  8.26827115880829      3.40555958883064      5.52055110801448
  N   7.25153700320530      3.38556250992207      7.51442974889142
  C   6.72889522621999      3.39798403242903      8.53600911138546
  C   6.07002822237310      3.41512186937593      9.82608573376822
  O   9.58812697944779      4.84356263130188      6.12214471812586
  C   10.45606182107827      5.28021003407642      5.30849758615841
  O   10.60346436842026      4.88376608888646      4.11382553877290
  O   7.00253889159473      4.85964371309625      4.80219095911045
  C   7.14800103009664      5.29151746911562      3.61959438322145
  C   6.22728764636670      6.39038207578320      3.16885849314888
  O   9.61070093396840      1.95484787288203      6.08802427801682
  C   10.48873875661129      1.55681336666299      5.26523834457737
  C   11.41224523676343      0.46059357006873      5.71677511820940
  C   11.36487224535842      6.37577297556412      5.78763684153975
  O   8.02189956752225      4.88877063362724      2.79459423929986
  O   10.63500532458410      1.98970107937825      4.08306833636438
  F   6.24030482316062      0.31287705237025      1.76573827368905
  F   6.62791530274919     -0.72856536180373      3.62955569117795
  F   4.97298046997593      0.67059334438965      3.48659796752185
  H   11.53280891621390      0.48303303807796      6.79777560087071
  H   10.96768161364621     -0.49815023835885      5.43789489683380
  H   12.37711979816674      0.54750658366108      5.22124933941679
  H   12.33544247609789      6.30381693145875      5.30076064179024
  H   10.91607581614400      7.33526911261458      5.51844615316304
  H   11.47425532139985      6.33634310723320      6.86921146677122
  H   5.26397760694203      6.30909467575481      3.66830975655473
  H   6.67727846847866      7.34784586701575      3.44342777092291
  H   6.10255238012221      6.36576299856289      2.08837591150228
  H   6.00583680330389      4.44082258822872     10.19094120081794
  H   5.06486932009791      3.00324146288850      9.73020527358439
  H   6.63959432040576      2.81475304627512     10.53622615875970
  H   11.35910109460565      2.57815071259645     -1.47394887610932
  H   11.35057929391091      4.35696207613564     -1.47792221180824
  H   12.70848281809130      3.47567458160120     -0.74026568138931
```

| Nucleus | Element | Isotropic | Anisotropy |
|---|---|---|---|
| 0 | C | 59.435 | 30.460 |
| 1 | C | 9.773 | -124.086 |
| 2 | O | 114.009 | 407.151 |
| 3 | Rh | -6936.292 | -6450.394 |
| 4 | N | 34.797 | 402.346 |
| 5 | C | 65.878 | 329.565 |
| 6 | C | 185.774 | 26.633 |
| 7 | O | 120.555 | 392.002 |
| 8 | Rh | -6943.071 | -6476.891 |
| 9 | N | 34.927 | 401.682 |
| 10 | C | 65.754 | 329.473 |
| 11 | C | 185.823 | 26.715 |
| 12 | O | 147.149 | 370.649 |
| 13 | C | -10.808 | -78.704 |

| | | | |
|---|---|---|---|
| 14 | O | 150.391 | 371.063 |
| 15 | O | 132.778 | 358.566 |
| 16 | C | -10.407 | -76.170 |
| 17 | C | 161.316 | 49.323 |
| 18 | O | 140.250 | 361.755 |
| 19 | C | -10.334 | -81.325 |
| 20 | C | 161.478 | 48.386 |
| 21 | C | 161.790 | 48.113 |
| 22 | O | 131.770 | 358.907 |
| 23 | O | 141.049 | 361.159 |
| 24 | F | 265.902 | 115.324 |
| 25 | F | 254.426 | 137.035 |
| 26 | F | 260.514 | 128.369 |
| 27 | H | 29.905 | 6.337 |
| 28 | H | 29.746 | 6.134 |
| 29 | H | 29.913 | 6.251 |
| 30 | H | 29.947 | 6.248 |
| 31 | H | 29.826 | 6.274 |
| 32 | H | 29.973 | 6.302 |
| 33 | H | 29.872 | 6.397 |
| 34 | H | 29.642 | 5.590 |
| 35 | H | 29.911 | 6.423 |
| 36 | H | 29.284 | 9.936 |
| 37 | H | 29.267 | 9.861 |
| 38 | H | 29.291 | 9.936 |
| 39 | H | 29.299 | 9.843 |
| 40 | H | 29.301 | 9.924 |
| 41 | H | 29.303 | 9.947 |

**3**

```
42
Coordinates from ORCA-job opt
  C   6.21311535699713      0.49150706437989      3.07736392833844
  F   4.94929498795931      0.94684631994461      3.13821016003176
  C   7.19941996192897      1.58026701717868      3.57262653922046
  O   7.01458062758213      1.93924725787831      4.75781869958122
  Rh  8.27169363796692      3.40661421730663      5.52929997736494
  O   9.62751533726001      1.94399308479864      6.11538775649576
  C   10.49079626898363      1.58993294191471      5.28389383176310
  O   10.66897323400175      1.98149241973383      4.10806955333875
  Rh  9.38469067544945      3.44378523974890      3.37174010192053
  O   8.04578156450362      4.87478753945799      2.80364296221695
  C   7.15527377477847      5.26424357252465      3.61786807842894
  C   6.16479779545686      6.27771632913730      3.12359799908728
  F   6.45782669057406      0.12071083265449      1.81775147748396
  F   6.29351865901197     -0.60137438011059      3.85570886433424
  O   8.05316406898603      1.97364720049177      2.74901982985286
  N   10.40734089033846      3.47919560662909      1.38857126700983
  C   10.93081046142117      3.49630417499845      0.36777087223524
  C   11.59015012352058      3.51791982597631     -0.92162115063795
  O   10.60982829626047      4.88877318132797      4.12862096693017
  C   10.45600131387503      5.28563860817123      5.32289208349455
  C   11.35775580823869      6.38425962188850      5.80502373786680
  N   7.24795173491807      3.38789555482656      7.51164113504656
  C   6.72080100457128      3.40301464158371      8.53055159571002
  C   6.05496852060640      3.42398989951843      9.81658354568629
  O   9.58705556996772      4.84435709950321      6.13394668338004
  O   7.01942347005361      4.84699928385765      4.80730109857630
  C   11.49789087520104      0.50869093721757      5.75307488193350
  F   11.28046227770126      0.12608995456972      7.01421497750135
  F   11.41307433180561     -0.57948843157277      4.96800103517209
  F   12.75557381739463      0.97646500714986      5.67199993534804
  H   12.32801109781935      6.32016336871702      5.31662453682108
  H   10.90154457087441      7.34135863159952      5.53968572464581
  H   11.46773410962313      6.34113470110024      6.88635178863962
  H   5.78170537618714      6.87120511351830      3.95088637549202
  H   6.61919274250711      6.91691276617310      2.36941260210860
  H   5.32948329093112      5.74277004057666      2.66462637192407
  H   6.34597095079684      4.32084254136959     10.36457627749967
  H   4.97449366927403      3.42584805583954      9.66890223420484
  H   6.33935370922805      2.54189802750137     10.39121538658855
  H   10.89043369094721      3.20035706942364     -1.69546514096817
  H   11.93457802805880      4.52941579794059     -1.13961382240047
  H   12.44493762643797      2.84091126355497     -0.90765775926789
```

| Nucleus | Element | Isotropic | Anisotropy |
|---|---|---|---|
| 0 | C | 59.365 | 29.954 |
| 1 | F | 256.068 | 134.898 |
| 2 | C | 9.078 | -121.509 |
| 3 | O | 124.591 | 384.692 |
| 4 | Rh | -7009.530 | -6504.688 |
| 5 | O | 122.212 | 417.996 |
| 6 | C | 8.865 | -124.189 |
| 7 | O | 133.864 | 399.052 |
| 8 | Rh | -7014.649 | -6502.319 |
| 9 | O | 143.183 | 350.728 |
| 10 | C | -12.050 | -78.596 |
| 11 | C | 161.046 | 49.574 |
| 12 | F | 266.911 | 113.051 |
| 13 | F | 257.345 | 132.474 |

| | | | |
|---|---|---|---|
| 14 | O | 114.109 | 406.929 |
| 15 | N | 36.657 | 399.109 |
| 16 | C | 65.194 | 330.130 |
| 17 | C | 185.847 | 26.836 |
| 18 | O | 154.280 | 367.955 |
| 19 | C | -11.749 | -66.007 |
| 20 | C | 161.568 | 48.211 |
| 21 | N | 36.787 | 398.601 |
| 22 | C | 65.126 | 329.998 |
| 23 | C | 185.810 | 26.678 |
| 24 | O | 151.309 | 366.309 |
| 25 | O | 141.403 | 352.216 |
| 26 | C | 59.328 | 29.241 |
| 27 | F | 264.770 | 113.259 |
| 28 | F | 255.346 | 135.959 |
| 29 | F | 256.047 | 134.748 |
| 30 | H | 29.905 | 6.126 |
| 31 | H | 29.792 | 6.516 |
| 32 | H | 29.929 | 6.187 |
| 33 | H | 29.809 | 6.348 |
| 34 | H | 29.795 | 6.355 |
| 35 | H | 29.596 | 5.958 |
| 36 | H | 29.260 | 9.997 |
| 37 | H | 29.247 | 9.866 |
| 38 | H | 29.266 | 9.892 |
| 39 | H | 29.261 | 9.891 |
| 40 | H | 29.283 | 10.012 |
| 41 | H | 29.287 | 9.896 |

**4**

```
42
Coordinates from ORCA-job opt
  C   6.19946128968192      0.49276879181924      3.08545570397375
  F   4.93809143679272      0.95339896511011      3.14981375716530
  C   7.19058926887217      1.57898272809698      3.57558965194280
  O   7.01849429407795      1.93409945788812      4.76373156142821
  Rh  8.27030796406387      3.39093510733262      5.52074100724221
  O   9.61729548329561      1.94355756141984      6.07777660962535
  C   10.50180532097493      1.55713931885994      5.25548291269168
  O   10.63306024340911      1.98067313741081      4.06755665476272
  Rh  9.36568541446140      3.43231739306224      3.35449344411350
  O   8.01869192508805      4.87992144720387      2.79740340881813
  C   7.14358935600667      5.27715939939499      3.62474832985605
  C   6.21954673335342      6.37369427680475      3.17875518474412
  F   6.43992202301838      0.12038059896205      1.82584862490243
  F   6.27886558686158     -0.59857302380187      3.86523003664816
  O   8.03520751282137      1.97788066499101      2.74514197431263
  N   10.37244124670656      3.44833846298229      1.36253992373623
  C   10.89192336119160      3.40684137122110      0.34041946285586
  C   11.54751463609808      3.35122476987223     -0.94983697314554
  O   10.61886248886474      4.88922191293522      4.10905885000332
  C   10.44267465882288      5.25056895533303      5.29336086911832
  C   11.36535608789424      6.39059336712828      5.79521392150705
  N   7.26076140478892      3.36295384166938      7.50913815043861
  C   6.73428039627232      3.36555813174794      8.52849499913740
  C   6.06909231672141      3.37037163510742      9.81501319068295
  O   9.60080164040153      4.84761290981041      6.12678559148384
  O   7.00148476249762      4.84105735306818      4.80679553187316
  C   11.49146425628919      0.53584001478069      5.73798979993295
  H   11.03599584726002     -0.11205192056767      6.48417883950944
  H   11.87477892376061     -0.04832951668544      4.90421233527547
  H   12.32700337499063      1.06376280284970      6.20465397254968
  F   12.49749237506533      6.46489437439585      5.08855387766070
  F   10.72424579456340      7.56994526223605      5.67567291718040
  F   11.68795686467315      6.22770140172898      7.08458051992201
  H   5.26093971539996      6.29396426487099      3.68717632647282
  H   6.67304382075010      7.33156137148401      3.44609601810680
  H   6.08608521670910      6.34502369623056      2.09944833514679
  H   6.36852581687048      4.25482269834178     10.37837879842771
  H   4.98864239165268      3.38484407341535      9.66780851570797
  H   6.34548178278375      2.47582210500658     10.37409195635193
  H   11.23413647891477      2.45114740296813     -1.48001520123778
  H   11.27656540560944      4.22891511395127     -1.53748979546813
  H   12.62880708166787      3.32979231956276     -0.80997259545722
```

| Nucleus | Element | Isotropic | Anisotropy |
|---|---|---|---|
| 0 | C | 59.475 | 30.182 |
| 1 | F | 255.857 | 134.672 |
| 2 | C | 8.608 | -122.484 |
| 3 | O | 130.946 | 385.146 |
| 4 | Rh | -7037.213 | -6552.894 |
| 5 | O | 144.762 | 359.118 |
| 6 | C | -11.320 | -69.051 |
| 7 | O | 144.647 | 356.667 |
| 8 | Rh | -7021.844 | -6515.008 |
| 9 | O | 134.703 | 355.751 |
| 10 | C | -11.345 | -69.238 |
| 11 | C | 161.127 | 49.586 |
| 12 | F | 266.922 | 112.904 |
| 13 | F | 257.210 | 131.998 |

| | | | |
|---|---|---|---|
| 14 | O | 120.975 | 406.706 |
| 15 | N | 36.641 | 399.240 |
| 16 | C | 65.144 | 330.163 |
| 17 | C | 185.802 | 26.884 |
| 18 | O | 134.540 | 420.675 |
| 19 | C | 8.404 | -123.955 |
| 20 | C | 59.332 | 29.502 |
| 21 | N | 36.822 | 398.260 |
| 22 | C | 65.077 | 330.022 |
| 23 | C | 185.801 | 26.665 |
| 24 | O | 136.678 | 412.702 |
| 25 | O | 135.069 | 355.574 |
| 26 | C | 161.276 | 48.679 |
| 27 | H | 29.860 | 6.461 |
| 28 | H | 29.884 | 6.435 |
| 29 | H | 29.687 | 5.788 |
| 30 | F | 263.519 | 117.737 |
| 31 | F | 251.908 | 139.985 |
| 32 | F | 260.916 | 124.205 |
| 33 | H | 29.807 | 6.501 |
| 34 | H | 29.571 | 5.377 |
| 35 | H | 29.843 | 6.510 |
| 36 | H | 29.259 | 9.929 |
| 37 | H | 29.239 | 9.869 |
| 38 | H | 29.259 | 9.937 |
| 39 | H | 29.266 | 9.876 |
| 40 | H | 29.280 | 9.946 |
| 41 | H | 29.289 | 9.936 |

**5**

```
42
Coordinates from ORCA-job opt
  C   6.07259848051764      0.55272955744823      3.18048053532478
  C   7.13233878762312      1.58263535686151      3.64887450044337
  O   7.96686218355761      1.95115059979106      2.79380572956546
  Rh  9.34141137116104      3.38892391327849      3.33881050077651
  N   10.32354763714809      3.40310882598550      1.34227147043749
  C   10.91565666348624      3.42782211597545      0.36015246784391
  C   11.66729954863829      3.46097022557912     -0.87709989642356
  O   7.01185943285725      1.93832439151798      4.84407701231980
  Rh  8.30860126768112      3.38127373502854      5.54665494083411
  N   7.33178880590099      3.37461565138477      7.54224355339896
  C   6.74663753122907      3.37161116929510      8.52877579823483
  C   6.00496885539027      3.36853329911875      9.77235963406133
  O   9.67923533794870      4.82304546568788      6.08682664428209
  C   10.51178750352861      5.19588943508004      5.22983738331050
  O   10.64126465242349      4.82988145066969      4.04004855802638
  O   6.99474337037250      4.83574995738420      4.84864673539670
  C   7.12368789672151      5.20636933445121      3.66030310668951
  C   6.13539289050234      6.30243193862054      3.18422482714581
  O   9.66442042835993      1.95226483393702      6.06828706895765
  C   10.52749824983731      1.55579782206367      5.22712621400456
  C   11.53928145724893      0.55605717460584      5.70255103599779
  C   11.48956546284342      6.30081058964306      5.70522616777836
  O   7.95939020105562      4.84316006972331      2.80205064278212
  O   10.61867058551595      1.95626791708831      4.02676476573676
  F   6.25719039932303      0.17999903559292      1.91211431805983
  F   6.11313051693170     -0.54619617173744      3.95081175942133
  F   4.84221910420298      1.08397760280086      3.28508315771123
  H   11.09686650239859     -0.10593153975273      6.44432636615864
  H   11.93679425677030     -0.01333476641924      4.86562292706346
  H   12.35944050899975      1.10221231587094      6.17547603815635
  F   12.62722588111399      6.29495121753521      5.00325801351319
  F   10.90850031590254      7.50514051162208      5.54580223962816
  F   11.79579408958325      6.15870090527989      7.00007969205100
  F   5.00123200602676      6.29431567609548      3.89216151340283
  F   6.70825201419784      7.51272864898291      3.33177305802066
  F   5.82184752843805      6.15011071782130      1.89169043989894
  H   5.94563101674682      4.38401828595326     10.16541468655870
  H   4.99731558492180      2.99095625334433      9.59449129041813
  H   6.50825197327398      2.72851386579963     10.49769963387716
  H   11.61645837113823      2.48650510952911     -1.36373181570466
  H   11.24855650222053      4.21879326545441     -1.54011728564656
  H   12.70892482625957      3.70383823600740     -0.66362942951423
```

| Nucleus | Element | Isotropic | Anisotropy |
|---|---|---|---|
| ------- | ------- | ------------ | ------------ |
| 0 | C | 59.451 | 29.775 |
| 1 | C | 7.717 | -121.226 |
| 2 | O | 125.062 | 406.081 |
| 3 | Rh | -7129.952 | -6541.728 |
| 4 | N | 38.432 | 396.357 |
| 5 | C | 64.413 | 330.527 |
| 6 | C | 185.900 | 27.045 |
| 7 | O | 134.987 | 382.541 |
| 8 | Rh | -7139.391 | -6584.411 |
| 9 | N | 38.606 | 394.966 |
| 10 | C | 64.273 | 330.432 |
| 11 | C | 185.762 | 26.960 |
| 12 | O | 139.073 | 412.381 |
| 13 | C | 7.504 | -121.798 |

| | | | |
|---|---|---|---|
| 14 | O | 137.738 | 411.984 |
| 15 | O | 122.205 | 401.633 |
| 16 | C | 8.303 | -121.243 |
| 17 | C | 59.446 | 30.389 |
| 18 | O | 156.838 | 356.524 |
| 19 | C | -13.060 | -68.126 |
| 20 | C | 160.878 | 49.088 |
| 21 | C | 59.298 | 29.708 |
| 22 | O | 125.719 | 399.901 |
| 23 | O | 155.104 | 351.174 |
| 24 | F | 266.998 | 112.350 |
| 25 | F | 257.446 | 131.303 |
| 26 | F | 254.848 | 134.267 |
| 27 | H | 29.775 | 6.529 |
| 28 | H | 29.826 | 6.479 |
| 29 | H | 29.664 | 5.754 |
| 30 | F | 263.157 | 119.155 |
| 31 | F | 251.680 | 137.676 |
| 32 | F | 260.798 | 123.786 |
| 33 | F | 264.405 | 119.191 |
| 34 | F | 254.190 | 136.395 |
| 35 | F | 261.897 | 122.907 |
| 36 | H | 29.231 | 9.822 |
| 37 | H | 29.208 | 9.828 |
| 38 | H | 29.237 | 10.039 |
| 39 | H | 29.268 | 10.040 |
| 40 | H | 29.259 | 9.908 |
| 41 | H | 29.261 | 9.866 |

**6**

```
42
Coordinates from ORCA-job opt
  C   6.20925956545915      0.48651951931657      3.09720387864367
  C   7.19466118682694      1.57973842432852      3.58490428032045
  O   8.04490852275780      1.97414003367083      2.75676732165051
  Rh  9.37132203912246      3.43047774114679      3.35651178968425
  N   10.38036209255456      3.44097597922758      1.38389782985822
  C   10.89909335469220      3.42063453668310      0.36139056845445
  C   11.55372821561503      3.39360039572169     -0.92938746471185
  O   7.01534286330366      1.94514166885201      4.76987821597610
  Rh  8.25560276028155      3.40543504171350      5.52878243654040
  N   7.24758747585667      3.38113043081654      7.50158068948624
  C   6.73143154452879      3.36278349034332      8.52541660425509
  C   6.08008108470511      3.33928227752154      9.81790824949714
  O   9.58236320824984      4.86405072922680      6.12452944320588
  C   10.43019245193934      5.26196289023099      5.29323768922751
  O   10.61346071849625      4.89020909574000      4.11235811103940
  O   6.98944541481240      4.85553327610633      4.79361316341059
  C   7.16749983435459      5.24843300527407      3.61895256431865
  C   6.23049908866826      6.38422637365160      3.13299464422384
  O   9.60406747518222      1.96072002731259      6.10738798656332
  C   10.46221445825145      1.59428412282512      5.27454648742898
  C   11.46565772919785      0.51073250952792      5.74665387876953
  C   11.34893543145377      6.40725790748415      5.79158070411492
  O   8.02275224858658      4.87780684787042      2.78246718180625
  O   10.63591475033997      1.98030003950873      4.09531179202647
  F   6.45619720597593      0.11341826685468      1.84032973524419
  F   6.29583349274152     -0.59903216521491      3.88222554164681
  F   4.94776960581067      0.94310504178242      3.15906922410493
  F   11.23880827952886      0.13128950319516      7.00543625845688
  F   11.37991287159554     -0.57350638929721      4.95935535218756
  F   12.72126582528954      0.98033807090939      5.66996906035697
  F   12.48193576571487      6.47592028576855      5.08810038938939
  F   10.70418618940481      7.58178564817101      5.65993182352336
  F   11.66397183412649      6.25082567067989      7.08235007087144
  F   5.11724754047394      6.45883793778705      3.86626787881982
  F   6.87427349103550      7.56246419140846      3.22994508750240
  F   5.88034808905759      6.20846228500667      1.85330746254088
  H   6.36788326459391      4.22312530941787     10.38821699255786
  H   4.99818482816597      3.33305908260099      9.68119658655161
  H   6.38149983018907      2.44364996677587     10.36226953653314
  H   11.25305353511470      2.49669151927060     -1.47210094127343
  H   11.26811416639429      4.27607231150593     -1.50286213006281
  H   12.63527066954798      3.38684109927602     -0.78986997474221
```

| Nucleus | Element | Isotropic | Anisotropy |
|---|---|---|---|
| 0 | C | 59.455 | 29.780 |
| 1 | C | 7.024 | -118.815 |
| 2 | O | 126.417 | 397.647 |
| 3 | Rh | -7207.440 | -6630.554 |
| 4 | N | 40.521 | 392.273 |
| 5 | C | 63.864 | 331.189 |
| 6 | C | 185.743 | 27.179 |
| 7 | O | 136.362 | 375.886 |
| 8 | Rh | -7204.243 | -6626.351 |
| 9 | N | 40.713 | 391.677 |
| 10 | C | 63.826 | 331.125 |
| 11 | C | 185.810 | 27.045 |

| | | | |
|---|---|---|---|
| 12 | O | 145.604 | 408.207 |
| 13 | C | 6.866 | -120.879 |
| 14 | O | 142.635 | 416.630 |
| 15 | O | 130.274 | 401.465 |
| 16 | C | 6.997 | -118.446 |
| 17 | C | 59.646 | 30.463 |
| 18 | O | 136.839 | 411.197 |
| 19 | C | 6.941 | -121.359 |
| 20 | C | 59.488 | 28.971 |
| 21 | C | 59.296 | 29.078 |
| 22 | O | 134.943 | 393.283 |
| 23 | O | 148.615 | 392.459 |
| 24 | F | 266.648 | 112.126 |
| 25 | F | 256.877 | 131.054 |
| 26 | F | 255.615 | 133.366 |
| 27 | F | 264.035 | 112.166 |
| 28 | F | 254.833 | 134.909 |
| 29 | F | 255.288 | 133.631 |
| 30 | F | 263.352 | 117.185 |
| 31 | F | 250.866 | 137.684 |
| 32 | F | 260.557 | 123.190 |
| 33 | F | 264.944 | 115.359 |
| 34 | F | 253.790 | 136.464 |
| 35 | F | 261.121 | 124.217 |
| 36 | H | 29.215 | 9.920 |
| 37 | H | 29.189 | 9.901 |
| 38 | H | 29.210 | 9.963 |
| 39 | H | 29.215 | 9.919 |
| 40 | H | 29.223 | 9.902 |
| 41 | H | 29.230 | 9.951 |

# 6. NMR Spectra

**Rhodium(III) acetylacetonate ($Rh(acac)_3$): H(C)Rh spectra stacked at variable temperatures (10mM, $CDCl_3$)**

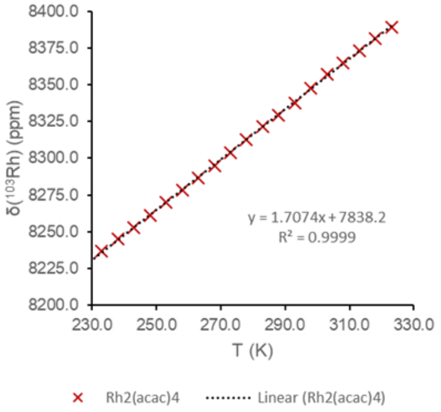


| T(K) | $\delta(^{103}Rh)$ (ppm) |
|---|---|
| 223.0 | 8219.5 |
| 228.0 | 8228.1 |
| 233.0 | 8236.8 |
| 238.0 | 8244.9 |
| 243.0 | 8252.7 |
| 248.0 | 8261.0 |
| 253.0 | 8270.1 |
| 258.0 | 8278.5 |
| 263.0 | 8286.4 |
| 268.0 | 8295.1 |
| 273.0 | 8303.8 |
| 278.0 | 8312.8 |
| 283.0 | 8321.4 |
| 288.0 | 8329.5 |
| 293.0 | 8337.8 |
| 298.0 | 8347.4 |
| 303.0 | 8357.3 |
| 308.0 | 8364.8 |
| 313.0 | 8372.9 |
| 318.0 | 8381.1 |
| 323.0 | 8389.4 |

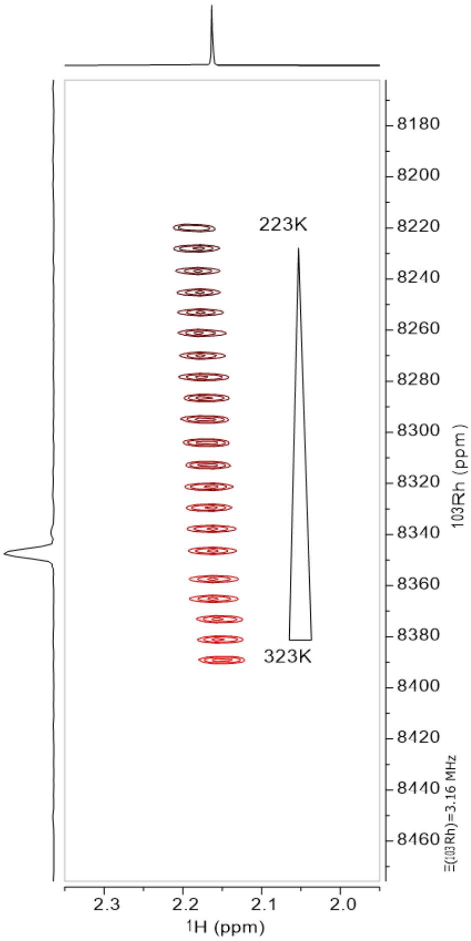

## Dirhodium(II) tetra(acetate) ($Rh_2(OAc)_4$) (1) : $^1H$-NMR (500 MHz, $CD_3CN$)

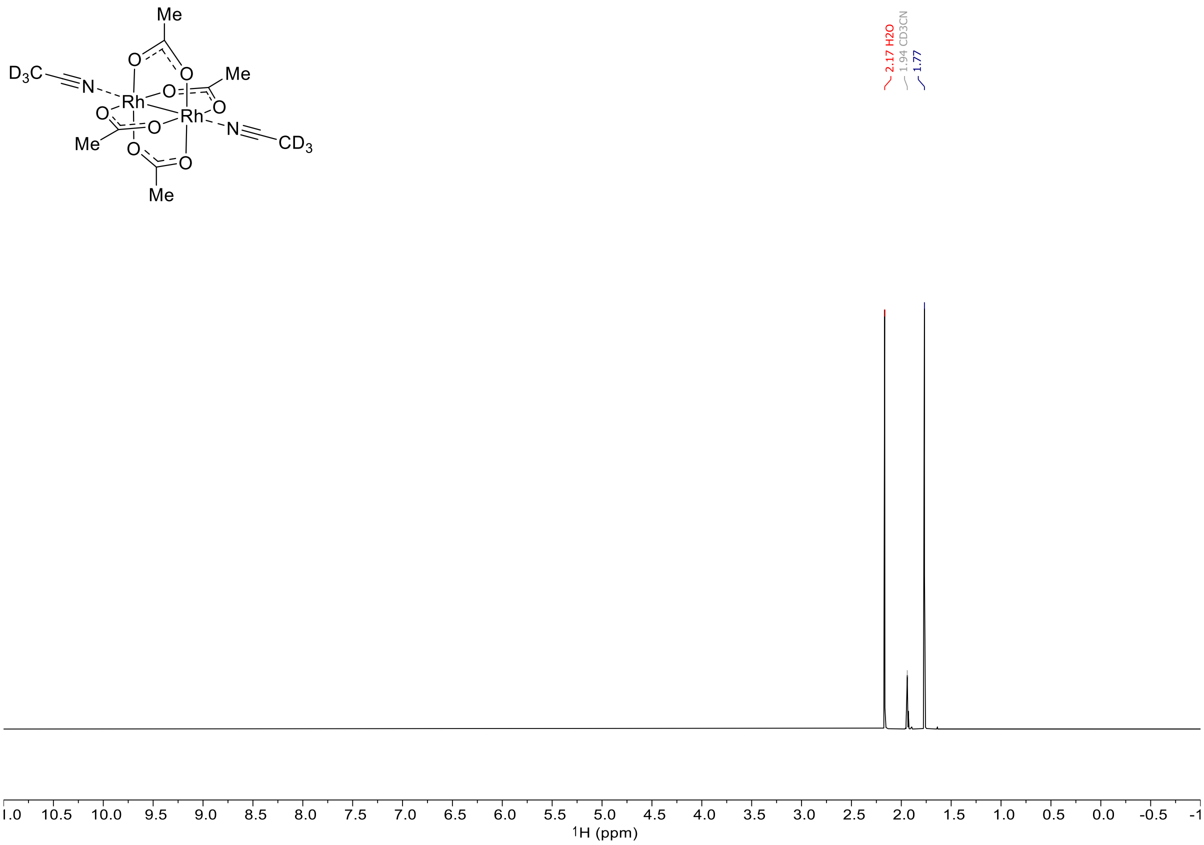


## Dirhodium(II) tetra(acetate) ($Rh_2(OAc)_4$) (1) : H(C)Rh spectrum ($CD_3CN$)

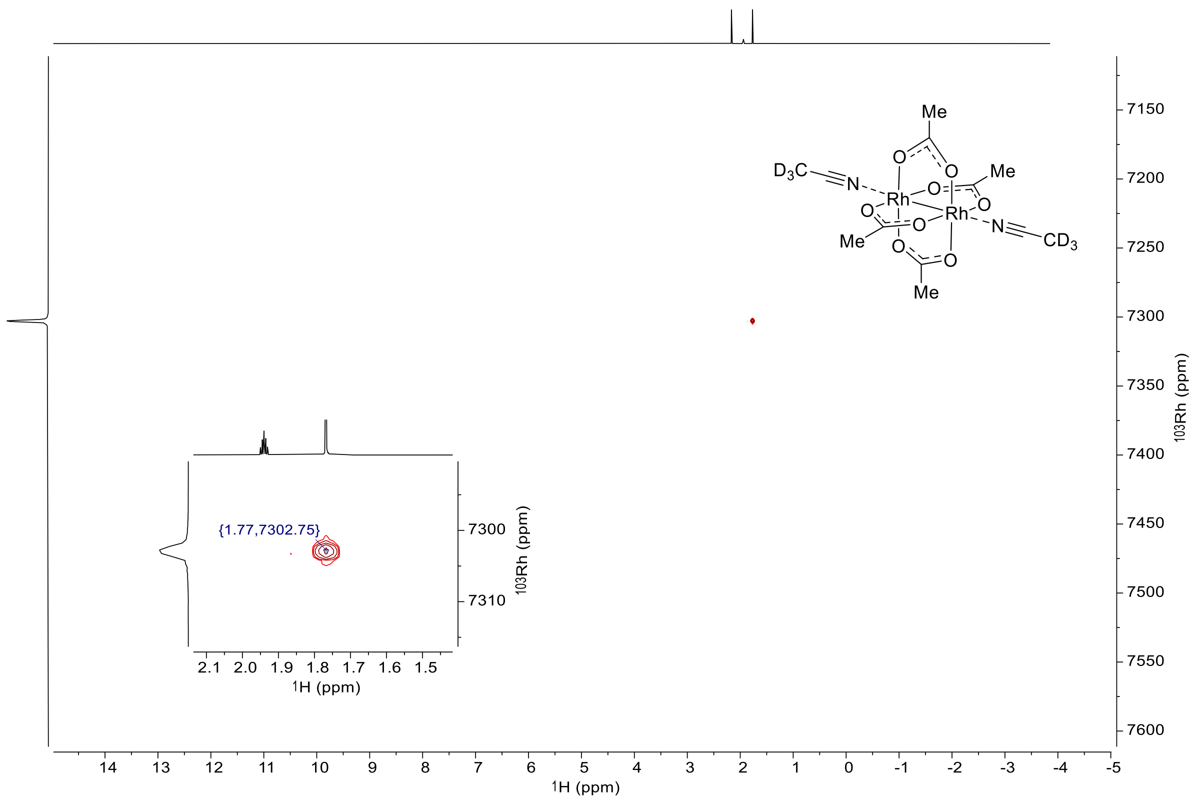

**Dirhodium(II) tetra(acetate) ($Rh_2(OAc)_4$) (1) : H(C)Rh spectra stacked at variable temperatures ($CD_3CN$)**

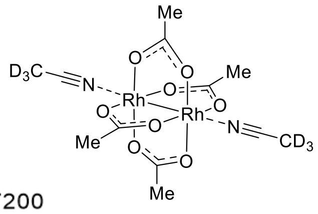


| T(K) | δ($^{103}$Rh) (ppm) |
|---|---|
| 298 | 7303.0 |
| 293 | 7296.5 |
| 283 | 7283.4 |
| 273 | 7269.9 |
| 263 | 7256.1 |
| 253 | 7242.8 |
| 243 | 7229.9 |
| 233 | 7217.3 |

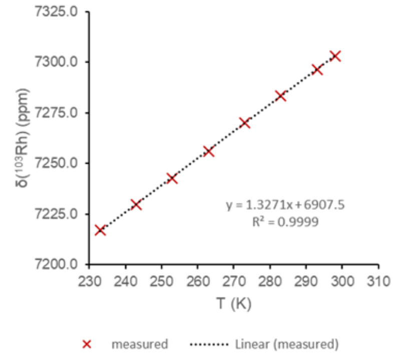


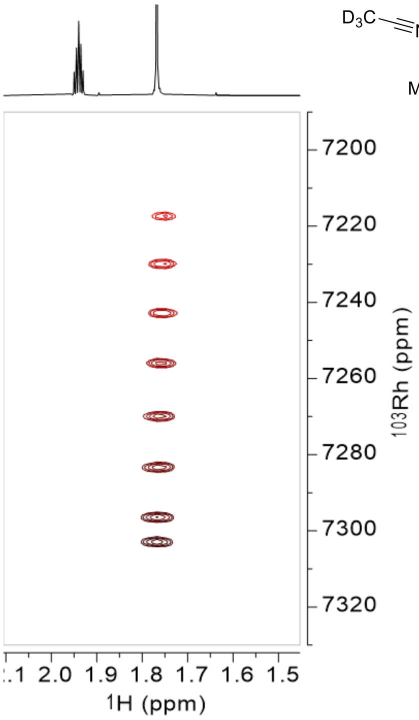

**Dirhodium(II) tetra(acetate) ($Rh_2(OAc)_4$) (1): $^1$H-NMR (500 MHz, [$D_8$]-THF)**

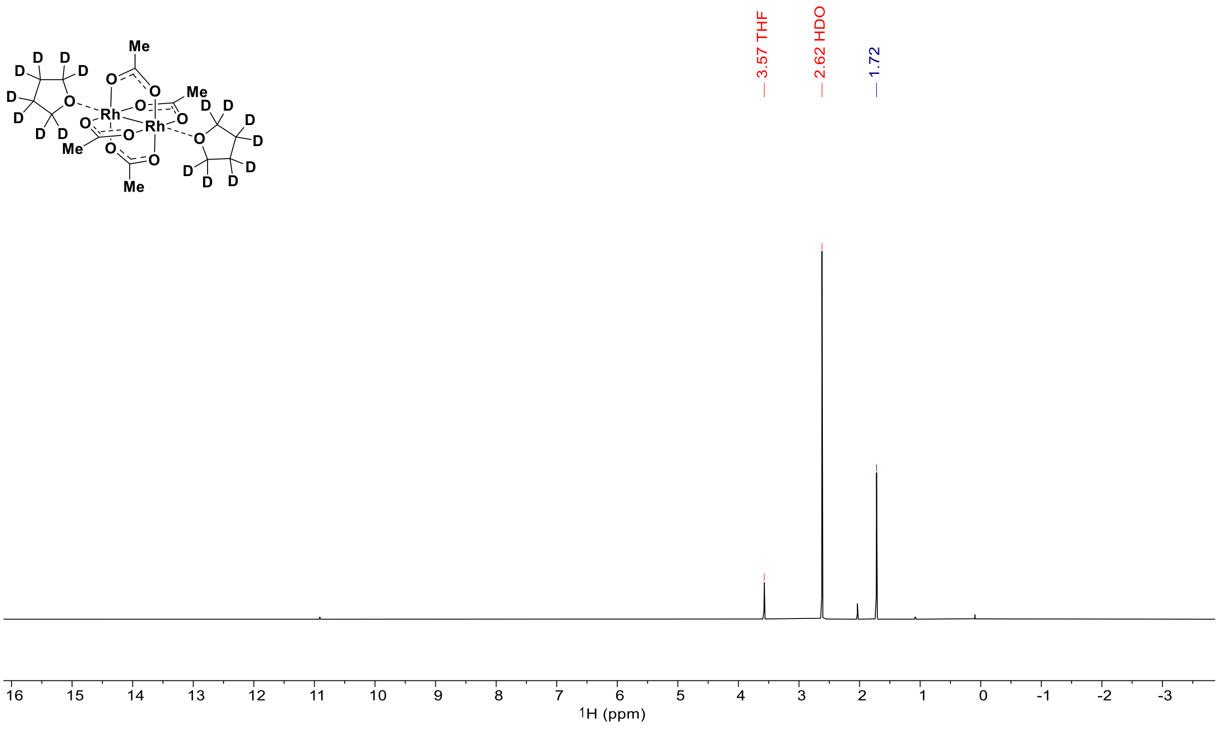


**Dirhodium(II) tetra(acetate) ($Rh_2(OAc)_4$) (1): 2D H(C)Rh spectrum ([$D_8$]-THF)**

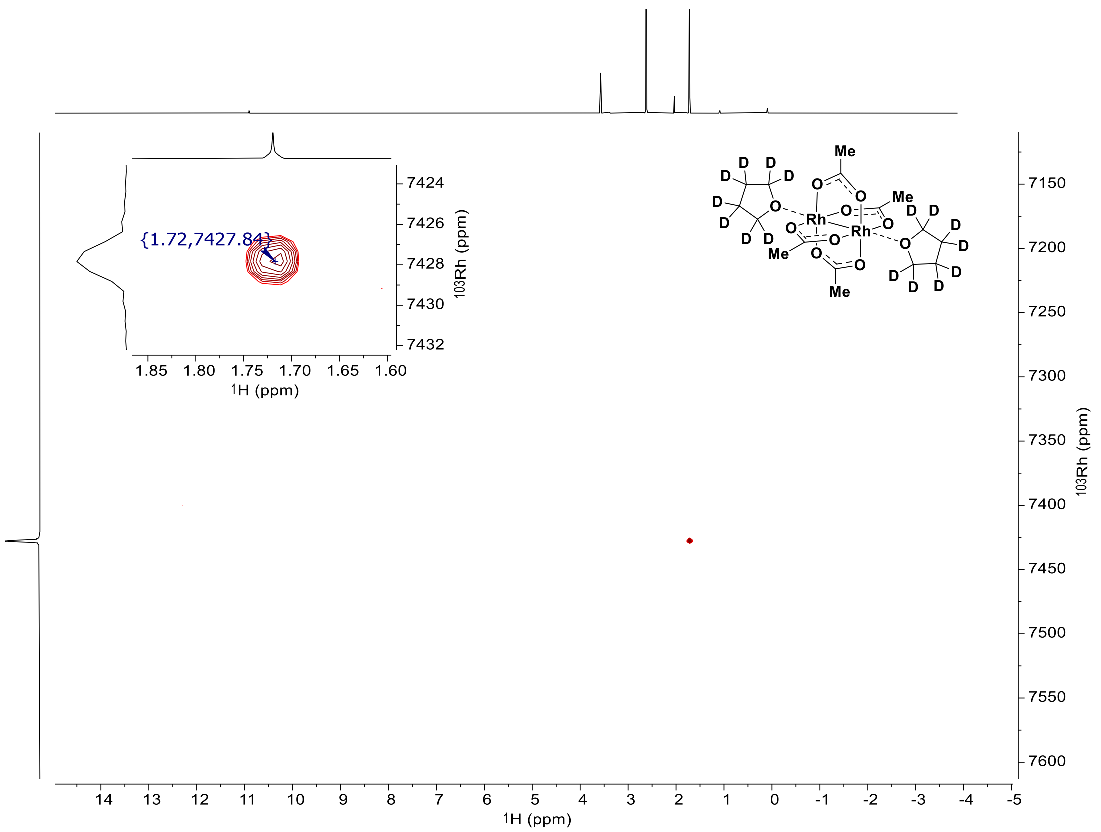

## Dirhodium(II) tetra(acetate) ($Rh_2(OAc)_4$) (1): $^1$H-NMR (500 MHz, [D$_6$]-acetone)

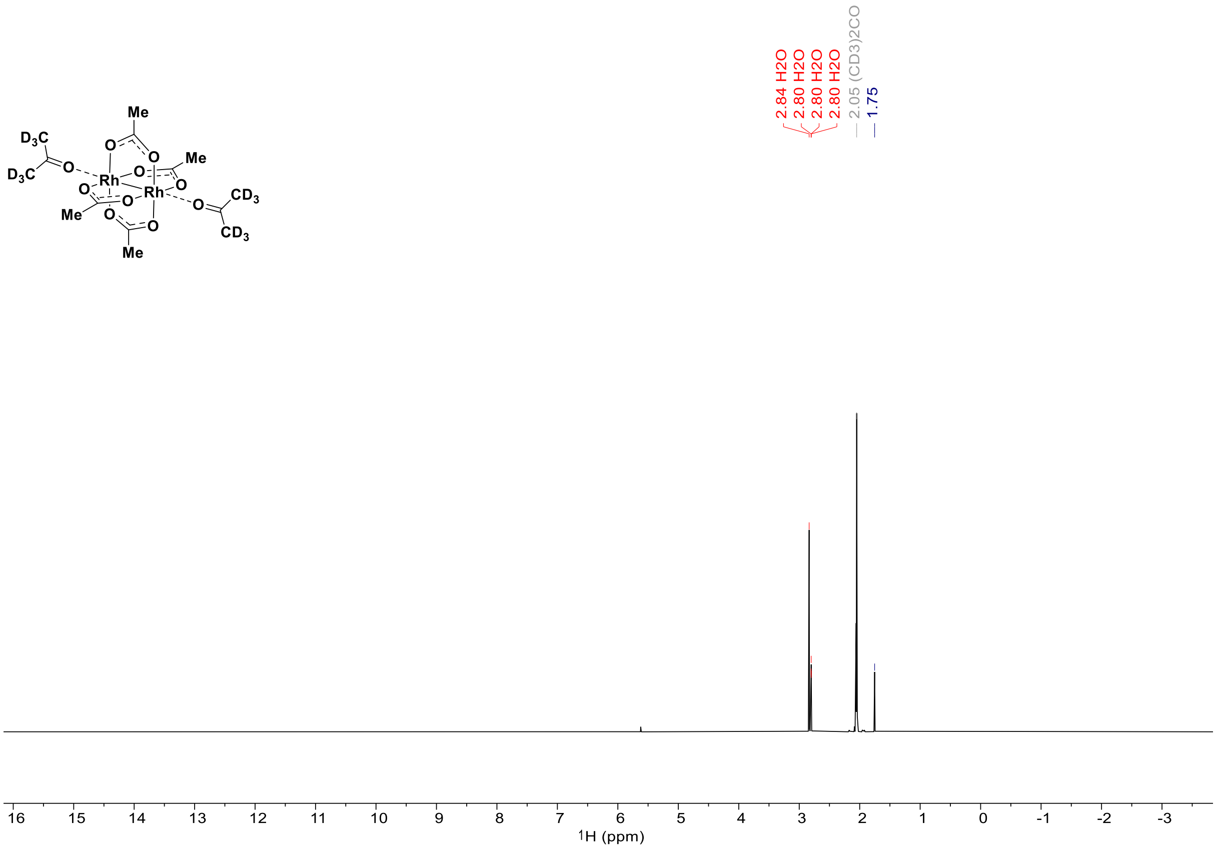


## Dirhodium(II) tetra(acetate) ($Rh_2(OAc)_4$) (1): 2D H(C)Rh spectrum ([D$_6$]-acetone)

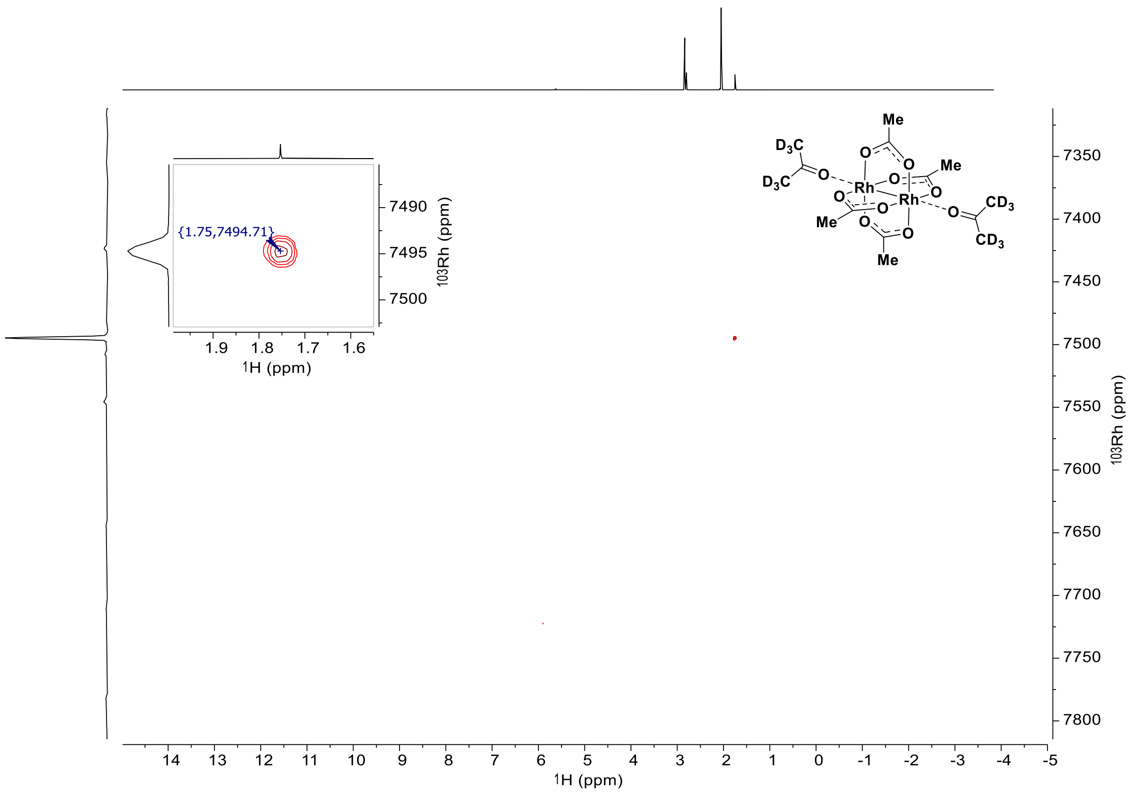

**$[Rh_2(OAc)_4]\cdot PPh_3$ (1·$PPh_3$): $^1$H-NMR (500 MHz, $CD_2Cl_2$)**

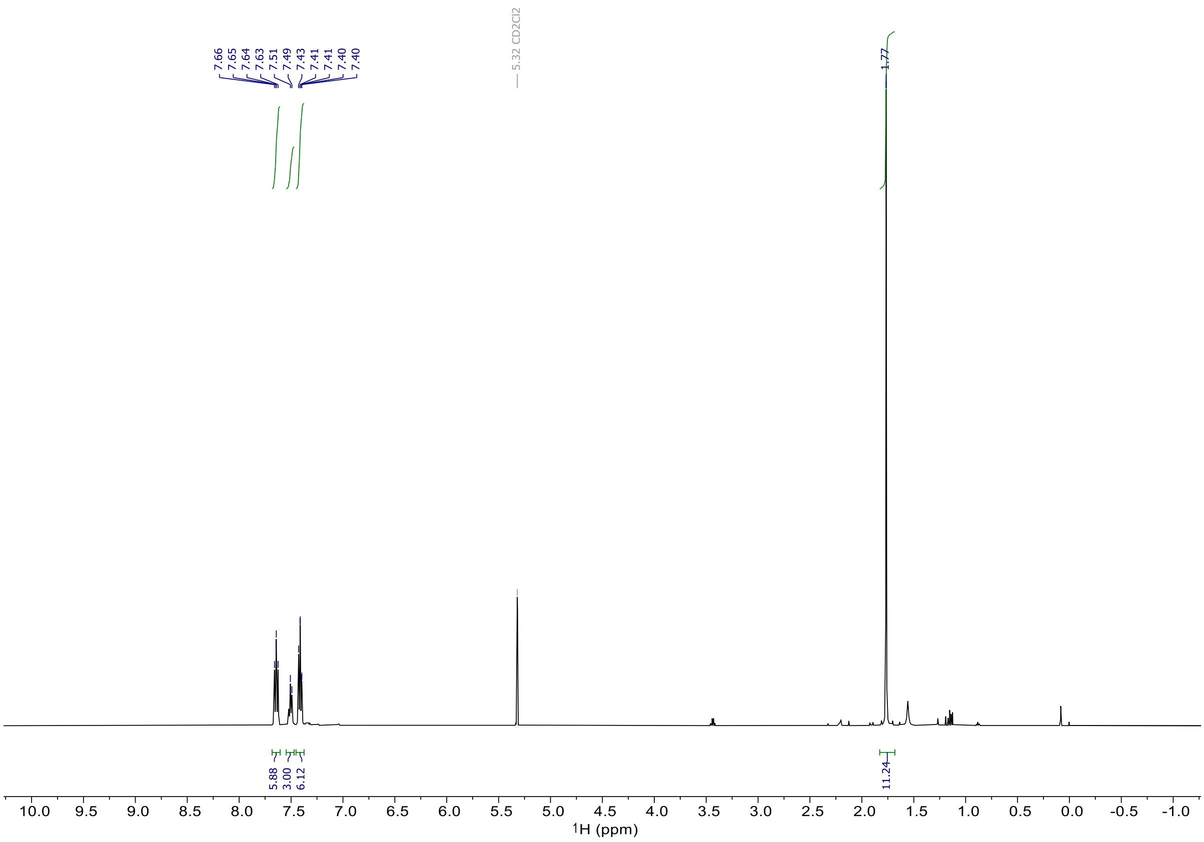


**$[Rh_2(OAc)_4]\cdot PPh_3$ (1·$PPh_3$): $^{31}$P-NMR (202 MHz, $CD_2Cl_2$)**

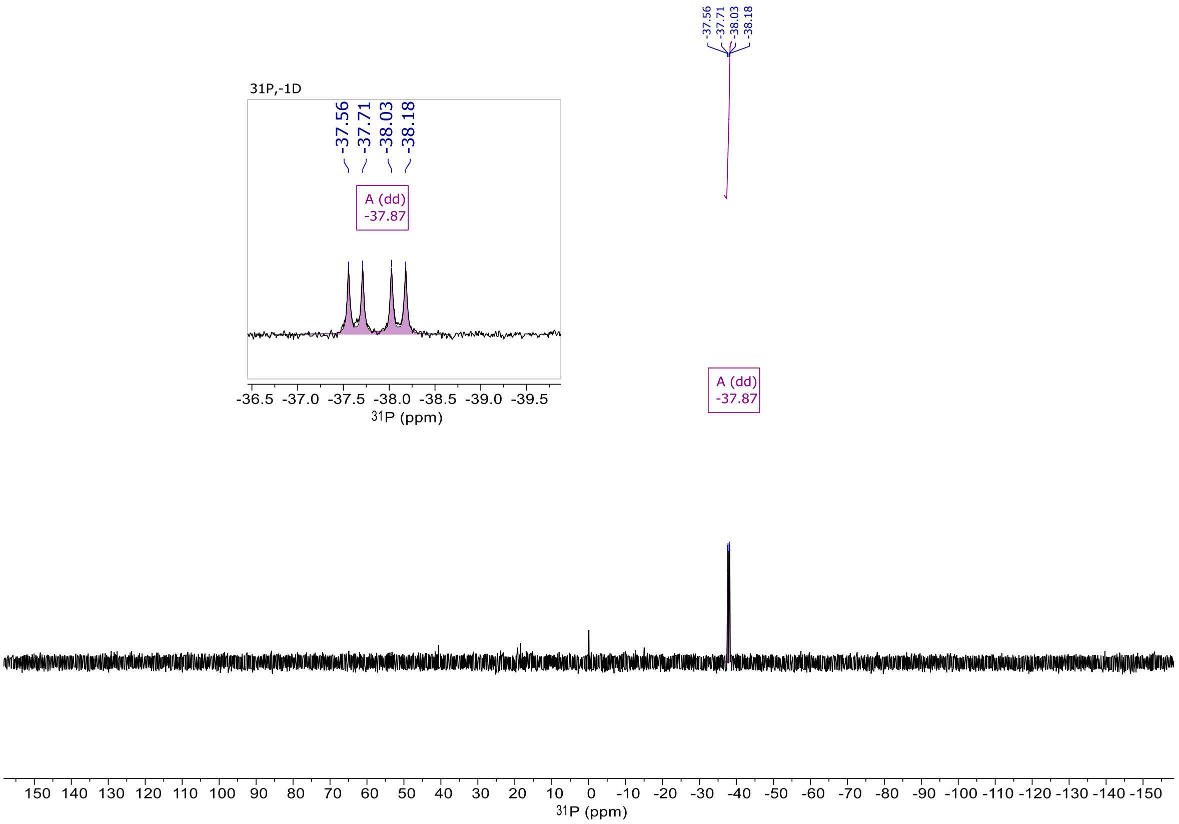

**$[Rh_2(OAc)_4]\cdot PPh_3$ ($1\cdot PPh_3$): 2D H(C)Rh spectrum ($CD_2Cl_2$)**

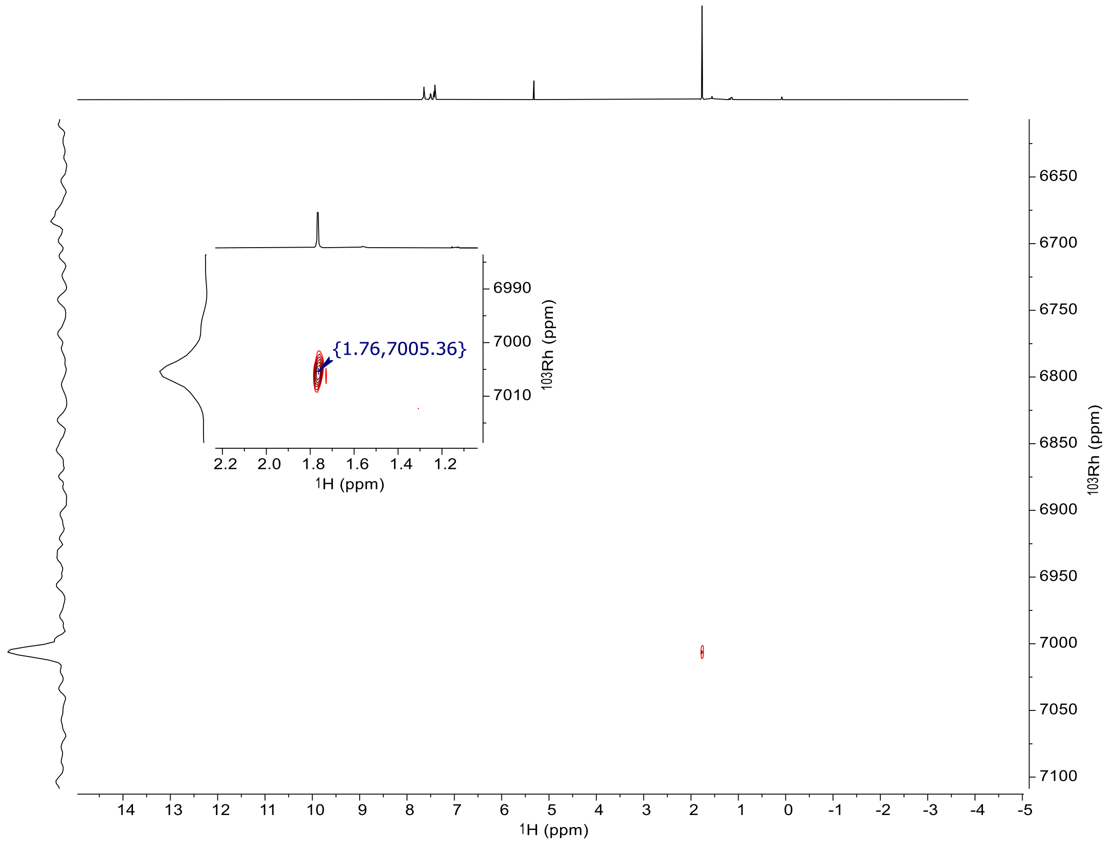

**Dirhodium(II) tetra(acetate-2-$^{13}C$) (2-$^{13}C$-1): $^{1}H$-NMR (400 MHz, $CD_3CN$)**

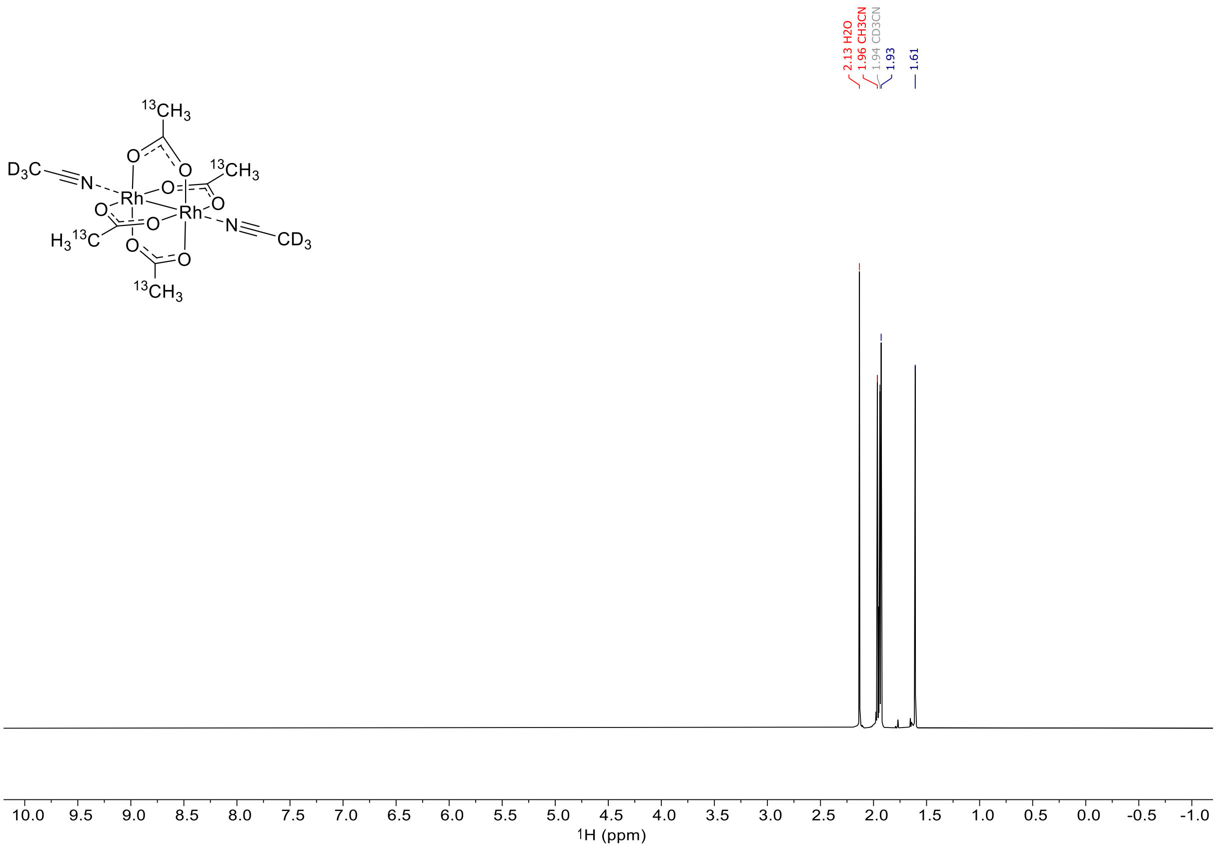


**Dirhodium(II) tetra(acetate-2-$^{13}C$) (2-$^{13}C$-1): $^{13}C\{^{1}H\}$-NMR (101 MHz, $CD_3CN$)**

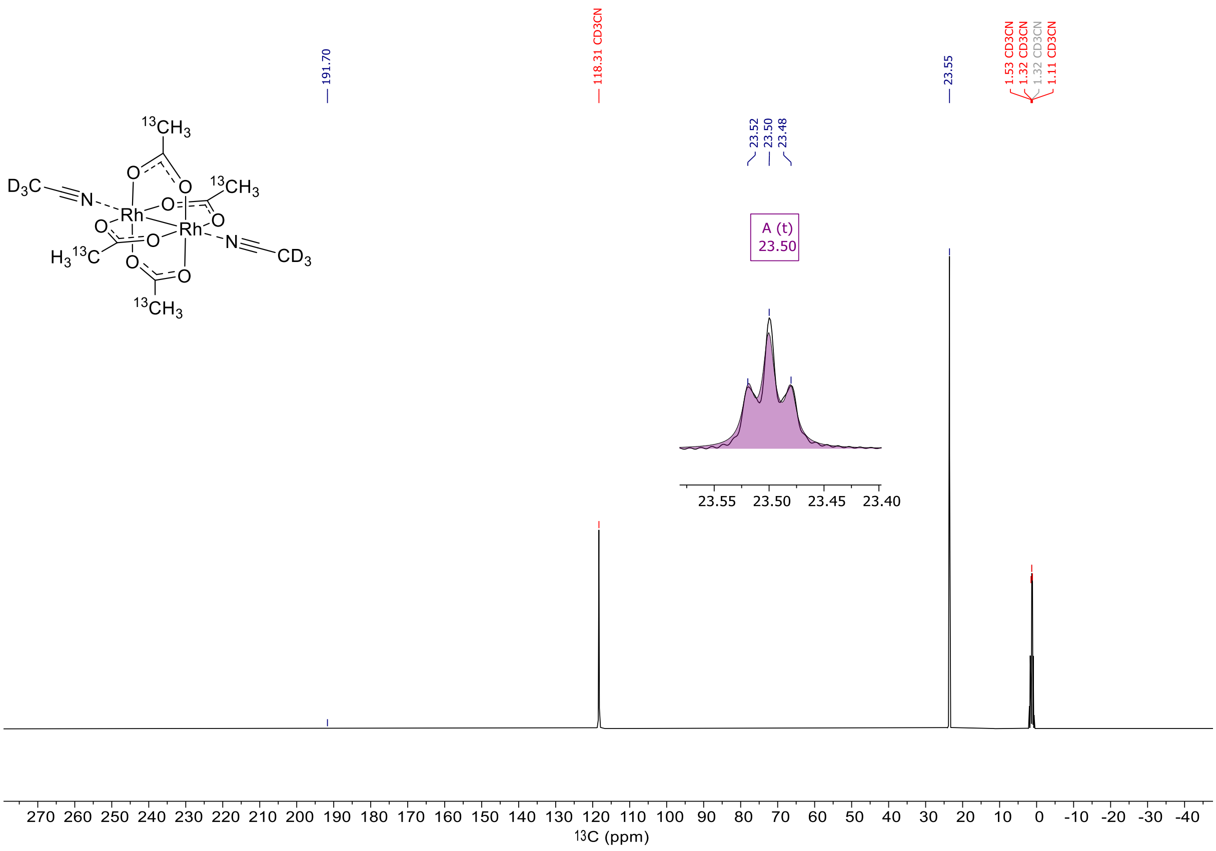

**Dirhodium(II) tetra(acetate-2-$^{13}$C): Left: $^{13}$C{$^{1}$H}-NMR (126 MHz, $CD_3CN$); Right: $^{13}$C{$^{1}$H, $^{103}$Rh}-NMR (126 MHz, $CD_3CN$)**

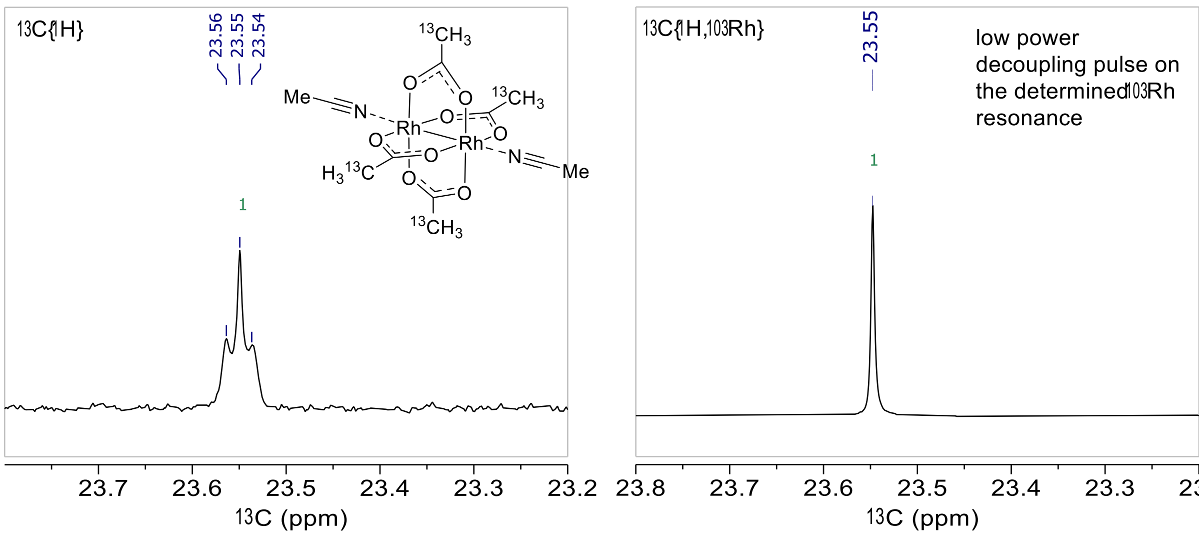


**Dirhodium(II) tetra(acetate-2-$^{13}$C) (2-$^{13}$C-1): $^{13}$C{$^{1}$H}-$^{103}$Rh-HMBC ($CD_3CN$)**

To verify that the $^{103}$Rh NMR signal is not folded, two spectra with the same offset, but a different spectral width in the indirect dimension were measured. By comparing the spectra, the signal appears at almost the same position (within 1 ppm). This confirms that the cross peaks is not folded and the correct $^{103}$Rh resonance was found.

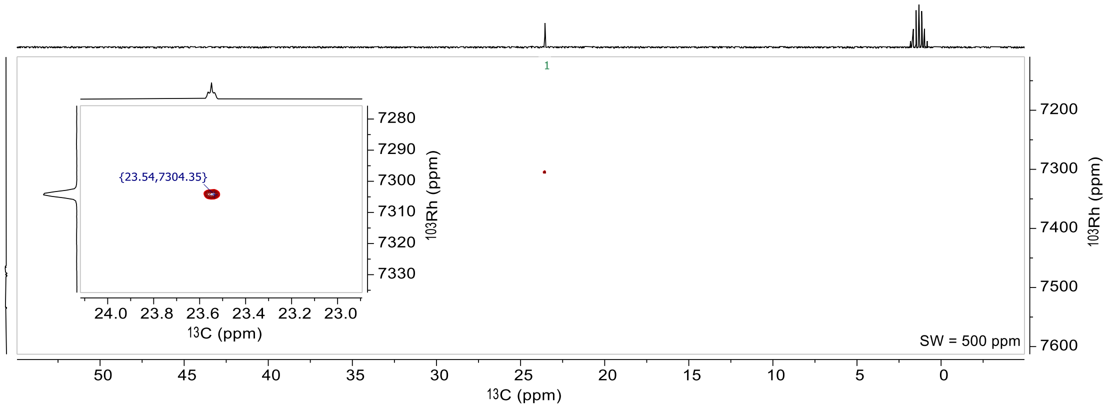


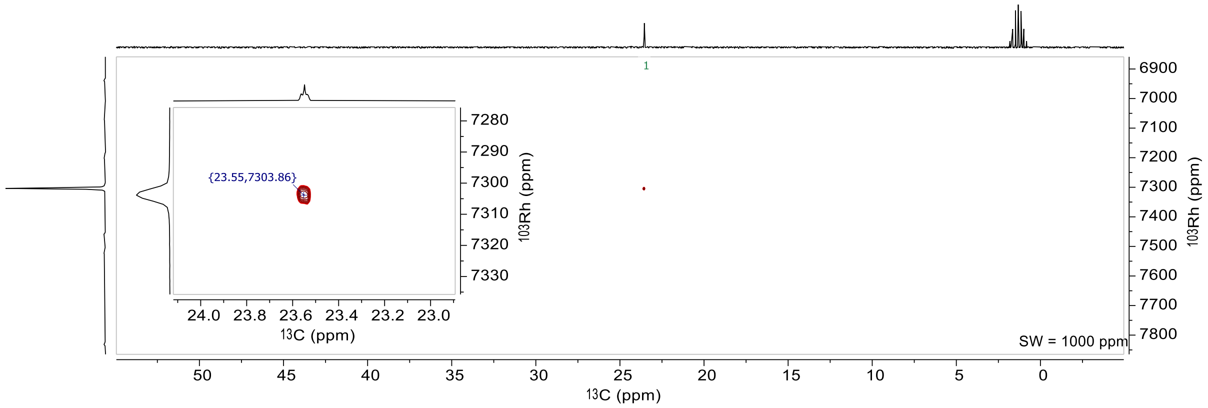

**Dirhodium(II) tetra(acetate-1-$^{13}$C) (1-$^{13}$C-1): $^1$H-NMR (400 MHz, $CD_3CN$)**

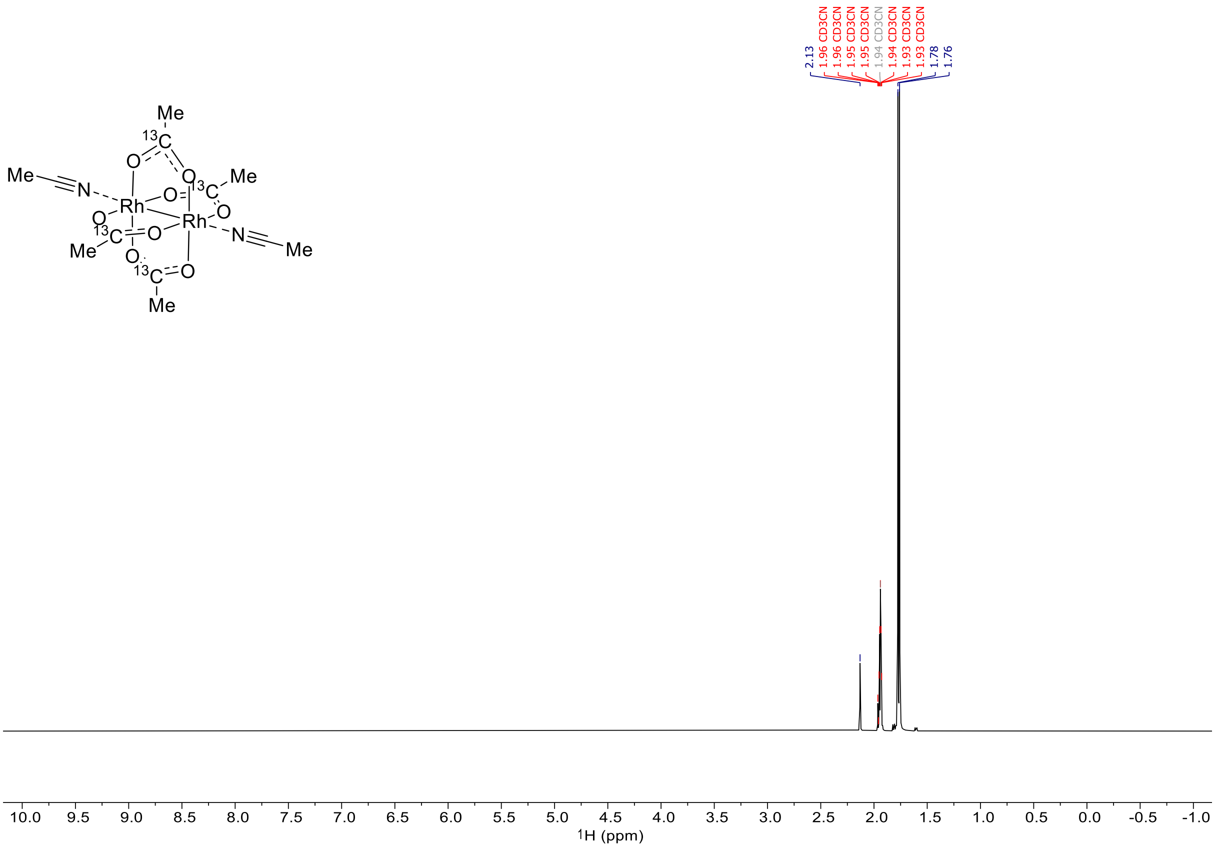


**Dirhodium(II) tetra(acetate-1-$^{13}$C) (1-$^{13}$C-1): $^{13}$C{$^1$H}-NMR (101 MHz, $CD_3CN$)**

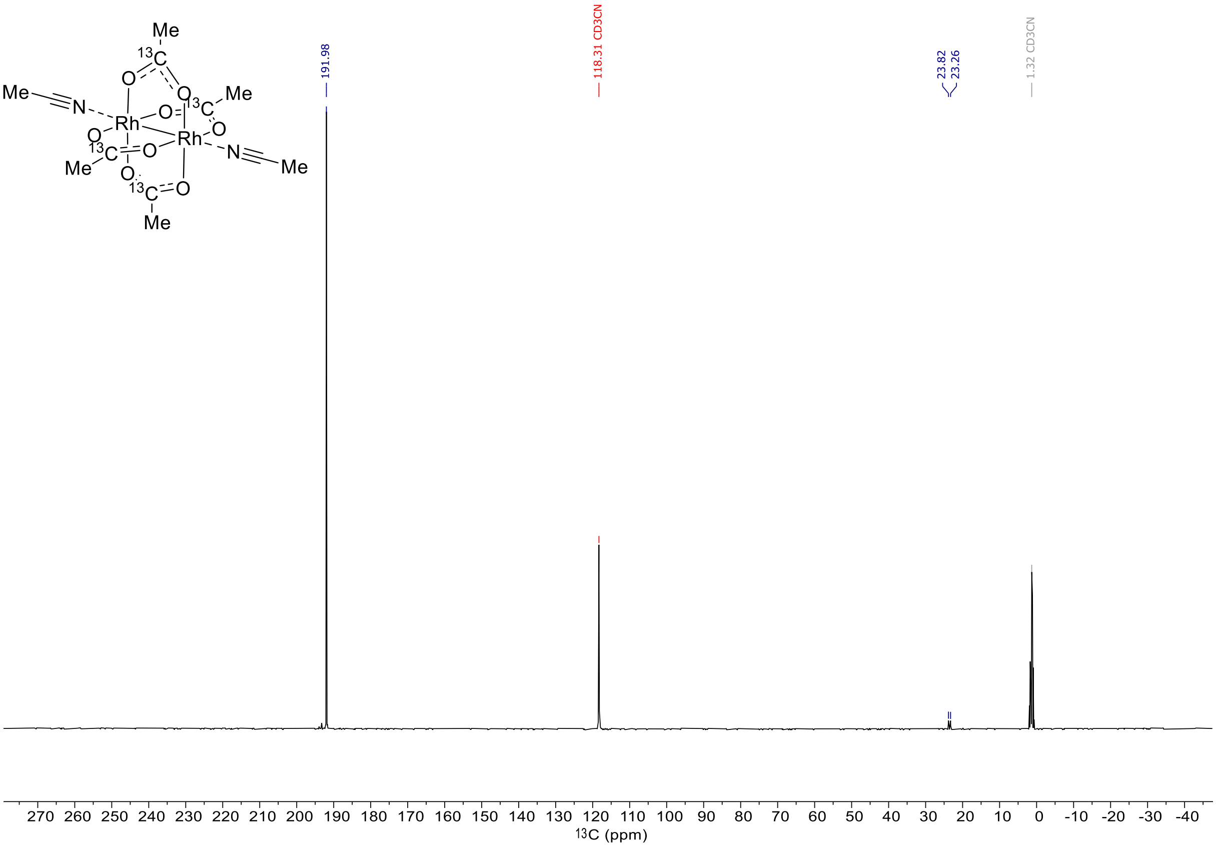

**Rh$_2$(OAc)$_3$(OTfa) (2): $^1$H-NMR (500 MHz, CD$_3$CN)**

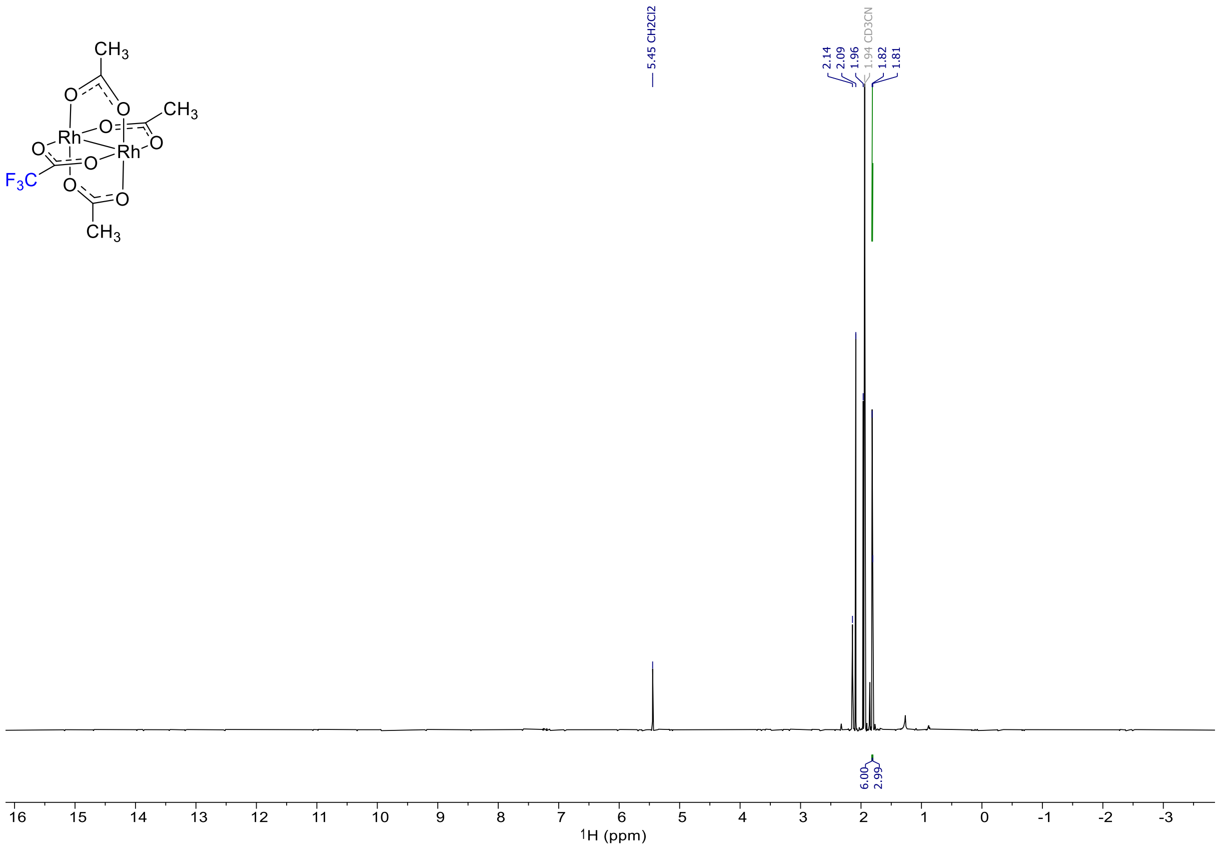


**Rh$_2$(OAc)$_3$(OTfa) (2): $^{19}$F-NMR (565 MHz, CD$_3$CN)**

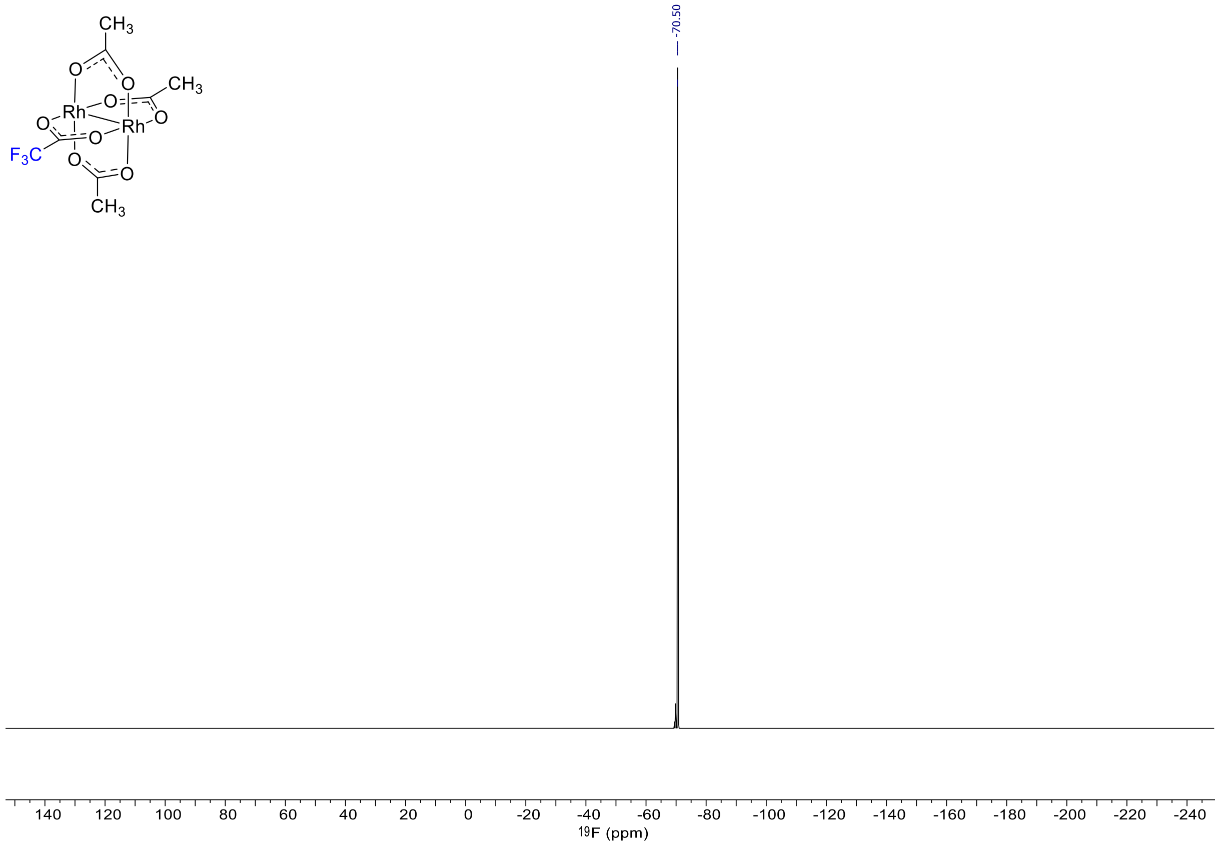

## $Rh_2(OAc)_3(OTfa)$ (2): $^{13}$C-NMR (151 MHz, $CD_3CN$)

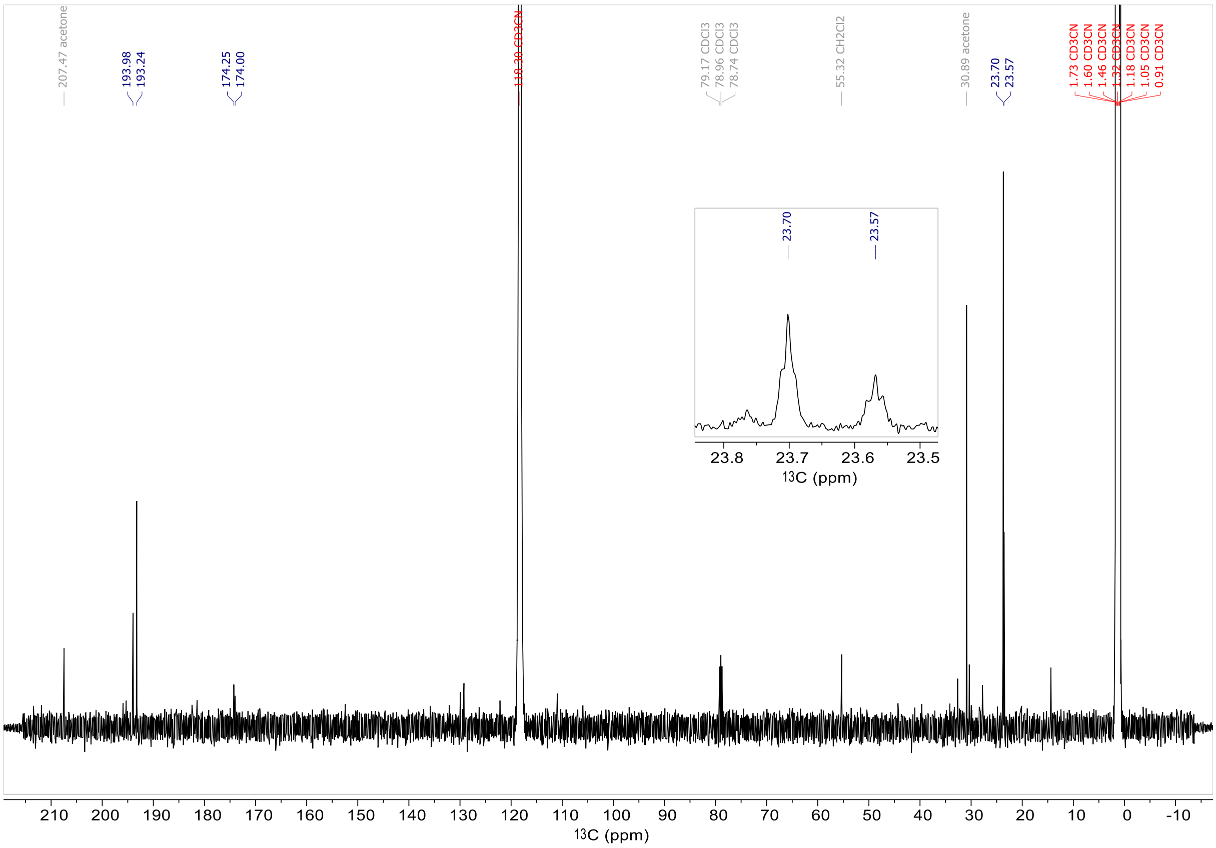


## $Rh_2(OAc)_3(OTfa)$ (2): $^1$H-$^{13}$C-*edited*-HSQC ($CD_3CN$)

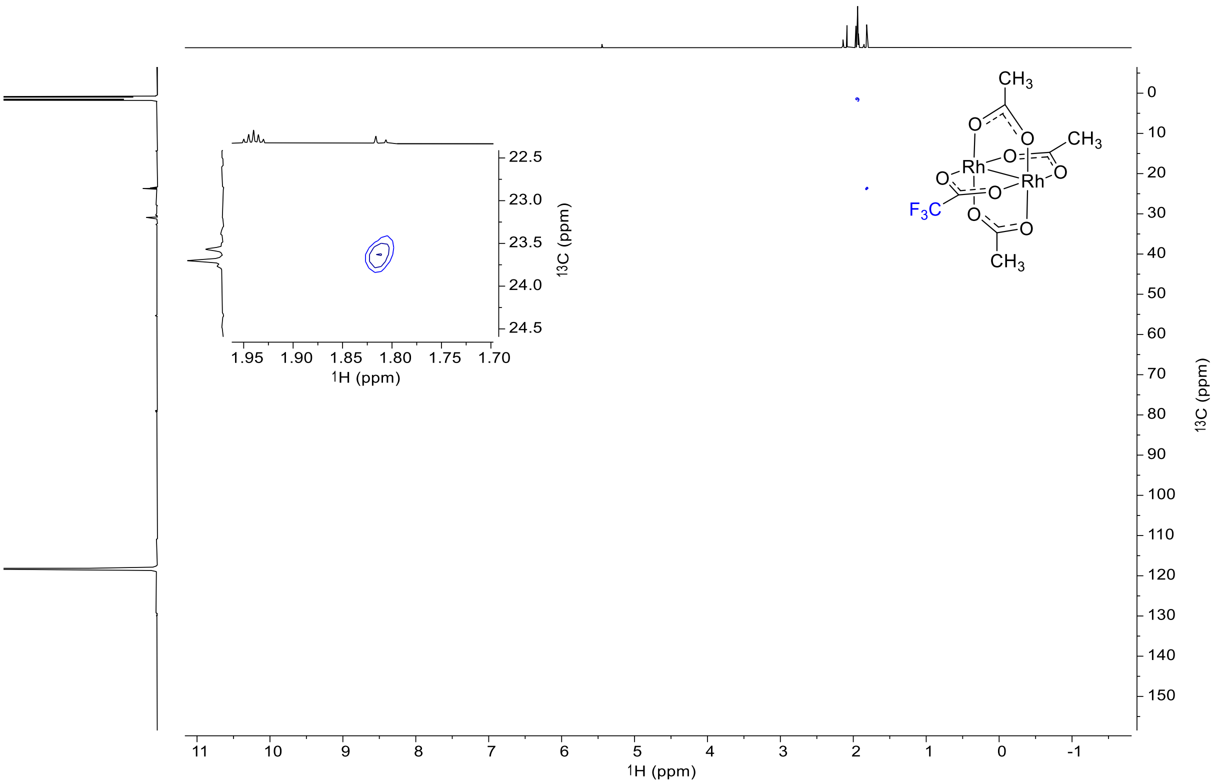

Rh$_2$(OAc)$_3$(OTfa) (2): $^1$H-$^{13}$C-HMBC (CD$_3$CN)

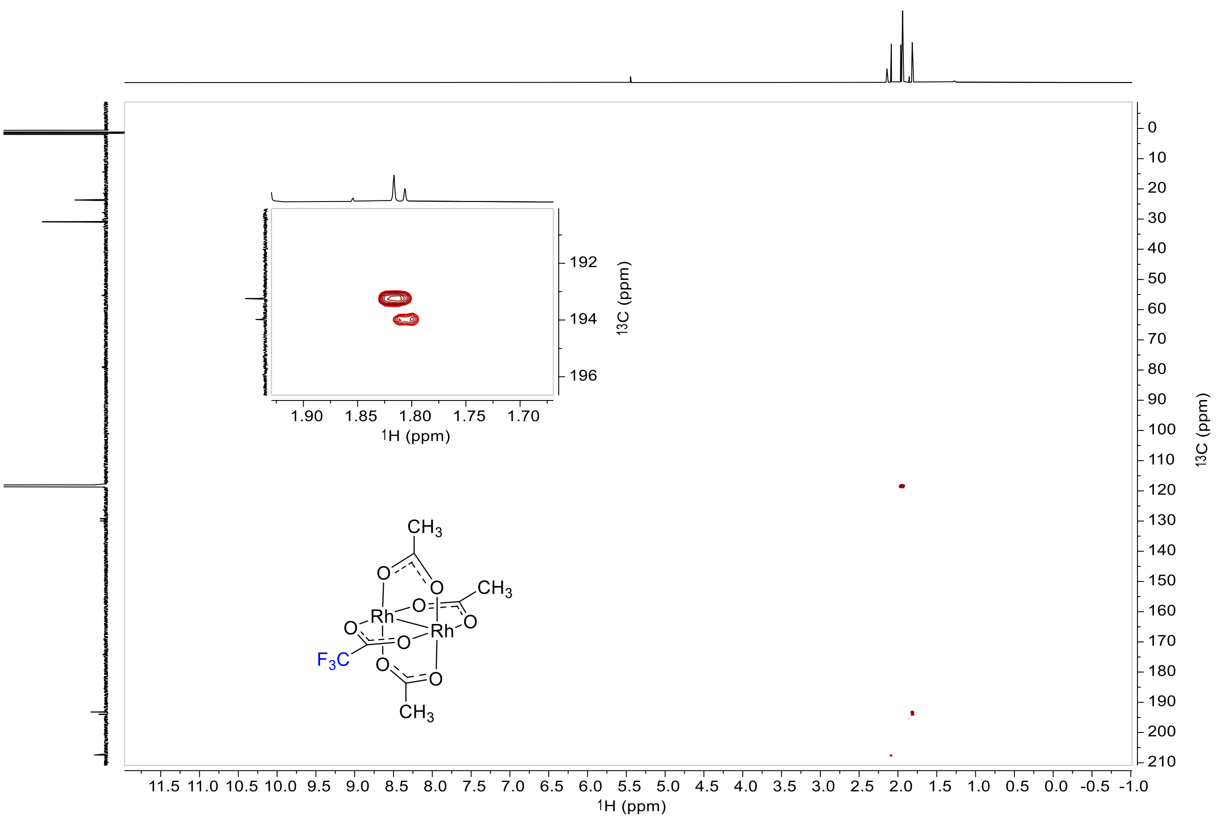


Rh$_2$(OAc)$_3$(OTfa) (2): H(C)Rh (CD$_3$CN)

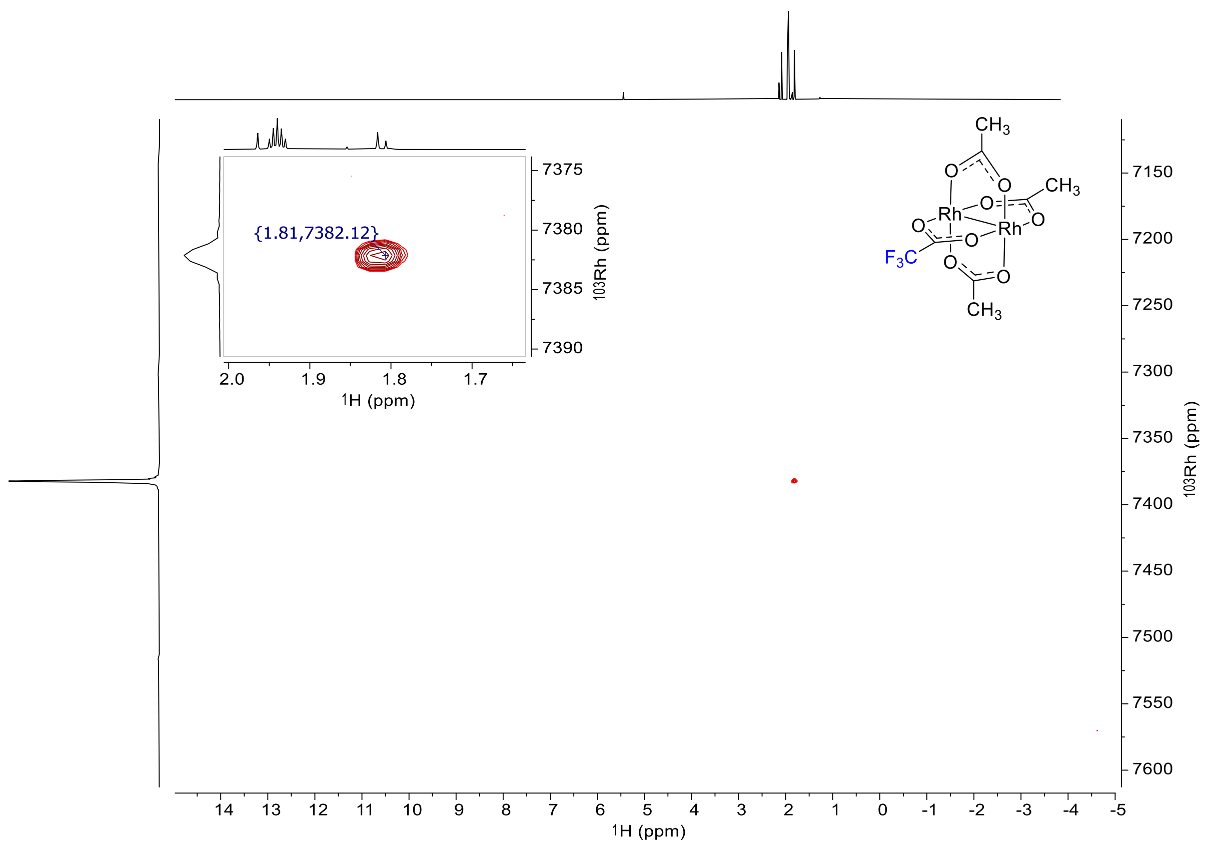

**_cis_-$Rh_2(OAc)_2(OTfa)_2$ (3): $^1$H-NMR (600 MHz, $CD_3CN$)**

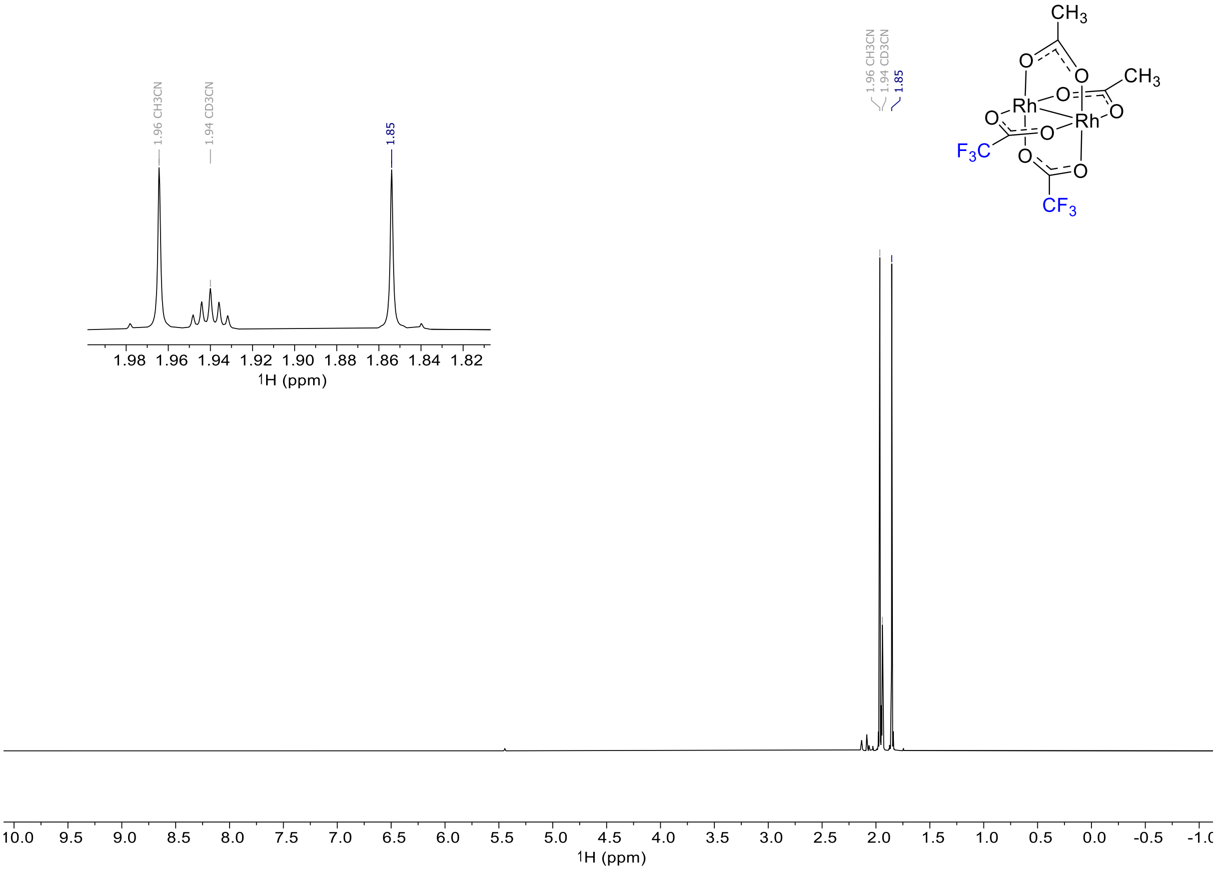


**_cis_-$Rh_2(OAc)_2(OTfa)_2$ (3): $^{13}$C{$^1$H}-NMR (151 MHz, $CD_3CN$)**

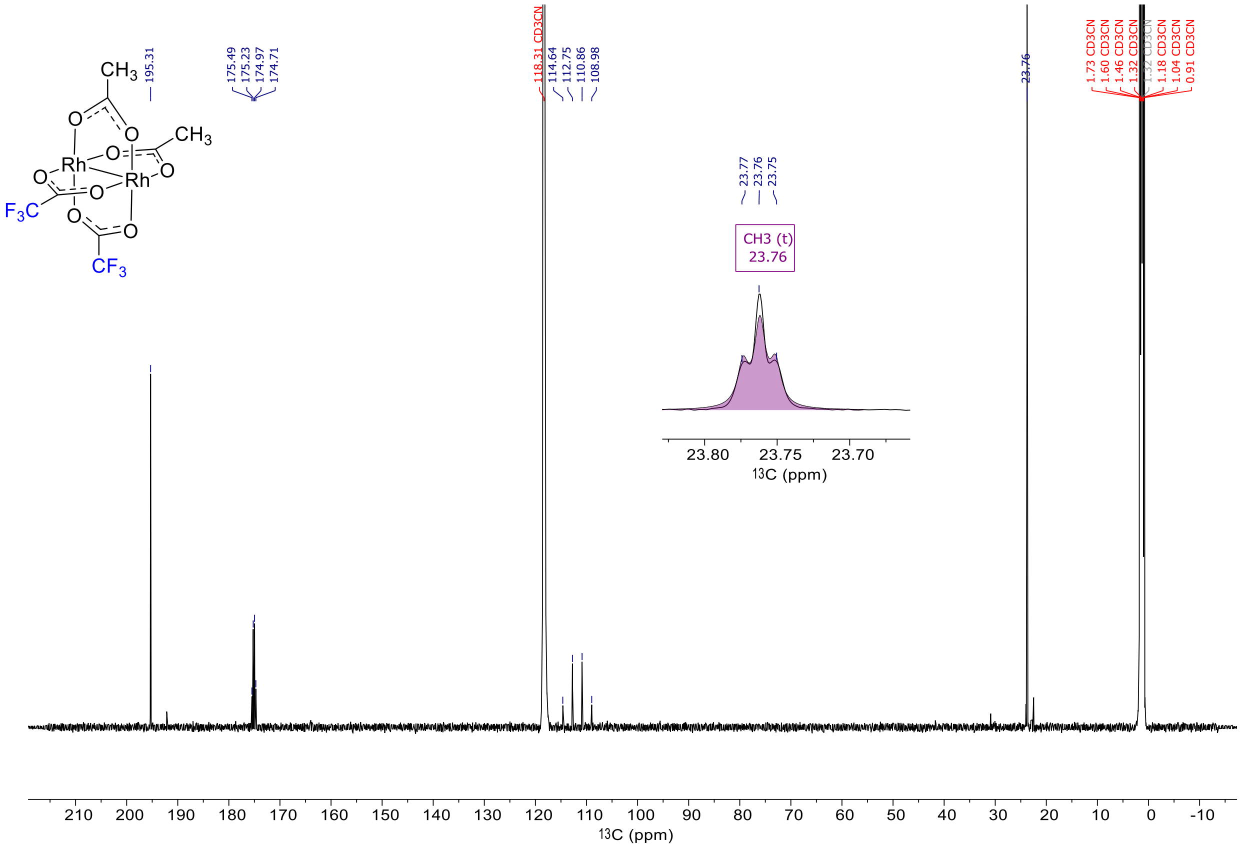

**_cis_-$Rh_2(OAc)_2(OTfa)_2$ (3): $^{19}$F-NMR (565 MHz, $CD_3CN$ )**

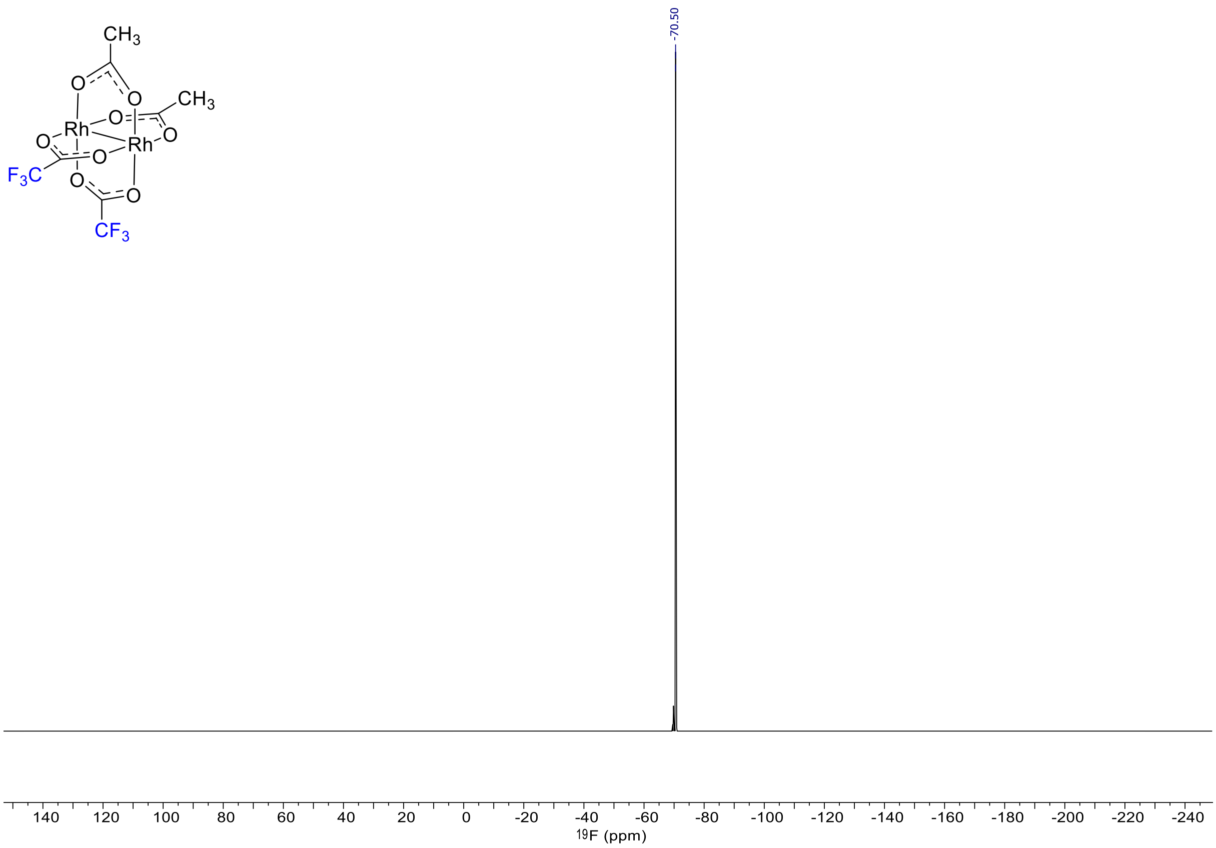


**_cis_-$Rh_2(OAc)_2(OTfa)_2$ (3): H(C)Rh ($CD_3CN$)**

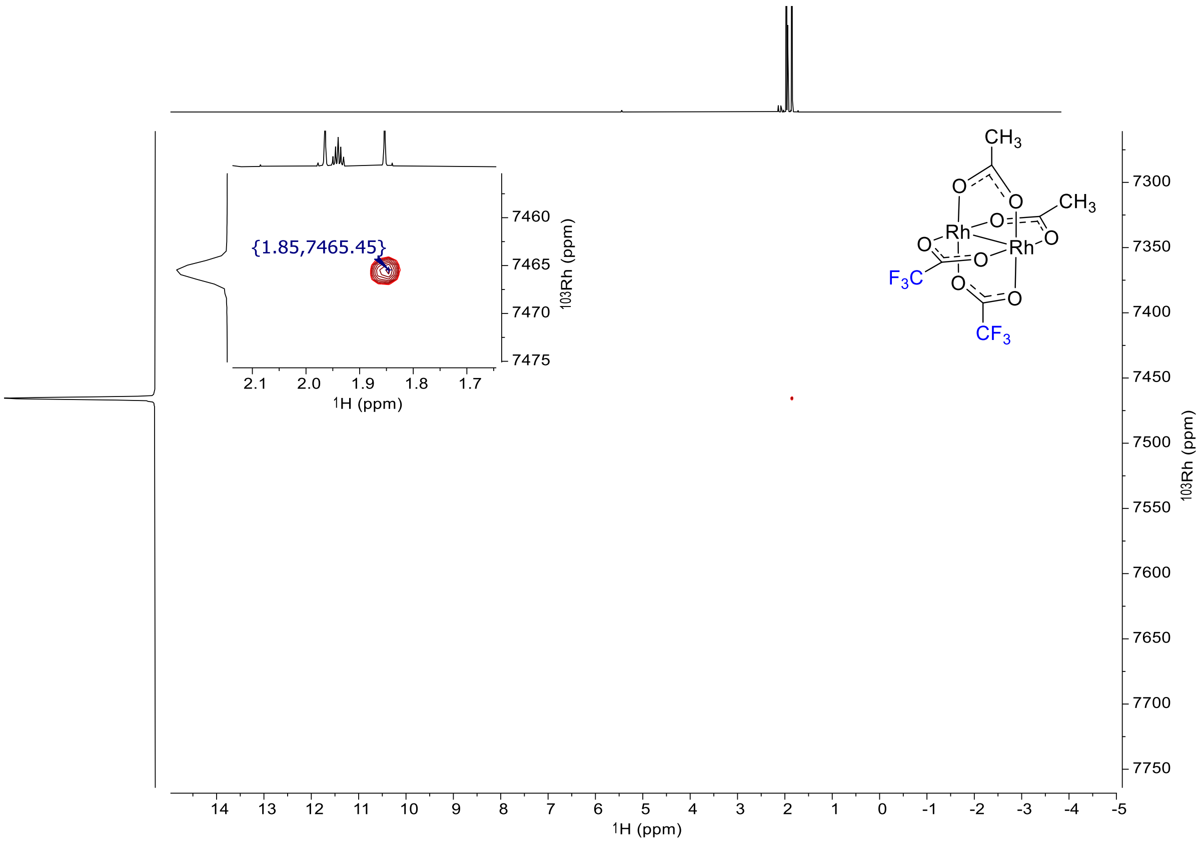

***trans*-$Rh_2(OAc)_2(OTfa)_2$ (4): $^1$H-NMR (600 MHz, $CD_3CN$)**

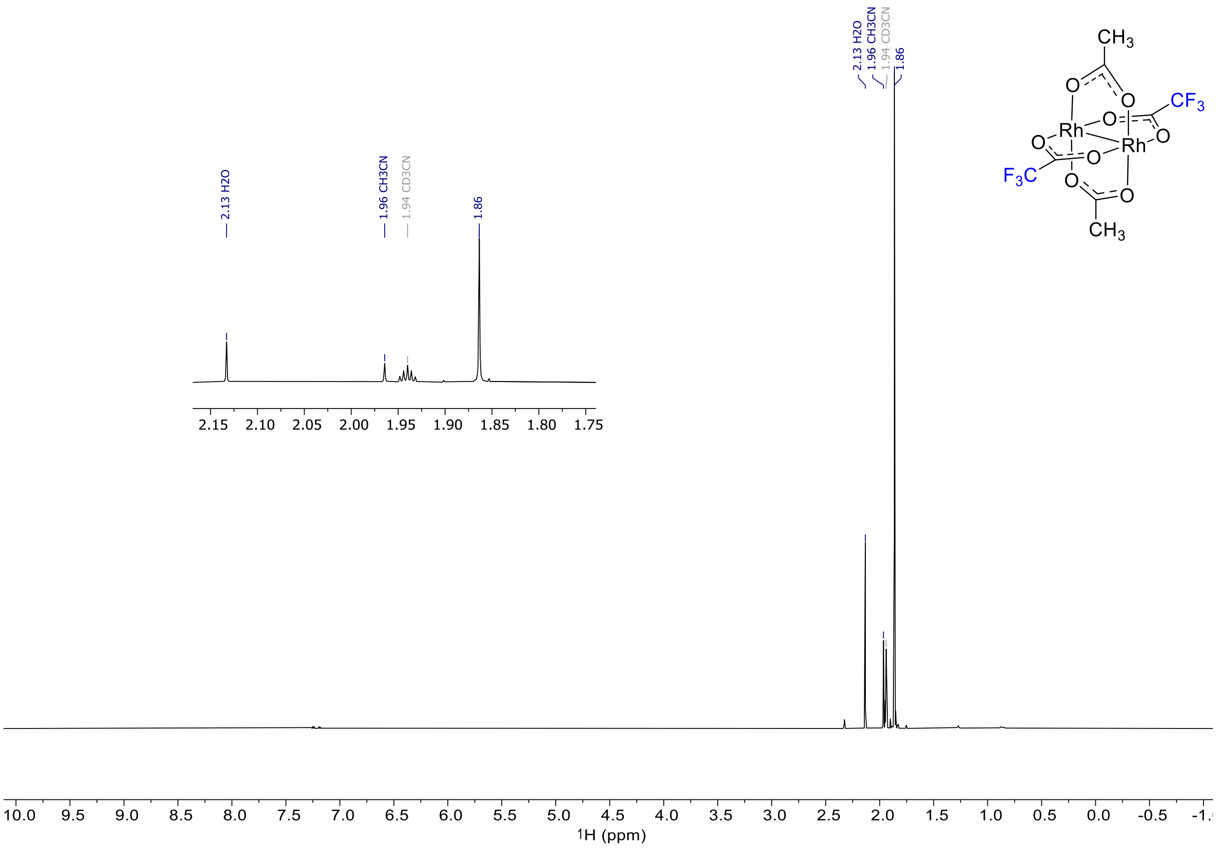


***trans*-$Rh_2(OAc)_2(OTfa)_2$ (4): $^{13}C\{^1H\}$-NMR (151 MHz, $CD_3CN$)**

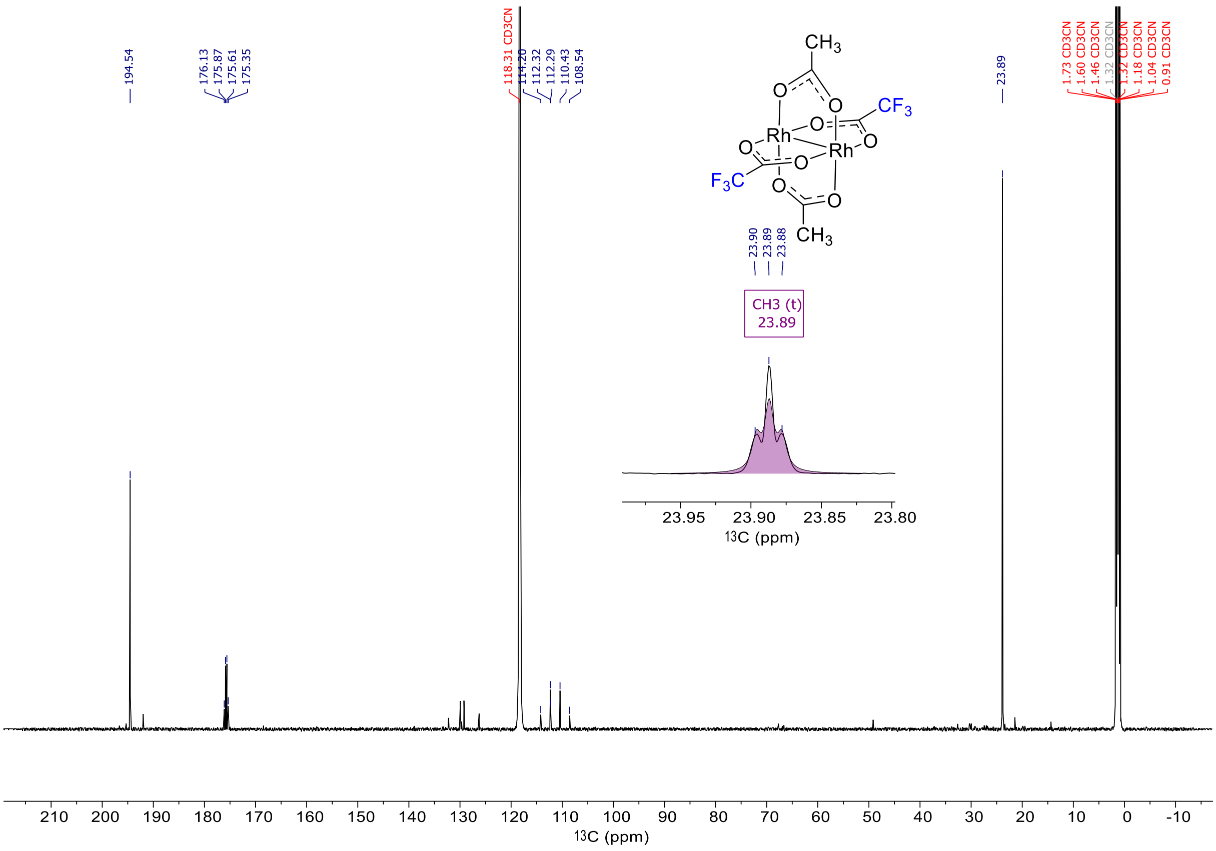

***trans*-Rh2(OAc)2(OTfa)2 (4): 19F-NMR (565 MHz, CD3CN)**

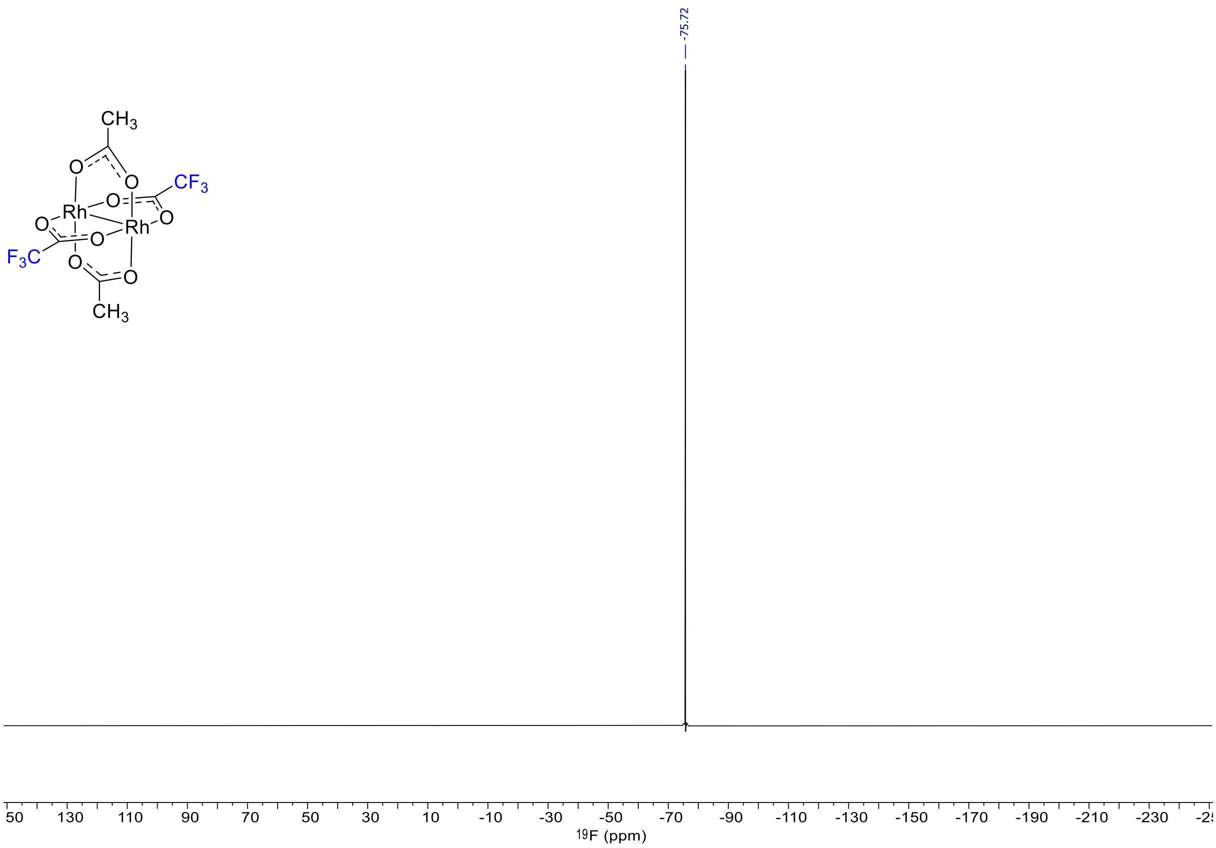


***trans*-Rh2(OAc)2(OTfa)2 (4): H(C)Rh (CD3CN)**

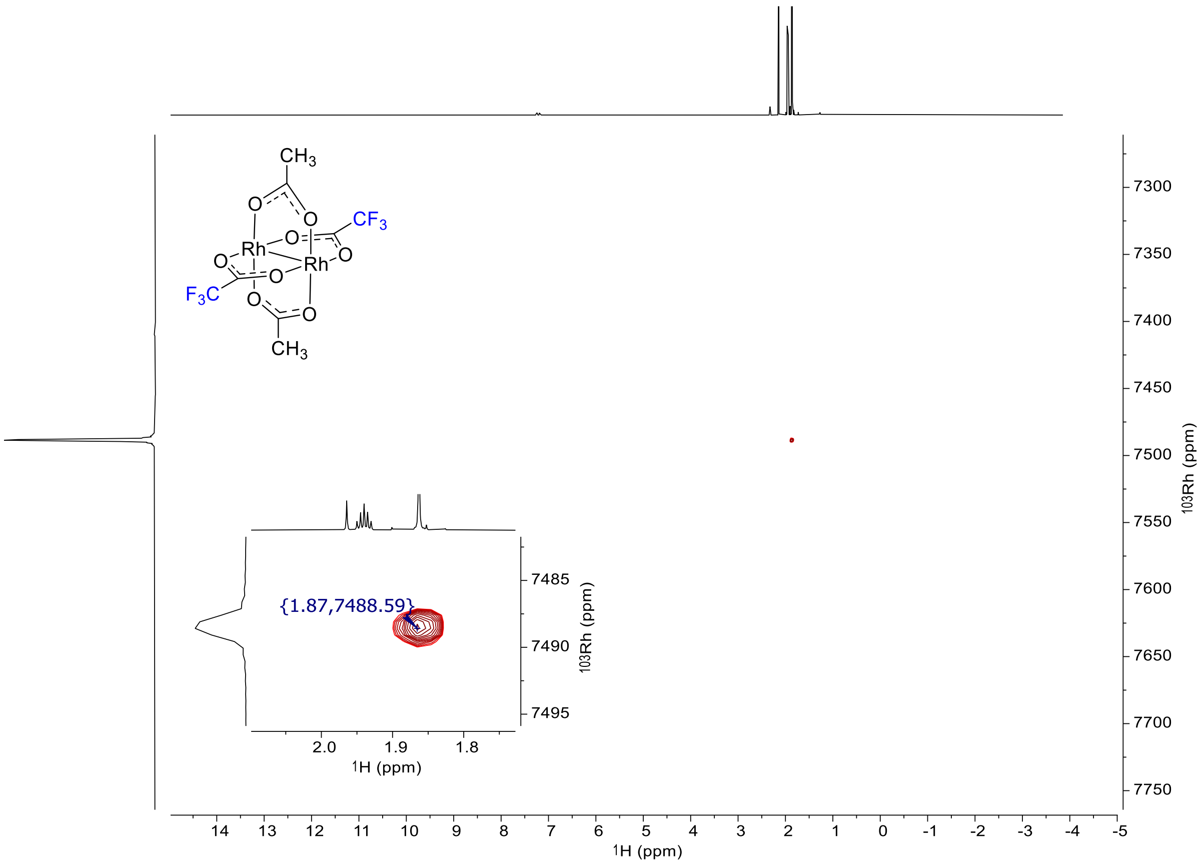

**$Rh_2(OAc)(OTfa)_3$ (5): $^1$H-NMR (500 MHz, $CD_3CN$)**

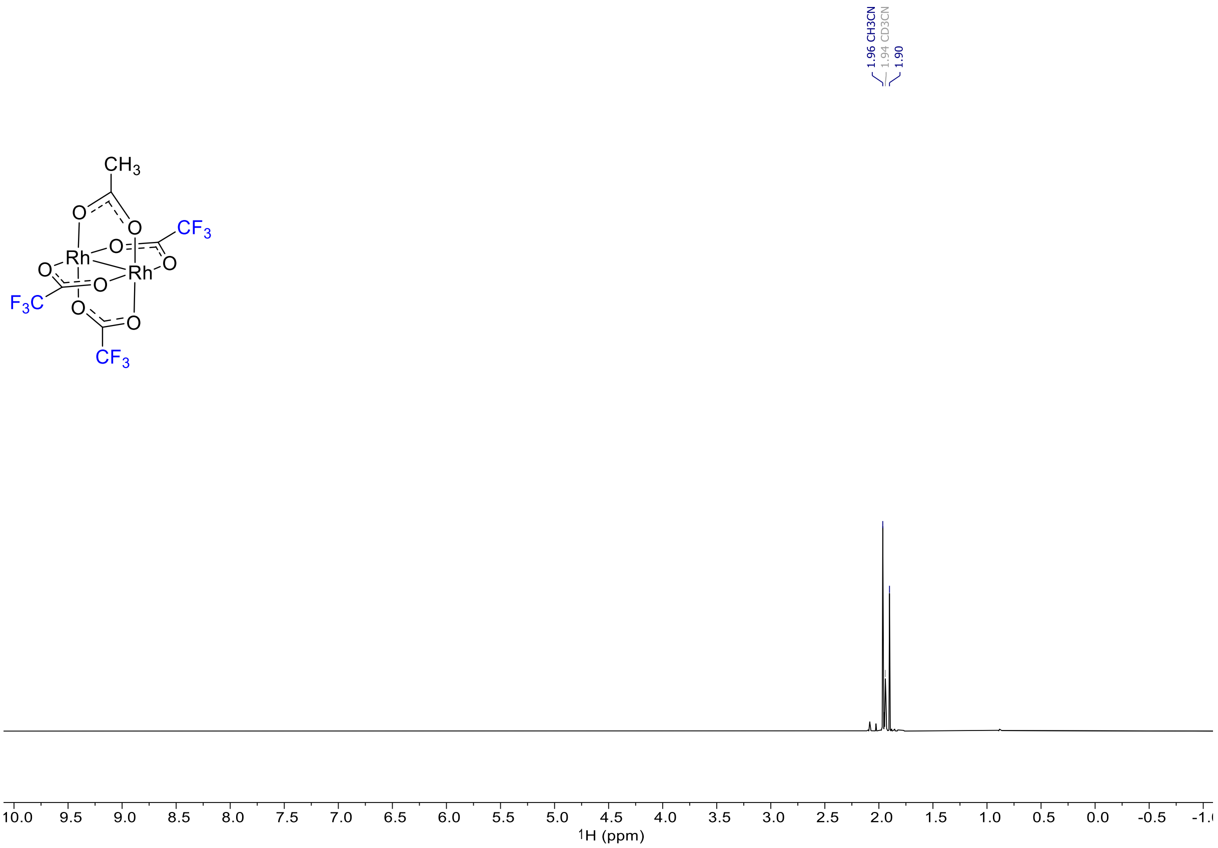


**$Rh_2(OAc)(OTfa)_3$ (5): $^{13}C\{^1H\}$-NMR (151 MHz, $CD_3CN$)**

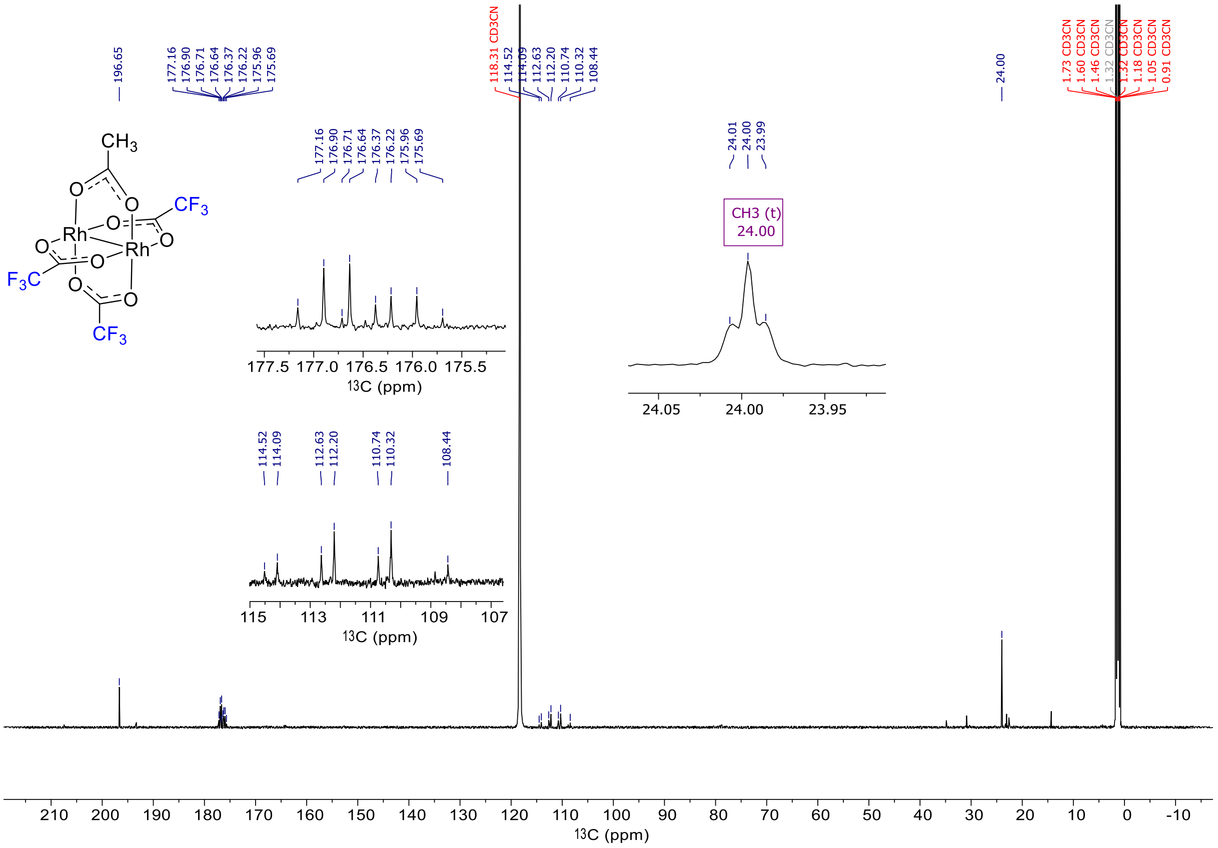

**$Rh_2(OAc)(OTfa)_3$ (5): $^{19}$F-NMR (565 MHz, $CD_3CN$)**

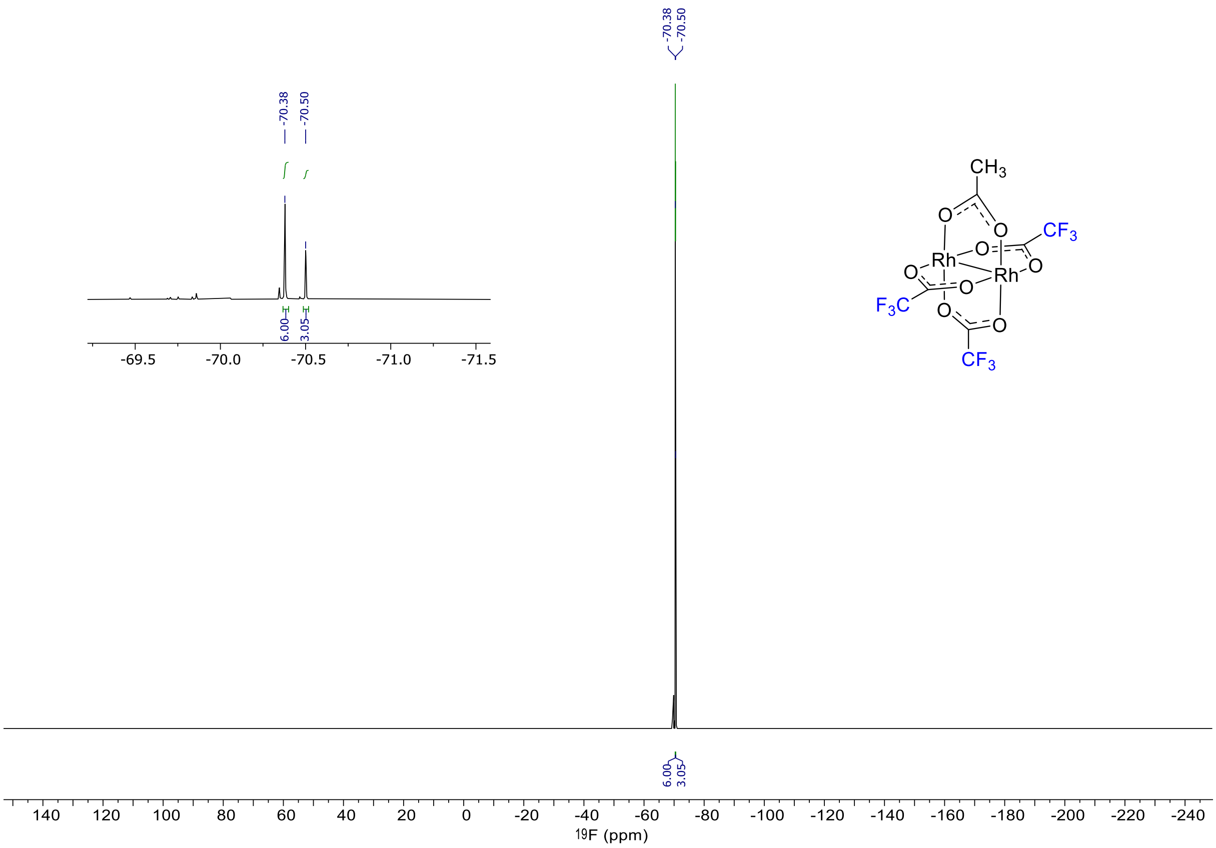


**$Rh_2(OAc)(OTfa)_3$ (5): H(C)Rh ($CD_3CN$)**

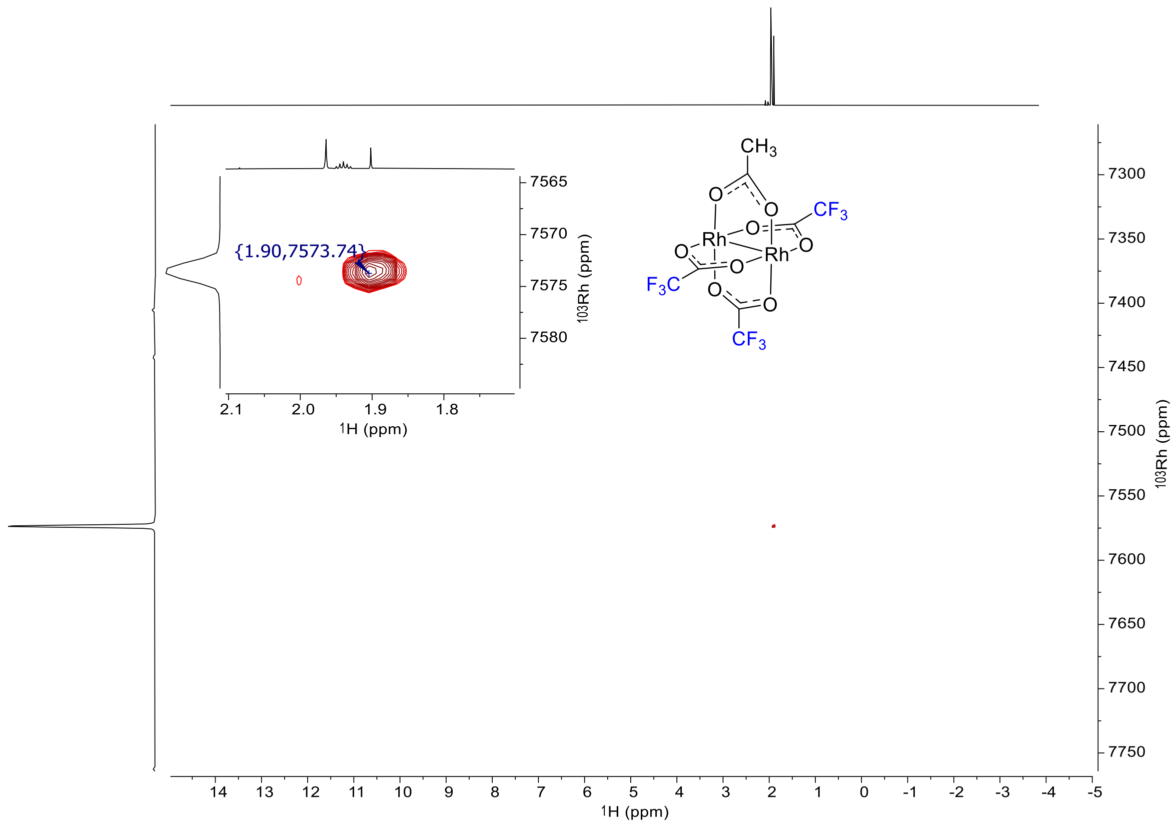

**Dirhodium(II) tetra(trifluoracetate) ($Rh_2(OTfa)_4$) (6): $^{19}$F-NMR (565 MHz, $CD_3CN$)**

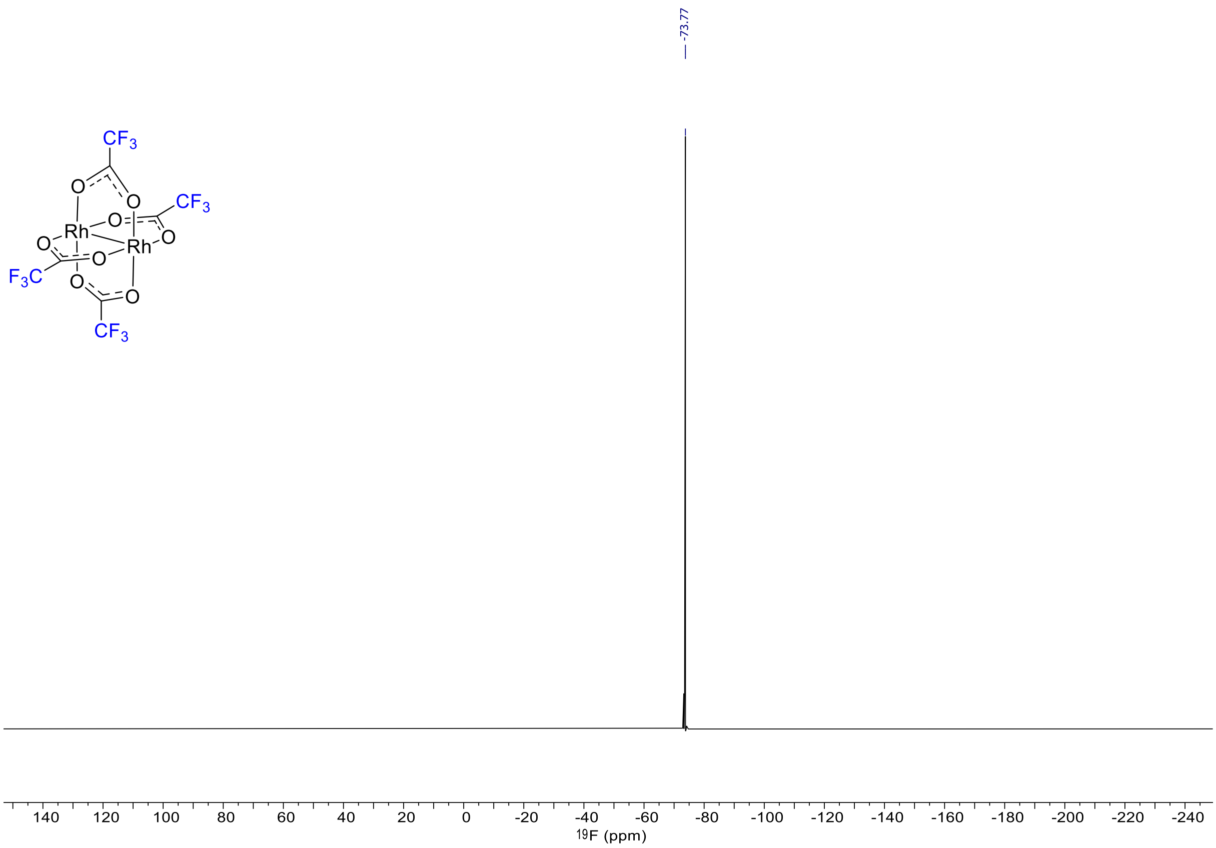


**Dirhodium(II) tetra(trifluoracetate) ($Rh_2(OTfa)_4$) (6): $^{13}$C-NMR (151 MHz, $CD_3CN$)**

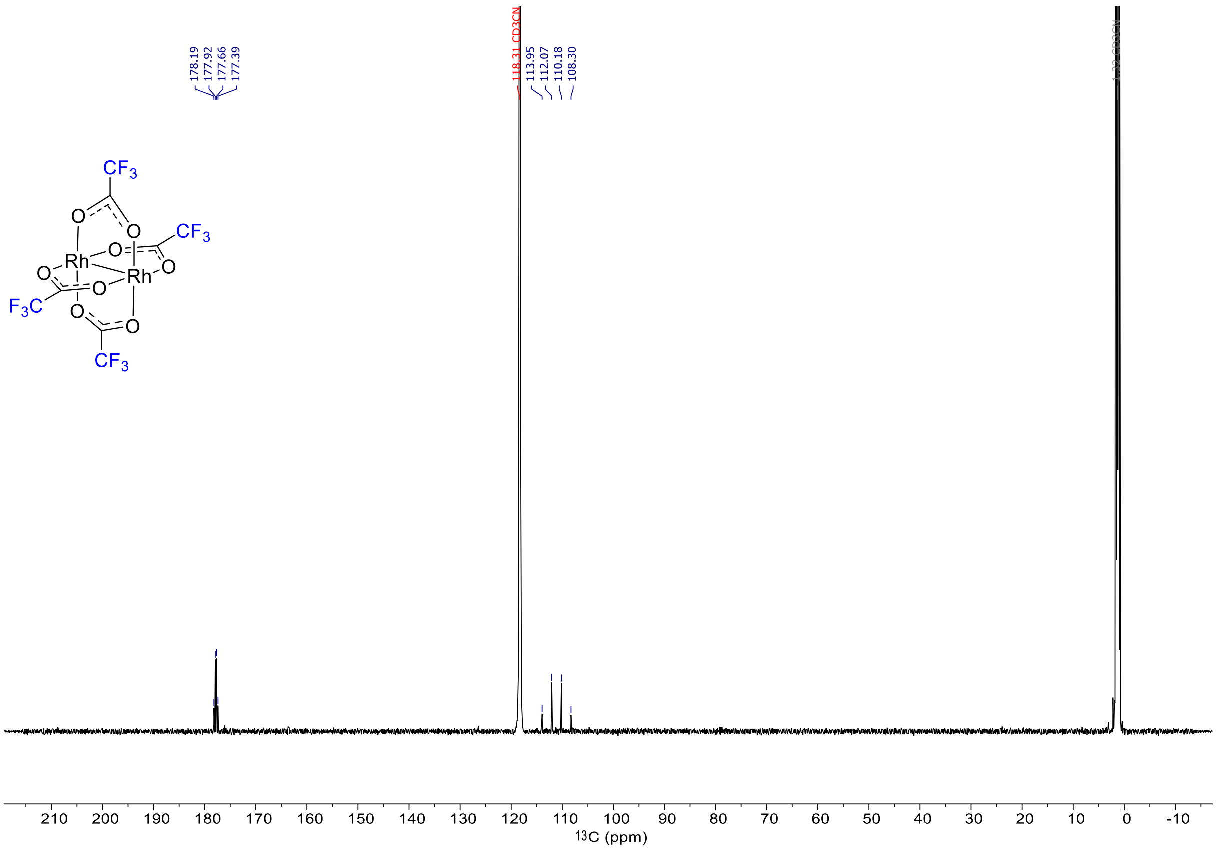

**Dirhodium(II) tetra(trifluoracetate) ($Rh_2(OTfa)_4$) (6): $^{103}$Rh-NMR (15.92 MHz, $CD_3CN$)**

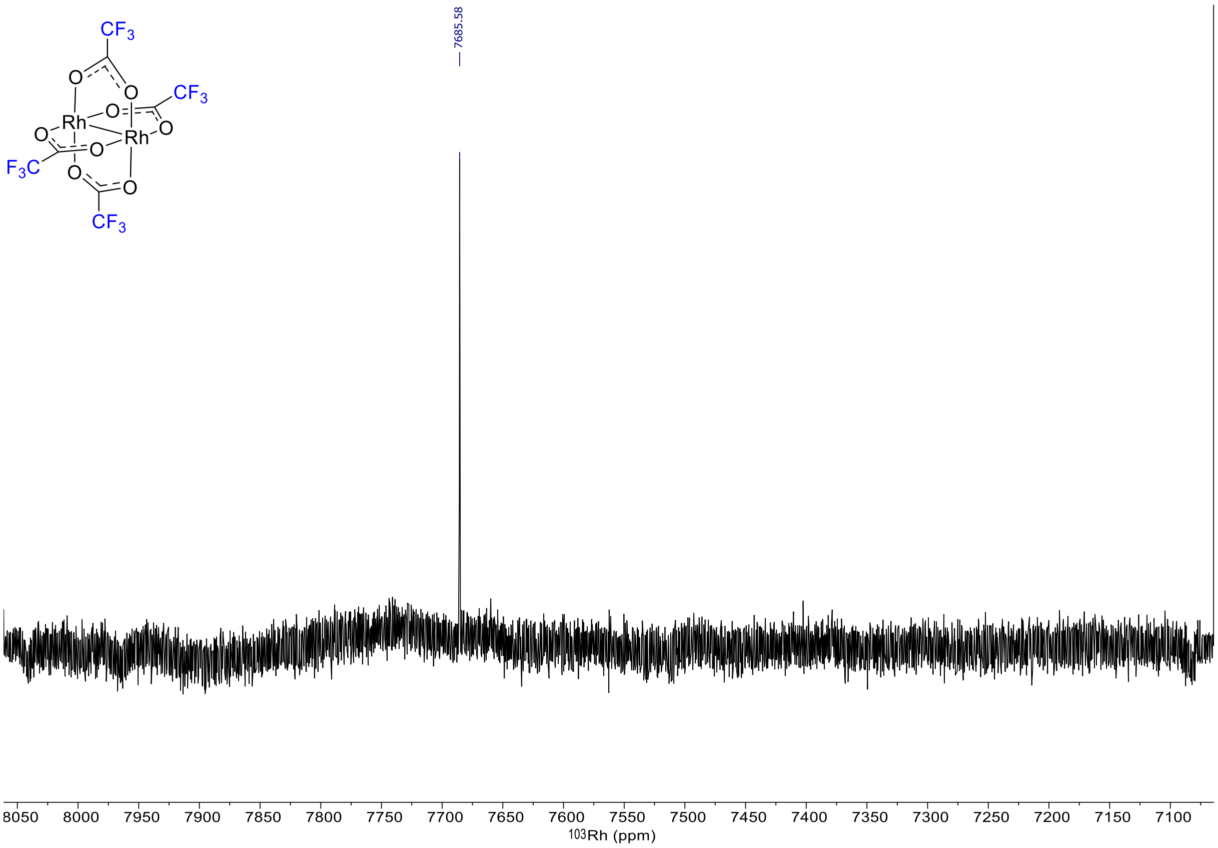

**$Rh_2(OPiv)_4$ (7): $^1$H-NMR (400 MHz, $CD_3CN$)**

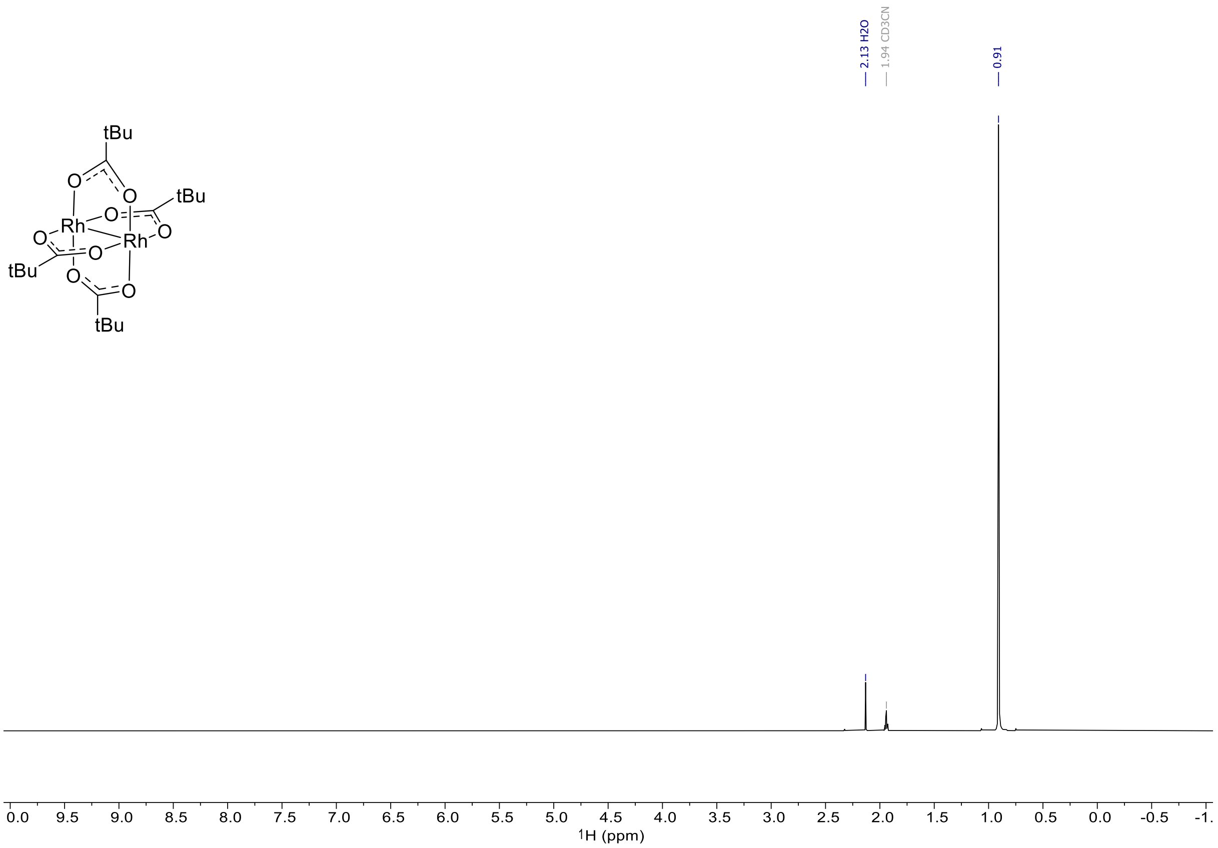


**$Rh_2(OPiv)_4$ (7): H(C)Rh ($CD_3CN$)**

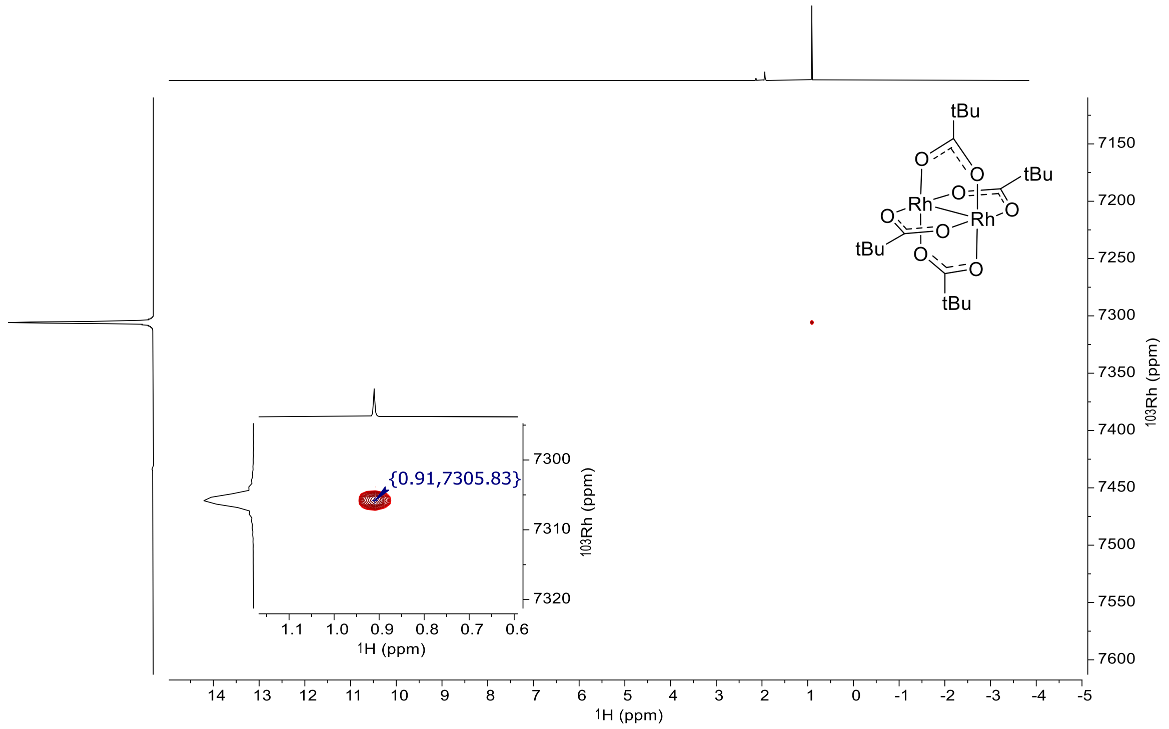

**$Rh_2(HCO_2)_4$ (8): $^1$H-NMR (400 MHz, $CD_3CN$)**

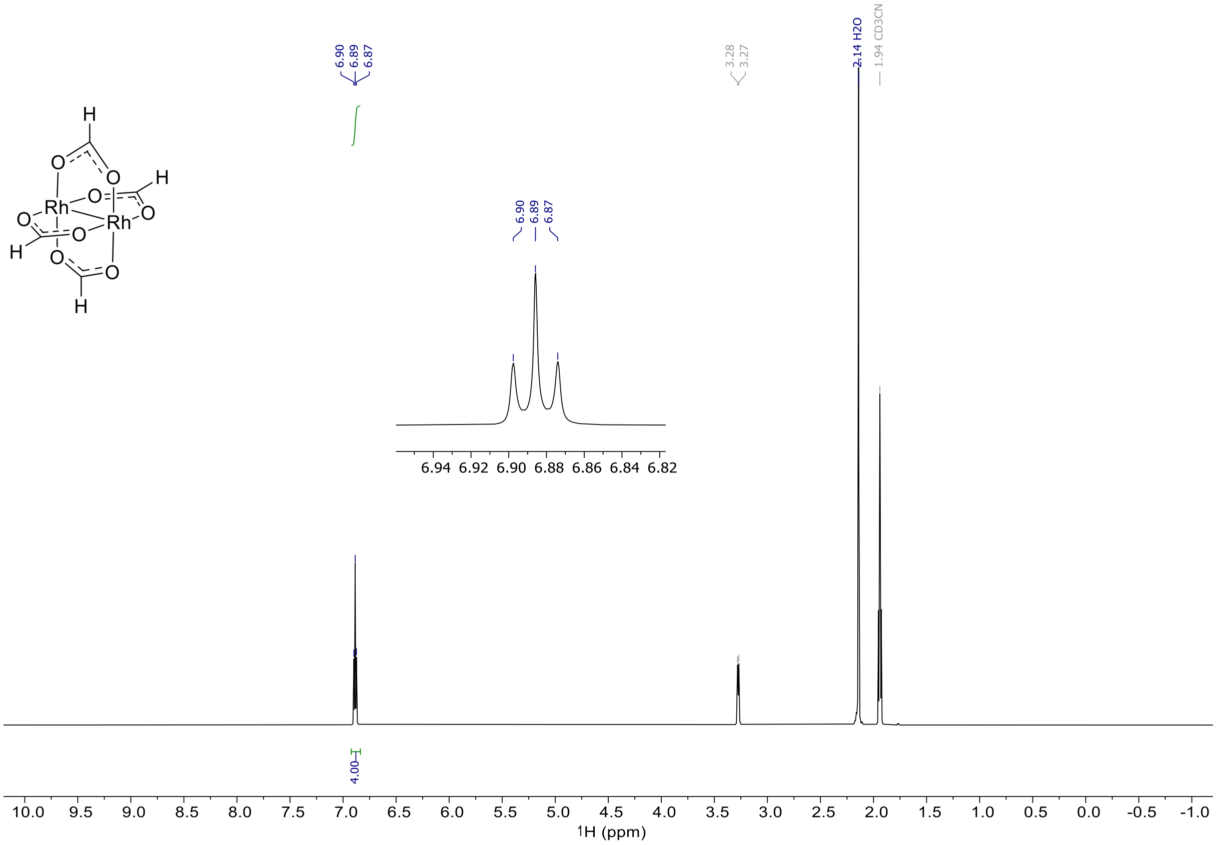


**$Rh_2(HCO_2)_4$ (8): $^{13}C\{^1H\}$-NMR (101 MHz, $CD_3CN$)**

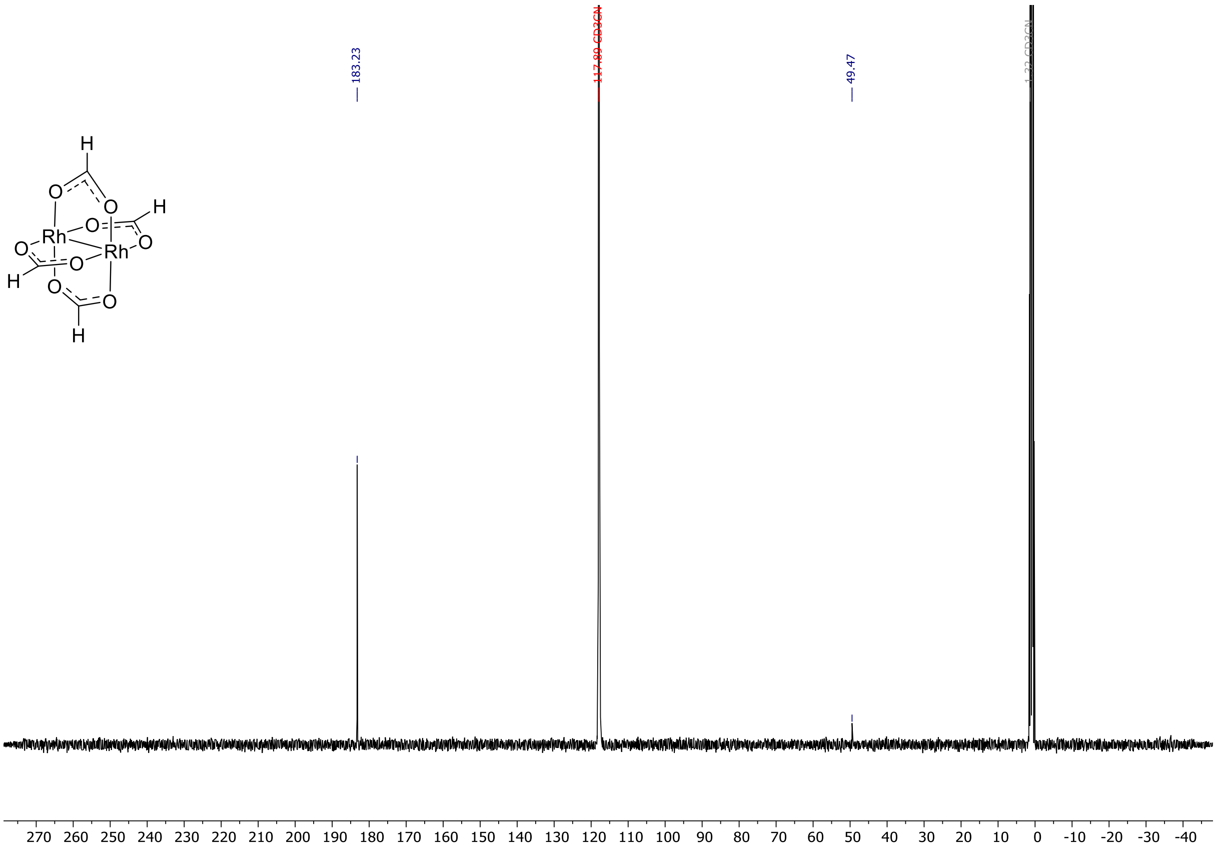

**$Rh_2(HCO_2)_4$ (8): $^1$H-$^{13}$C-*edited*-HSQC ($CD_3CN$)**

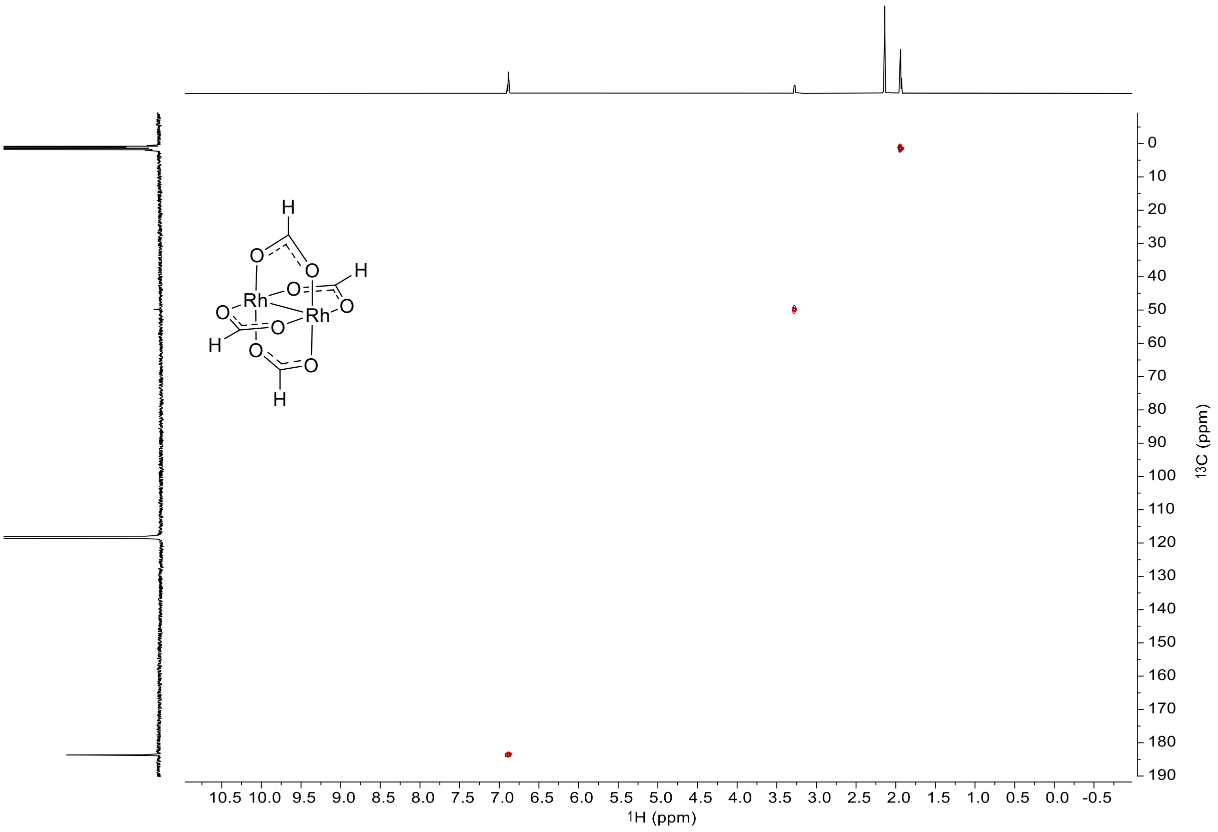


**$Rh_2(HCO_2)_4$ (8): $^1$H-$^{103}$Rh-HMBC ($CD_3CN$)**

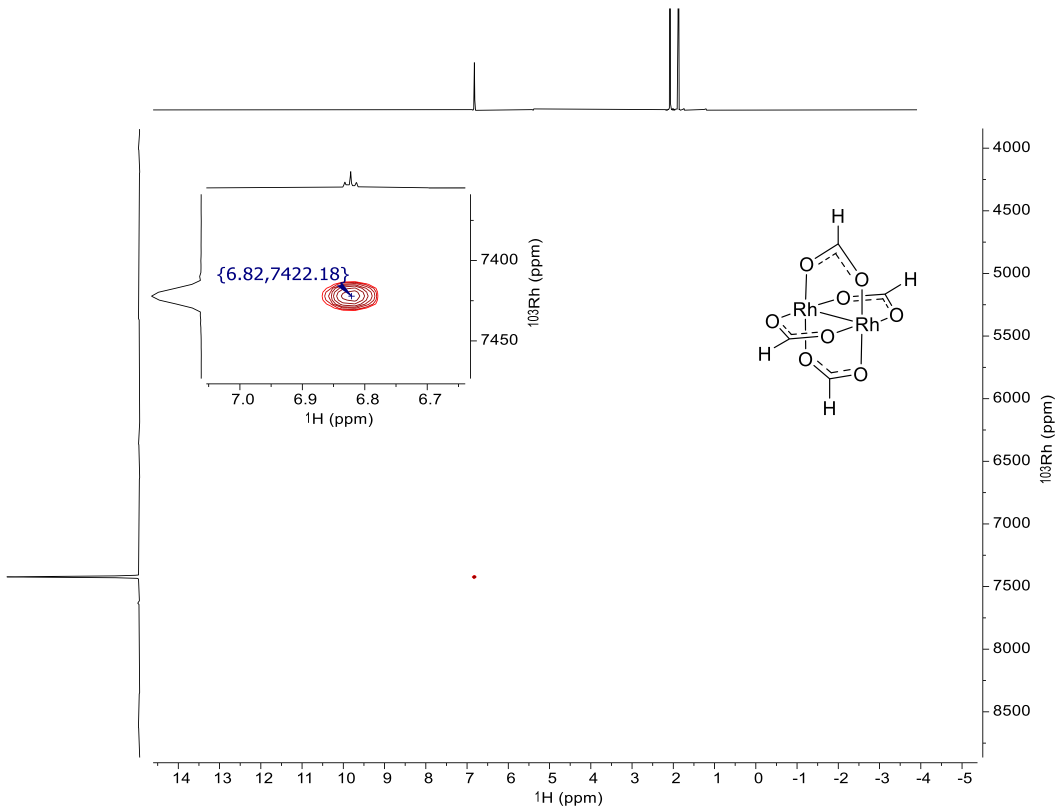
{6.82,7422.18}
7400
7450
103Rh (ppm)
7.0
6.9
6.8
6.7
1H (ppm)
H
O
O
H
Rh
O
O
Rh
O
O
H
O
O
H
4000
4500
5000
5500
6000
6500
7000
7500
8000
8500
103Rh (ppm)
14
13
12
11
10
9
8
7
6
5
4
3
2
1
0
-1
-2
-3
-4
-5
1H (ppm)

## $Rh_2(OBz)_4$ (9): $^1$H-NMR (400 MHz, $CD_3CN$)

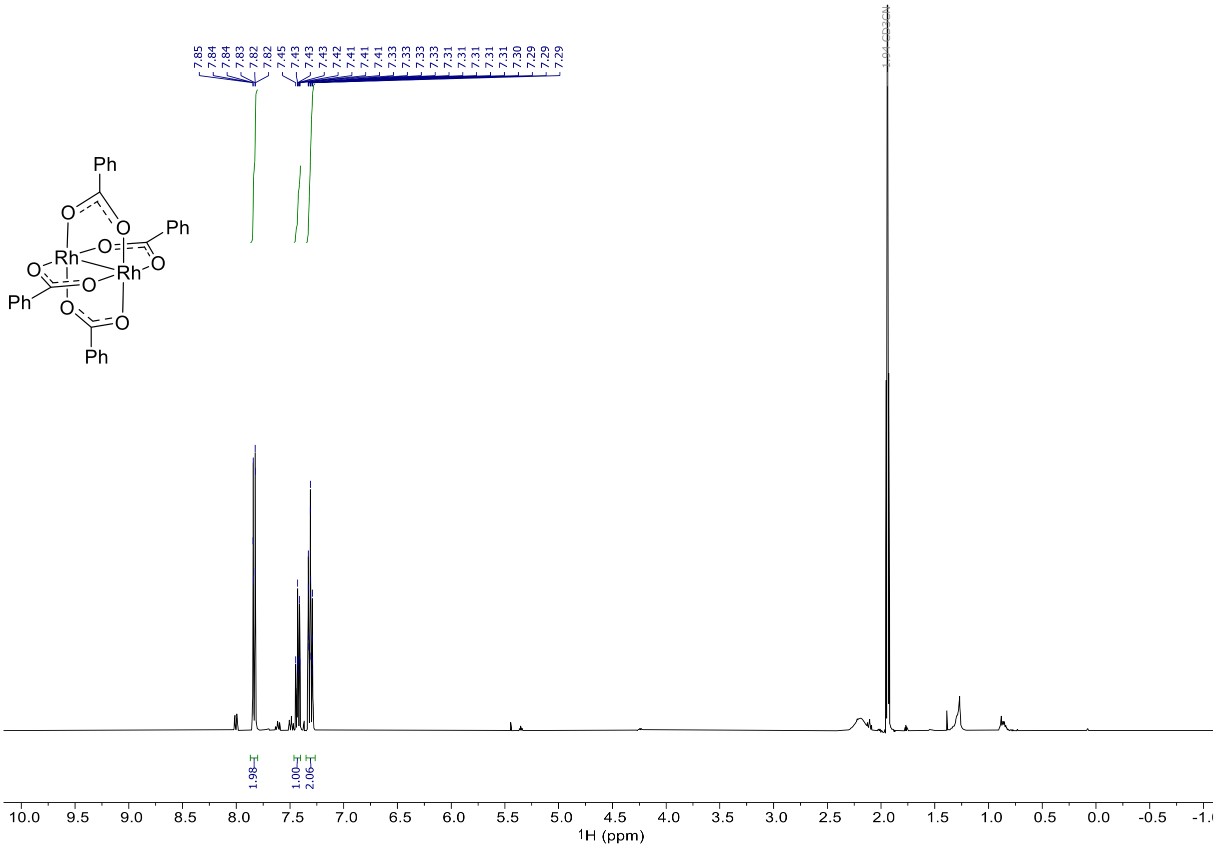


## $Rh_2(OBz)_4$ (9): $^{13}$C{$^1$H}-NMR (101 MHz, $CD_3CN$)

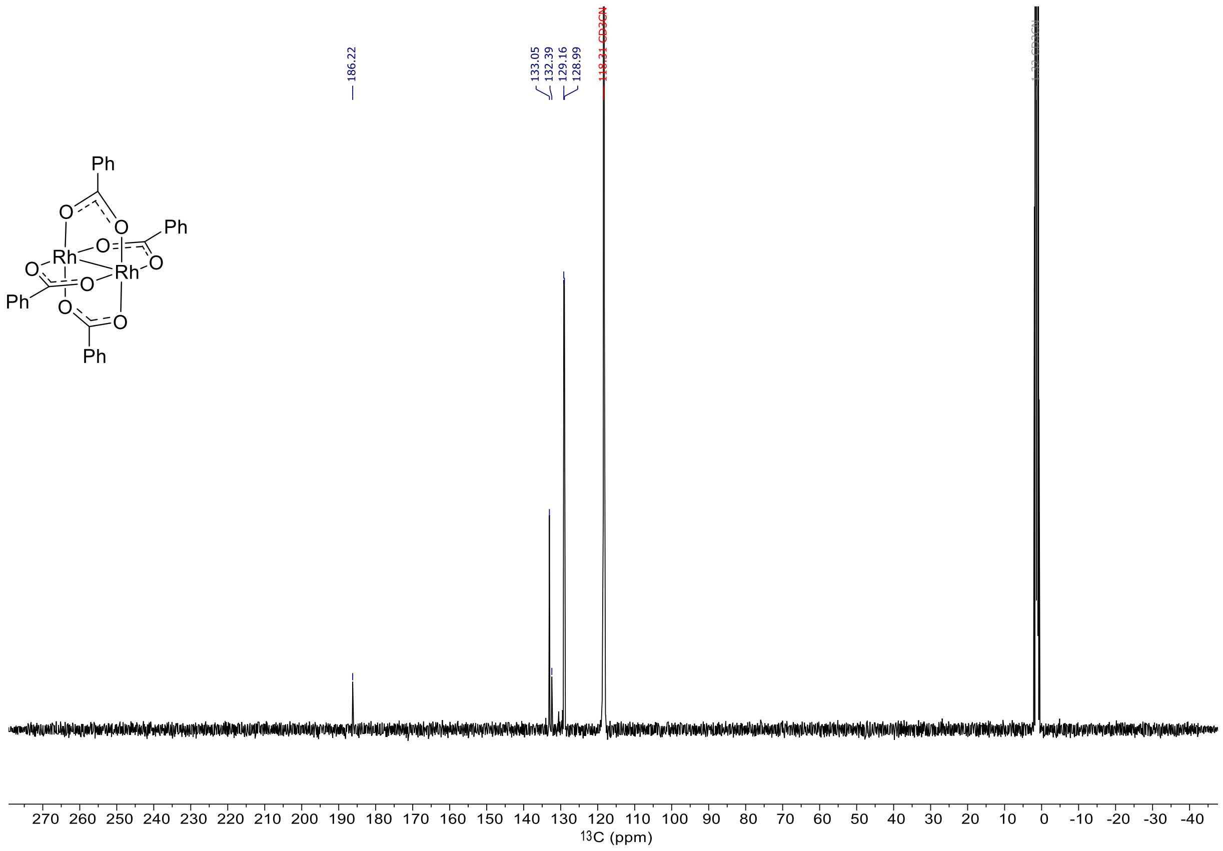

**$Rh_2(OBz)_4$ (9): H(C)Rh ($CD_3CN$)**

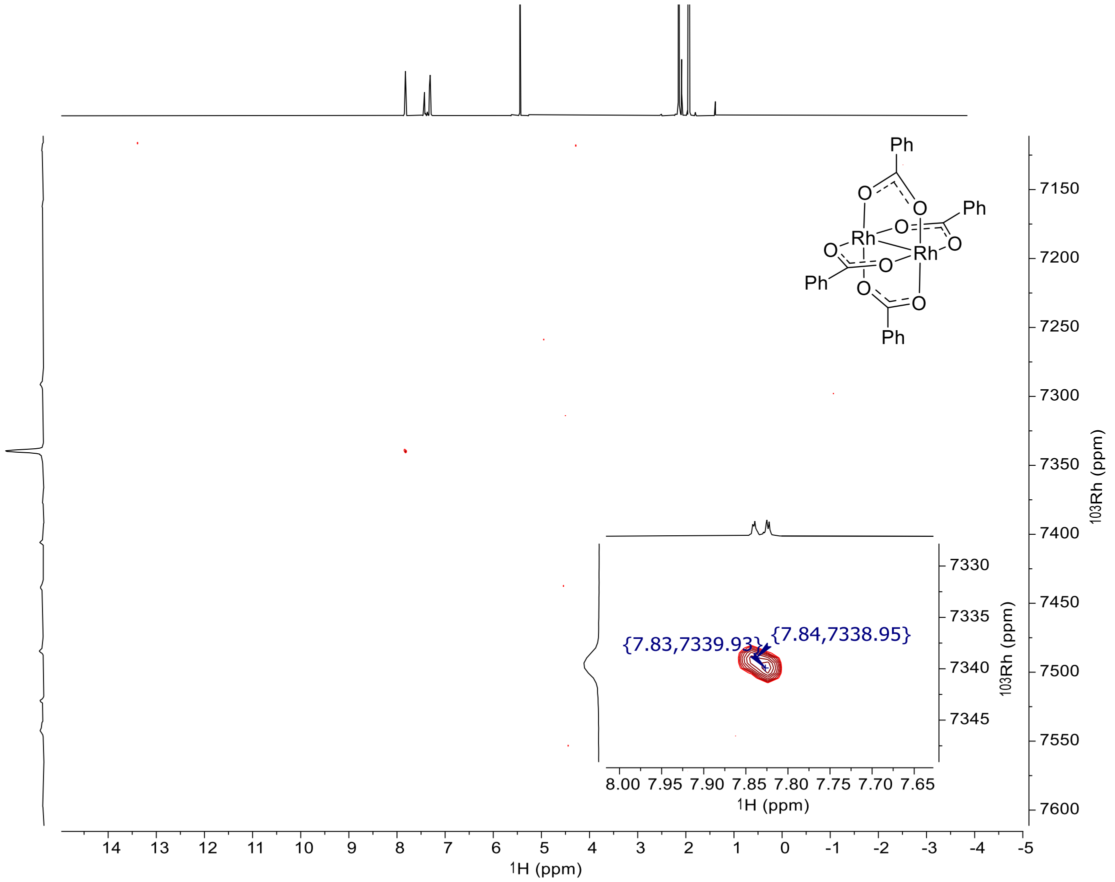

**$Rh_2(OBz)_4$ (9): $^1$H-NMR (500 MHz, [$D_8$]-THF)**

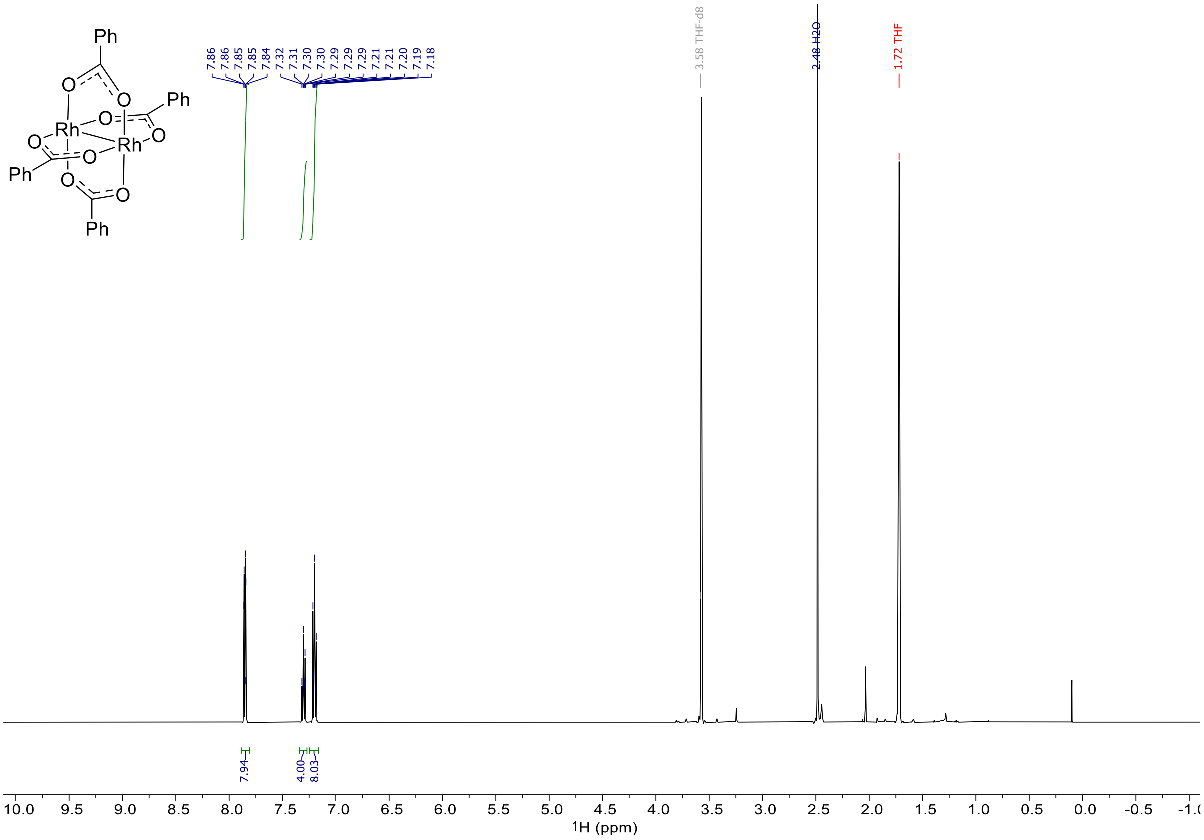


**$Rh_2(OBz)_4$ (9): H(C)Rh ([$D_8$]-THF)**

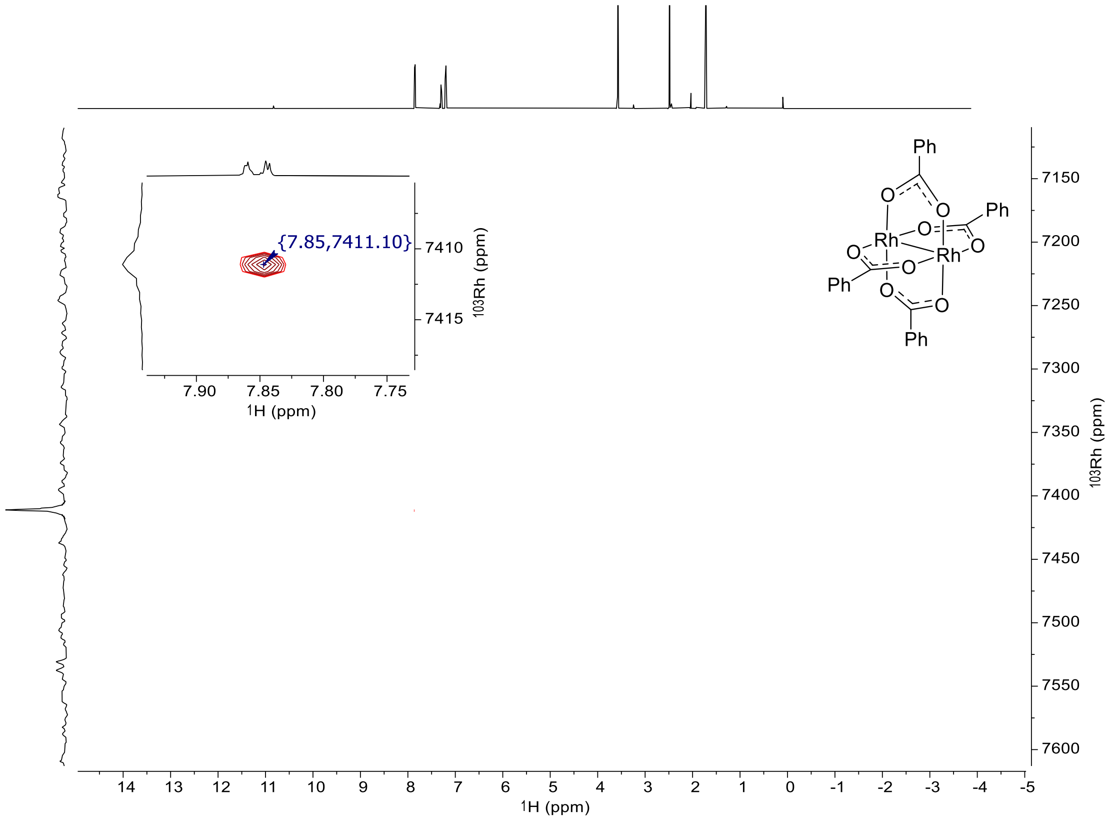

**$Rh_2$(*p*-$FC_6H_4COO$)$_4$ (10): $^1$H-NMR (400 MHz, [$D_8$]-THF)**

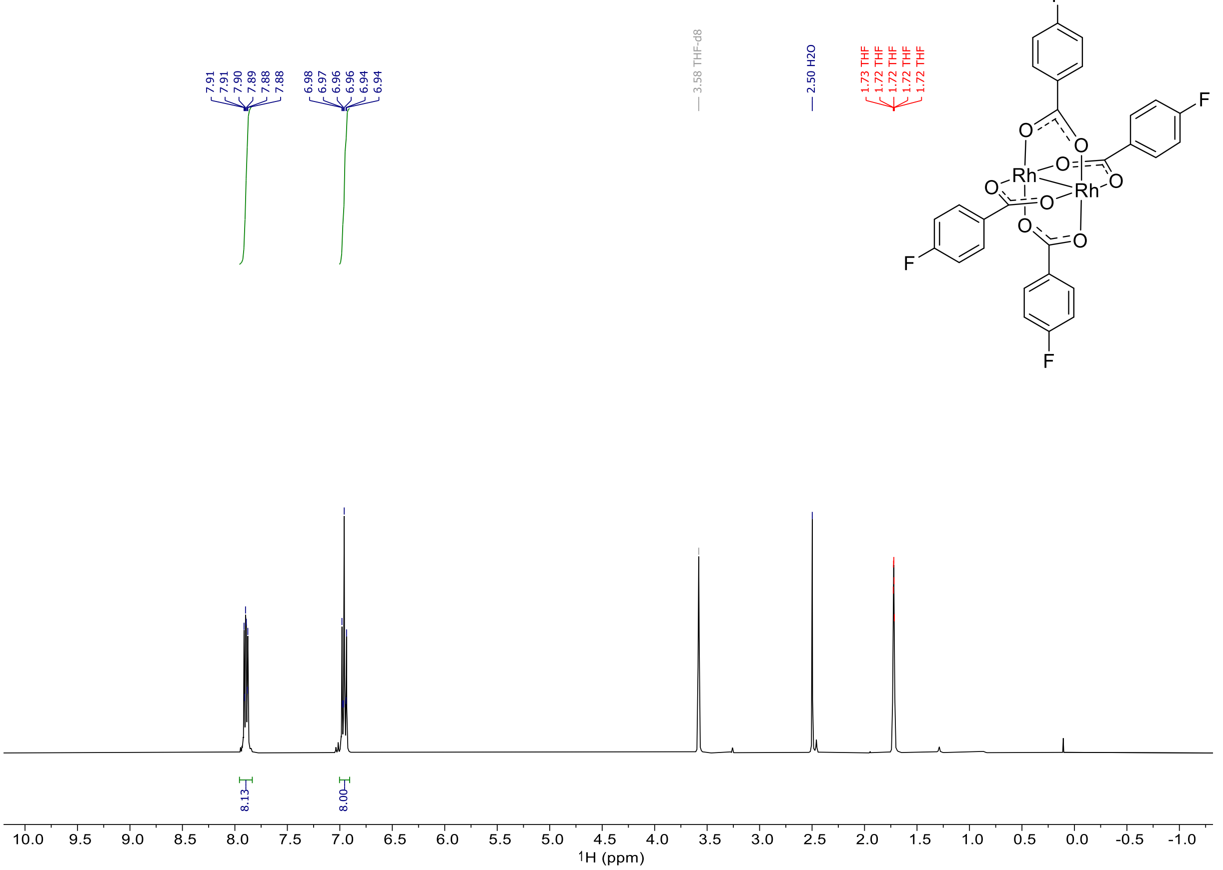


**$Rh_2$(*p*-$FC_6H_4COO$)$_4$ (10): H(C)Rh ([$D_8$]-THF)**

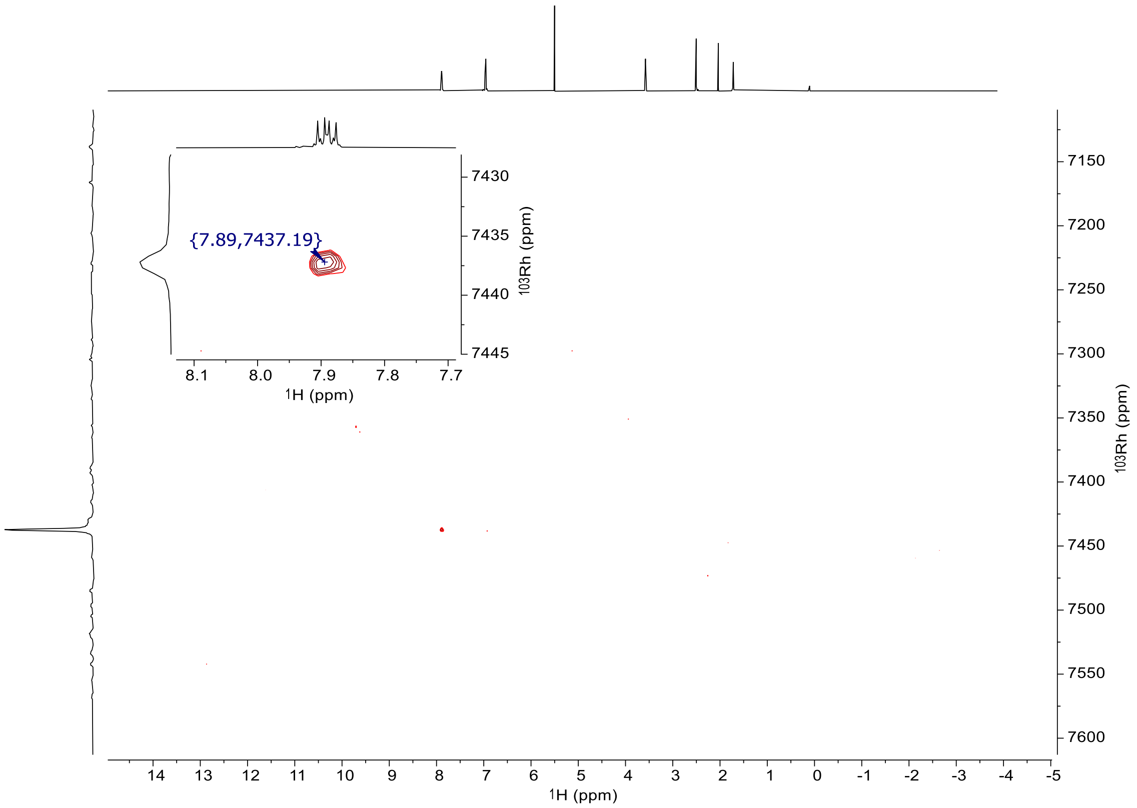

**$Rh_2$(*p*-$F_3CC_6H_4COO)_4$ (11): $^1$H-NMR (400 MHz, [$D_8$]-THF)**

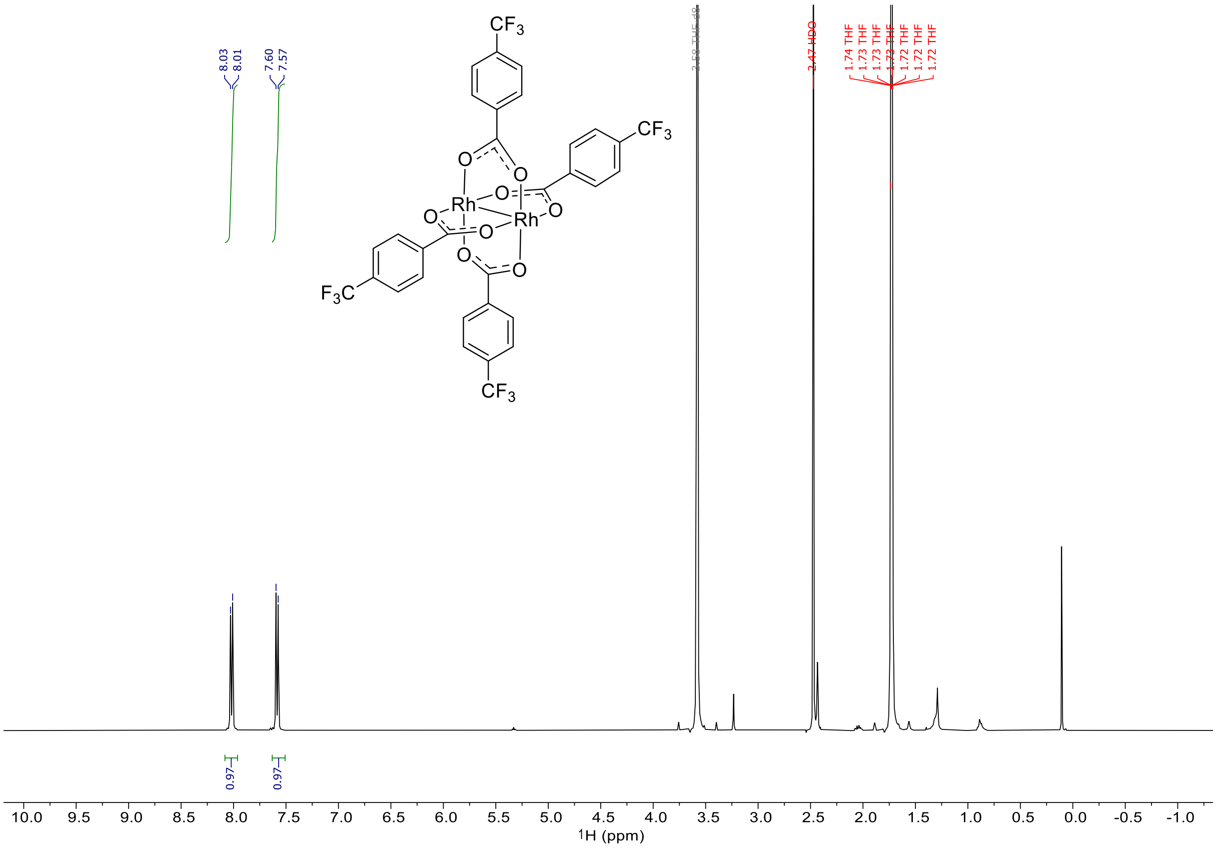

**$Rh_2$(*p*-$F_3CC_6H_4COO)_4$ (11): H(C)Rh ([$D_8$]-THF)**

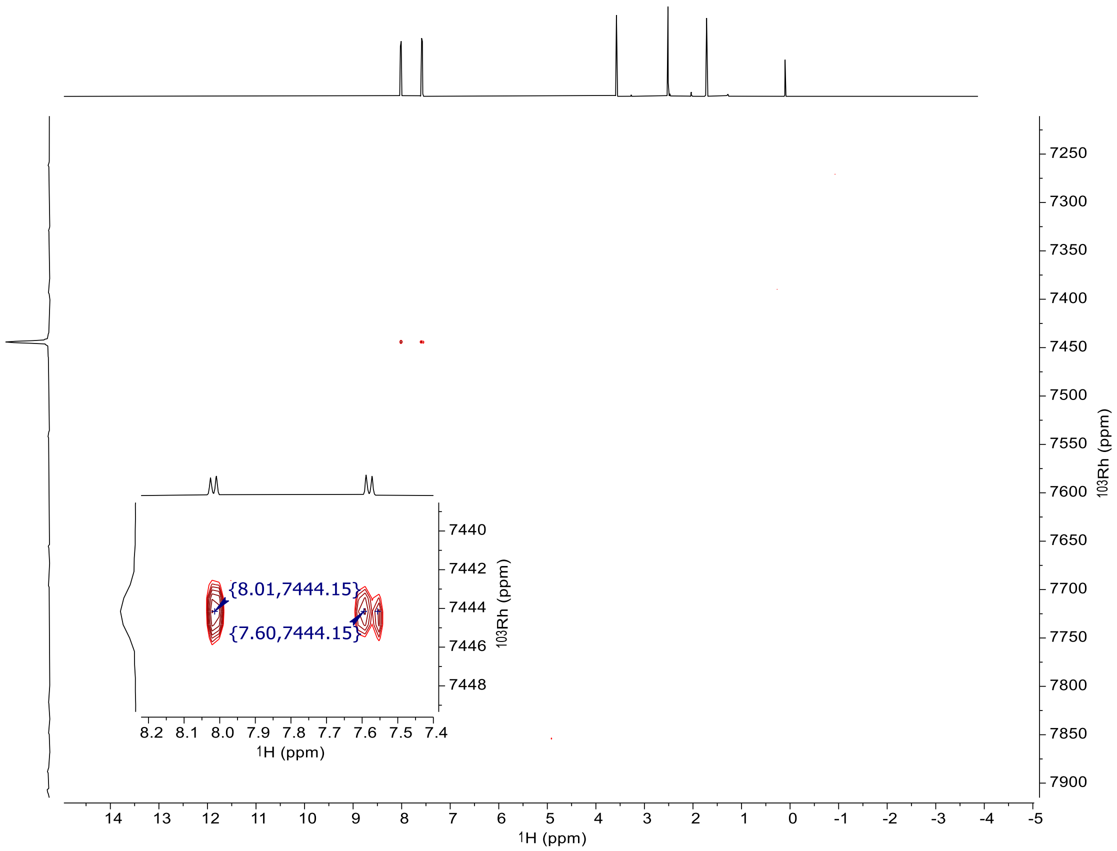

**$Rh_2(ClCH_2CO_2)_4$ (12): $^1$H-NMR (600 MHz, $CD_3CN$)**

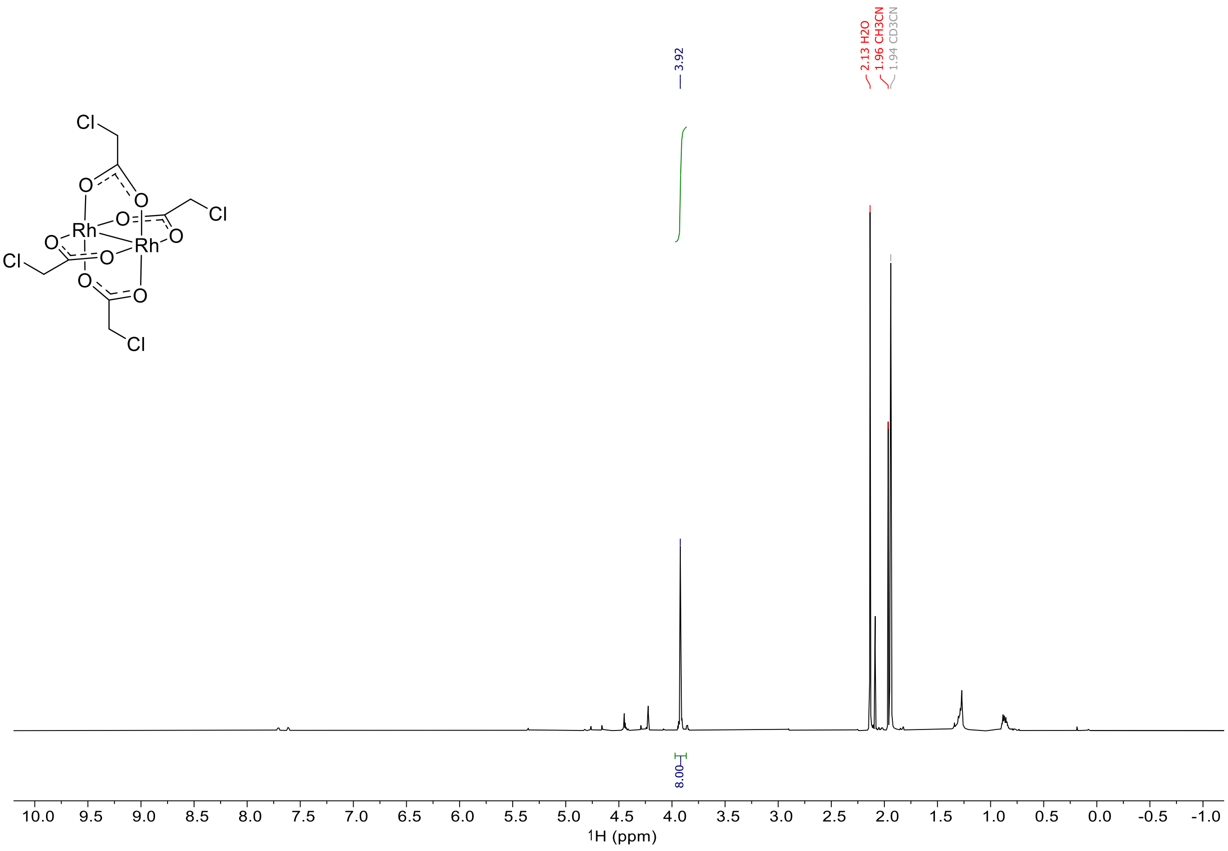


**$Rh_2(ClCH_2CO_2)_4$ (12): $^{13}$C-NMR (151 MHz, $CD_3CN$)**

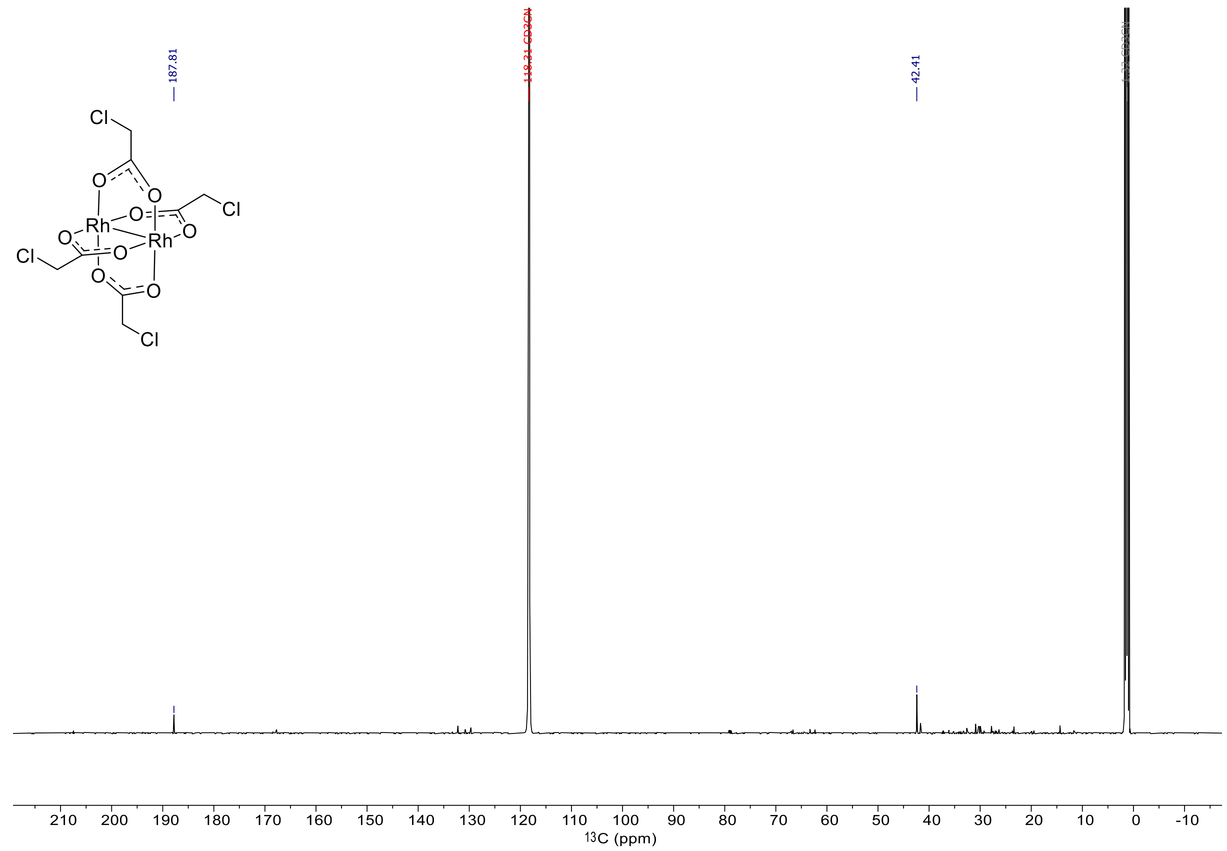

**$Rh_2(ClCH_2CO_2)_4$ (12): H(C)Rh ($CD_3CN$)**

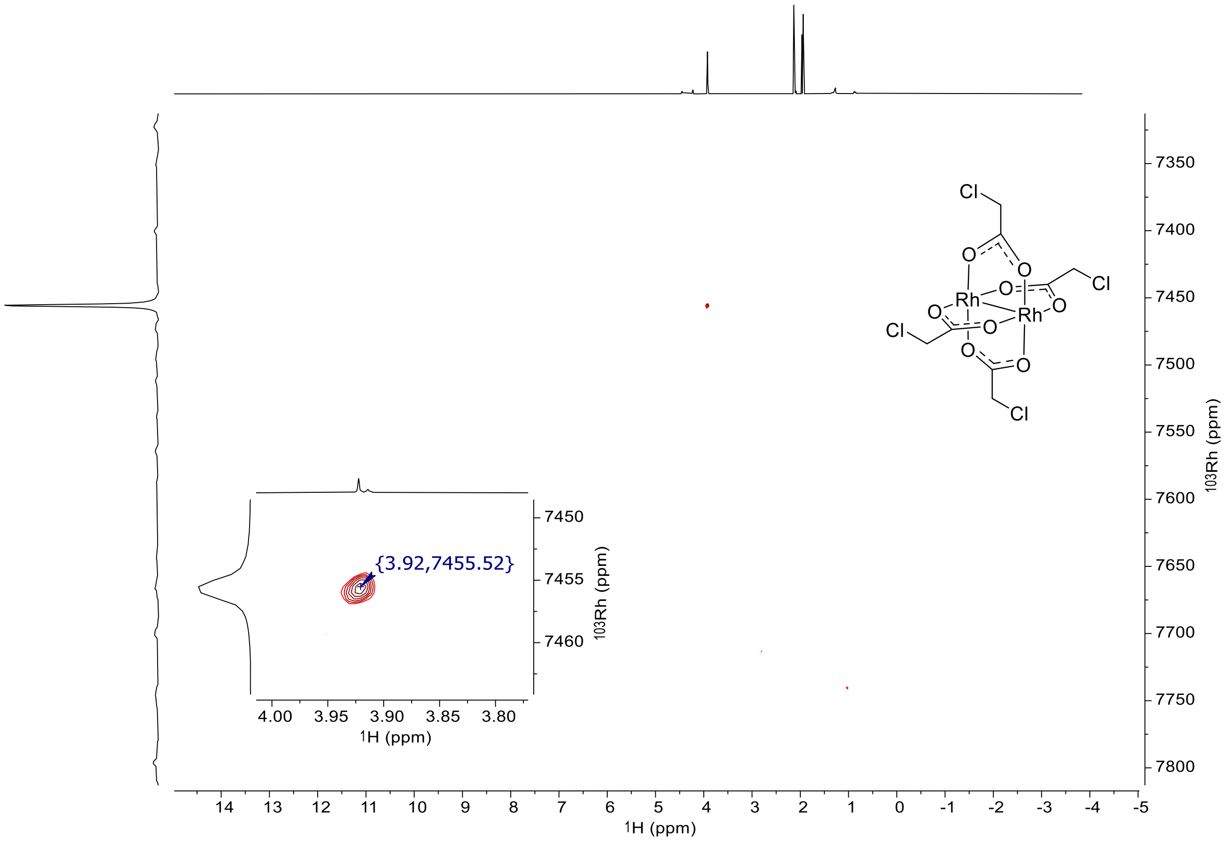

**Rh$_2$(triphenylacetate)$_4$ (13)**: **$^1$H-NMR (500 MHz, [D$_8$]-THF)**

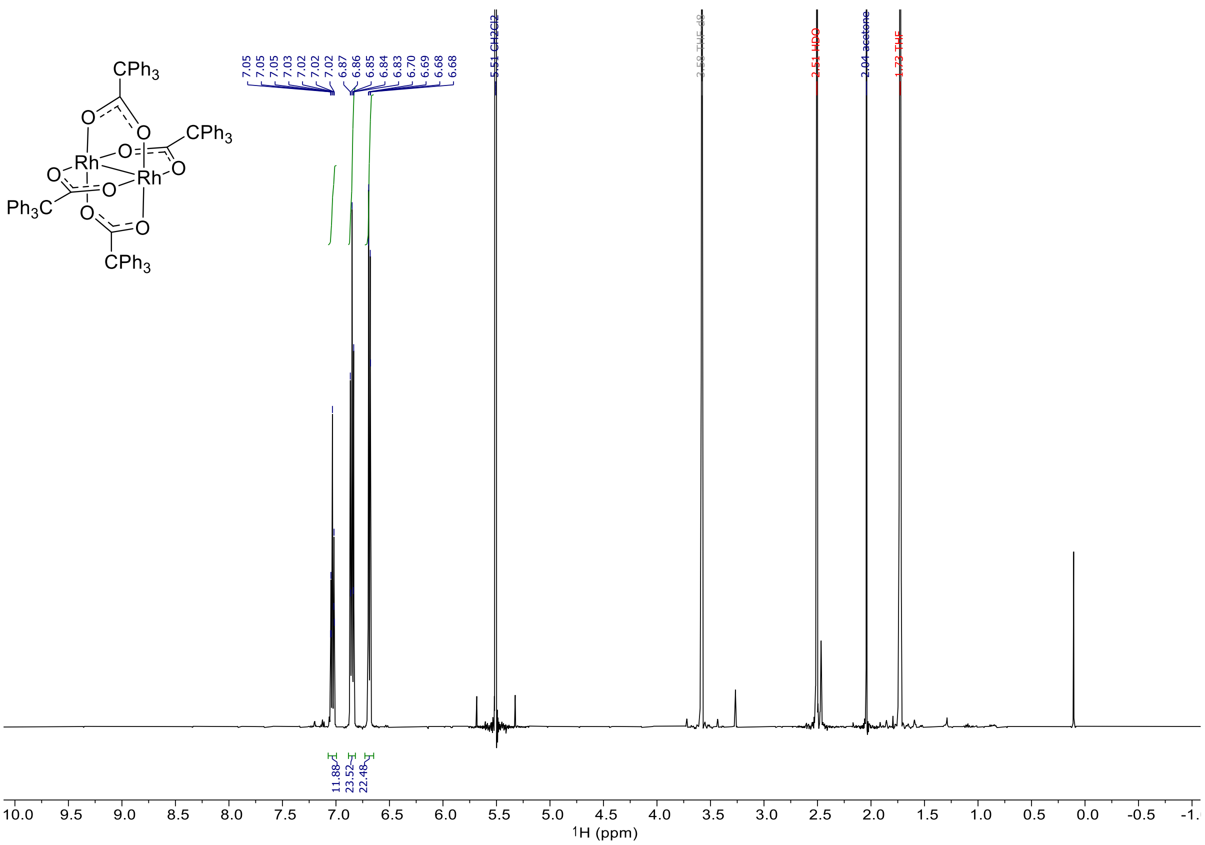


**Rh$_2$(triphenylacetate)$_4$ (13)**: **H(C)Rh ([D$_8$]-THF)**

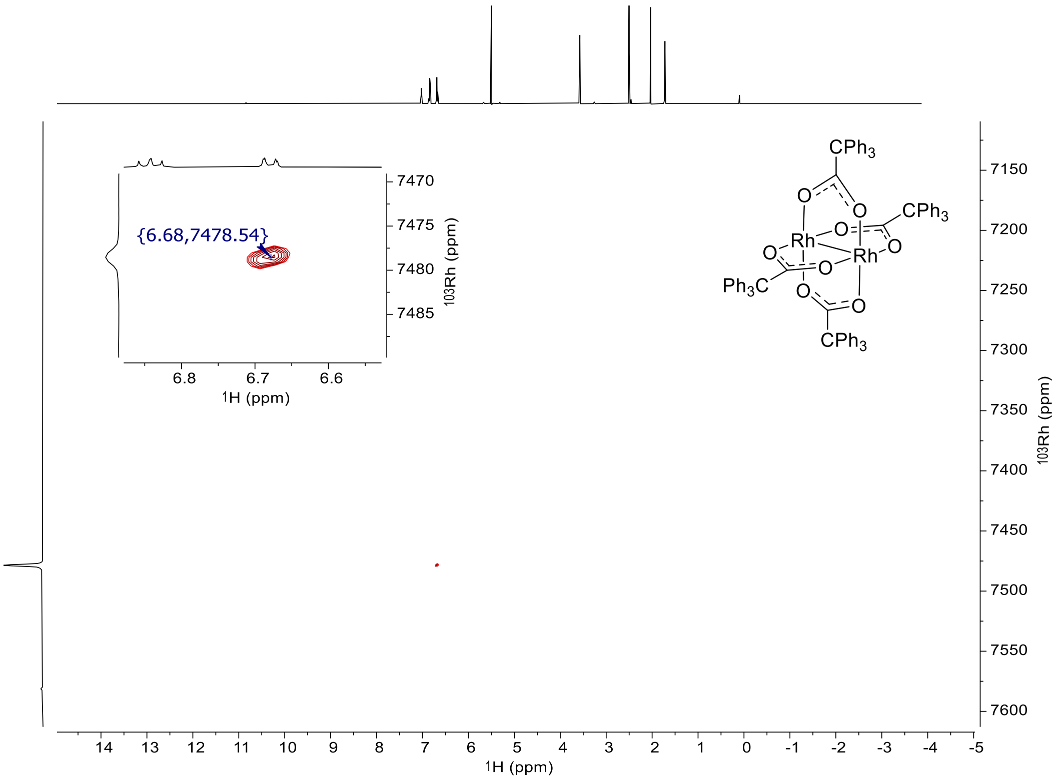

**Rh$_2$(esp)$_2$ (14): $^1$H-NMR (500 MHz, CD$_3$CN)**

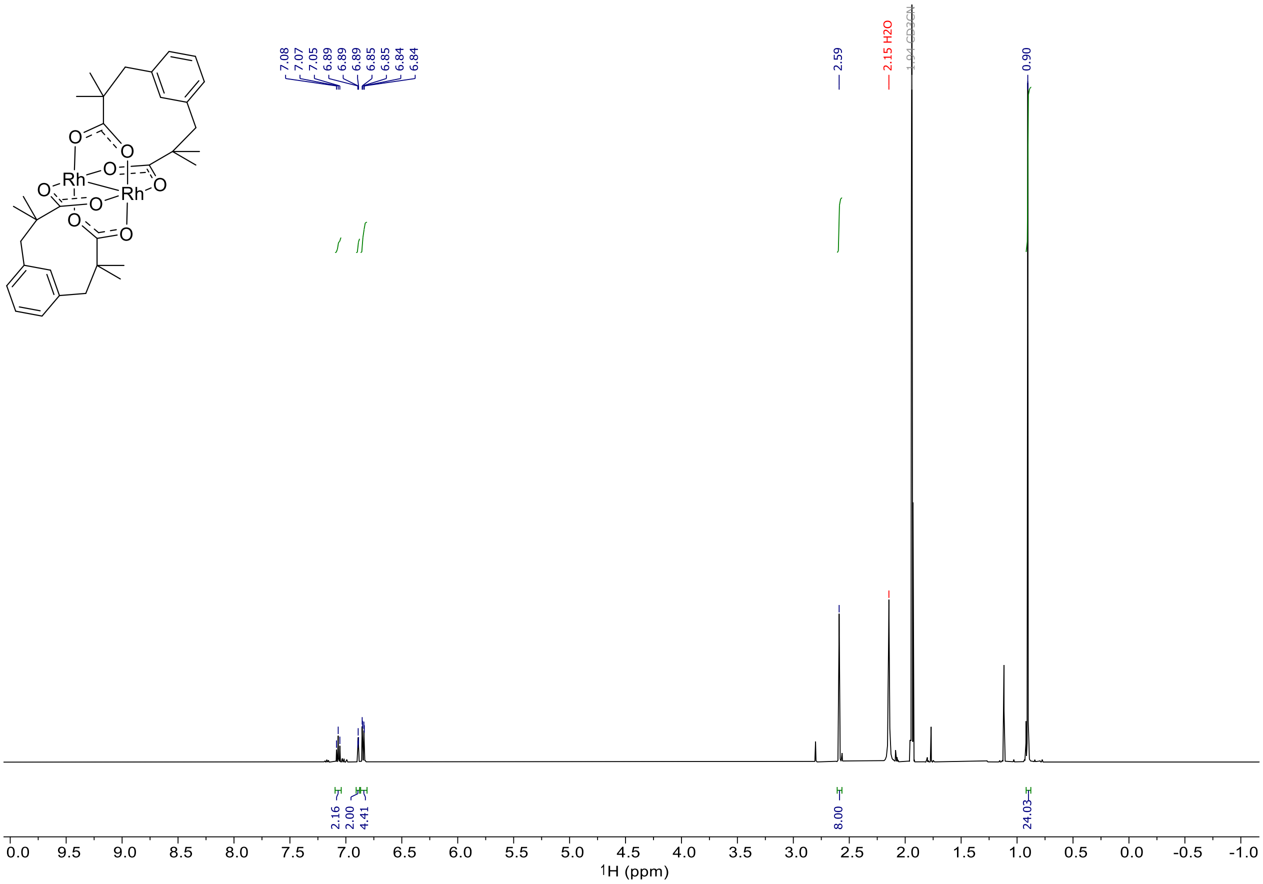

**Rh$_2$(esp)$_2$ (14): H(C)Rh (CD$_3$CN)**

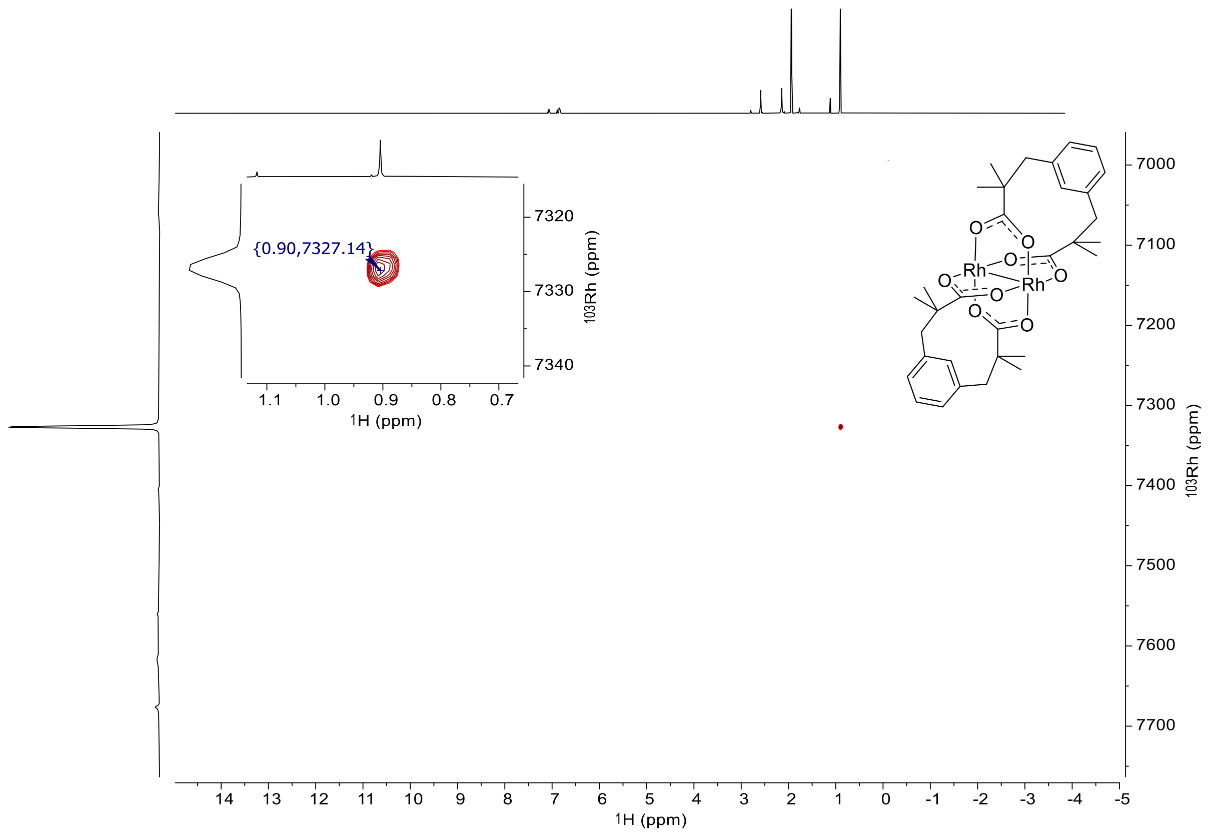

**Rh$_2$(*S*-PTTL)$_4$ (15): $^1$H-NMR (500 MHz, CD$_3$CN)**

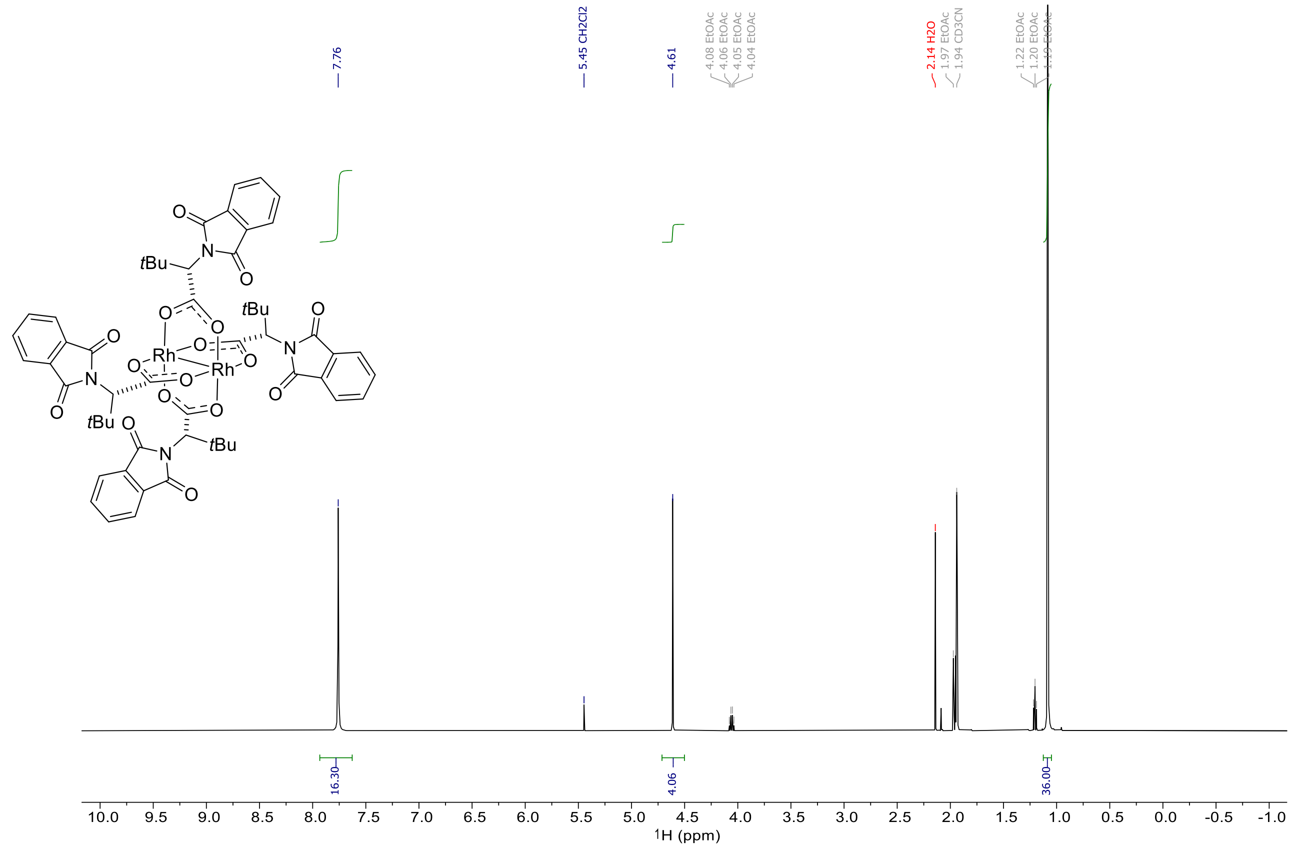


**Rh$_2$(*S*-PTTL)$_4$ (15): H(C)Rh (CD$_3$CN)**

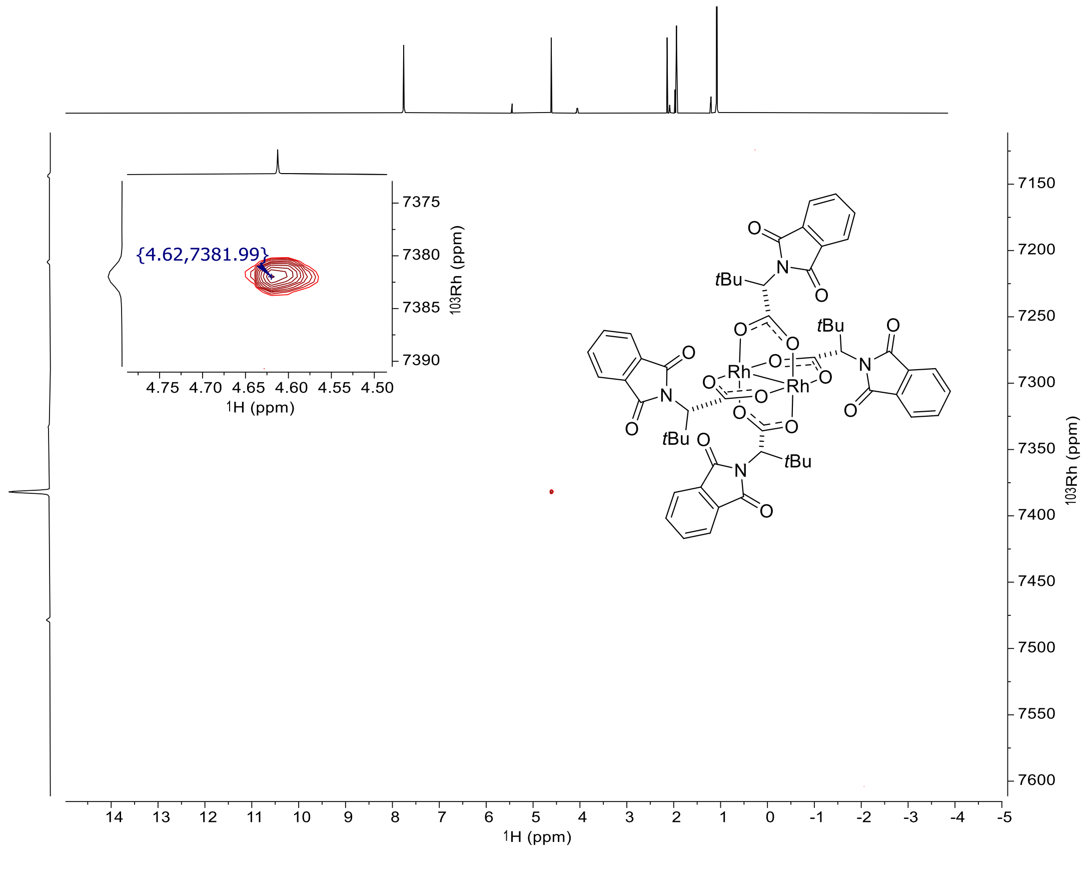

**$Rh_2(ACAM)(OPiv)_3$ (16): $^1$H-NMR (600 MHz, $CD_3CN$)**

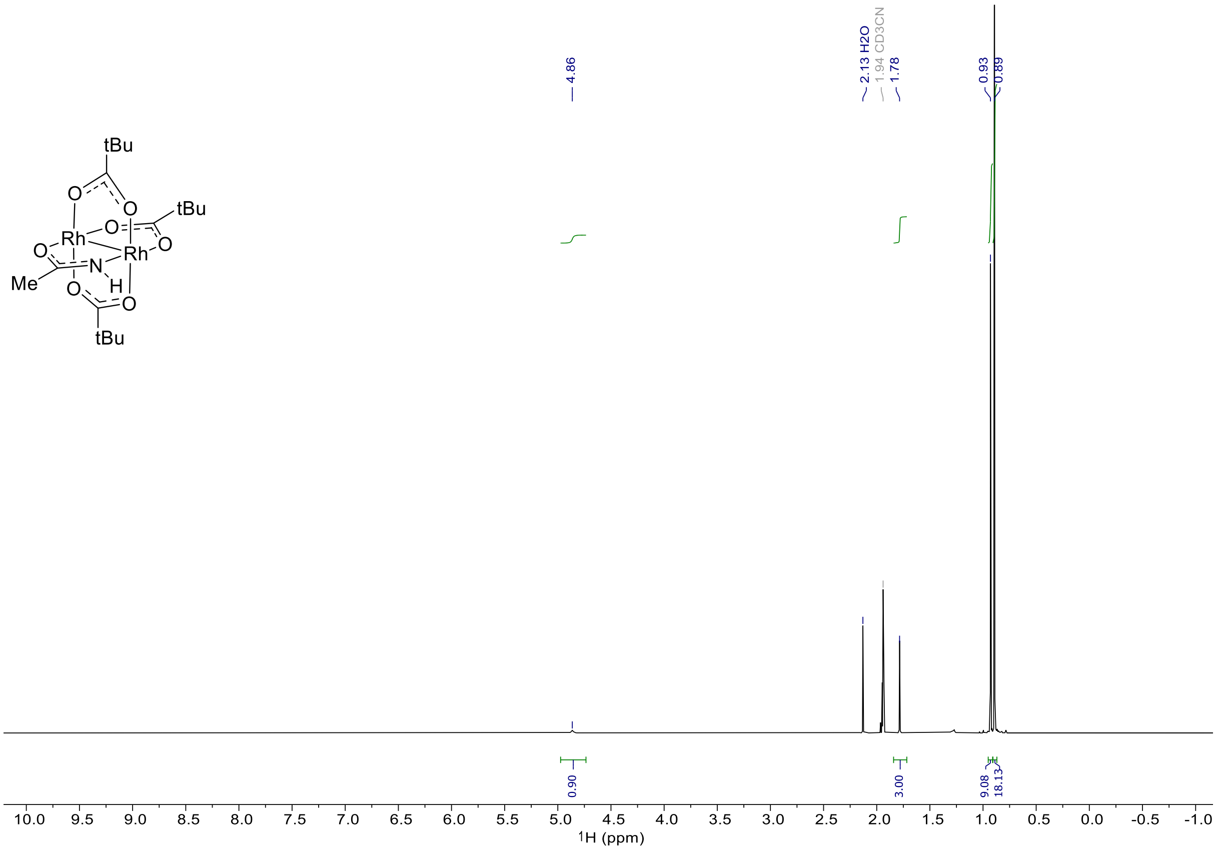


**$Rh_2(ACAM)(OPiv)_3$ (16): $^{13}C\{^1H\}$-NMR (151 MHz, $CD_3CN$)**

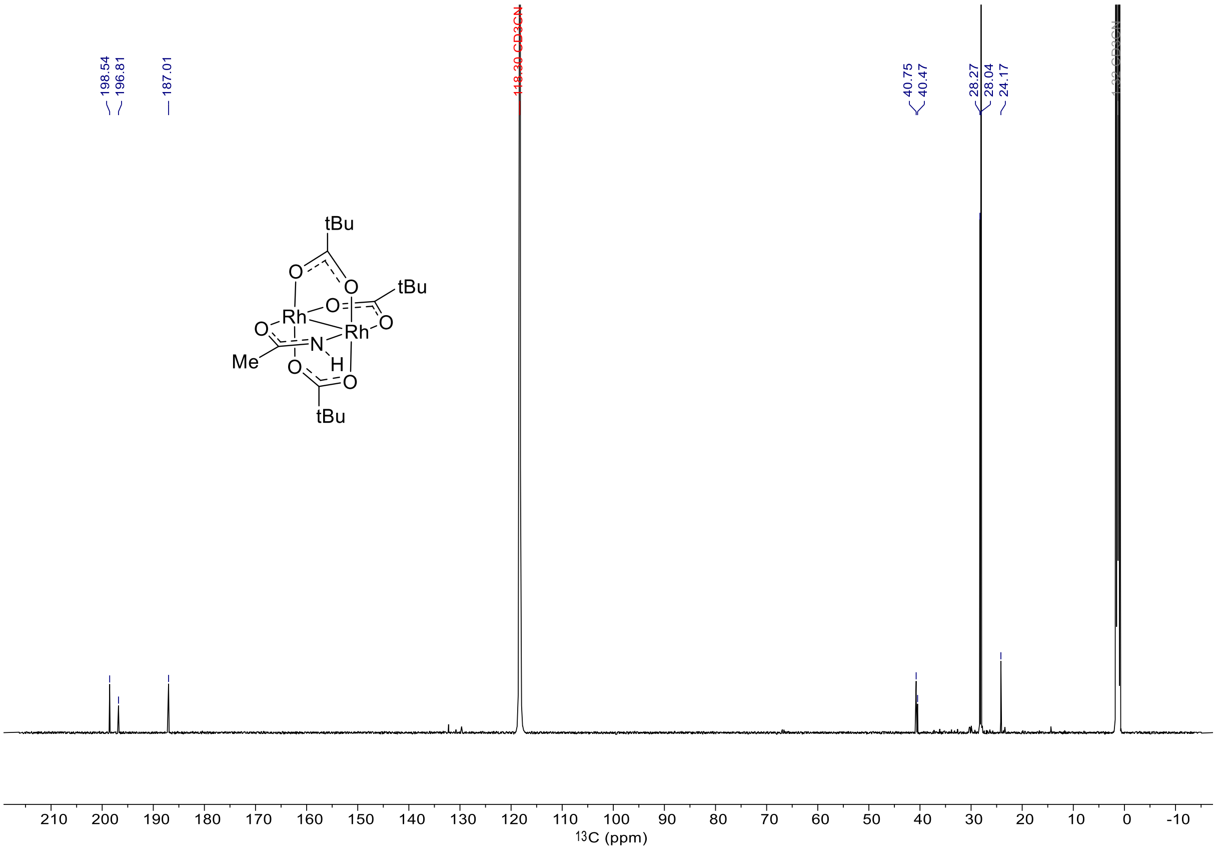

**$Rh_2$(ACAM)(OPiv)$_3$ (16): $^1$H-$^{15}$N-HMBC ($CD_3CN$)**

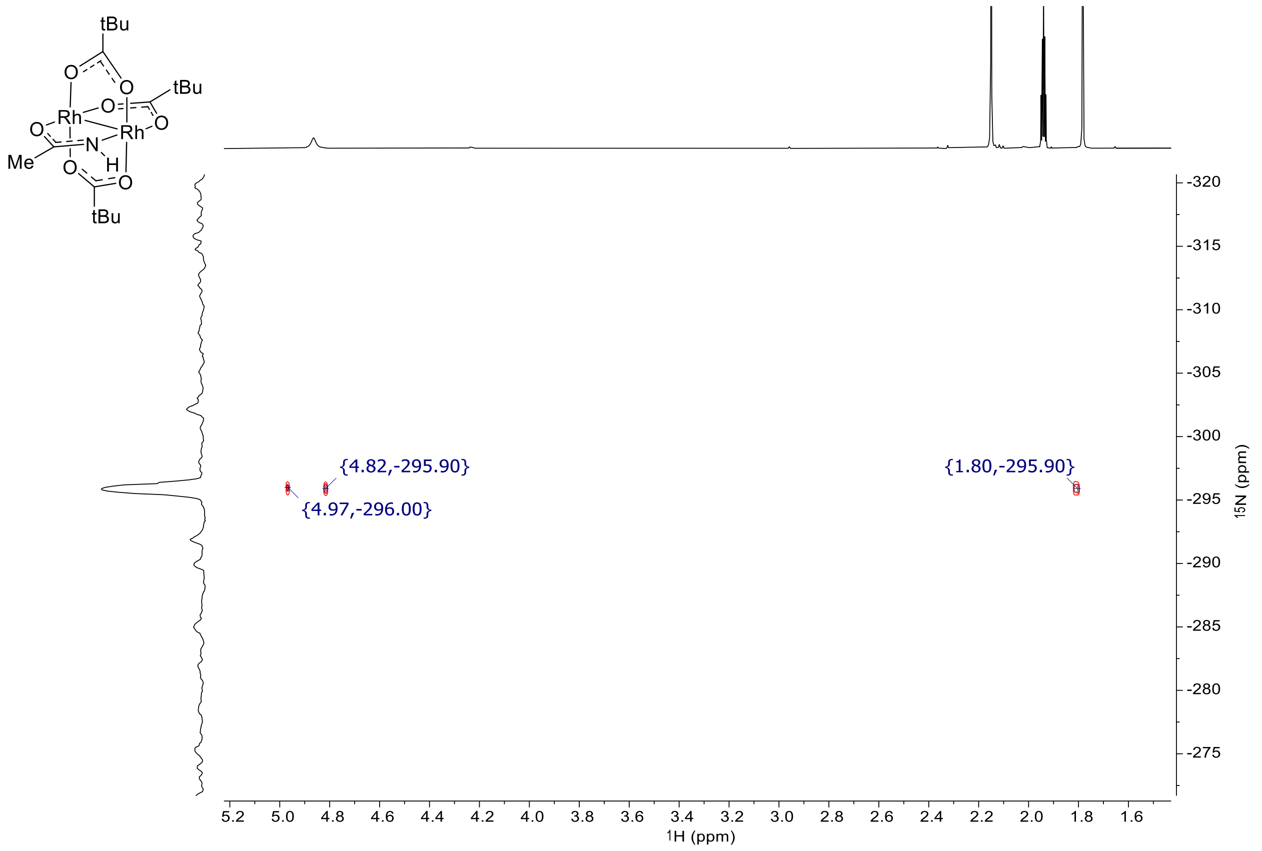


**$Rh_2$(ACAM)(OPiv)$_3$ (16): H(C)Rh ($CD_3CN$)**

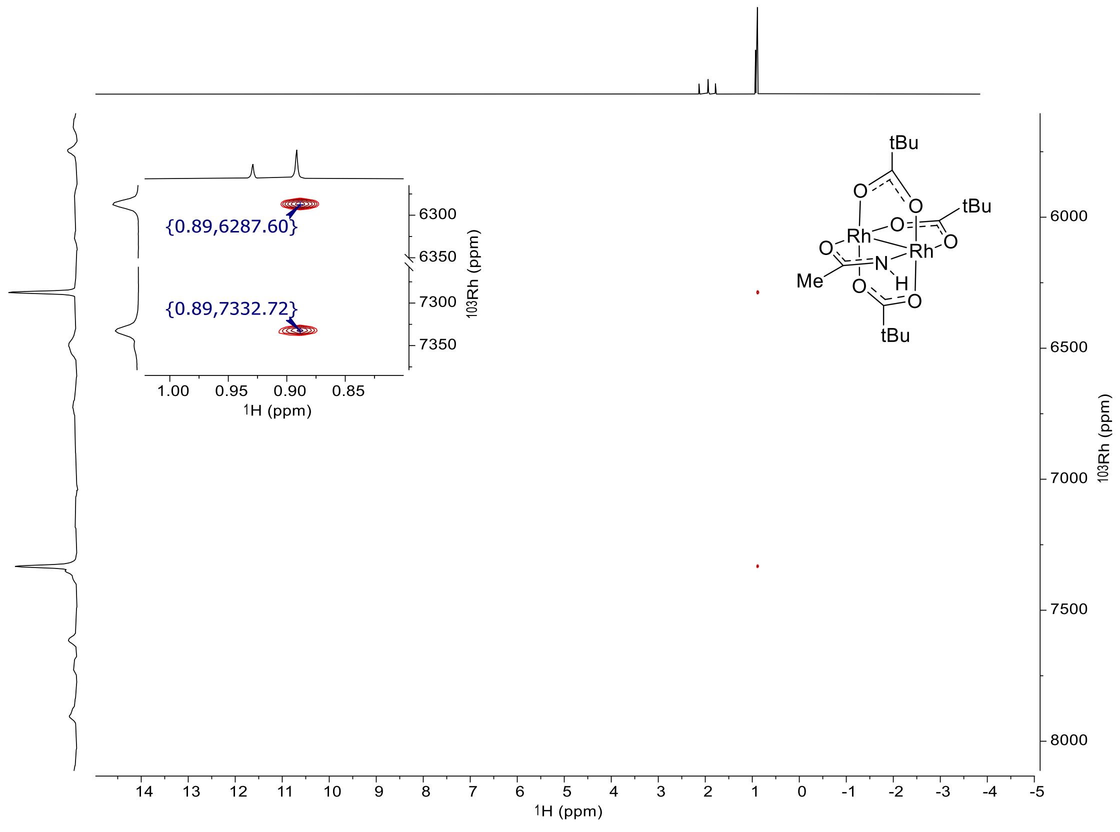

**$Rh_2(mhp)_4$ (17): H(C)Rh ($CD_2Cl_2/CD_3CN$, 50:50 *v/v*)**

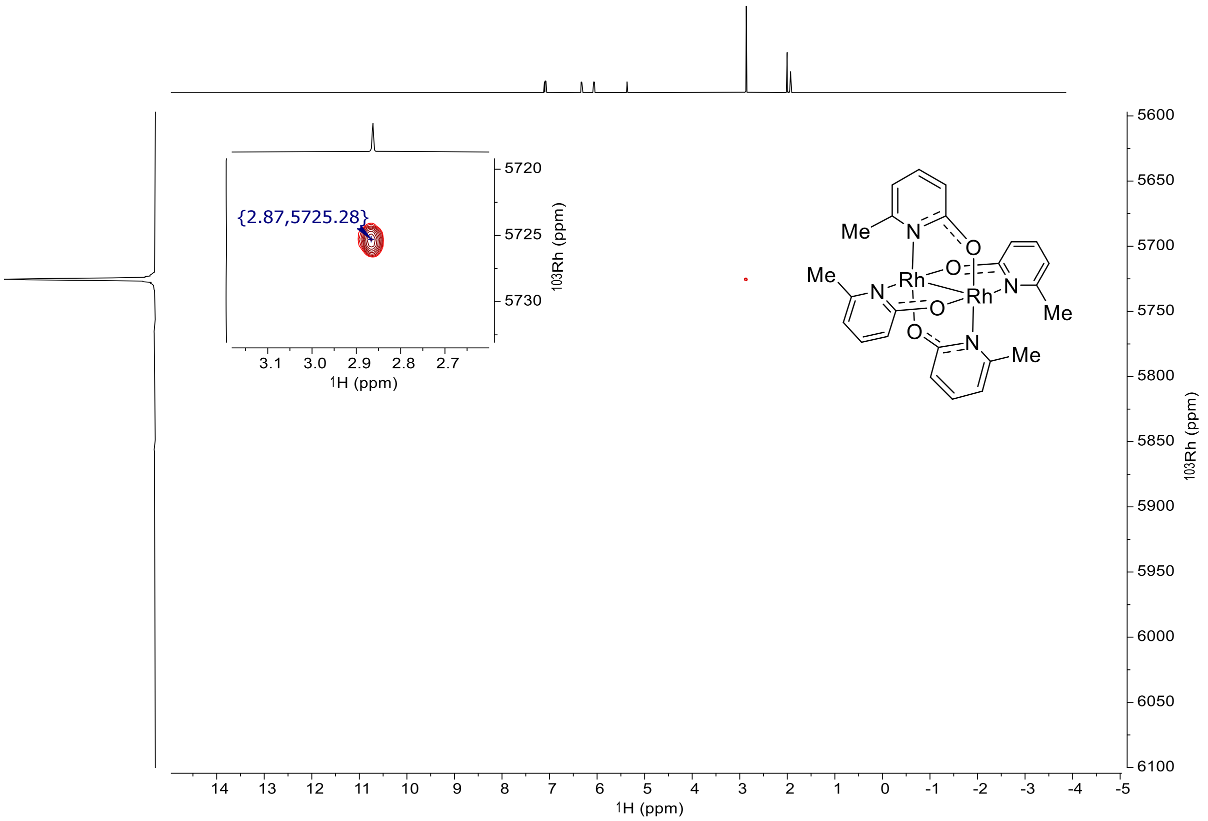


**$Rh_2(mhp)_4$ (17): $^1H$-$^{103}Rh$ HMBC ($CD_2Cl_2/CD_3CN$, 50:50 *v/v*)**

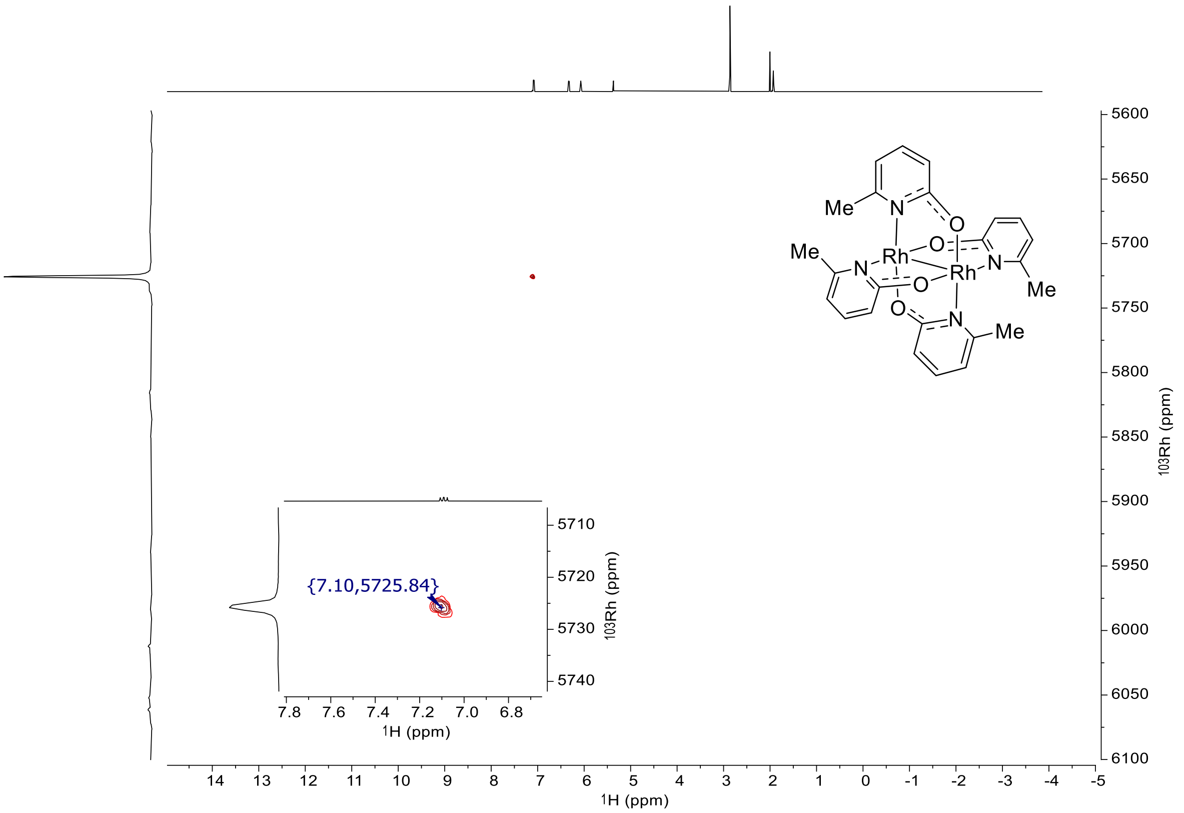

**Rh$_2$(*S*-BNAZ)$_4$ (18): $^1$H-NMR (400 MHz, CD$_3$CN)**

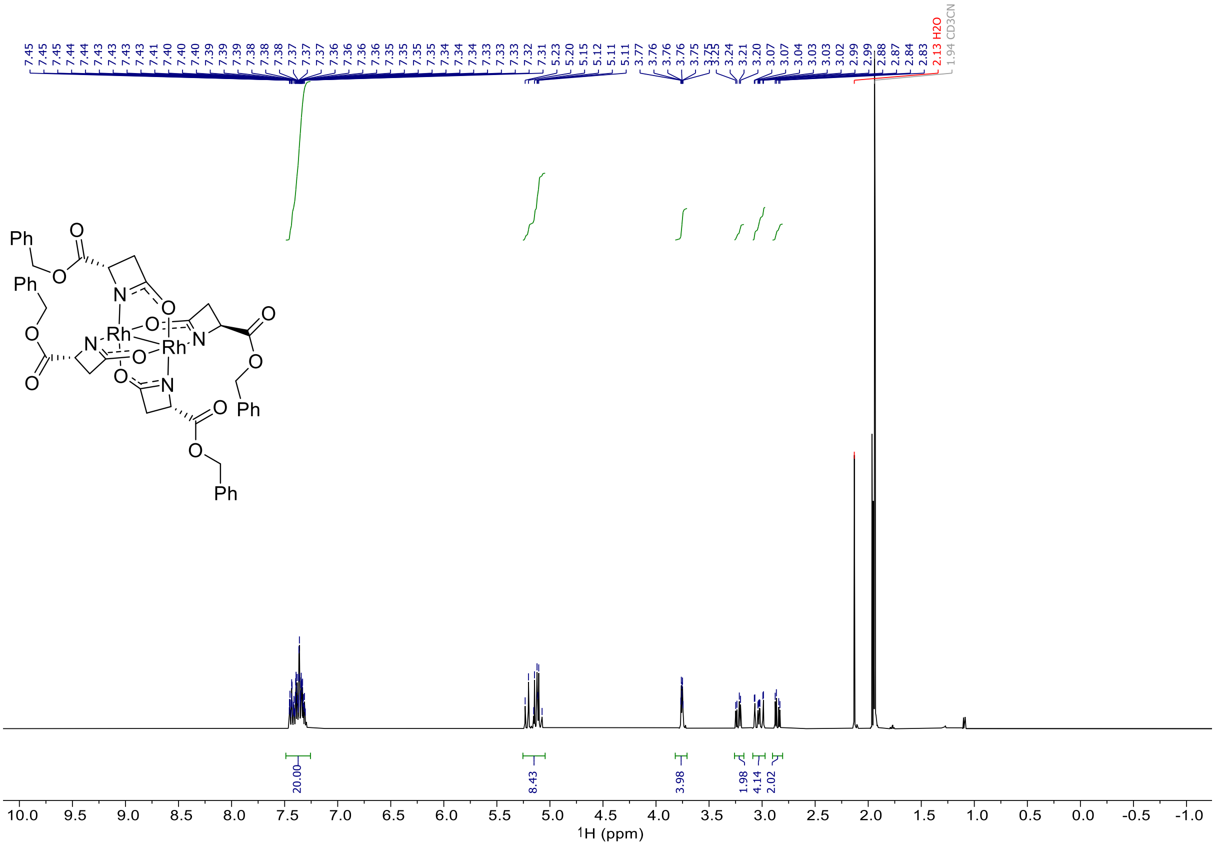


**Rh$_2$(*S*-BNAZ)$_4$ (18): $^{13}$C{$^1$H}-NMR (101 MHz, CD$_3$CN)**

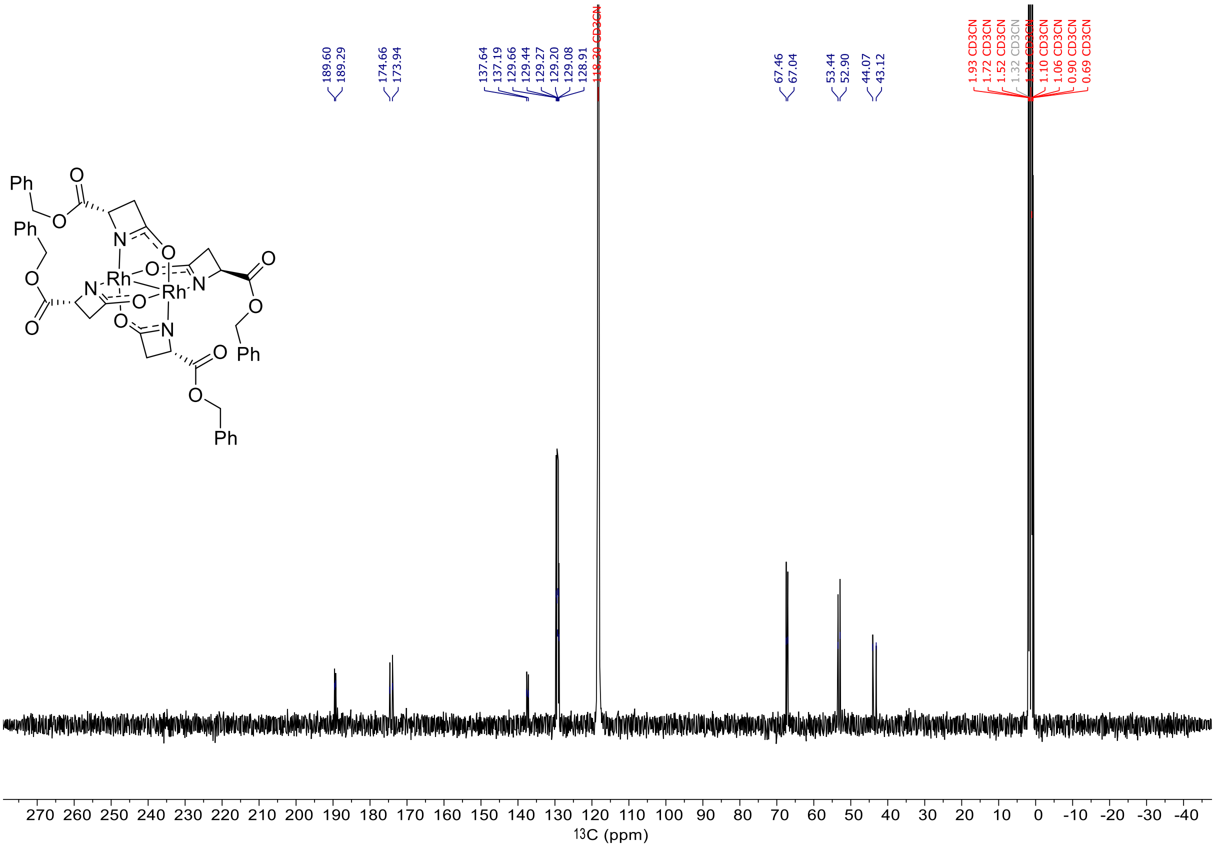

**$Rh_2$(*S*-BNAZ)$_4$ (18): $^1$H-$^{103}$Rh-HMBC ($CD_3CN$)**

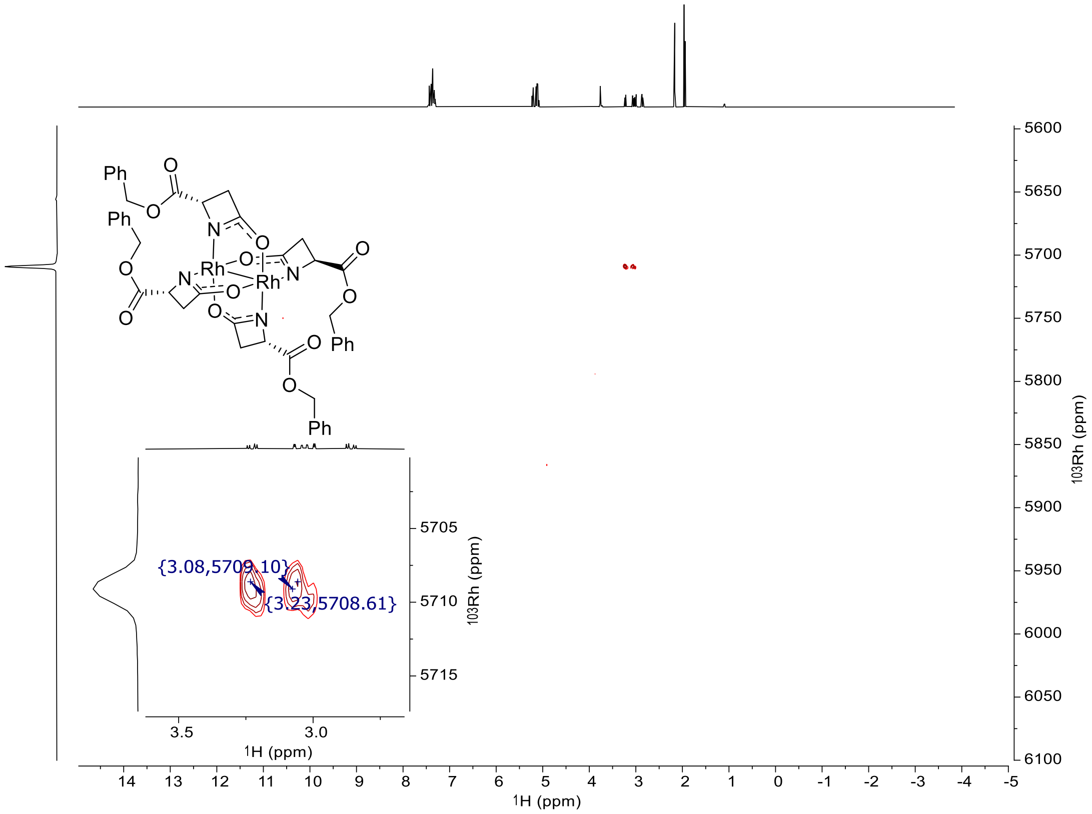

**[BiRh(OTfa)$_4$]·EtOAc (19): $^1$H-NMR (500 MHz, $CD_3CN$)**

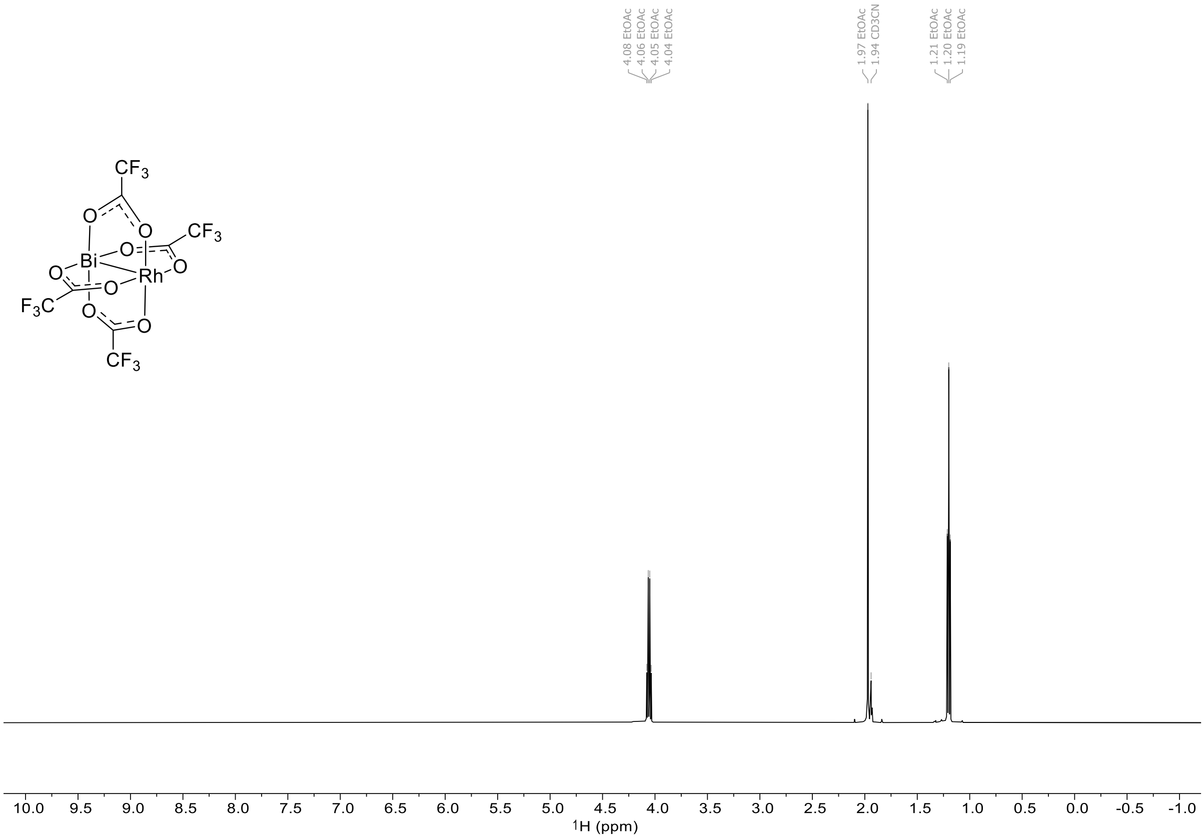


**[BiRh(OTfa)$_4$]·EtOAc (19): $^{19}$F-NMR (470 MHz, $CD_3CN$)**

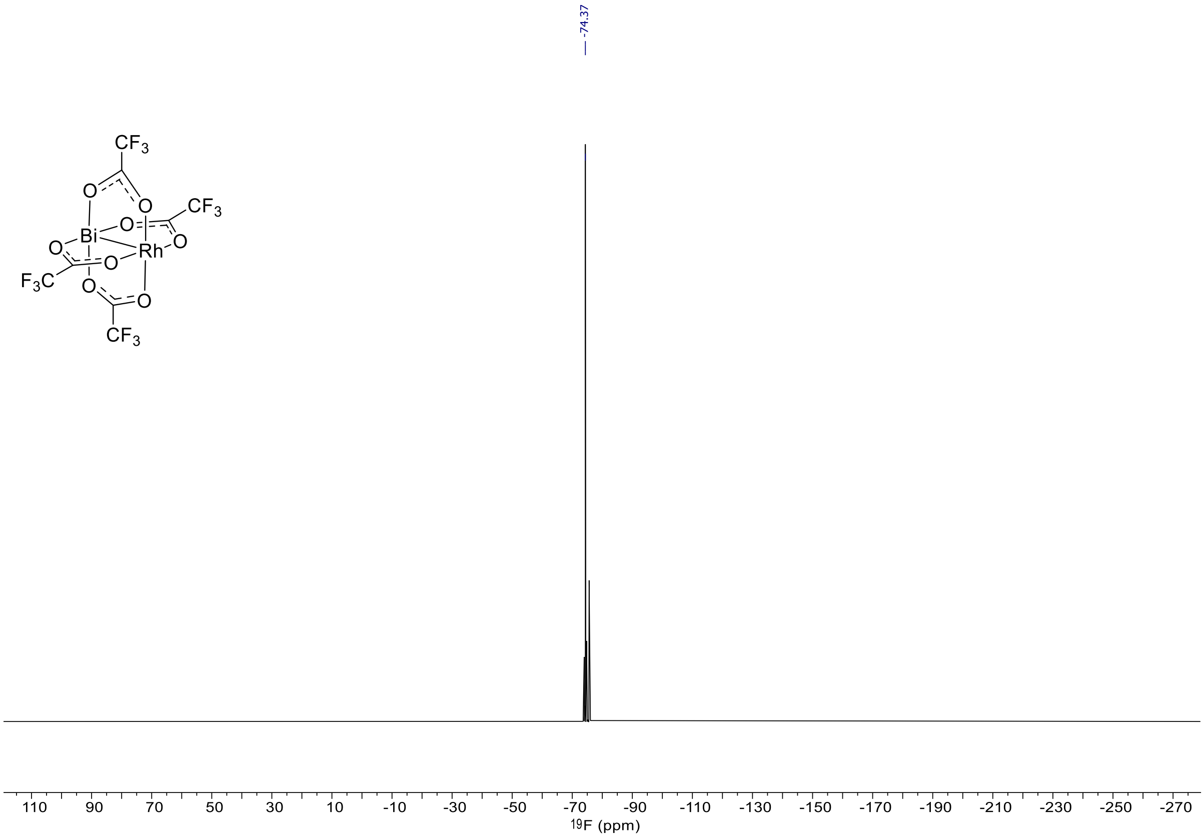

**[BiRh(OTfa)$_4$]·EtOAc (19): $^{103}$Rh-NMR (15.9 MHz, $CD_3CN$)**

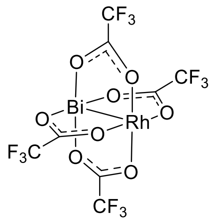

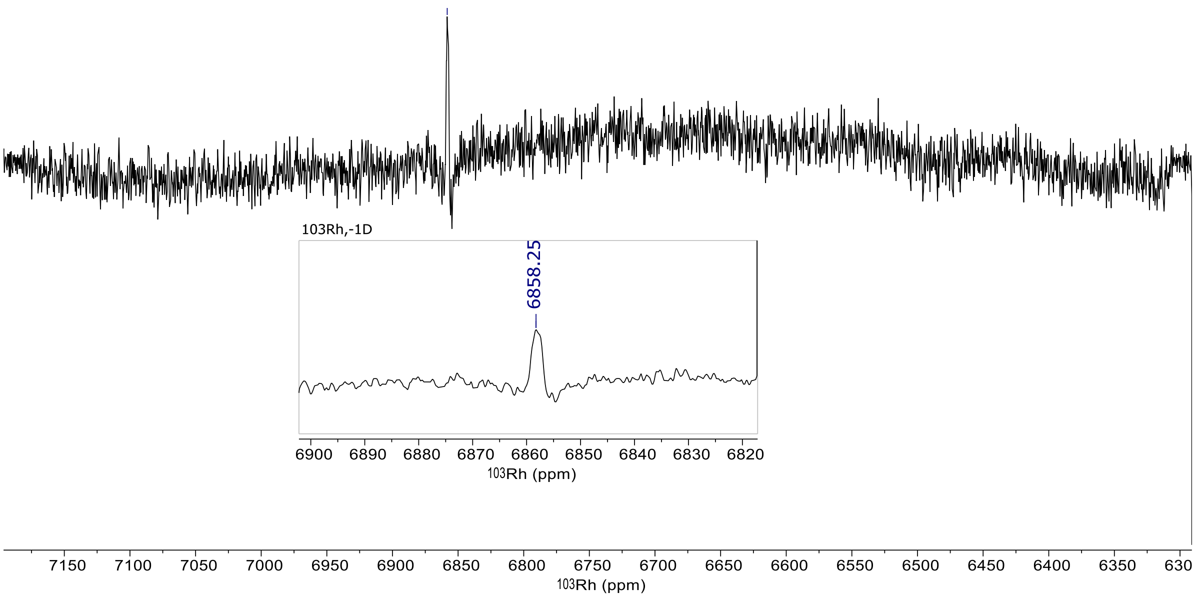

## BiRh(OAc)$_4$ (20): $^1$H-NMR (400 MHz, $CD_3CN$)

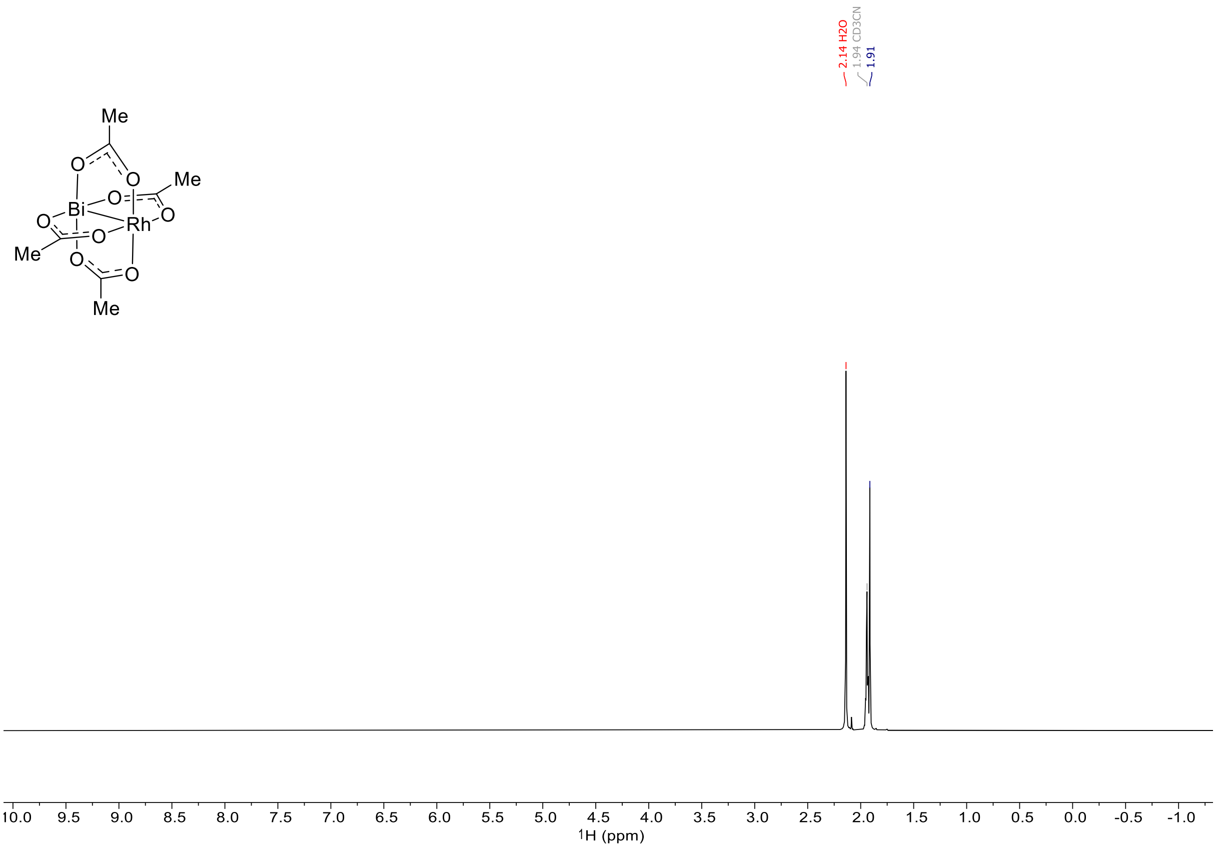


## BiRh(OAc)$_4$ (20): $^{13}$C{$^1$H}-NMR (151 MHz, $CD_3CN$)

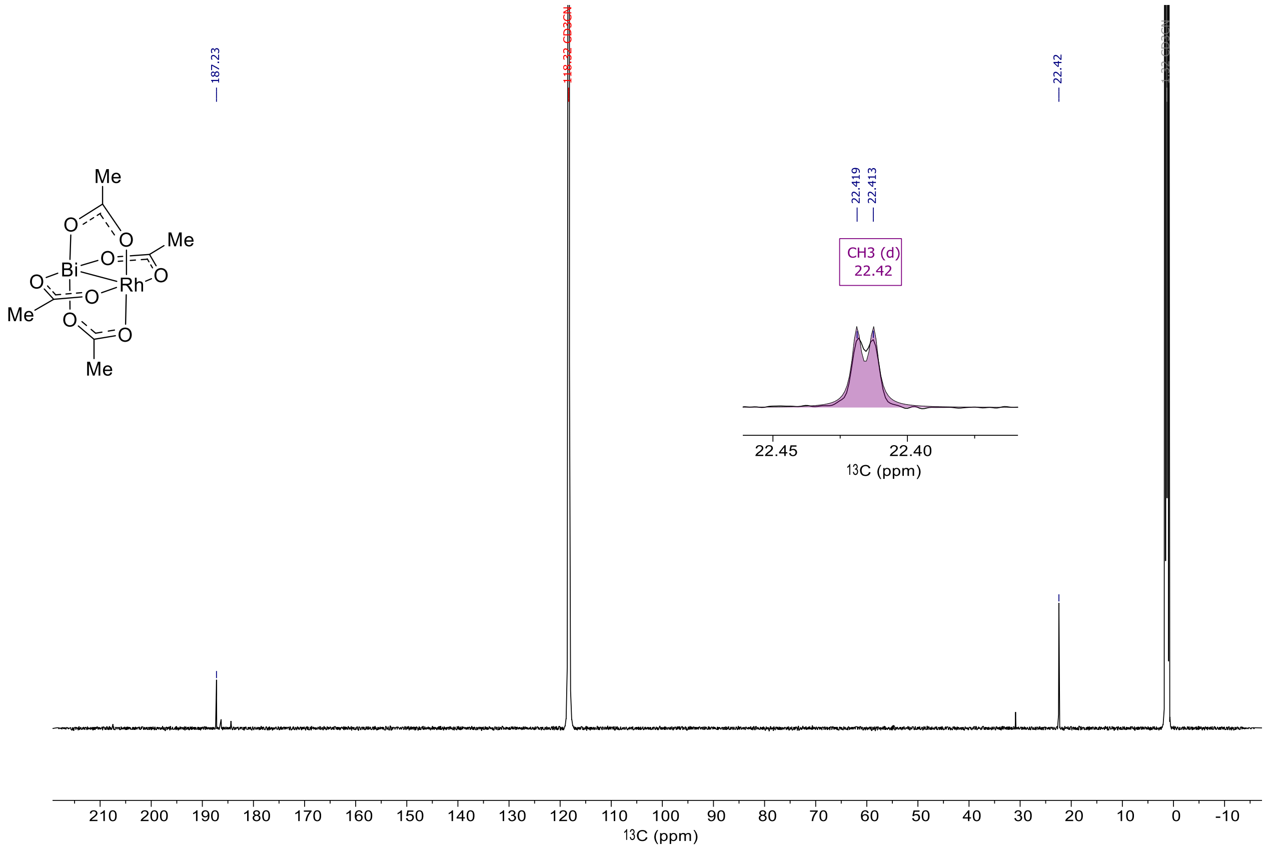

**BiRh(OAc)$_4$ (20): H(C)Rh (CD$_3$CN)**

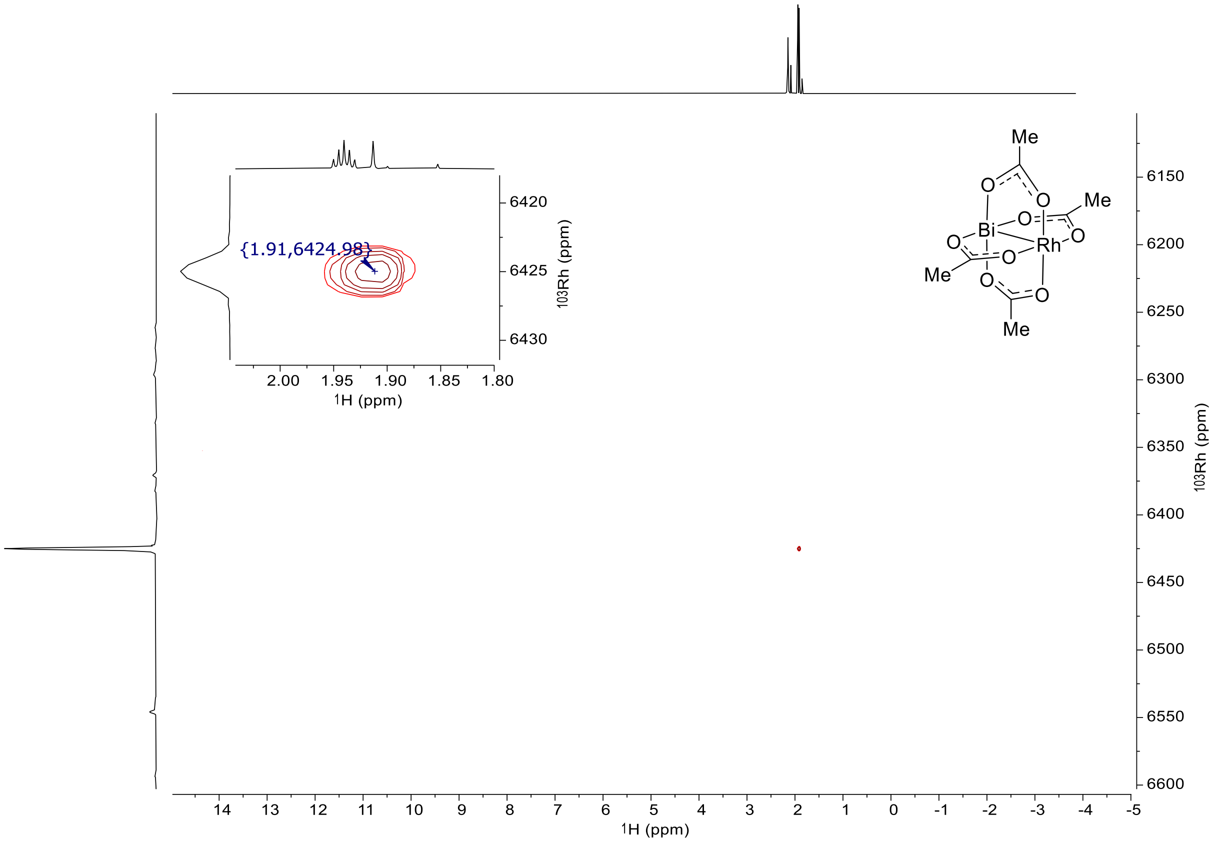

**BiRh(OAc-2-$^{13}$C) (21-2-$^{13}$C): $^{1}$H-NMR (500 MHz, $CD_3CN$)**

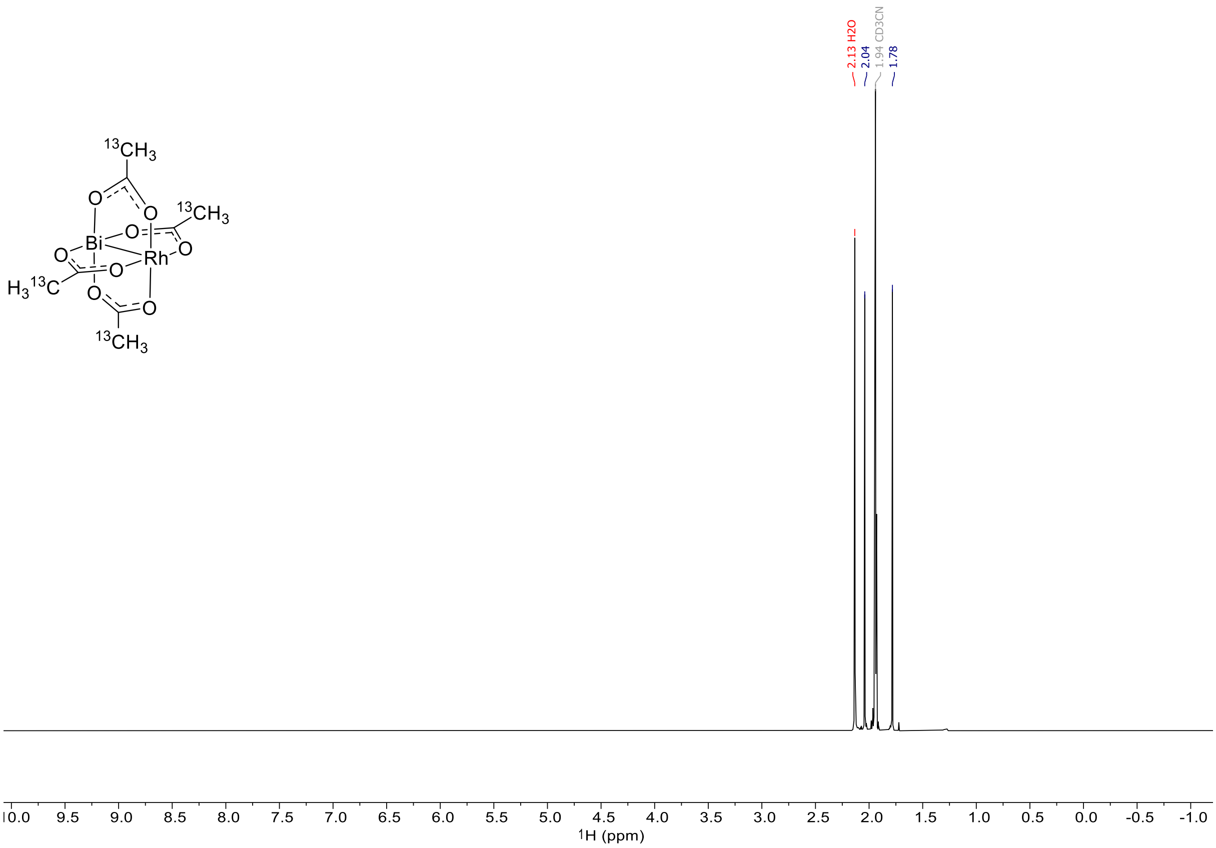


**BiRh(OAc-2-$^{13}$C) (21-2-$^{13}$C): $^{13}$C{$^{1}$H}-NMR (101 MHz, $CD_3CN$)**

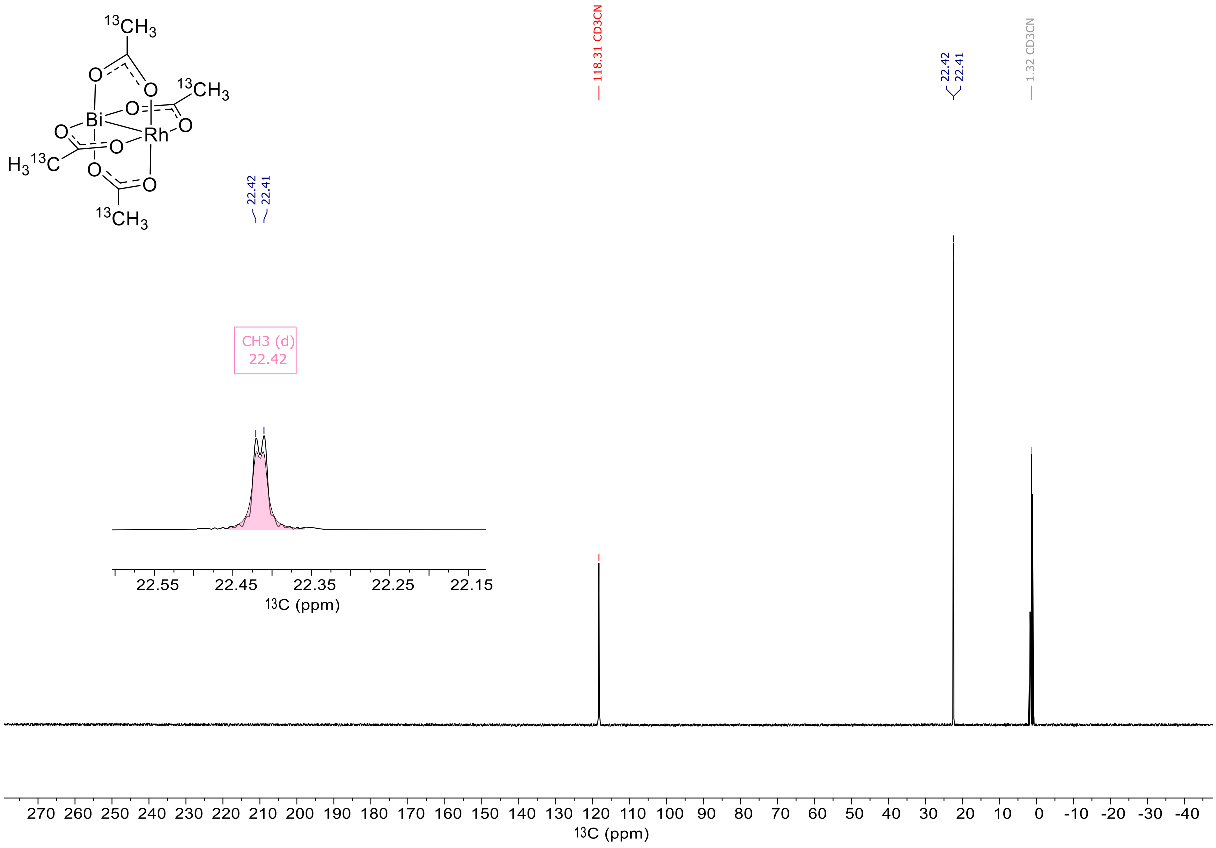

**BiRh(HCO$_2$)$_4$ (21): $^1$H-NMR (400 MHz, CD$_3$CN)**

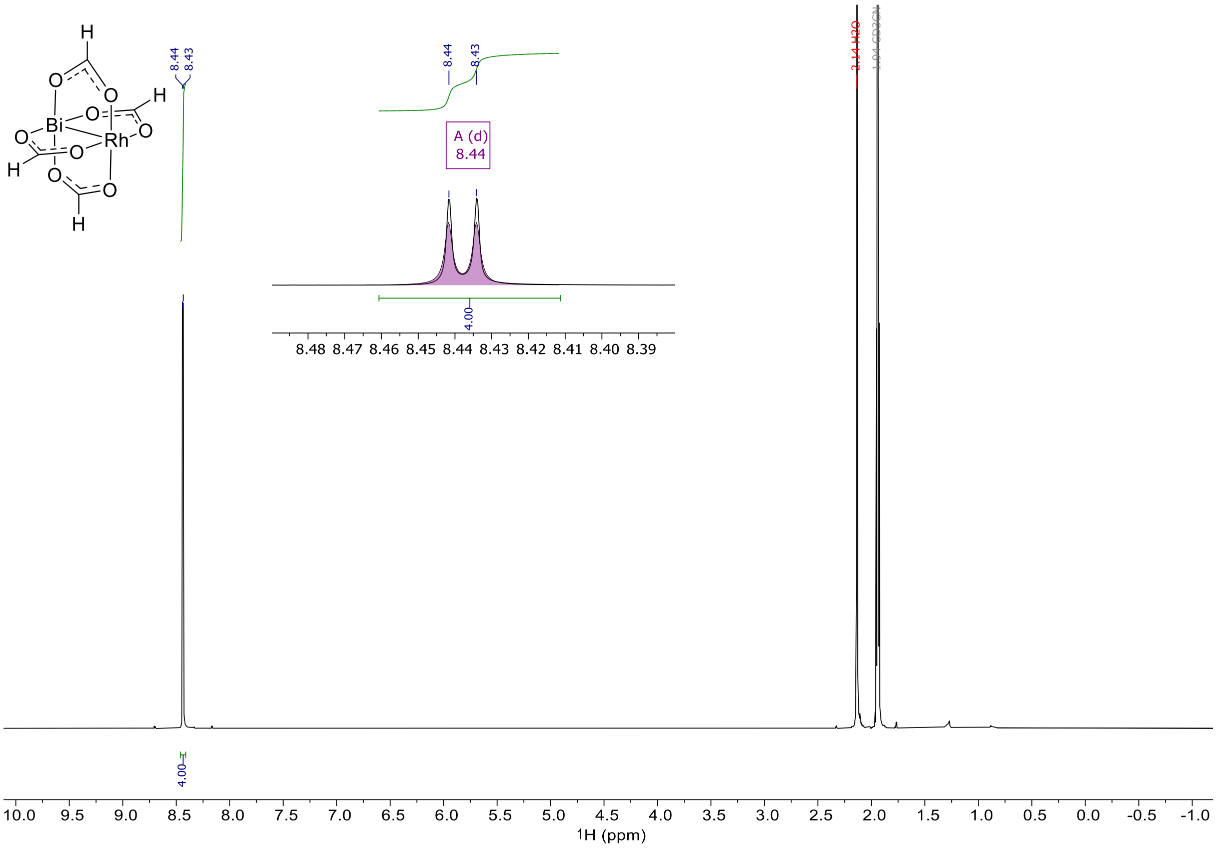


**BiRh(HCO$_2$)$_4$ (21): $^{13}$C{$^1$H}-NMR (101 MHz, CD$_3$CN)**

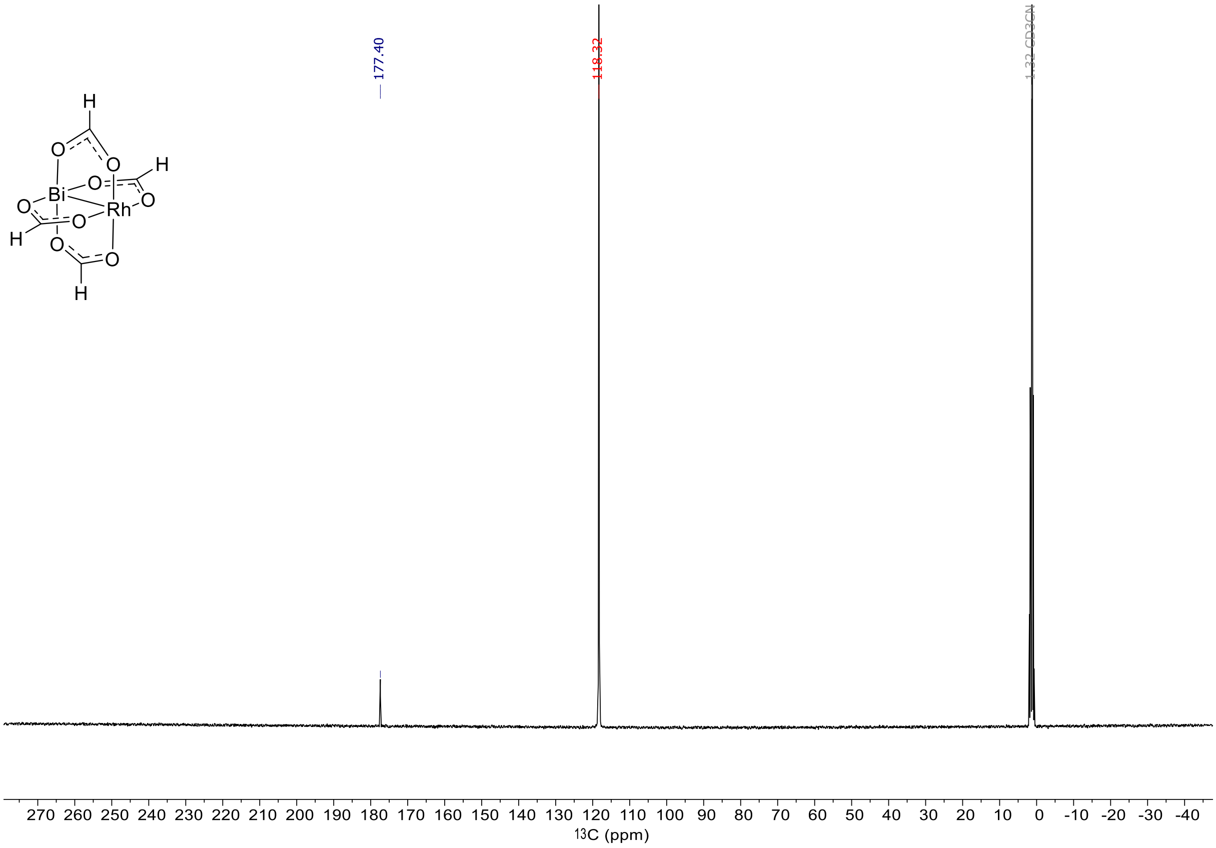

**BiRh(HCO$_2$)$_4$ (21): $^1$H-$^{13}$C-*edited*-HSQC (CD$_3$CN)**

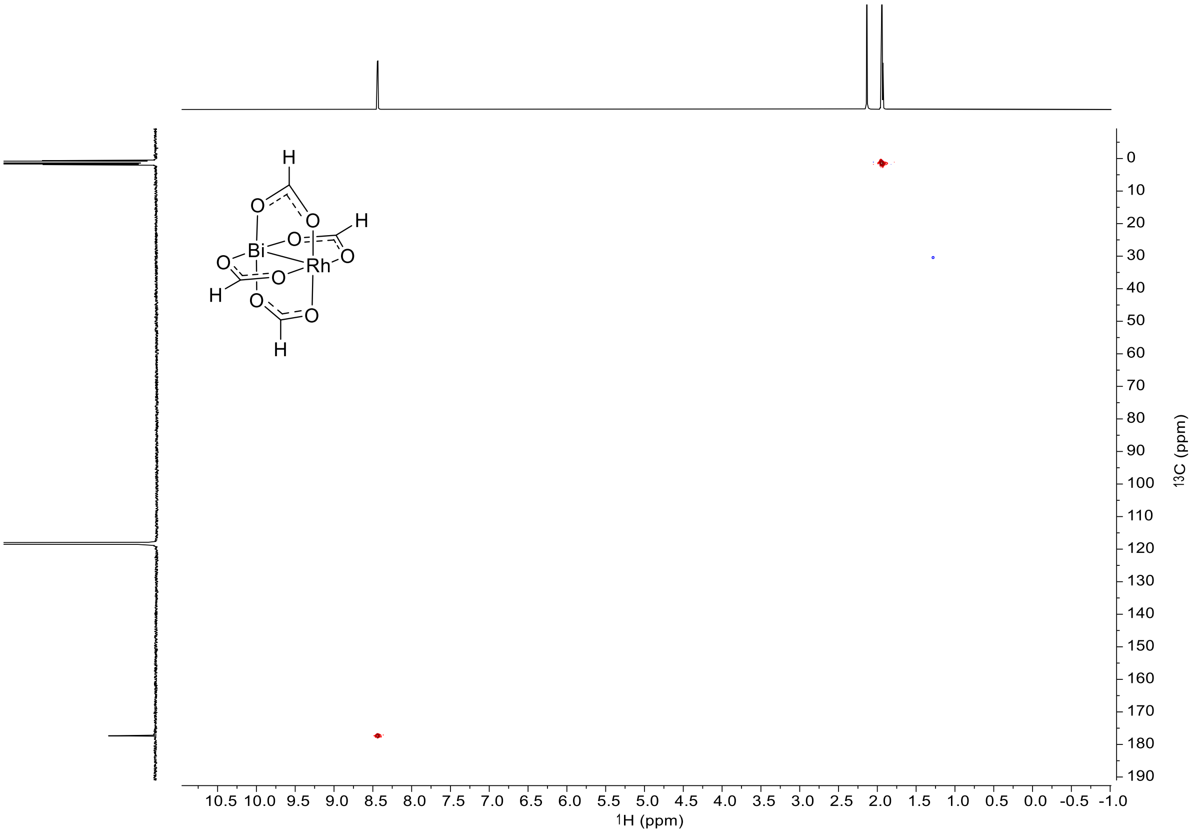


**BiRh(HCO$_2$)$_4$ (21): $^1$H-$^{103}$Rh-HMBC (CD$_3$CN)**

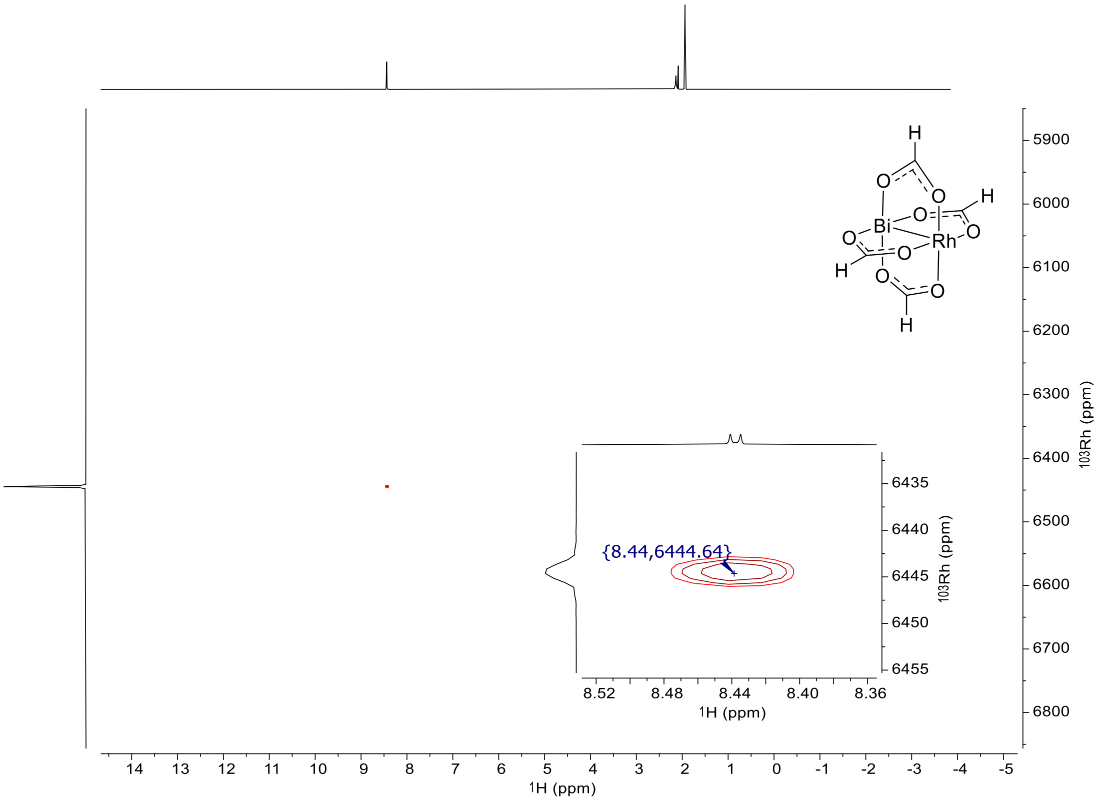

**BiRh(esp)$_2$ (22): $^1$H-NMR (400 MHz, $CD_3CN$)**

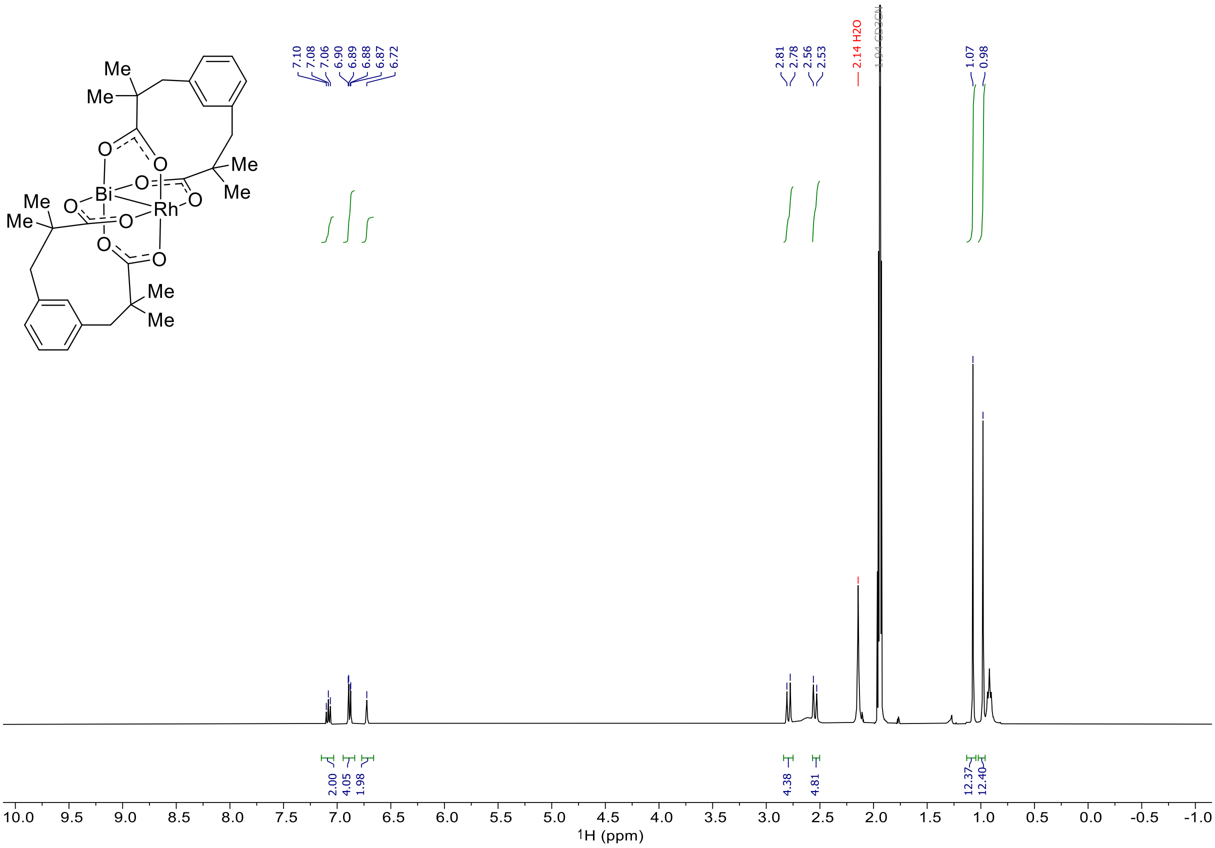


**BiRh(esp)$_2$ (22): $^1$H-NMR (500 MHz, [D$_8$]-THF)**

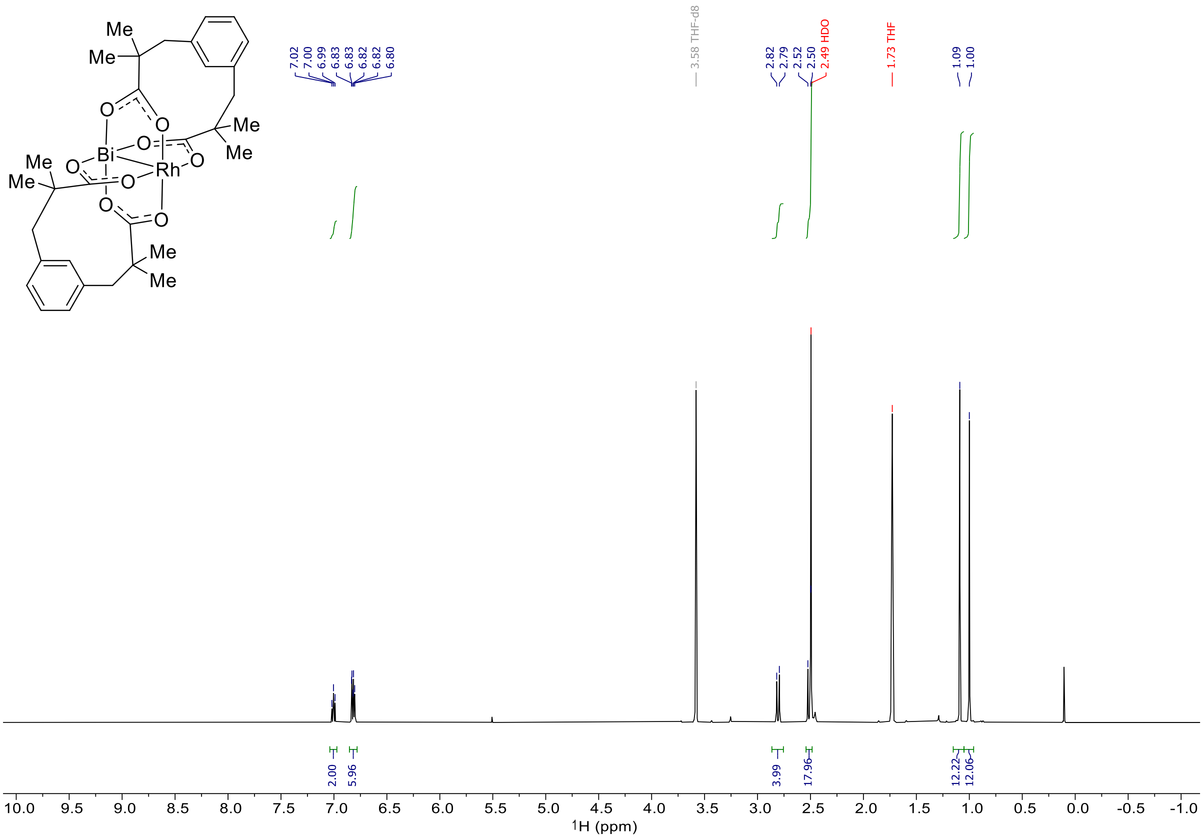

**BiRh(esp)$_2$ (22): H(C)Rh ([D$_8$]-THF)**

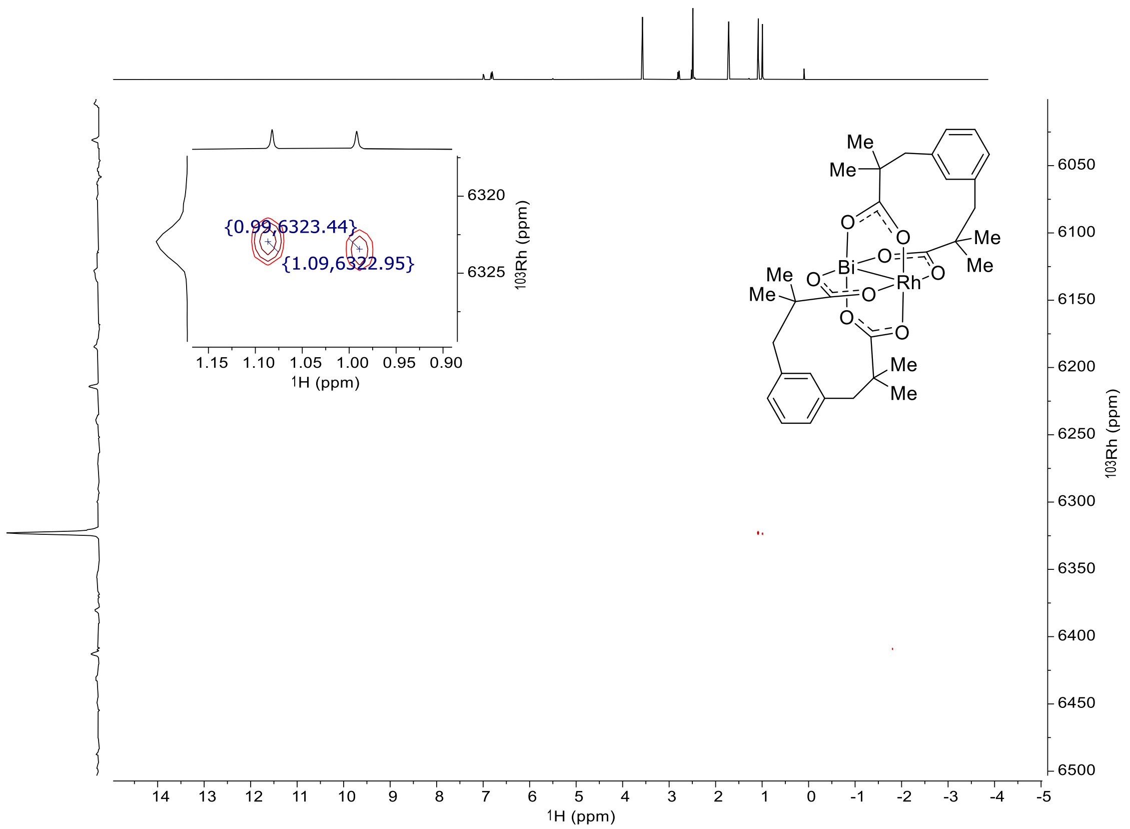

# 7. References


1. Fulmer, G. R.; Miller, A. J. M.; Sherden, N. H.; Gottlieb, H. E.; Nudelman, A.; Stoltz, B. M.; Bercaw, J. E.; Goldberg, K. I., NMR Chemical Shifts of Trace Impurities: Common Laboratory Solvents, Organics, and Gases in Deuterated Solvents Relevant to the Organometallic Chemist. *Organometallics* **2010,** *29* (9), 2176-2179.
2. Harris, R. K.; Becker, E. D.; Menezes, S. M. C. d.; Granger, P.; Hoffman, R. E.; Zilm, K. W., Further conventions for NMR shielding and chemical shifts (IUPAC Recommendations 2008). *Pure Appl. Chem*. **2008,** *80* (1), 59-84.
3. Collins, L. R.; van Gastel, M.; Neese, F.; Fürstner, A., Enhanced Electrophilicity of Heterobimetallic Bi–Rh Paddlewheel Carbene Complexes: A Combined Experimental, Spectroscopic, and Computational Study. *J. Am. Chem. Soc.* **2018,** *140* (40), 13042-13055.
4. Berry, M.; Garner, C. D.; Hillier, I. H.; Macdowell, A. A.; Clegg, W., Crystal structure and u.v. photoelectron spectra of tetrakis-(6-methyl-2-oxopyridinato)dirhodium. *J. Chem. Soc., Chem. Commun.* **1980,** (11), 494-495.
5. Singha, S.; Buchsteiner, M.; Bistoni, G.; Goddard, R.; Fürstner, A., A New Ligand Design Based on London Dispersion Empowers Chiral Bismuth–Rhodium Paddlewheel Catalysts. *J. Am. Chem. Soc.* **2021**.
6. Lou, Y.; Remarchuk, T. P.; Corey, E. J., Catalysis of enantioselective [2+1]-cycloaddition reactions of ethyl diazoacetate and terminal acetylenes using mixed-ligand complexes of the series $Rh_2(RCO_2)_n(L^*_{4-n})$. Stereochemical heuristics for ligand exchange and catalyst synthesis. *J. Am. Chem. Soc.* **2005,** *127* (41), 14223-30.
7. Doyle, M. P.; Bagheri, V.; Wandless, T. J.; Harn, N. K.; Brinker, D. A.; Eagle, C. T.; Loh, K. L., Exceptionally high trans (anti) stereoselectivity in catalytic cyclopropanation reactions. *J. Am. Chem. Soc.* **1990,** *112* (5), 1906-1912.
8. Alvariño, C.; Simond, D.; Lorente, P. M.; Besnard, C.; Williams, A. F., Chains, Necklaces and Weaving Chain-link Grids from Self-Assembly Reactions. *Chem. Eur. J.* **2015,** *21* (24), 8851-8858.
9. Rempel, G. A.; Legzdins, P.; Smith, H.; Wilkinson, G.; Ucko, D. A., Tetrakis(acetato)dirhodium(II) and Similar Carboxylato Compounds. *Inorg. Synth.*, **1972**, 90-91.
10. Pirrung, M. C.; Morehead, A. T., Electronic Effects in Dirhodium(II) Carboxylates. Linear Free Energy Relationships in Catalyzed Decompositions of Diazo Compounds and CO and Isonitrile Complexation. *J. Am. Chem. Soc.* **1994,** *116* (20), 8991-9000.
11. Doyle, M. P.; Zhou, Q.-L.; Simonsen, S. H.; Lynch, V., Dirhodium(II) Tetrakis[alkyl 2-oxaazetidine-4(*S*)-carboxylates]. A New Set of Effective Chiral Catalysts for Asymmetric Intermolecular Cyclopropanation Reactions with Diazoacetates. *Synlett* **1996,** *1996* (07), 697-698.

12. Sunderland, T. L.; Berry, J. F., Expanding the family of heterobimetallic Bi–Rh paddlewheel carboxylate complexes via equatorial carboxylate exchange. *Dalton Trans.* **2016,** *45* (1), 50-55.

13. Smith, M. B., *March's Advanced Organic Chemistry : Reactions, Mechanisms, and Structure 7th Edition*. Wiley: New York, 2001.

14. Mobley, T. A.; Tennyson, E. G.; Hisao, G. S., Indirect detection of the $^{183}$W and $^{57}$Fe nuclei using 119Sn-relayed 1H,X correlation spectroscopy. *Magn. Res. Chem.* **2010,** *48* (10), 787-792.

15. Xiang, B.; Winemiller, M. D.; Briggs, T. F.; Fuller, D. J.; Collum, D. B., Optimizing HMQC for $IS_n$ spin systems. *Magn. Res. Chem.* **2001,** *39* (3), 137-140.

16. Helgaker, T.; Jaszuński, M.; Ruud, K., Ab Initio Methods for the Calculation of NMR Shielding and Indirect Spin−Spin Coupling Constants. *Chem. Rev.* **1999,** *99* (1), 293-352.

17. Lenthe, E. v.; Baerends, E. J.; Snijders, J. G., Relativistic regular two-component Hamiltonians. *The Journal of Chemical Physics* **1993,** *99* (6), 4597-4610.

18. van Lenthe, E.; Baerends, E. J.; Snijders, J. G., Relativistic total energy using regular approximations. *The Journal of Chemical Physics* **1994,** *101* (11), 9783-9792.

19. Sadlej, A. J.; Snijders, J. G.; van Lenthe, E.; Baerends, E. J., Four component regular relativistic Hamiltonians and the perturbational treatment of Dirac's equation. *The Journal of Chemical Physics* **1995,** *102* (4), 1758-1766.

20. Wolff, S. K.; Ziegler, T.; van Lenthe, E.; Baerends, E. J., Density functional calculations of nuclear magnetic shieldings using the zeroth-order regular approximation (ZORA) for relativistic effects: ZORA nuclear magnetic resonance. *The Journal of Chemical Physics* **1999,** *110* (16), 7689-7698.

21. Bouten, R.; Baerends, E. J.; van Lenthe, E.; Visscher, L.; Schreckenbach, G.; Ziegler, T., Relativistic Effects for NMR Shielding Constants in Transition Metal Oxides Using the Zeroth-Order Regular Approximation. *J. Phys. Chem. A* **2000,** *104* (23), 5600-5611.

22. Schreckenbach, G.; Ziegler, T., Calculation of NMR Shielding Tensors Using Gauge-Including Atomic Orbitals and Modern Density Functional Theory. *The Journal of Physical Chemistry* **1995,** *99* (2), 606-611.

23. Wolff, S. K.; Ziegler, T., Calculation of DFT-GIAO NMR shifts with the inclusion of spin-orbit coupling. *The Journal of Chemical Physics* **1998,** *109* (3), 895-905.

24. Hagemann, H. "Computational studies of relativistic effects on NMR shieldings using perturbative approaches for spin-orbit coupling", Master Thesis, HHU Düsseldorf, 2020.

25. Stoychev, G. L.; Auer, A. A.; Izsák, R.; Neese, F., Self-Consistent Field Calculation of Nuclear Magnetic Resonance Chemical Shielding Constants Using Gauge-Including Atomic Orbitals and Approximate Two-Electron Integrals. *J. Chem. Theory Comput.* **2018,** *14* (2), 619-637.

26. Perdew, J. P.; Ruzsinszky, A.; Tao, J.; Staroverov, V. N.; Scuseria, G. E.; Csonka, G. I., Prescription for the design and selection of density functional approximations: More constraint satisfaction with fewer fits. *The Journal of Chemical Physics* **2005,** *123* (6), 062201.
27. Ramsey, N. F., Magnetic Shielding of Nuclei in Molecules. *Physical Review* **1950,** *78* (6), 699-703.
28. Widdifield, C. M.; Schurko, R., Understanding Chemical Shielding Tensors using Group Theory, MO Analysis, and Modern Density-Functional Theory. *Concepts Magn. Reson., Part A* **2009,** *34A*, 91.
29. Becke, A. D., Density-Functional Exchange-Energy Approximation with Correct Asymptotic-Behaviour, *Phys. Rev. A* **1998**, *38*, 3098−3100.
30. Lee, C.; Yang, W.; Parr, R. G., Development of the Colle-Salvetti correlation-energy formula into a functional of the electron density, *Phys. Rev. B* **1998**, *37*, No. 785.
31. Becke, A. D., Density-functional thermochemistry. III. The role of exact exchange. *The Journal of Chemical Physics* **1993,** *98* (7), 5648-5652.
32. Weigend, F.; Ahlrichs, R., Balanced Basis Sets of Split Valence, Triple Zeta Valence and Quadruple Zeta Valence Quality for H to Rn: Design and Assessment of Accuracy. *Phys. Chem. Chem. Phys.* **2005,** *7*, 3297.
33. Weigend, F., Accurate Coulomb-fitting basis sets for H to Rn. *Physical Chemistry Chemical Physics* **2006,** *8* (9), 1057-1065.
34. Barone, V.; Cossi, M., Quantum Calculation of Molecular Energies and Energy Gradients in Solution by a Conductor Solvent Model. *J. Phys. Chem. A* **1998,** *102*, 1995.
35. Wolinski, K.; Hinton, J. F.; Pulay, P., Efficient implementation of the gauge-independent atomic orbital method for NMR chemical shift calculations. *J. Am. Chem. Soc.* **1990,** *112* (23), 8251-8260.
36. London; F., Théorie quantique des courants interatomiques dans les combinaisons aromatiques, *J. Phys. Radium*, **1937**, *8*, 397-409.
37. Hameka; H. F., Gauge-invariant calculation of nuclear magnetic shielding constants at the coupled–cluster singles and doubles level , Mol. Phys., 1958, 1, 203-215.
38. Ditchfield, R., Molecular Orbital Theory of Magnetic Shielding and Magnetic Susceptibility. *The Journal of Chemical Physics* **1972,** *56* (11), 5688-5691.
39. Helgaker, T.; Jørgensen, P., An electronic Hamiltonian for origin independent calculations of magnetic properties. *The Journal of Chemical Physics* **1991,** *95* (4), 2595-2601.
40. Perdew J.P.; Ruzsinszky, A.; Csonka, G. I.; Constantin, L. A.; Sun; J., Workhorse Semilocal Density Functional for Condensed Matter Physics and Quantum Chemistry, *Phys. Rev. Lett.*, **2009**, *103*, 026403.

41. Tao, J.; Perdew, J. P.; Staroverov, V.N.; Scuseria, G. E., Climbing the Density Functional Ladder: Nonempirical MetaGeneralized Gradient Approximation Designed for Molecules and Solids, *Phys. Rev. Lett*. **2003**, *91*, 146401.
42. Staroverov, V. N.; Scuseria, G. E.; Tao, J.; Perdew, J. P., Comparative assessment of a new nonempirical density functional: Molecules and hydrogen-bonded complexes. *The Journal of Chemical Physics* **2003,** *119* (23), 12129-12137.
43. Pantazis, D. A.; Chen, X.-Y.; Landis, C. R.; Neese, F., All-Electron Scalar Relativistic Basis Sets for Third-Row Transition Metal Atoms. *J. Chem. Theory Comput.* **2008,** *4* (6), 908-919.
44. Stoychev, G. L.; Auer, A. A.; Neese, F., Automatic Generation of Auxiliary Basis Sets. *J. Chem. Theory Comput.* **2017,** *13* (2), 554-562.
45. Bouten, R.; Baerends, E. J.; van Lenthe, E.; Visscher, L.; Schreckenbach, G.; Ziegler, T., Relativistic Effects for NMR Shielding Constants in Transition Metal Oxides Using the Zeroth-Order Regular Approximation. *The Journal of Physical Chemistry A* **2000,** *104* (23), 5600-5611.
